# Asymmetric Catalytic Friedel–Crafts Reactions of Unactivated Arenes

**DOI:** 10.1021/jacs.3c05148

**Published:** 2023-07-13

**Authors:** Sebastian Brunen, Benjamin Mitschke, Markus Leutzsch, Benjamin List

**Affiliations:** Max-Planck-Institut für Kohlenforschung, 45470 Mülheim an der Ruhr, Germany

## Abstract

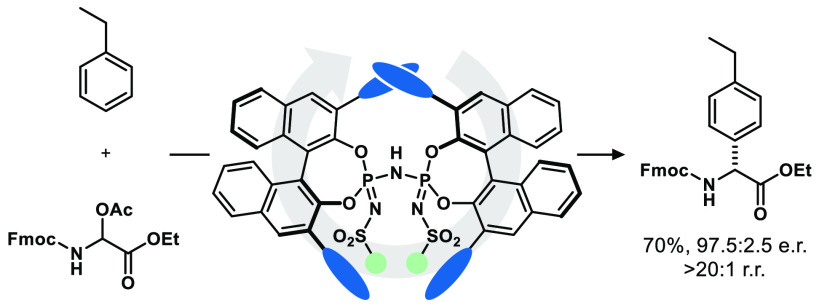

Since its discovery more than a century ago, the Friedel–Crafts reaction has manifested itself as a powerful method for the introduction of carbon substituents to arenes. Despite its potential generality, the scope of the reaction is intrinsically limited by the arene’s nucleophilicity, which has previously restrained the applicability of asymmetric variants to activated substrates. To overcome this fundamental limitation, we report herein an asymmetric Friedel–Crafts reaction of unactivated, purely hydrocarbon arenes, alkoxybenzenes, and heteroarenes with *N*,*O*-acetals to give enantioenriched arylglycine esters. Highly regio- and stereoselective C–C bond formation was achieved using strong and confined Brønsted acid organocatalysts, enabling the first asymmetric catalytic Friedel–Crafts reaction of simple alkylbenzenes.

Electrophilic aromatic substitution reactions are an indispensable tool for the functionalization of aromatic compounds and a cornerstone of the chemical industry.^1,2^ Pioneered by Charles Friedel and James Crafts, the Friedel–Crafts reaction stands out for its straightforward introduction of carbon substituents to arenes without the necessity for prior functionalization.^3−5^ Despite the plethora of possibilities for the selective assembly of carbon scaffolds they offer, Friedel–Crafts reactions suffer from a variety of intrinsic difficulties.^6^ Among others, overriding substrate-inherent regioselectivities by means of catalyst control is a challenging task that is often worsened by harsh reaction conditions. Furthermore, the scope of the Friedel–Crafts reaction is limited by the arene’s nucleophilicity, which is particularly apparent in the construction of benzylic stereocenters. In the past decades, asymmetric catalytic Friedel–Crafts reactions have been investigated extensively.^7−9^ Careful analysis of these methods, however, reveals that the full variety of aromatic molecules is not represented equally throughout the field of asymmetric catalytic Friedel–Crafts reactions: while reactive heterocyclic arenes, especially indoles^10,11^ or pyrroles^12^ are well investigated, less nucleophilic arenes such as naphthols or phenols^13^ find less application and even more inert only hydrocarbon arenes have, to the best of our knowledge, not yet been transformed in an asymmetric fashion (Figure 1A). Overcoming this formidable challenge would enable a shortcut to enantioenriched building blocks and has the potential to streamline chemical processes by improving step-, time-, and atom-efficiencies.^14^

**Figure 1:**
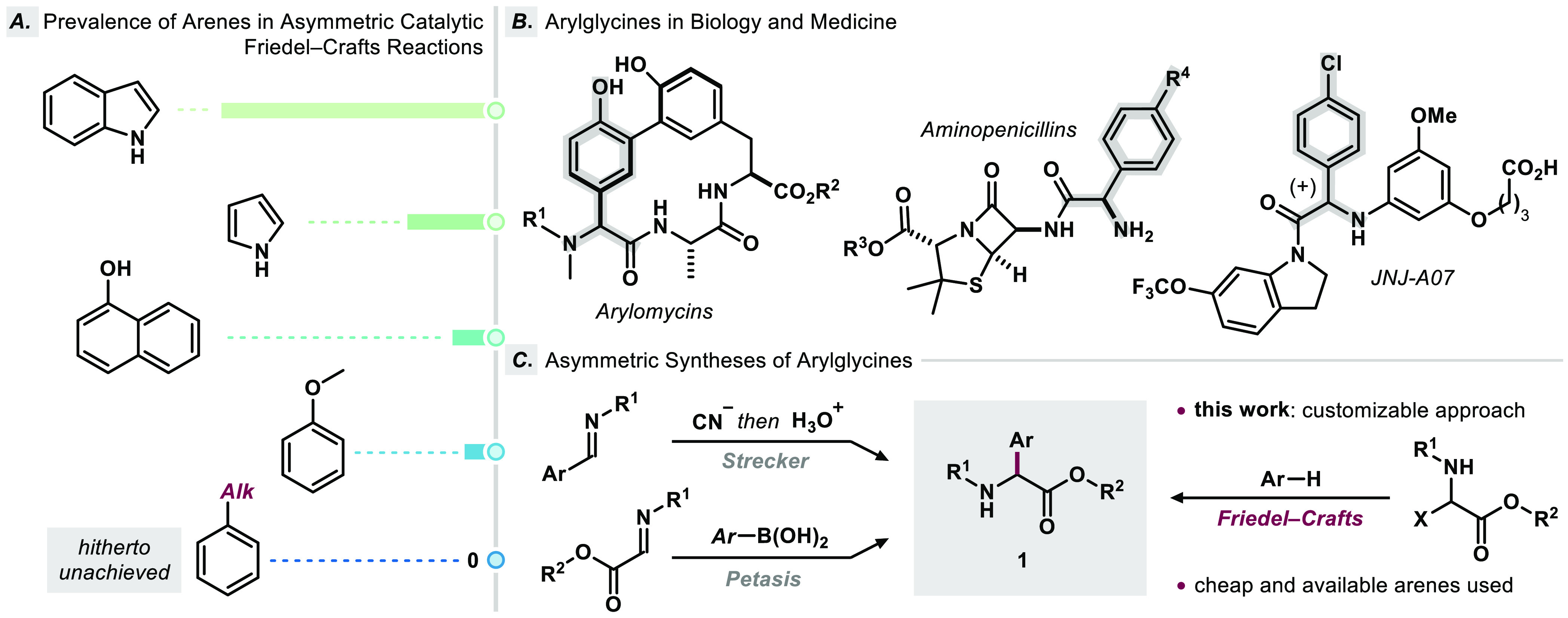
(A) Relative comparison of arenes used in asymmetric catalytic Friedel–Crafts reactions. Data evaluated via https://scifinder-n.cas.org, status as of May 2023. (B) Arylglycines as structural elements in biologically active molecules. (C) Synthetic methods for the preparation of enantioenriched arylglycines **1**.

Arylglycines represent central units in a multitude of biologically active compounds (Figure 1B).^15−20^ Traditionally, these non-canonical amino acids are accessible through various synthetic approaches (Figure 1C). Especially in the past decades, asymmetric Strecker-^21−24^ and Petasis-Mannich^25−28^ procedures have shown to be successful strategies in the stereoselective assembly of arylglycines—both approaches, however, rely on prefunctionalized arenes. The direct transformation of cheap and readily available arenes represents an efficient alternative.^29−38^ The reported methods are typically limited to a reduced scope and often require electronically biased arenes or synthetically disadvantageous protecting groups, though. In the context of recent studies in our laboratory to transforming unfunctionalized early stage chemical feedstocks to valuable building blocks,^39^ we report herein the synthesis of enantioenriched arylglycines in the first asymmetric catalytic Friedel–Crafts reaction of simple alkylbenzene arenes.

We initiated our investigations using toluene (**3a**) as representative substrate along with *N*,*O*-acetal **2** as electrophilic reagent^40,41^ in the presence of Brønsted acid organocatalysts under neat reaction conditions (Table 1). Moderately acidic chiral phosphoric acid (CPA, p*K*_a_ ∼ 13 in MeCN) catalysts and the more acidic imidodiphosphate (IDP, p*K*_a_ ∼ 11 in MeCN) or iminoimidodiphosphate (iIDP, p*K*_a_ ∼ 9 in MeCN)-catalysts failed to form the desired product **1**.^42^ Also highly acidic imidodiphosphorimidates (**4**, IDPi) initially did not promote the desired bond formation. However, exchange of the 3,3′-phenyl-substituents of the chiral BINOL backbone (p*K*_a_ = 4.5 in MeCN)^43^ to the more electron deficient 3,5-(CF_3_)_2_C_6_H_3_-modified motif (estimated p*K*_a_ ∼ 2 in MeCN)^42^ eventually led to the formation of the desired arylglycine **1a** in moderate yield, good enantioselectivity, and excellent *para*-selectivity (>20:1 r.r.). While exchange of the *N*-Cbz- to an *N*-Boc-protecting group on reagent **2** completely shut down the reactivity, installation of an *N*-Fmoc-group not only restored reactivity but also significantly improved enantioselectivity. Due to the described effect of the Fmoc-protecting group along with its general synthetic utility, we chose electrophile **2c** for further investigations. Optimization of the catalyst finally revealed that a 3,5-(SF_5_)_2_-C_6_H_3_ group in the BINOL’s 3,3′-position along with a C_2_F_5_-chain in the catalyst’s imidodiphosphorimidate core (catalyst **4g**) gave superior reactivity along with high enantiocontrol. It is worth mentioning that the highly reactive nature of the electrophile **2** can lead to cleavage of catalyst **4b**’s imidodiphosphorimidate core under the reaction conditions described above. Using optimized catalyst **4g**, however, this undesired process could be suppressed to a minimal level (see Section 9 of the Supporting Information).

**Table 1:**
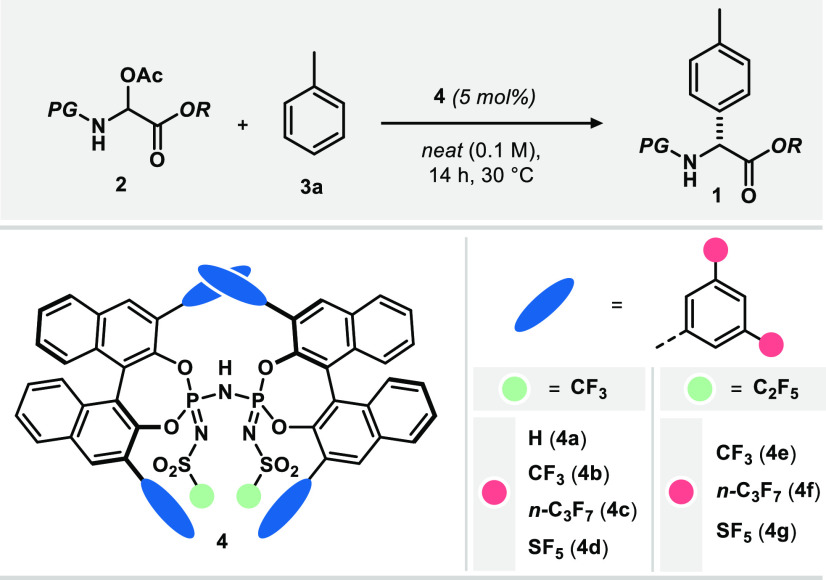
Reaction Development

entry^a^	catalyst	PG	*R*	yield (%)^b^	e.r.^c^	r.r.^d^
1	**4a**	Cbz	Et (**2a**)	<5	n.d.	n.d.
2	**4b**	Cbz	Et (**2a**)	50	82:18	20:1
3	**4b**	Boc	Et (**2b**)	<5	n.d.	n.d.
4	**4b**	**Fmoc**	Et (**2c**)	42	***90:10***	>20:1
5	**4b**	Fmoc	*i*Pr (**2d**)	44	90:10	>20:1
6	**4c**	Fmoc	Et (**2c**)	29	94:6	>20:1
7	**4d**	Fmoc	Et (**2c**)	36	90.5:9.5	>20:1
8	**4e**	Fmoc	Et (**2c**)	55	92.5:7.5	>20:1
9	**4f**	Fmoc	Et (**2c**)	15	95.5:4.5	>20:1
10	**4g**	Fmoc	Et (**2c**)	**70**	***93:7***	>20:1
11^e^	**4g**	Fmoc	Et (**2c**)	**55**	***96:4***	>20:1

a Reactions were performed with *N*,*O*-acetal **2** (25 μmol) in toluene (0.25 mL) using (*S,S*)-IDPi catalysts **4** (5 mol %) at 30 °C under argon atmosphere.

b Yields determined via ^1^H NMR using dimethyl sulfone as internal standard.

c Determined via HPLC analysis.

d For the *para* regioisomer, determined via ^1^H NMR or HPLC.

e Performed at 15 °C over 5 d.

With the optimized conditions for the transformation of toluene (**3a**) in hand, we went on to investigate the scope of alkylbenzene substrates applicable in the reaction (Figure 2A). Elongation of the carbon chain on the alkylbenzene side was found to be compatible: Et- (**3b**), *n*-Pr- (**3c**), and *n*-Bu-benzene (**3d**) could be transformed to the corresponding arylglycine esters with moderate to good yields and excellent enantiomeric ratios (Figure 2A). Installation of branched alkyl chains and small rings appeared to be well tolerated, yielding products **1e**–**1g** with slightly reduced yields and similar, excellent enantiomeric ratios. *o*-Xylene (**3h**) and 1,2-diethylbenzene (**3i**) were found to be markedly more reactive, and excellent enantioselectivity was retained in both cases. Aiming to determine the method’s limitations with respect to the arene’s nucleophilicity, benzene (**3j**) was treated with substrate **2c** in the presence of catalyst **4h**, bearing an extended perfluoroalkyl core modification. Under harsher reaction conditions, phenylglycine **1j** was obtained in moderate yield and enantioenrichment.

**Figure 2:**
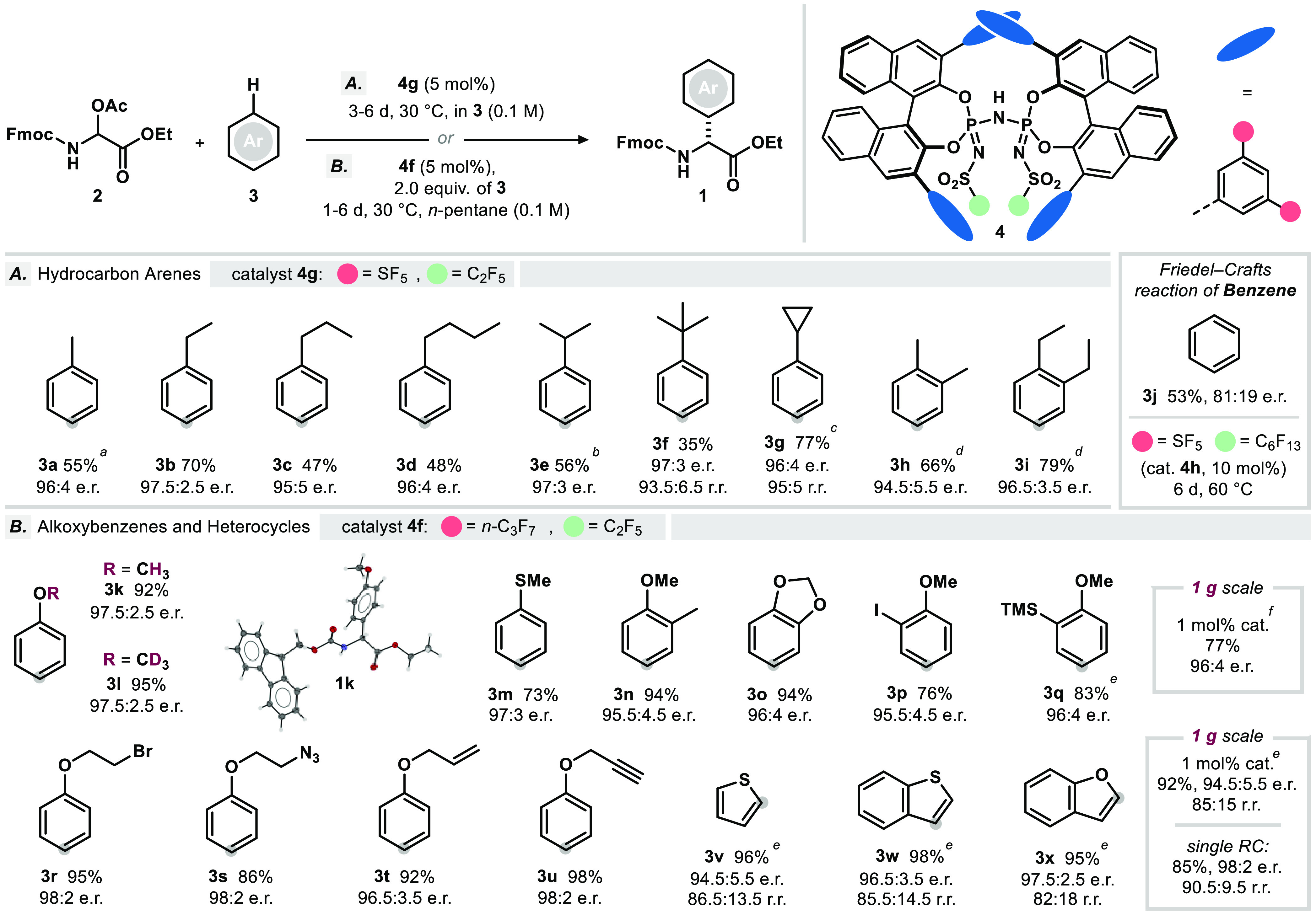
Substrate scope. Yields are those of isolated product **1** after chromatography. Reactions were performed on a 100 μmol scale referring to *N*,*O*-acetal **2c**, regioisomeric ratio >20:1 for the indicated position (grey sphere) if not stated differently. (A) Scope of purely hydrocarbon substrates. (B) Scope of alkoxybenzenes and heteroarenes. *a* performed at 15 °C. *b* = catalyst **4f** (5 mol %) used. *c* = performed at 20 °C. *d* = performed at 0 °C. *e* = in CyMe (0.1 M). *f* = in *n*-hexanes (0.1 M). For detailed reaction conditions, see the Supporting Information.

To explore the full synthetic range of our method, we set out to study the reaction of a more general scope of alkoxybenzenes and heterocyclic arenes (Figure 2B). To our delight, a diverse range of alkoxybenzenes bearing alkyl, halide-, alkenyl-, or alkynyl-residues and azide- or silyl groups could be transformed with excellent yields and enantiomeric ratios following a slightly modified protocol. Intriguingly, 2-trimethylsilyl-anisole (**3q**) smoothly underwent conversion to the desired product **1q**. In contrast, using bistriflimide as a Brønsted acid catalyst yielded protodesilylated arylglycine **1k**, highlighting the ability of the confined microenvironment along with the carefully adjusted acidity of IDPi catalysts to instill chemoselectivity.

The *O*- and *S*-heterocyclic arenes thiophene (**3v**), benzothiophene (**3w**), and benzofuran (**3x**) were transformed analogously to yield corresponding heteroarylglycines with high yields and enantiomeric ratios. Notably, upon conversion of these heterocyclic substrates as described above, only reduced levels of regiocontrol could be observed (see Figure 2B). The transformation was furthermore found to be easily scalable, as demonstrated in the reaction of benzofuran (**3x**) and 2-trimethylsilylanisole (**3q**) on a gram scale, yielding corresponding products **1x** and **1q** with good yields and excellent enantiomeric ratios.

The direct transformation of unactivated hydrocarbon arenes in Friedel–Crafts pathways usually requires comparatively harsh reaction conditions to overcome higher activation barriers.^44,45^ Since high regio- and enantioselectivities could still be obtained in the reaction described herein, we were interested in the elucidation of the mechanism at hand. Our initial interest was focused on the observation that exclusively our most acidic IDPi catalysts **4b**–**g** were found to deliver the desired products **1a**–**x**, while structurally related representatives of type **4a** failed to do so. To evaluate the catalysts’ general capability to activate electrophile **2c**, we subjected catalysts **4a** and **4b** to reaction with electrophile **2c** in the presence of acetic acid-*d*_4_ (Figure 3A). While only small amounts of acetate-*d*_3_ incorporation were observed using catalyst **4a**, an equilibrium between **2c** and **2c-*d***_**3**_ is reached within 1 h using catalytically active IDPi **4b**. Supported by earlier reports,^46^ we propose the equilibration of **4b** and **2c** toward ion pair **I** via release of acetic acid. Insufficiently acidic catalyst **4a** seems to be significantly less capable of promoting iminium ion formation. Addition of acetic acid should consequentially shift the equilibrium, reduce the effective concentration of ion pair **I**, and therefore slow down the reaction. Indeed, the addition of acetic acid to the reaction of anisole (**1k**) leads to a significant drop of the reaction rate, confirming its potent inhibitory properties for the reaction (Figure 3B).

**Figure 3:**
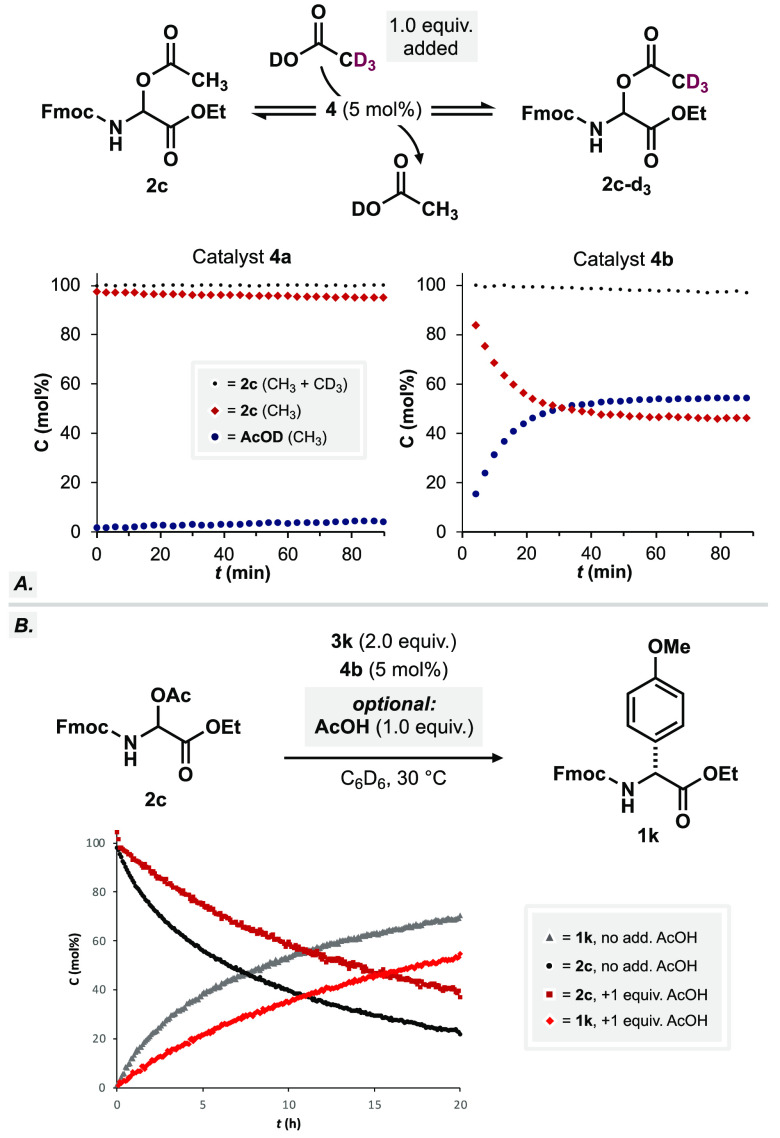
(A) AcO-*h*_3_/AcO-*d*_3_ equilibration experiments. (B) Inhibition studies used additional AcOH.

We furthermore determined a kinetic isotope effect (KIE) of 1.55 ± 0.04 in a direct competition experiment between toluene-*h*_8_ and toluene-*d*_8_ (see Supporting Information for experimental details and discussion). Based on these mechanistic experiments, we propose a fast upstream equilibration of electrophile **2c** and catalyst **4** toward ion pair **I** (Figure 4A). This step is then followed by the nucleophilic attack of arene **3** to form Wheland-type intermediate **II**. The subsequent rearomatization releases product **1** and closes the catalytic cycle. As indicated by the measured KIE, rearomatization is expected to be at least partially rate determining.^47−49^ The overall KIE might additionally be influenced by a preceding equilibrium isotope effect (EIE) originating from the reversible formation of complex **II**, which could be additionally stabilized by the electron rich carbamate moiety (as depicted in Figure 4A).^50^

**Figure 4:**
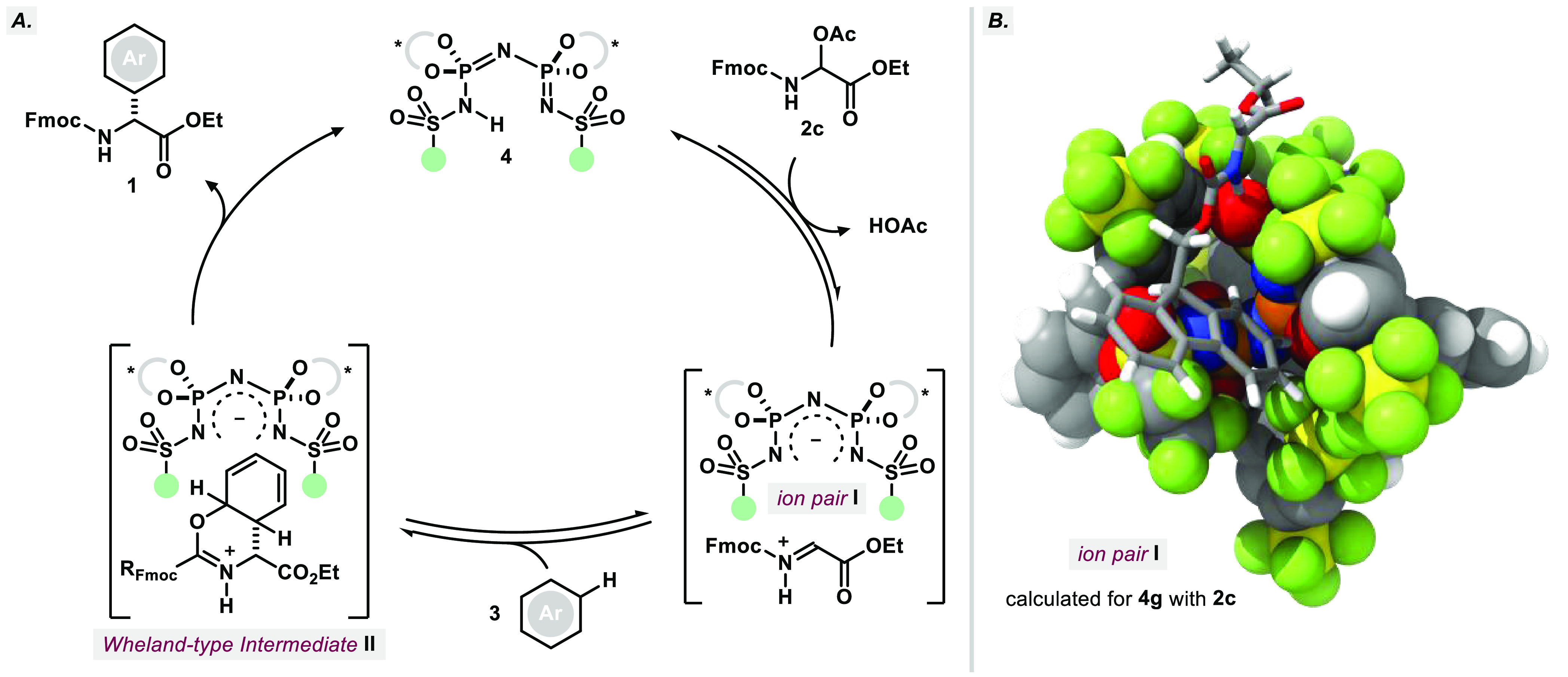
(A) Proposed catalytic cycle for the IDPi catalyzed Friedel–Crafts reaction of *N*,*O*-acetals **2** and aromatic substrates. (B) DFT-calculated structure of ion pair **I** derived from *N*,*O*-acetal **2c** and IDPi **4g** (for further details, see the Supporting Information).

To further investigate the factors governing stabilization of the proposed iminium ion, we chose to investigate the ion pair **I** resulting from reaction of **2c** with optimized IDPi **4g** computationally; the resulting DFT structure of the lowest energy conformer is depicted below (Figure 4B, see the Supporting Information for additional computational details). Key interaction clearly is a tight hydrogen bond of the iminium NH to the pentafluoroethylsulfonyl core (1.54 Å). Interestingly, a second hydrogen bond (2.36 Å) from the bis-benzylic proton of **2c**’s fluorenyl portion seems to aid in conformational locking within the confined cavity, supported by additional π–π stacking interactions of the aromatic system with one of the 3,5-(SF_5_)_2_-C_6_H_3_ groups. This computational finding is consistent with the observed increase in enantioselectivity when switching from Cbz to Fmoc carbamates (Table 1). Moreover, the ester functionality protrudes from the chiral pocket of IDPi **4**, which solidifies the experimentally observed independence of enantioselectivity on the size of the ester’s alkyl group (Table 1).

In accordance with our inhibition studies, progressive release of acetic acid originating from **2c** should slow down and potentially impede the Friedel–Crafts reaction, which is particularly evident for less reactive arenes at higher levels of conversion. Simultaneously, modification of the electrophile’s leaving group or *in situ* removal of released acetic acid might lead to an increased concentration of ion-pair **I** and therefore permit the selective transformation of even less reactive aromatic substrates. Studies on this matter are currently being carried out in our laboratory.

In summary, an asymmetric catalytic Friedel–Crafts reaction of *N*,*O*-acetal-electrophiles **2** toward enantioenriched α-arylglycines **1** has been developed. Excellent enantio- and regioisomeric ratios could be achieved using strong and confined imidodiphosphorimidate organocatalyst **4** in the first asymmetric catalytic Friedel–Crafts reaction of simple hydrocarbon arenes. Extension of the scope to alkoxybenzenes and heteroarenes enabled the synthesis of a broad variety of functionalized aryl- and heteroarylglycines in a single step from bench-stable *N*,*O*-acetal **2c**. Investigations using benzene (**3j**) as an aromatic substrate allowed initial advances into the realm of asymmetric catalytic Friedel–Crafts reactions of even less reactive arenes. Further studies to expand the boundaries within the field of selective electrophilic aromatic substitutions of minimally nucleophilic arenes are currently ongoing in our laboratory.

## Supporting Information Available

Experimental details, computational data, compound characterization (PDF)

## Abbreviations

IDPi: imidodiphosphorimidate
CPA: chiral phosphoric acid
IDP: imidodiphosphate
iIDP: iminoimidodiphosphate
BINOL: 1,1′-bi-2-naphtol
Boc: *tert*-butyloxycarbonyl
Cbz: benzyloxycarbonyl
Fmoc: fluorenylmethyloxycarbonyl
TMS: trimethylsilyl
Et: ethyl
Me: methyl
*i*Pr: isopropyl
Cy: cyclohexyl
KIE: kinetic isotope effect
e.r.: enantiomeric ratio
r.r.: regioisomeric ratio
DFT: density functional theory
RC: recrystallization
Ar: aryl
Alk: alkyl.
