# Supplementary material for: Asymmetric Catalytic Friedel–Crafts Reactions of Unactivated Arenes
Source: J Am Chem Soc. 2023 Jul 13;145(29):15708–13. doi: 10.1021/jacs.3c05148 (PMC10375537; doi:10.1021/jacs.3c05148)
Supplement: Supplementary file 1 — ja3c05148_si_001.pdf [file ja3c05148_si_001.pdf]

Supporting Information for

Asymmetric Catalytic Friedel–Crafts Reactions of Unactivated Arenes

Sebastian Brunen, Benjamin Mitschke, Markus Leutzsch and Benjamin List*

Max-Planck-Institut für Kohlenforschung, Kaiser-Wilhelm-Platz 1, 45470 Mülheim an der Ruhr, Germany
*E-mail: list@kofo.mpg.de

1. Materials and Methods 1
2. Synthesis of Substrates and Reagents 3
   2.1 Synthesis of *N,O*-Acetals 3
   2.2 Synthesis of Arene Substrates 10
3. Synthesis of (*S,S*)-IDPi Catalysts 11
   3.1 Synthesis of substituted (*S*)-BINOLs 11
   3.2 Synthesis of (*S,S*)-IDPi Catalysts from 3,3'-disubstituted BINOLs 13
4. Reaction Development 16
   4.1 Hydrocarbon Arene Substrates 16
   4.2 Anisoles and Heteroaromatic Substrates 17
5. Asymmetric Friedel–Crafts Reactions toward Arylglycines 19
   5.1 Friedel–Crafts Reaction of Only-Hydrocarbon Arenes 20
   5.2 Friedel–Crafts Reaction of Anisoles and Heteroarenes 27
   5.3 Friedel–Crafts Reaction with Differing N,O-Acetals 39
6. Gram-Scale Synthesis of Arylglycine Derivates 42
7. Determination of the Absolute Configuration of Arylglycine Products 44
8. Mechanistic Investigations 46
   8.1 Reactivity Assessment via Acetate Scrambling Experiments 46
   8.2 Investigation of Inhibitory Effects of Acetic Acid 49
   8.3 Kinetic Isotope Effect Studies 52
9. Catalyst Stability Studies 59
10. Computational Methods 61
   10.1 Calculated Structure of Iminium Ion Pair I 62
   10.2 Molecular Electrostatic Potential 63
   10.3 XYZ Structures 64
11. NMR Spectra 68
12. HPLC Traces 121
13. Crystallographic Data 154
References 162

1. Materials and Methods

Chemicals: Unless otherwise indicated, starting materials were obtained from Sigma-Aldrich, ABCR-GmbH, TCI, Acros Co. Ltd., Fluorochem or Deutero GmbH. Commercially available substances were used without further purification.

Solvents: Solvents (Et_2O, THF, 1,4-Dioxane, Cyclohexane, CH_2Cl_2, $CHCl_3$, PhH and PhMe) were dried by distillation from appropriate drying agents in the technical department of the Max-Plack-Institut für Kohlenforschung and obtained in Schlenk flasks under argon atmosphere. Further solvents (CyMe, o-xylene, *n*-hexanes) were obtained from commercial suppliers and stored under an atmosphere of argon in a Schlenk tube.

Inert Gas: Anhydrous argon was purchased from Air Liquide with >99.5% purity.

Thin Layer Chromatography: Thin layer chromatography (TLC) was performed using silica gel pre-coated plastic sheets (Polygram SIL G/UV254, 0.2 mm, with fluorescent indicator; Macherey-Nagel) which was visualized with a UV lamp (254 nm) and/or phosphomolybdic acid (PMA), and/or Cerium Ammonium Molybdate (CAM), and/or ninhydrin. PMA stain: PMA (20 g) in EtOH (200 mL). CAM stain: Ammonium molybdate tetrahydrate (2.5 g), Cerium ammonium sulfate dihydrate (1 g) and Sulfuric acid (10 mL) in Water (90 mL). Ninhydrin stain: ninhydrin (1.5 g) in EtOH (200 mL) with AcOH (3 mL).

Column Chromatography: Column chromatography was carried out using Merck silica gel (60 Å, 230−400 mesh, particle size 0.040−0.063 mm) using technical grade solvents. Elution was accelerated using compressed air. All reported yields, unless otherwise specified, refer to spectroscopically and chromatographically pure compounds.

Nomenclature: Nomenclature follows the suggestions proposed by the computer program ChemBioDraw (12.0.3.1216) of CBD/Cambridgesoft.

Nuclear Magnetic Resonance Spectroscopy: ^{1}H, ^{13}C, ^{19}F, ^{31}P Nuclear magnetic resonance (NMR) spectra for compound characterization were recorded on a Bruker Avance III 500 or a Bruker Avance Neo 600 MHz NMR spectrometer in a suitable deuterated solvent unless specified otherwise. The solvent employed and the respective measuring frequency are indicated for each experiment. Chemical shifts are reported with tetramethylsilane (TMS) serving as a universal reference of all

nuclides. The resonance multiplicity is described as s (singlet), d (doublet), t (triplet), q (quadruplet), p (pentet), h (heptet), m (multiplet), and br (broad). All spectra were recorded at 298 K unless specified differently, processed with MestReNova 14.1.2 suits of program, and coupling constants are reported as observed. The residual deuterated solvent signal relative to tetramethylsilane was used as the internal reference in 1H NMR spectra (e.g. $CDCl_3$ = 7.26 ppm, CD_2Cl_2 = 5.32 ppm). Signals are reported as follows: chemical shift δ in ppm (multiplicity, coupling constant J in Hz, number of protons). All X-nuclei spectra were acquired proton decoupled unless otherwise noted. $^{13}C\{^{1}H, ^{19}F\}$ NMR spectra were acquired with a Bruker TBO probe (^{1}H, ^{19}F, BB) with inverse gated decoupling. For ^{1}H waltz16 was used for decoupling. For ^{19}F the decoupling scheme bi_p5m4sp_4sp.2 with adiabatic chirp pulses with an offset at -105 ppm was used to ensure the broadband decoupling on ^{19}F.

Mass Spectrometry: Electrospray ionization (ESI) mass spectrometry was conducted on a Bruker ESQ 3000 spectrometer. High resolution mass spectrometry (HRMS) was performed on a Finnigan MAT 95 (EI) or Bruker APEX III FTMS (7 T magnet, ESI). The ionization method and mode of detection employed is indicated for the respective experiment and all masses are reported in atomic units per elementary charge (m/z) with an intensity normalized to the most intense peak.

Specific Rotations: Specific rotations ($\alpha_{\mathrm{D}}^{\mathrm{T}}$) were measured with a Rudolph RA Autopol IV automatic polarimeter at the indicated temperature with a sodium lamp (sodium D line, λ = 589 nm). Measurements were performed in an acid resistant 1 mL cell (50 mm length) with concentrations (g/(100 mL)) reported in the corresponding solvent.

High Performance Liquid Chromatography: High performance liquid chromatography (HPLC) was performed on a Shimadzu LC-20AD liquid chromatograph SIL-20AC auto sampler, CMB-20A using Daicel/Merck columns with a chiral stationary phase. All solvents used were HPLC-grade solvents purchased from Sigma-Aldrich. The column employed and the respective solvent mixture are indicated for each experiment.

Abbreviations: e.r. = enantiomeric ratio, TLC = thin layer chromatography, Boc = *tert*-butyloxycarbonyl, Cbz = benzyloxycarbonyl, Fmoc = fluorenylmethyloxycarbonyl, PG = protecting group, Me = methyl, Et = ethyl, *n*-Pr = *n*-propyl, *i*-Pr = isopropyl, *n*-Bu = *n*-Butyl, TMS = trimethylsilyl, GP = general procedure, IDPi = imidodiphosphorimidate, *i*IDP = imino-imidodiphosphate, IDP = imidodiphosphate, DSI = disulfonimide, CPA = chiral phosphoric acid, KIE = kinetic isotope effect, RC = recrystallization.

2. Synthesis of Substrates and Reagents

2.1 Synthesis of *N,O*-Acetals

General Procedure A (GP A): Synthesis of Hemiaminals

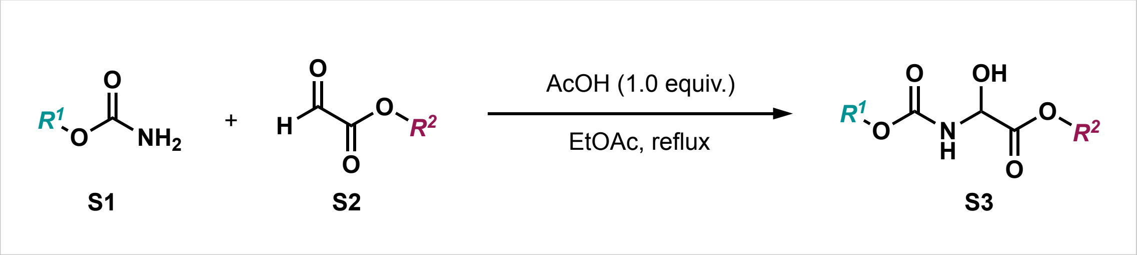

Following a procedure reported by Jacobsen[1], in an oven-dried 500 mL two-neck flask equipped with a reflux condenser, the respective carbamate **S1** (14.2 mmol, 1.0 equiv.) was suspended in EtOAc (200 mL) and alkyl glyoxylate **S2** (20.1 mmol, 1.4 equiv., added pure or as solution in PhMe) was added. AcOH (14.2 mmol, 0.8 mL, 1.0 equiv.) was added dropwise under vigorous stirring and the mixture was heated to reflux for 24 to 48 h. When complete conversion of the carbamate was observed via TLC and/or NMR analysis, the mixture was cooled to room temperature and concentrated under reduced pressure. The obtained white residue was dissolved in a minimal amount of warm CH_2Cl_2 (approximately 100 to 200 mL, warmed to 30 °C) and an equal volume of *i*-hexanes was added slowly under swirling of the flask. The solution was kept at room temperature for two hours whereupon precipitation initiates. The mixture was then cooled to –20 °C overnight and the formed solids were filtered off, washed with *i*-hexanes and dried under vacuum to yield the desired hemiaminals **S3** as white solids.

General Procedure B (GP B): Synthesis of *N,O*-Acetals

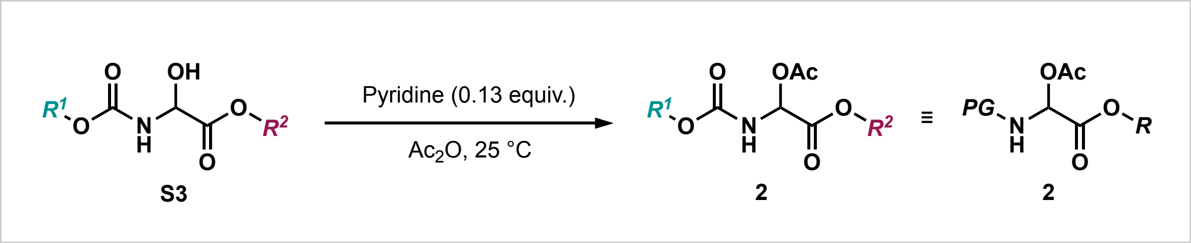

Following a modified procedure reported by Luo[2], hemiaminal **S3** (10 mmol, 1.0 equiv.) was given to a flame dried Schlenk tube followed by acetic anhydride (30 mL) and pyridine (1.3 mmol, 0.10 mL, 0.13 equiv.). The heterogenous mixture was stirred for 20 h under an atmosphere of argon whereupon complete dissolution of the solids was observed. The solution was then concentrated under reduced pressure at 70 °C and the residue was purified via flash column chromatography on silica gel (eluent: *i*-hexanes/EtOAc mixtures) or via recrystallization from CH_2Cl_2/*i*-hexanes to yield the *N,O*-acetals **2** as colorless oils or white solids.

methyl 2-((((9H-fluoren-9-yl)methoxy)carbonyl)amino)-2-hydroxyacetate (S3-a)

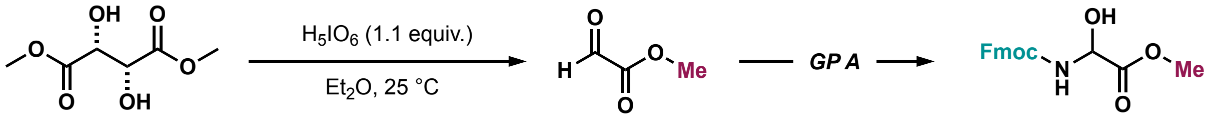

S4-a S2-a S3-a

methylglyoxylate (S2-a): Following modified procedure reported by Yus,[3] dimethyl (2*R*,3*R*)-2,3-dihydroxysuccinate (**S4-a**, 1.9 g, 10.7 mmol, 1.0 equiv.) was given to a 50 mL round bottom flask and dissolved in Et_2O (20 mL). H_5IO_6 (2.67 g, 11.7 mmol, 1.1 equiv.) was added slowly, the flask was sealed and the mixture was stirred at 25 °C for 2 h. The solids were filtered off, washed with EtOAc (3 x 40 mL) and the filtrate was dried over $MgSO_4$. The drying agent was filtered off and the solvent was removed under reduced pressure to yield the crude glyoxylate **S2-a** as colorless oil. The spectral data are in agreement with the reported literature[4]. The compound was directly used in the subsequent step.

**^{1}H-NMR:**	(501 MHz, $CDCl_3$): δ = 9.41 (s, 1H), 3.94 (s, 3H).

methyl 2-((((9H-fluoren-9-yl)methoxy)carbonyl)amino)-2-hydroxyacetate (S3-a): Following GP A, in an oven-dried 250 mL two-neck flask, (9H-fluoren-9-yl)methyl carbamate (**S1-a**, 1.81 g, 7.57 mmol, 1.0 equiv.) was suspended in EtOAc (125 mL) and freshly prepared methylglyoxylate (**S2-a**, 1.00 g, 11.4 mmol, 1.5 equiv.) was added. AcOH (0.43 mL, 7.57 mmol, 1.0 equiv.) was added dropwise under vigorous stirring and the mixture was heated to reflux for 48 h. The mixture was cooled to room temperature and concentrated under reduced pressure. The obtained white residue was dissolved in warm CH_2Cl_2 (100 mL, warmed in a 30 °C water bath) and an equal volume of *i*-hexanes was added slowly under swirling of the flask. The solution was kept at room temperature for two hours whereupon initial precipitation was observed. The mixture was then cooled to –20 °C overnight, the formed solids were filtered off, washed with *i*-hexanes and dried under vacuum to yield the desired hemiaminal **S3-a** (1.51 g, 8.78 mmol, 53% over two steps) as white solid.

**TLC:**	R_F (*i*-hexanes/EtOAc 2:1) = 0.36.
**^{1}H-NMR:**	(501 MHz, $CDCl_3$): δ = 7.77 (d, J = 7.5 Hz, 2H), 7.58 (d, J = 7.5 Hz, 2H), 7.40 (t, J = 7.4 Hz, 2H), 7.31 (t, J = 7.4 Hz, 2H), 6.06 (s, br, 1H), 5.50 (s, br, 1H), 4.46–4.40 (m, 2H), 4.22 (t, J = 6.9 Hz, 1H), 4.08 (s, br, 1H), 3.83 (s, 3H).
**^{13}C-NMR:**	(126 MHz, $CDCl_3$): δ = 169.82, 155.76, 143.69, 143.64, 141.45, 127.96, 127.26, 125.12, 120.19, 73.70, 67.58, 53.46, 47.09.
**ESI-HRMS:**	calculated for $C_{18}H_{17}NNaO_5$ ($[M+Na]^+$): 350.10044, found: 350.09972.

isopropyl 2-((((9H-fluoren-9-yl)methoxy)carbonyl)amino)-2-hydroxyacetate (S3-b)

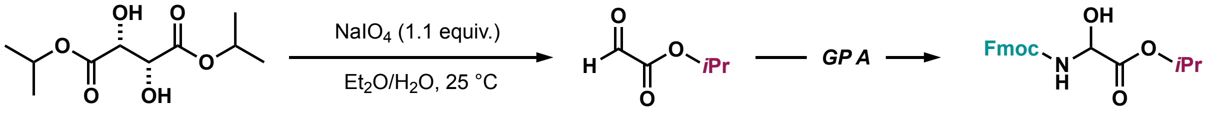

S4-b S2-b S3-b

isopropyl 2-oxoacetate (S2-b): Following a modified procedure reported by Xu and Loh,[5] diisopropyl (2*R*,3*R*)-2,3-dihydroxysuccinate (**S4-b**, 2.5 g, 10.7 mmol, 1.0 equiv.) was given to a 50 mL round bottom flask, dissolved in Et_2O (5 mL) and the solution was cooled to 0°C. A solution of $NaIO_4$ (2.97 g, 13.9 mmol, 1.3 equiv.) in H_2O (20 mL) was added dropwise over 20 min, the flask was sealed and the mixture was stirred at 0 °C for 2 h. The mixture was then warmed to 25 °C and EtOAc (20 mL) was added. The layers were separated and the aqueous layer was extracted with EtOAc (5 x 30 mL). The combined organic extracts were dried over Na_2SO_4, the solids were filtered off and the filtrate was concentrated under reduced pressure to yield the crude glyoxylate **S2-b** as colourless oil. The crude product was directly used in the next step without further purification. The spectral data are in agreement with the reported literature.[6]

^{1}H-NMR: (501 MHz, $CDCl_3$): δ = 9.38 (s, 1H), 5.12 (sept, J = 6.4 Hz, 1H), 1.30 (d, J = 6.3 Hz, 6 H).

methyl 2-((((9H-fluoren-9-yl)methoxy)carbonyl)amino)-2-hydroxyacetate (S3-b): Following GP A, in an oven-dried 250 mL two-neck flask, (9H-fluoren-9-yl)methyl carbamate (**S1-a**, 1.81 g, 7.57 mmol, 1.0 equiv.) was suspended in EtOAc (125 mL) and freshly prepared isopropyl 2-oxoacetate (**S2-b**, 1.32 g, 11.4 mmol, 1.5 equiv.) was added. AcOH (0.43 mL, 7.57 mmol, 1.0 equiv.) was added dropwise under vigorous stirring and the mixture was heated to reflux for 48 h. The mixture was then cooled to room temperature and concentrated under reduced pressure. The obtained white residue was dissolved in warm CH_2Cl_2 (200 mL, warmed in a 30 °C water bath) and an equal volume of *i*-hexanes was added slowly under swirling of the flask. The solution was kept at room temperature for two hours whereupon the initiation of crystallization was observed. The mixture was then cooled to –20 °C overnight and the formed solids were filtered off, washed with *i*-hexanes and dried under vacuum to yield **S3-b** (2.10 g, 5.90 mmol, 78% over two steps) as white solid.

^{1}H-NMR: (501 MHz, CD_2Cl_2): δ = 7.79 (d, J = 7.6 Hz, 2H), 7.61 (d, J = 7.5 Hz, 2H), 7.42 (t, J = 7.6, 2H), 7.33 (t, J = 7.4, 2H), 5.95 (s, br, 1H), 5.33 (s, br, 1H), 5.09 (p, J = 6.3 Hz, 1H), 4.46–4.37 (m, 2H), 4.25 (t, J = 6.9 Hz, 1H), 3.68 (s, br, 1H), 1.29 (d, J = 6.3 Hz, 3H) 1.27 (d, J = 6.3 Hz, 3H).

^{13}C-NMR: (126 MHz, CD_2Cl_2): δ = 169.30, 144.17, 141.71, 128.17, 127.50, 125.41, 120.39, 74.12, 71.17, 67.59, 47.45, 21.75 (carbamate carbon not detected).

ESI-HRMS: calculated for $C_{20}H_{21}NNaO_5$ ($[M+Na]^+$): 378.13174, found: 378.13146.

ethyl 2-((((9H-fluoren-9-yl)methoxy)carbonyl)amino)-2-hydroxyacetate (S3-c)

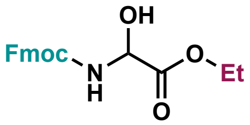

S3-c

Following GP A, in an oven-dried 250 mL two-neck flask equipped with a reflux condenser, (9H-fluoren- 9-yl)methyl carbamate (**S1-a**, 2.1 g, 8.78 mmol, 1.0 equiv.) was suspended in EtOAc (125 mL) and commercial ethyl 2-oxoacetate (**S2-c**, 50% solution in PhMe, 2.7 mL, 13.2 mmol, 1.5 equiv.) was added. AcOH (0.5 mL, 8.78 mmol, 1.0 equiv.) was added dropwise under vigorous stirring and the mixture was heated to reflux for 48 h. The mixture was cooled to room temperature and concentrated under reduced pressure. The obtained white residue was dissolved in warm CH_2Cl_2 (120 mL, warmed in a 30 °C water bath) and an equal volume of *i*-hexanes was added slowly under swirling of the flask. The solution was kept at room temperature for two hours whereupon initiation of the crystallization was observed. The mixture was then cooled to –20 °C overnight and the formed solids were filtered off, washed with *i*-hexanes and dried under vacuum to yield the desired hemiaminal **S3-c** (2.26 g, 6.62 mmol, 75%) as white solid.

**TLC:**	R_F (*i*-hexanes/EtOAc 2:1) = 0.26.
**^{1}H-NMR:**	(501 MHz, $CDCl_3$): δ = 7.77 (d, J = 7.6 Hz, 2H), 7.58 (d, J = 7.5 Hz, 2H), 7.41 (t, J = 7.5 Hz, 2H), 7.32 (t, J = 7.4 Hz, 2 H), 5.99 (s, br, 1H), 5.46 (s, br, 1H), 4.48–4.40 (m, 2H), 4.30 (q, J = 7.0 Hz, 2H), 4.24 (t, J = 7.0 Hz, 1H), 3.85 (s, br, 1H), 1.33 (t, J = 7.2 Hz, 3H).
**^{13}C-NMR:**	(126 MHz, MeCN-d_3): δ = 170.50, 156.49, 145.02, 145.00, 142.17, 128.77, 128.16, 126.15, 121.03, 74.44, 67.53, 62.79, 47.90, 14.38.
**ESI-HRMS:**	calculated for $C_{19}H_{19}NNaO_5$ ($[M+Na]^+$): 364.11609, found: 364.11558.

ethyl 2-(((benzyloxy)carbonyl)amino)-2-hydroxyacetate (S3-d)

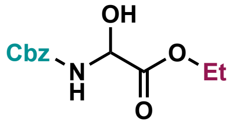

S3-d

Following GP A, benzyl carbamate (**S1-b**, 3.00 g, 20.0 mmol, 1.0 equiv.) was given to an oven-dried 50 mL pressure vial, suspended in anhydrous EtOAc (20 mL) and ethyl 2-oxoacetate (**S2-c**, 50% solution in PhMe, 4.86 mL, 23.8 mmol, 1.2 equiv.) was added. The mixture was stirred vigorously and AcOH (0.1 mL, 2.0 mmol, 0.10 equiv.) was added dropwise. The vial was sealed and heated to 75 °C for 48 h. The solution was then cooled to room temperature, concentrated under reduced pressure and the obtained white residue was dissolved in warm CH_2Cl_2 (200 mL, warmed in a 30 °C water bath). An equal volume of *i*-hexanes was added under swirling of the flask and the flask was left at room temperature for 2 h whereupon initiation of the crystallization was observed. The mixture was then cooled to –20 °C overnight and the formed solids were filtered off, washed with *i*-hexanes and dried under vacuum to yield the desired hemiaminal **S3-d** (2.26 g, 8.94 mmol, 45%) as white solid.

**TLC:**	R_F (*i*-hexanes/EtOAc 2:1) = 0.54.
**^{1}H-NMR:**	(501 MHz, $CDCl_3$): δ = 7.40 – 7.30 (m, 5H), 6.04 (s, br, 1H), 5.47 (d, J = 8.0 Hz, 1H), 5.14 (s, 2H), 4.27 (q, J = 7.2 Hz, 2H), 4.09 (s, br, 1H), 1.30 (t, J = 7.1 Hz, 3H).

**^{13}C-NMR:**	(126 MHz, $CDCl_3$): δ = 169.40, 155.74, 135.84, 128.72, 128.52, 128.35, 73.78, 67.56, 62.81, 14.13.
**ESI-HRMS:**	calculated for $C_{12}H_{15}NNaO_5$ ($[M+Na]^+$): 276.08479, found: 276.08424.

methyl 2-((((9H-fluoren-9-yl)methoxy)carbonyl)amino)-2-acetoxyacetate (2e)

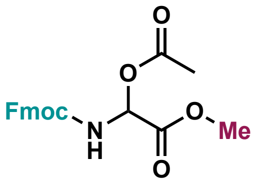

2e

Following GP B, methyl 2-((((9H-fluoren-9-yl)methoxy)carbonyl)amino)-2-hydroxyacetate (**S3-a**, 1.0 g, 3.06 mmol, 1.0 equiv.) was given to a flame dried 25 mL Schlenk tube followed by acetic anhydride (9 mL) and anhydrous pyridine (30 µL, 0.40 mmol, 0.13 equiv.). The heterogenous mixture was stirred at 25 °C for 20 h under an atmosphere of argon whereupon a clear homogenous solution was obtained. The mixture was then concentrated under reduced pressure at 70 °C and the residue was purified via flash column chromatography on silica gel (eluent: *i*-hexanes/EtOAc 4:1 ⟶ 3:1) to yield the desired *N,O*-acetal **2e** (761 mg, 2.06 mmol, 67%) as white solid.

**TLC:**	R_f (*i*-hexanes/EtOAc 3:1) = 0.27.
**^{1}H-NMR:**	(501 MHz, $CDCl_3$): δ = 7.77 (d, J = 7.6, 2H), 7.59 (d, 7.8 Hz, 2H), 7.41 (t, J = 7.5, 2H), 7.32 (td, J = 7.5, 1.2 Hz, 2H) 6.29 (d, J = 9.4 Hz, 1H), 6.20 (s, br, 1H), 4.49–4.41 (m, 2H), 4.24 (t, J = 7.0 Hz, 1H), 3.83 (s, 3H), 2.13 (s, 3H).
**^{13}C-NMR:**	(126 MHz, $CDCl_3$): δ = 170.37, 166.96, 155.00, 143.65, 143.58, 141.46, 127.98, 127.26, 125.16, 125.14, 120.21, 74.50, 67.90, 53.52, 47.07, 20.83.
**ESI-HRMS:**	calculated for $C_{20}H_{19}NNaO_6$ ($[M+Na]^+$): 392.11101, found: 392.11066.

ethyl 2-((((9H-fluoren-9-yl)methoxy)carbonyl)amino)-2-acetoxyacetate (2c)

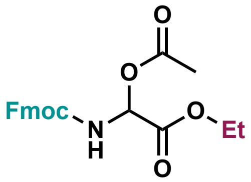

2c

Following GP B, ethyl 2-((((9H-fluoren-9-yl)methoxy)carbonyl)amino)-2-hydroxyacetate (**S3-c**, 860 mg, 2.52 mmol, 1.0 equiv.) was given to a flame dried 25 mL Schlenk tube followed by acetic anhydride (7.5 mL) and anhydrous pyridine (27 µL, 0.33 mmol, 0.13 equiv.). The heterogenous mixture was stirred at 25 °C for 20 h under an atmosphere of argon whereupon a clear homogenous solution was obtained. The mixture was then concentrated under reduced pressure at 70 °C and the residue was purified via flash column chromatography on silica gel (eluent: *i*-hexanes/EtOAc 4:1 ⟶ 3:1) to yield the desired acetate **2c** (819 mg, 2.14 mmol, 96%) as white solid.

**TLC:**	R_f (*i*-hexanes/EtOAc 3:1) = 0.46.
**^{1}H-NMR:**	(501 MHz, $CDCl_3$): δ = 7.77 (d, J = 7.6, 2H), 7.59 (d, J = 7.7 Hz, 2H), 7.41 (t, J = 7.5, 2H), 7.32 (td, J = 7.4, 1.1 Hz, 2H), 6.26 (d, J = 9.4 Hz, 1H), 6.22–6.17 (m,

1H), 4.49–4.42 (m, 2H), 4.29 (q, J = 7.2 Hz, 2H), 4.24 (t, J = 7.1 Hz, 1H), 2.13 (s, 3H), 1.31 (t, J = 7.1 Hz, 3H).

^{13}C-NMR: (126 MHz, $CDCl_3$): δ = 170.34, 166.44, 155.05, 143.66, 143.60, 141.44, 127.97, 127.25, 125.17, 125.15, 120.20, 74.67, 67.86, 62.87, 47.06, 20.82, 14.11.

ESI-HRMS: calculated for $C_{21}H_{21}NNaO_6$ ($[M+Na]^+$): 406.12666, found: 406.12630.

isopropyl 2-((((9H-fluoren-9-yl)methoxy)carbonyl)amino)-2-acetoxyacetate (2d)

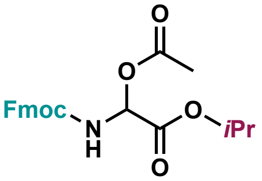

2d

Following GP B, isopropyl 2-((((9H-fluoren-9-yl)methoxy)carbonyl)amino)-2-hydroxyacetate (**S3-b**, 1.0 g, 2.81 mmol, 1.0 equiv.) was given to a flame dried 25 mL Schlenk tube followed by acetic anhydride (8 mL) and anhydrous pyridine (30 µL, 0.37 mmol, 0.13 equiv.). The heterogenous mixture was stirred at 25 °C for 20 h under an atmosphere of argon whereupon a clear homogenous solution was obtained. The mixture was then concentrated under reduced pressure at 70 °C and the residue was purified via flash column chromatography on silica gel (eluent: *i*-hexanes/EtOAc 4:1 ⟶ 3:1) to yield the desired acetate **2d** (660 mg, 1.66 mmol, 59%) as white solid.

TLC: R_f (*i*-hexanes/MTBE 2:1) = 0.48.

^{1}H-NMR: (501 MHz, $CDCl_3$): δ = 7.77 (d, J = 7.6, 2H), 7.59 (d, J = 7.5 Hz, 2H), 7.41 (t, J = 7.5, 2H), 7.32 (td, J = 7.5, 1.2 Hz, 2H), 6.22–6.16 (m, 2H), 5.11 (hept, J = 6.2 Hz, 1H), 4.50–4.39 (m, 2H), 4.25 (t, J = 7.1 Hz, 1H), 2.12 (s, 3H), 1.30 (d, J = 6.3 Hz, 3H), 1.28 (d, J = 6.2 Hz, 3H).

^{13}C-NMR: (126 MHz, $CDCl_3$): δ = 170.36, 165.93, 155.07, 143.68, 143.63, 141.45, 127.97, 127.25, 125.19, 125.17, 120.20, 74.89, 70.97, 67.85, 47.07, 21.72, 21.64, 20.81.

ESI-HRMS: calculated for $C_{22}H_{23}NNaO_6$ ($[M+Na]^+$): 420.14231, found: 420.14156.

ethyl 2-acetoxy-2-(((benzyloxy)carbonyl)amino)acetate (2a)

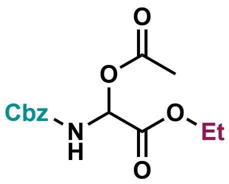

2a

Following GP B, ethyl 2-(((benzyloxy)carbonyl)amino)-2-hydroxyacetate (**S3-d**, 1.0 g, 3.95 mmol, 1.0 equiv.) was given to a flame dried 25 mL Schlenk tube followed by acetic anhydride (12 mL) and anhydrous pyridine (42 µL, 0.51 mmol, 0.13 equiv.). The homogeneous mixture was stirred at 25 °C for 20 h under an atmosphere of argon and subsequently concentrated under reduced pressure at 70 °C. The residue was purified via flash column chromatography on silica gel (eluent: *i*-hexanes/EtOAc 4:1 ⟶ 3:1) to yield the desired acetate **2a** (1.0 g, 3.39 mmol, 86%) as colorless oil.

TLC: R_f (*i*-hexanes/MTBE 2:1) = 0.28.

**^{1}H-NMR:**	(501 MHz, $CDCl_3$): δ = 7.41–7.29 (m, 5H), 6.25 (d, J = 9.4 Hz, 1H), 6.22 (s, br, 1H), 5.18–5.13 (m, 2H), 4.25 (q, J = 7.1 Hz, 2H), 2.10 (s, 3H), 1.28 (t, J = 7.2 Hz, 3H).
**^{13}C-NMR:**	(126 MHz, $CDCl_3$): δ = 170.31, 166.39, 155.01, 135.68, 128.71, 128.56, 128.42, 74.67, 67.81, 62.80, 20.77, 14.07.
**ESI-HRMS:**	calculated for $C_{14}H_{17}NNaO_6$ ($[M+Na]^+$): 318.09536, found: 318.09473.

ethyl 2-acetoxy-2-((*tert*-butoxycarbonyl)amino)acetate (2b)

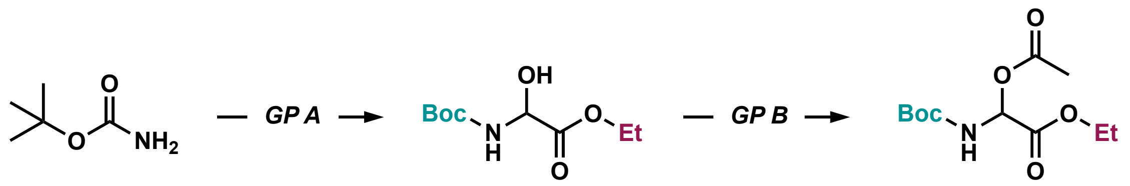

S1-c S3-e 2b

Following GP A, *tert*-butylcarbamate (**S1-c**, 351 mg, 3.0 mmol, 1.0 equiv.) was given to a 100 mL two-neck flask equipped with a reflux condenser. Anhydrous EtOAc (30 mL) was added followed by commercial ethyl glyoxylate (50% in PhMe, 0.80 mL, 3.90 mmol, 1.3 equiv.). The mixture was stirred vigorously, AcOH (20.0 µL, 0.30 mmol, 0.10 equiv.) was added dropwise and the mixture was then heated to reflux for 24 h. The mixture was cooled to room temperature and concentrated under reduced pressure. The crude material was directly used for the subsequent acetylation step.

Following GP B, crude ethyl 2-((tert-butoxycarbonyl)amino)-2-hydroxyacetate (**S3-e**) was given to a flame dried 10 mL Schlenk tube followed by acetic anhydride (3.5 mL) and anhydrous pyridine (12 µL, 0.15 mmol, 0.13 equiv.). The homogeneous mixture was stirred at 25 °C for 20 h under an atmosphere of argon and subsequently concentrated under reduced pressure at 70 °C. The residue was purified via flash column chromatography on silica gel (eluent: *i*-hexanes/EtOAc 7:1 ⟶ 3:1) to yield acetate **2b** (208 mg, 0.80 mmol, 27% over two steps) as colorless oil.

**TLC:**	R_f (*i*-hexanes/EtOAc 3:1) = 0.52
**^{1}H-NMR:**	(501 MHz, $CDCl_3$): δ = 6.18 (d, J = 9.4 Hz, 1H), 5.91 (s, br, 1H), 4.25 (q, J = 7.1 Hz, 2H), 2.11 (s, 3H), 1.46 (s, 9H), 1.29 (t, J = 7.1 Hz, 3H).
**^{13}C-NMR:**	(126 MHz, $CDCl_3$): δ = 170.45, 166.75, 154.12, 81.44, 74.65, 62.67, 28.30, 20.87, 14.12.
**ESI-HRMS:**	calculated for $C_{11}H_{19}NNaO_6$ ($[M+Na]^+$): 284.11101, found: 284.11055.

2.2 Synthesis of Arene Substrates

(2-methoxyphenyl)trimethylsilane (3r)

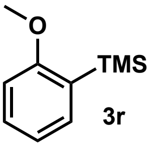

3r

Following a modified procedure reported by Schoenebeck,[7] 2-bromoanisole (**S5** 1.4 mL, 11.2 mmol, 1.0 equiv.) was given to a flame dried Schlenk tube under an atmosphere of argon, dissolved in anhydrous THF (23 mL) and cooled to –78 °C. A solution of *n*-BuLi (2.5 M in hexanes, 5.0 mL, 12.4 mmol, 1.1 equiv.) was added dropwise and the mixture was stirred for 1 h at –78 °C. TMSCl (1.71 mL, 13.5 mmol, 1.2 equiv.) was added dropwise, the mixture was slowly warmed to room temperature and stirred for 18 h at room temperature. The mixture was poured onto Et_2O (100 mL), washed with H_2O (3x 20 mL) and brine (20 mL), dried over anhydrous Na_2SO_4, filtered and concentrated under reduced pressure to yield anisole **3r** (2.0 g, 11.1 mmol, 98%) as colorless oil. The spectral data are in agreement with the reported literature.[7]

^{1}H-NMR: (501 MHz, $CDCl_3$): δ = 7.38 (dd, J = 7.1, 1.8 Hz, 1H), 7.35 (ddd, J = 8.1, 7.3, 1.8 Hz, 1H), 6.95 (td, J = 7.2, 0.9 Hz, 1H), 6.83 (d, J = 8.1 Hz, 1H), 3.81 (s, 3H), 0.27 (s, 9H).

(2-azidoethoxy)benzene (3s)

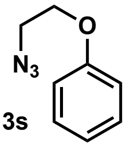

3s

Following a modified procedure reported by Elmali,[8] (2-bromoethoxy)benzene (**3t**, 2.0 g, 9.95 mmol, 1.0 equiv.) and NaN_3 (1.29 g, 19.9 mmol, 2.0 equiv.) were given to a 250 mL round bottom flask with an attached pressure compensation bubbler and dissolved in anhydrous DMF (100 mL). The mixture was heated to 100 °C for 16 h, then cooled to room temperature and poured onto water (100 mL). The mixture was extracted with CH_2Cl_2 (3x 25 mL) and concentrated under reduced pressure. The residue was filtered over a short plug of silica gel (eluent: *i*-hexanes) and concentrated under reduced pressure to yield anisole **3s** (1.50 g, 9.21 mmol, 93%) as colorless oil. The compound was stored in an aluminum foil coated vial at 4 °C. The spectral data are in agreement with the reported literature.[8]

^{1}H-NMR: (501 MHz, $CDCl_3$): δ = 7.35 – 7.27 (m, 2H), 6.99 (tt, J = 7.4, 1.1 Hz, 1H), 6.96–6.92 (m, 2H), 4.16 (t, J = 5.1 Hz, 2H), 3.60 (t, J = 5.0 Hz, 2H).

3. Synthesis of (*S,S*)-IDPi Catalysts

(*S,S*)-IDPi catalysts **4a**, **4b**, **4c**, **4d**, **4e** and **4g** were prepared according to reported literature procedures.[9–11] Phosphazene reagents **S9-a**[9] and **S9-b**[10] were prepared following reported literature procedures.

General Remark on the Synthesis of (*S,S*)-IDPi Catalysts: Our group recently reported an alternative synthesis for the preparation of IDPi catalysts based on the usage of hexachlorobisphosphazonium salts.[12] While this route has proven its versatility in the modular synthesis of broad IDPi libraries, we chose to follow the previously established phosphazene-route as a variety of phosphazene reagents are regularly prepared on large scales in our laboratory which consequentially facilitates the reaction setup.

3.1 Synthesis of substituted (S)-BINOLs

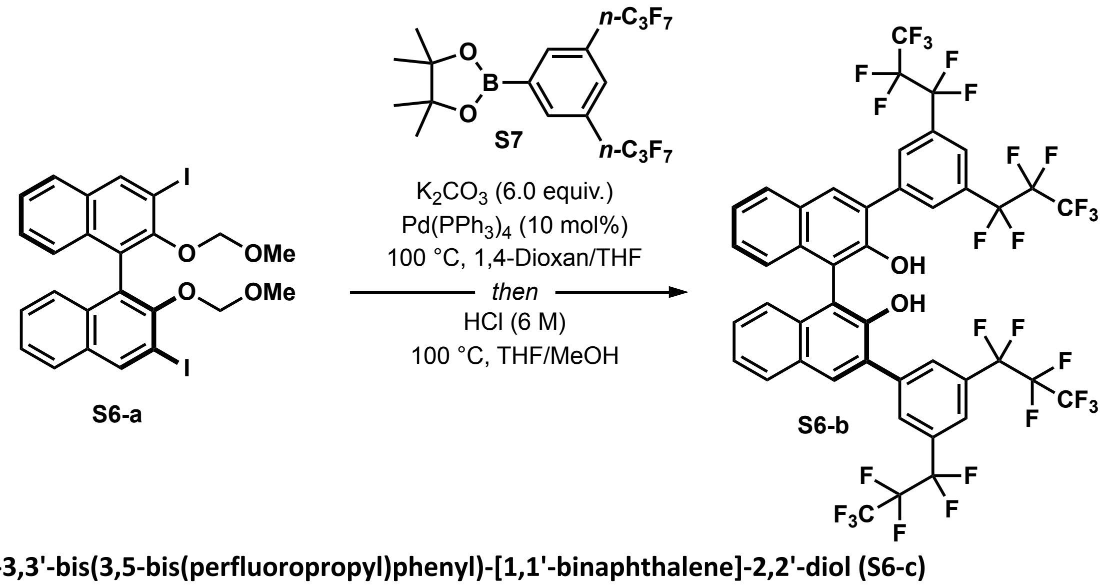

(S)-3,3'-bis(3,5-bis(perfluoropropyl)phenyl)-[1,1'-binaphthalene]-2,2'-diol (S6-c)

A Schlenk tube was charged with (*S*)-3,3'-diiodo-2,2'-bis(methoxymethoxy)-1,1'-binaphthalene (**S6-a**, 1.35 g, 2.16 mmol, 1.0 equiv.), 2-(3,5-bis(perfluoropropyl)phenyl)-4,4,5,5-tetramethyl-1,3,2-dioxaborolane (**S7**, 3.5 g, 6.48 mmol, 3.0 equiv.) and K_2CO_3 (1.79 g, 13.0 mmol, 6.0 equiv.). A 4:1 (*v:v*) mixture of H_2O and 1,4-dioxane (28 mL) was added and the mixture was degassed in a constant stream of Ar bubbling through the mixture under stirring for 20 min. $Pd(PPh_3)_4$ (250 mg, 0.22 mmol, 10 mol%) was added, the tube was sealed and heated to 110 °C for 16 h. The mixture was then cooled to room temperature, diluted with CH_2Cl_2 (100 mL) and water (100 mL) was added. The layers were separated, the aqueous layer was extracted with CH_2Cl_2 (3 x 70 mL) and the combined organic extracts were concentrated under reduced pressure.

The obtained residue was dissolved in a 6:1 (*v:v*) mixture of THF and MeOH (42 mL) and then treated with HCl (6.0 M, 13 mL). The mixture was transferred to two separate microwave reaction vials and heated to 100 °C for 1 h in a microwave reactor, respectively. The vials were cooled to 25 °C, combined and diluted with H_2O (100 mL) and CH_2Cl_2 (100 mL) and the layers were separated. The aq. layers were

extracted with CH_2Cl_2 (3 x 70 mL), the combined organic extracts were washed with brine (100 mL) and dried over anhydrous Na_2SO_4. The solids were filtered off, the filtrate was adsorbed on celite® and the solvent was removed under reduced pressure. The crude mixture was purified via flash column chromatography on silica gel (eluent: *i*-hexanes/CH_2Cl_2 98:2 ⟶ 95:5) to yield (*S*)-BINOL **S6-b** (1.99 g, 1.79 mmol, 83%) as white solid. The analytical data are in agreement with the reported literature.[9]

TLC: R_f (*i*-hexanes/CH_2Cl_2 10:1) = 0.32.

^{1}H-NMR: (501 MHz, $CDCl_3$): δ = 8.25 (s, 4H), 8.13 (s, 2H), 8.02 (d, J = 8.1 Hz, 2H), 7.84 (s, 2H), 7.50 (ddd, J = 8.0, 6.8, 1.3 Hz, 2H), 7.44 (ddd, J = 8.3, 6.9, 1.4 Hz, 2H), 7.25 (d, J = 8.0 Hz, 2H), 5.40 (s, 2H).

^{13}C-NMR: (126 MHz, $CDCl_3$): δ = 150.08, 139.47, 133.52, 132.63, 131.79, 131.75, 131.69, 130.18, 129.99, 129.79, 129.66, 129.10, 128.89, 127.76, 125.40, 124.39, 124.19, 121.51, 119.50, 119.23, 118.96, 117.21, 116.95, 116.70, 115.17, 114.92, 114.67, 113.13, 112.89, 112.64, 111.97, 111.36, 111.06, 110.75, 110.45, 109.26, 108.96, 108.65, 108.35, 107.16, 106.86, 106.55, 106.25, 77.41, 77.37, 77.16, 76.91.

^{19}F-NMR: (471 MHz, $CDCl_3$) δ = –79.94 (t, J = 9.8 Hz), –111.90 (q, J = 10.0 Hz), –126.16.

ES-HRMS: calculated for $C_{44}H_{17}F_{28}O_2$ [$(M-H)^-$]: 1109.07869, found: 1109.07881.

(S)-3,3'-bis(3,5-bis(pentafluoro-λ⁶-sulfaneyl)phenyl)-[1,1'-binaphthalene]-2,2'-diol (S6-d)

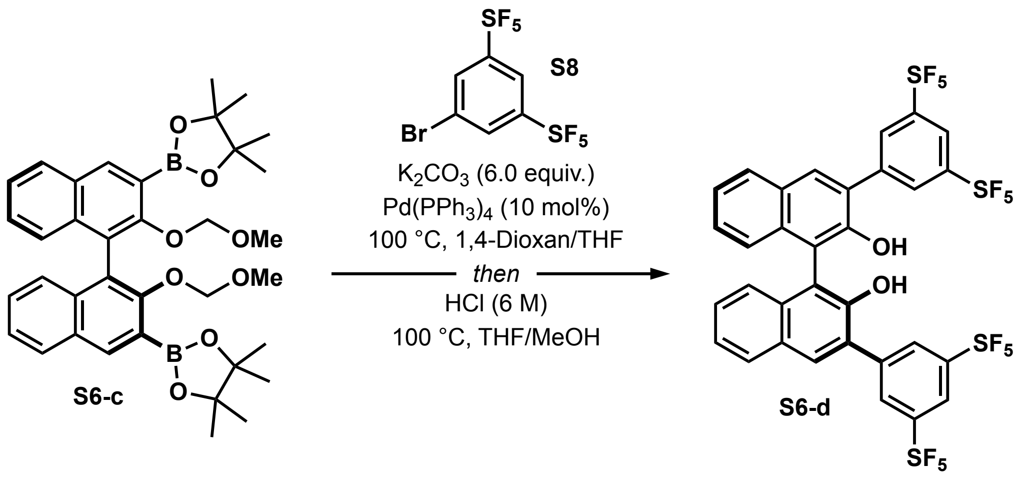

A Schlenk tube was charged with (*S*)-2,2'-(2,2'-bis(methoxymethoxy)-[1,1'-binaphthalene]-3,3'-diyl)bis(4,4,5,5-tetramethyl-1,3,2-dioxaborolane) (**S6-c**, 250 mg, 0.40 mmol, 1.0 equiv.), (5-bromo-1,3-phenylene)bis(pentafluoro-λ^6-sulfane) (**S8**, 375 mg, 0.92 mmol, 2.3 equiv.) and K_2CO_3 (331 mg, 2.4 mmol, 6.0 equiv.). A 6:1 (*v*:*v*) mixture of H_2O and 1,4-dioxane (6 mL) was added and the mixture was degassed in a constant stream of Ar bubbling through the mixture under stirring for 20 min. $Pd(PPh_3)_4$ (46 mg, 0.04 mmol, 10 mol%) was added, the tube was sealed and heated to 110 °C for 16 h. The mixture was then cooled to room temperature, diluted with CH_2Cl_2 (10 mL) and water (10 mL) was added. The layers were separated, the aqueous layer was extracted with CH_2Cl_2 (3 x 10 mL) and the combined organic extracts were concentrated under reduced pressure.

The obtained residue was dissolved in a 6:1 (*v:v*) mixture of THF and MeOH (8.5 mL) and then treated with HCl (6.0 M, 1.2 mL). The mixture was transferred to a microwave reaction vial and heated to 100 °C for 1 h in a microwave reactor. The vial was cooled to room temperature, diluted with H_2O (10 mL) and CH_2Cl_2 (10 mL) and the layers were separated. The aqueous layer was extracted with CH_2Cl_2 (3 x 10 mL), the combined organic extracts were washed with brine (10 mL) and dried over anhydrous Na_2SO_4. The solids were filtered off, the filtrate was adsorbed on celite® and the solvent was removed under reduced pressure. The crude mixture was purified via flash column chromatography on silica gel (eluent: *i*-hexanes/CH_2Cl_2 3:1 ⟶ 2:1) to yield (*S*)-BINOL **S6-d** (239 mg, 0.25 mmol, 64%) as white solid. The analytical data are in agreement with the reported literature.[9]

**TLC:**	R_f (*i*-hexanes/EtOAc 20:1) = 0.58.
**^{1}H-NMR:**	(501 MHz, $CDCl_3$): δ = 8.33 (d, J = 2.1 Hz, 4H), 8.16 (t, J = 2.1 Hz, 2H), 8.08 (s, 2H), 8.00 (d, J = 8.0 Hz, 2H), 7.47 (ddd, J = 8.6, 7.6, 0.97 Hz, 2H), 7.41 (ddd, J = 8.9, 8.0, 1.0 Hz, 2H), 7.21 (d, J = 8.3 Hz, 2H), 5.84 (s, 2H).
**^{13}C-NMR:**	(126 MHz, $CDCl_3$): δ = 153.55 (p, J = 19.0 Hz), 149.95, 139.77, 133.71, 132.42, 130.41, 129.44, 129.03, 128.95, 127.31, 125.40, 124.18, 123.03, 112.37.
**^{19}F-NMR**	(471 Mhz, $CDCl_3$): δ = 81.98 (p, J = 150.6 Hz), 63.08 (d, J = 150.6 Hz).
**ES-HRMS:**	calculated for $C_{32}H_{17}F_{20}O_2S_4$ [$(M-H)^-$]: 940.97975, found: 940.97990.

3.2 Synthesis of (S,S)-IDPi Catalysts from 3,3'-disubstituted BINOLs

(*S,S*)-IDPi catalysts were prepared following a modified procedure reported by our group:

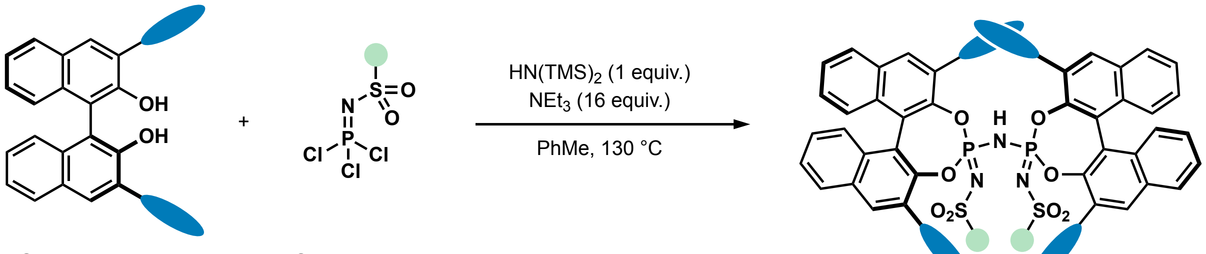

<u>**General Procedure C (GP C):**</u> **Synthesis of (*S,S*)-IDPi Catalysts**

Following a modified procedure reported by List, the respective 3,3'-disubstituted (*S*)-BINOL **S6** (2.1 equiv.) was given to a flame dried Schlenk tube under an atmosphere of argon and dissolved in anhydrous PhMe.[10] Phosphazene reagent **S9** (2.1 equiv.) was added followed by NEt_3 (16 equiv.) and the mixture was stirred at room temperature for 30 min. Hexamethyldisilazane (1.0 equiv.) was added, the mixture was stirred for another 20 min at room temperature and subsequently heated to 130 °C for 3 days. The tube was then cooled to room temperature, diluted with CH_2Cl_2 and quenched via the addition of HCl (10%). The layers were separated and the aqueous layer was extracted with CH_2Cl_2, the combined organic extracts dried over anhydrous Na_2SO_4 and concentrated under reduced pressure. The crude residue was purified via flash column chromatography on silica gel to yield the salts of the

(*S,S*)-IDPis **4** which were acidified via filtration over a short plug of DOWEX 50WX8 (H-form, eluted with CH_2Cl_2) to yield (*S,S*)-IDPis **4** as white or off-white solids.

(*S,S*)-(3,5-bis-perfluoropropylphenyl)-C_2F_5 IDPi (4f)

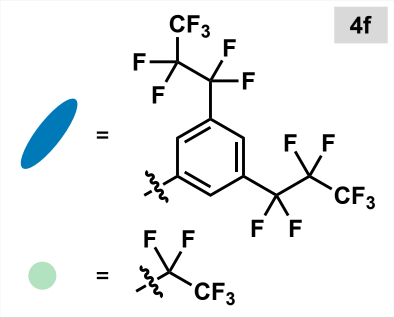

Prepared according to GP C: (*S*)-3,3'-bis(3,5-bis(perfluoropropyl)-phenyl)-[1,1'-binaphthalene]-2,2'-diol (**S6-b**, 671 mg, 0.60 mmol, 2.1 equiv.) was given to a flame dried Schlenk tube under an atmosphere of argon and dissolved in anhydrous PhMe (6.5 mL). ((perfluoroethyl)sulfonyl)phosphorimidoyl trichloride (**S9-a,** 202 mg, 0.60 mmol, 2.1 equiv.) was added followed by triethylamine (0.64 mL, 4.60 mmol, 16 equiv.) and the mixture was stirred at room temperature for 30 min. Hexamethyldisilazane (60 µL, 0.29 mmol, 1.0 equiv.) was added, the mixture was stirred for another 20 min at room temperature and subsequently heated to 130 °C for 3 days. The tube was then cooled to room temperature, diluted with CH_2Cl_2 (30 mL) and quenched via the addition of HCl (10%, 30 mL). The layers were separated, and the aqueous layer was extracted with CH_2Cl_2 (3 x 30 mL), the combined organic extracts were dried over anhydrous Na_2SO_4 and concentrated under reduced pressure. The crude residue was purified via flash column chromatography on silica gel (eluent: *i*-hexanes/CH_2Cl_2 1:1 ⟶ CH_2Cl_2/EtOAc 99:1) and the isolated salt was acidified via filtration over a short plug of DOWEX 50WX8 (H-form, eluted with CH_2Cl_2) to yield (*S,S*)-IDPi **4f** (570 mg, 0.21 mmol, 74%) as off-white solid.

**TLC:**	R_F (i-hexanes/EtOAc 3:1) = 0.72.
**^{1}H-NMR:**	(501 MHz, $CDCl_3$): δ = 8.10 (s, 2H), 8.09 (d, J = 8. Hz, 2H), 7.90 (d, J = 3.9 Hz, 4H), 7.74 (s, 4H) 7.72 (d, J = 8.9 Hz, 2H), 7.69–7.65 (m, 6H), 7.62 (t, J = 7.5 Hz 2H), 7.38 (ddd, J = 8.4, 6.8, 1.2 Hz, 2H), 7.32 (s, 4H), 7.10 (d, J = 8.6 Hz, 2H), 6.57 (s, 2H), 5.99 (s, br, 1H).
**^{13}C{^{1}H, ^{19}F}-NMR:**	(126 MHz, $CDCl_3$): δ = 143.86 (t, J = 5.4 Hz), 141.75 (t, J = 5.1 Hz), 138.14, 137.96, 133.94, 132.22, 131.97 (d, J = 8.3 Hz), 131.96, 131.50, 131.13, 130.86, 130.73, 130.18, 130.14, 129.86, 129.24, 128.64, 128.00, 127.23, 127.08, 126.74, 125.35 (d, J = 12.5 Hz), 123.67, 121.47, 118.02, 117.79, 117.05, 114.42 (d, J = 10.7 Hz), 111.29, 108.70, 108.39.
**^{19}F-NMR:**	(471 MHz, $CDCl_3$): δ = –79.00 (s), –79.98 (t, J = 9.5 Hz), –80.14 (t, J = 9.6 Hz), –112.06 (s), –112.65 (s), –113.92 (d, J = 277.8 Hz), –116.50 (s), –126-04 (s), –126.55 (d, J = 291.3 Hz).
**^{31}P-NMR:**	(202 MHz, $CDCl_3$) δ = –14.27.
**ESI-HRMS:**	calculated for $C_{92}H_{32}F_{66}N_3O_8P_2S_2$ ($[M-H]^-$): 2686.00522, found: 2686.00395.
**$[\propto]_D^{25}$:**	184.3 (c = 0.13, $CHCl_3$).

(*S,S*)-(3,5-bis-(pentafluoro-λ^6-sulfaneyl)phenyl)-C_6F_{13} IDPi (4h)

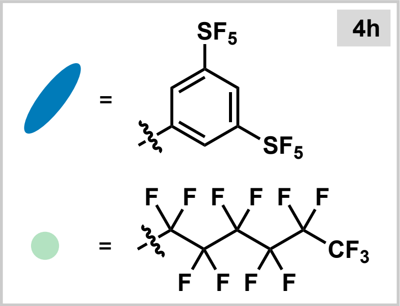

Prepared according to GP C: (*S*)-3,3'-bis(3,5-bis(pentafluoro-λ^6-sulfaneyl)phenyl)-[1,1'-binaphthalene]-2,2'-diol (**S6-d**, 64 mg, 68 µmol, 2.1 equiv.) was given to a flame dried Schlenk tube under an atmosphere of argon and dissolved in anhydrous PhMe (1.2 mL). ((perfluorohexyl)sulfonyl)phosphorimidoyl trichloride (**S9-b,** 36 mg, 68 µmol, 2.1 equiv.) was added followed by triethylamine (72 µL, 0.52 mmol, 16 equiv.) and the mixture was stirred at room temperature for 30 min. Hexamethyldisilazane (6.7 µL, 32 µmol, 1.0 equiv.) was added, the mixture was stirred for another 20 min at room temperature and subsequently heated to 130 °C for 3 days. The tube was then cooled to room temperature, diluted with CH_2Cl_2 (10 mL) and quenched via the addition of HCl (10%, 10 mL). The layers were separated, and the aqueous layer was extracted with CH_2Cl_2 (3 x 10 mL), the combined organic extracts dried over anhydrous Na_2SO_4 and concentrated under reduced pressure. The crude residue was purified via flash column chromatography on silica gel (eluent: CH_2Cl_2/EtOAc 99.75:0.25 ⟶ 99:1) and the isolated salt was acidified via filtration over a short plug of DOWEX 50WX8 (H-form, eluted with CH_2Cl_2) to yield (*S,S*)-IDPi **4h** (57 mg, 21 µmol, 64%) as off-white solid.

**TLC:**	R_F (CH_2Cl_2/EtOAc 99:1) = 0.63.
**^{1}H-NMR:**	(501 MHz, $CDCl_3$): δ = 8.18 (s, 2H), 8.14 (d, H = 1.6 Hz, 2H), 8.13 (d, J = 4.5 Hz, 2H), 8.09 (t, J = 1.9 Hz, 2H), 7.96–7.87 (m, 4H), 7.82 (s, 4H), 7.81–7.76 (m, 2H), 7.73 (ddd, J = 8.3, 6.2, 1.7 Hz, 2H), 7.65 (t, J = 7.5 Hz, 2H), 7.47–7.37 (m, 6H), 7.07 (d, J = 8.6 Hz, 2H), 6.55 (s, 2H).
**^{13}C{^{1}H, ^{19}F}-NMR:**	(126 MHz, $CDCl_3$): δ = 153.71 (q, J = 19.1 Hz), 143.50 (t, J = 5.4 Hz), 141.29 (t, J = 5.1 Hz), 138.25, 137.29, 133.89, 132.58, 132.29, 132.08, 131.00, 130.36, 130.18, 129.85, 129.52, 129.14, 128.49, 127.53, 127.47, 127.18, 126.69, 124.31, 124.01, 123.93, 121.60, 117.23, 113.15, 110.60, 110.44, 110.17, 108.38, 100.12.
**^{19}F-NMR:**	(471 MHz, $CDCl_3$): δ = 80.97 (p, J = 149.6 Hz), 63.29 (d, J = 149.7 Hz), 62.35 (d, J = 150.0 Hz), -81.01 (t, J = 9.9 Hz), -111.97 (d, J = 258.8 Hz), -113.35 (d, J = 258.7 Hz), -119.93 (dt, J = 34.3, 15.8 Hz), -122.03 (d, J = 82.4 Hz), -123.08 (d, J = 86.3 Hz), -126.36 (dt, J = 28.9, 14.6 Hz).
**^{31}P-NMR:**	(203 MHz, $CDCl_3$) δ = –14.34.
**ESI-HRMS:**	calculated for $C_{76}H_{32}F_{66}N_3O_8P_2S_{10}$ ($[M-H]^-$): 2749.78178, found: 2749.78287.
$[\propto]_D^{25}$:	267.2 (c = 0.13, $CHCl_3$).

4. Reaction Development

4.1 Hydrocarbon Arene Substrates

Reaction conditions for the conversion of hydrocarbon arenes were optimized as described below using toluene (**3a**) as representative substrate: An oven dried 1 mL screwcap vial equipped with a magnetic stirring bar was charged with the respective catalyst (5 mol%) and *N*,*O*-acetal **2** (1.0 equiv., 0.025 mmol). The vial was evacuated and placed under an atmosphere of argon. Anhydrous PhMe (**3a**, 0.25 mL) was added via syringe, the vial was sealed and the mixture was stirred at 30 °C for 16 h if not indicated otherwise. The reaction was then quenched via addition of triethylamine (0.3 M solution in PhMe, 50 µL), dimethylsulfone (2.0 M solution in MeCN, 12.5 µL, 1.0 equiv.) was added as internal standard and the mixture was diluted with CH_2Cl_2 (0.2 mL). An aliquot of the reaction was diluted with $CDCl_3$ (0.6 mL) and analyzed via ^{1}H-NMR for the determination of the reaction's yield. The remaining solution was purified via preparative thin layer chromatography (PTLC) and analyzed via HPLC for the determination of the regioisomeric ratio as well as the enantiomeric ratio.

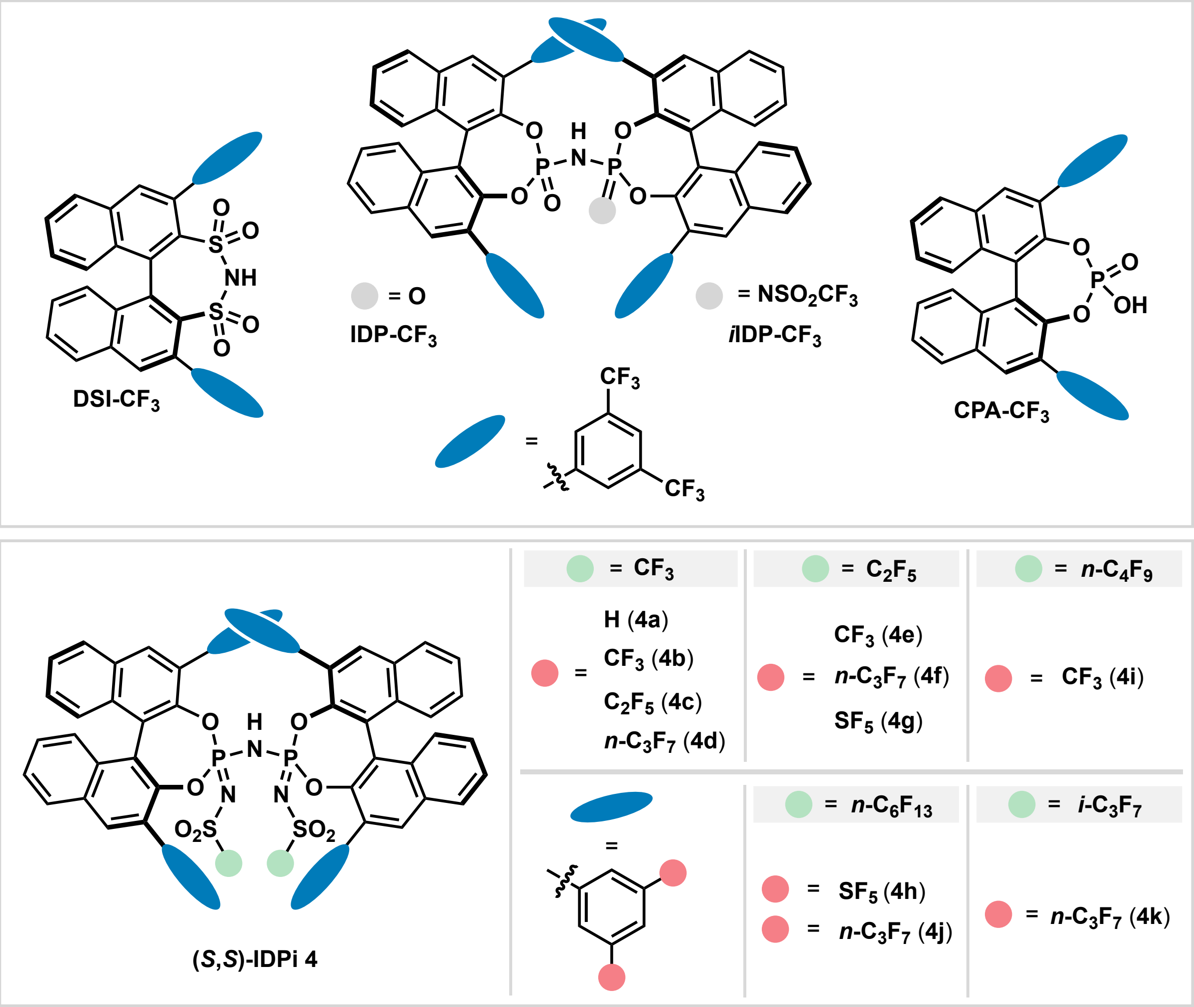

Figure SI-1: Catalysts investigated herein for the reaction development.

Table S-1: Reaction optimization for toluene (**3a**) as representative substrate for hydrocarbon arenes.

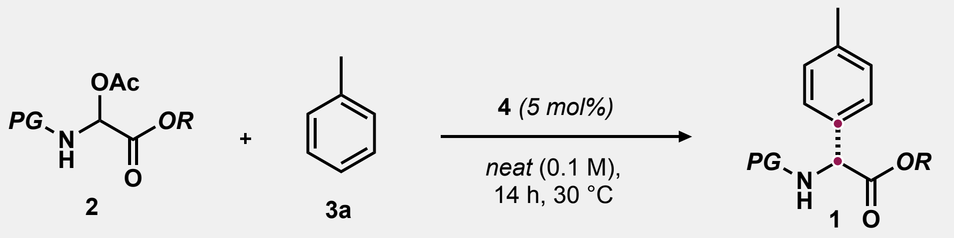

entry	catalyst	*PG*	*R*	yield (%)[d]	e.r.[e]	r.r.[e]
1[a]	CPA-CF_3	Cbz	Et (**2a**)	<5	n.d.	n.d.
2[a]	DSI-CF_3	Cbz	Et (**2a**)	<5	n.d.	n.d.
3[a]	IDP-CF_3	Cbz	Et (**2a**)	<5	n.d.	n.d.
4[a]	*i*IDP-CF_3	Cbz	Et (**2a**)	<5	n.d.	n.d.
5[a]	4a	Cbz	Et (**2a**)	<5	n.d.	n.d.
6	4b	Cbz	Et (**2a**)	50	82:18	20:1
7	4b	Boc	Et (**2b**)	<5	n.d.	n.d.
8	4b	Fmoc	Et (**2c**)	42	**90:10**	>20:1
9[b]	4b	Fmoc	Et (**2c**)	19	95.5:4.5	>20:1
10	4b	Fmoc	Me (**2e**)	38	90:10	>20:1
11	4b	Fmoc	*i*Pr (**2d**)	44	91:9	>20:1
12	4c	Fmoc	Et (**2c**)	12	93.5:6.5	>20:1
13	4d	Fmoc	Et (**2c**)	29	94:6	>20:1
14	4e	Fmoc	Et (**2c**)	55	92.5:7.5	>20:1
15	4i	Fmoc	Et (**2c**)	23	91.5:8.5	>20:1
16	4f	Fmoc	Et (**2c**)	15	95.5:4.5	>20:1
17	4g	Fmoc	Et (**2c**)	**70**	93:7	>20:1
18[c]	4g	Fmoc	Et (**2c**)	**55**	**96:4**	>20:1
19	4h	Fmoc	Et (**2c**)	46	93:7	>20:1
20	4j	Fmoc	Et (**2c**)	30	96:4	>20:1
21	4k	Fmoc	Et (**2c**)	45	94.5.5.5	>20:1

a = no relevant product formation even at 90 °C over 3 d; ***b*** = at 0 °C, 3 d reaction time; ***c*** = at 15 °C, 5 d reaction time; ***d*** = determined via ^{1}H-NMR using dimethylsulfone as internal standard; ***e*** = determined via HPLC analysis

General Remark for the Reaction of Purely Hydrocarbon Arenes: The transformations of only hydrocarbon arenes were exclusively performed neatly in the respective hydrocarbon arene **1** as we observed significantly reduced reactivities when solvent mixtures of arenes **1** and non-aromatic cosolvents were used.

4.2 Anisoles and Heteroaromatic Substrates

Building up on the experiments performed for the Friedel–Crafts reaction of toluene, the conditions for the conversion of anisole-derived arenes and heterocyclic arenes were optimized as described below using anisole as representative substrate: An oven dried 1 mL screwcap vial equipped with a magnetic stirring bar was charged with the catalyst (5 mol%) and *N*,*O*-acetal **2c** (1.0 equiv., 0.025 mmol). The vial was evacuated under high vacuum and subsequently placed under an atmosphere of argon. Solvent (0.25 mL) was added via syringe quickly followed by anisole (2.0 equiv., 0.05 mmol), the vial was sealed and the mixture was stirred at 30 °C for 16 h. The reaction was then

quenched via addition of triethylamine (0.3 M solution in PhMe, 50 µL), dimethylsulfone (2.0 M solution in MeCN, 12.5 µL, 1.0 equiv.) was added as internal standard and the mixture was diluted with CH_2Cl_2 (0.2 mL). An aliquot of the reaction was diluted with $CDCl_3$ (0.6 mL) and analyzed via ^{1}H-NMR to determine the reaction yield. The remaining mixture was purified via preparative thin layer chromatography (PTLC) for the subsequent HPLC analysis for the determination of the regioisomeric ratio as well as the enantiomeric ratio.

Table S-2: Reaction optimization for anisole-derived arenes and heterocyclic arenes using anisole (**3k**) as representative substrate.

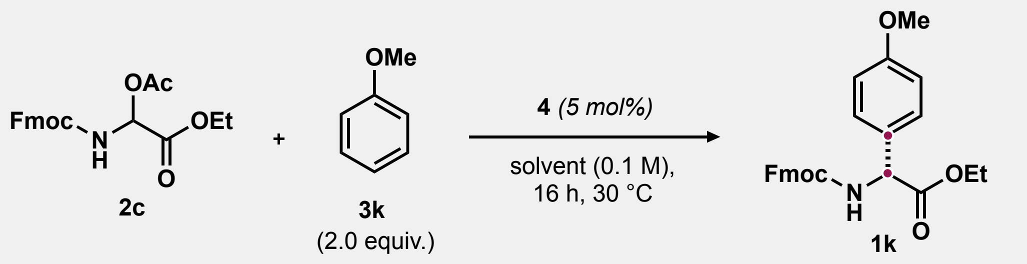

entry	catalyst	solvent	yield (%)[b]	e.r.[c]	r.r.[c]
1	4b	CH_2Cl_2	68	58:42	>20:1
2	4b	PhMe	83	84:16	>20:1
3[a]	4b	CyH	46	91:9	>20:1
4[a]	4b	CyMe	68	87:13	>20:1
5[a]	4d	CyMe	50	82:18	>20:1
6[a]	4f	CyMe	**90**	**96:4**	>20:1
7[a]	4f	*n*-pentane	81	**98:2**	>20:1
8[a]	4f	*n*-hexane	94	96:4	>20:1

a = heterogeneous reaction conditions; ***b*** = determined via ^{1}H-NMR using dimethylsulfone as internal standard; ***c*** = determined via HPLC analysis

General Remark for the Reaction of Alkoxybenzenes and Heteroarenes: IDPis **4** and *N,O*-acetal **2c** are poorly soluble in *n*-pentane, *n*-hexane or CyMe which generally results in heterogeneous reaction conditions for the transformation of alkoxybenzenes and heteroarenes. We could however observe that in *n*-hexane and especially CyMe, the solubility of *N,O*-acetal **2c** is improved compared to *n*-pentane (while usage of *n*-pentane leads to visible aggregation of **2c**, evenly dispersed heterogeneous mixtures are obtained using CyMe). We speculate that differences in solubility correlate with increased or decreased reactivity. For substrates **3q** and **3v-x**, CyMe or *n*-hexane was used to avoid the more significantly reduced reactivity observed in *n*-pentane. We however chose to use *n*-pentane as solvent wherever possible for maximum enantioselectivity.

5. Asymmetric Friedel–Crafts Reactions toward Arylglycines

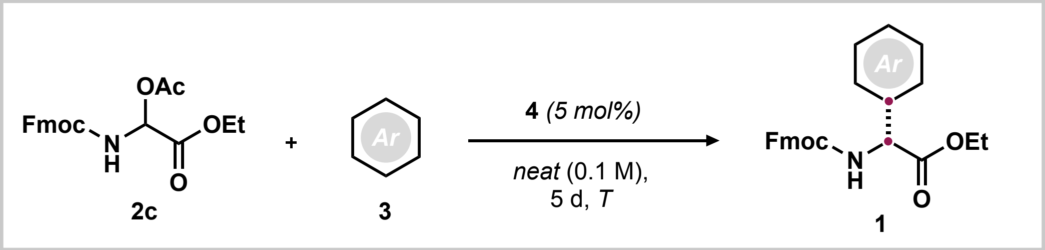

General Procedure D (GP D): Friedel–Crafts Reaction of Only Hydrocarbon Arenes

A flame-dried 5 mL finger Schlenk tube under an atmosphere of argon equipped with a magnetic stirring bar was charged with *N,O*-acetal **2c** (38.3 mg, 0.1 mmol, 1.0 equiv.) and (*S,S*)-IDPi catalyst **4** (5 µmol, 5 mol%). The respective hydrocarbon arene (1.0 mL) was then added via syringe. If the reaction was performed at temperatures below 30 °C, the tube was cooled on dry ice during the addition of the respective arene substrate. The tube was sealed and the reaction was stirred at the indicated temperature for the indicated reaction time. The mixture was then quenched with triethylamine (0.3 M solution in PhMe, 200 µL) and applied directly on a silica gel column equilibrated with *i*-hexanes. The mixture was flushed with *i*-hexanes (100 mL) and then purified via flash column chromatography on silica gel (*i*-hexanes/MTBE eluents) to yield arylglycines **1** as white solids. Regioisomeric ratios were determined either via ^{1}H-NMR- or HPLC analysis. *Deviations from the general protocol are indicated at the respective entry.*

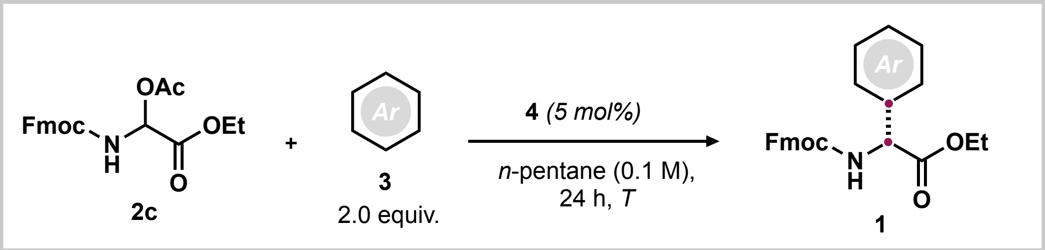

General Procedure E (GP E): Friedel–Crafts Reaction of Anisoles and Heterocyclic Arenes

A flame-dried 5 mL finger Schlenk tube under an atmosphere of argon and equipped with a magnetic stir bar was charged with *N,O*-acetal **2c** (38.3 mg, 0.1 mmol, 1.0 equiv.) and (*S,S*)-IDPi catalyst **4** (5 µmol, 5 mol%). *n*-pentane (1.0 mL) or CyMe (1.0 mL) was added via syringe followed by the respective arene substrate **3** (0.2 mmol, 2.0 equiv.). If the reaction was performed at temperatures below 30 °C, the tube was cooled on dry ice during the addition of arene **3**. The tube was sealed and the reaction was stirred at the respective temperature for the indicated reaction time. The mixture was then quenched with NEt_3 (0.3 M solution in PhMe, 200 µL) and applied directly on a silica gel column equilibrated with *i*-hexanes. The mixture was flushed with *i*-hexanes (100 mL) and then purified via flash column chromatography on silica gel (*i*-hexanes/MTBE eluents) to yield arylglycines **1** as white solids. Regioisomeric ratios were determined either via ^{1}H-NMR- or HPLC analysis. *Deviations from the general protocol are indicated at the respective entry.*

Preparation of Racemic Reference Samples

Racemic reference samples for the determination of the enantiomeric ratios of arylglycine derivates **1** were prepared using bistriflimide ($HNTf_2$, added as stock solution in CH_2Cl_2: 0.2 M, 50 µL, 10 µmol, 10 mol%) instead of (*S,S*)-IDPi catalysts under the reaction conditions described in General Procedures D and E. *Deviations from the general protocol are indicated at the respective entry.*

5.1 Friedel–Crafts Reaction of Only-Hydrocarbon Arenes

ethyl (*R*)-2-((((9H-fluoren-9-yl)methoxy)carbonyl)amino)-2-(p-tolyl)acetate (1a)

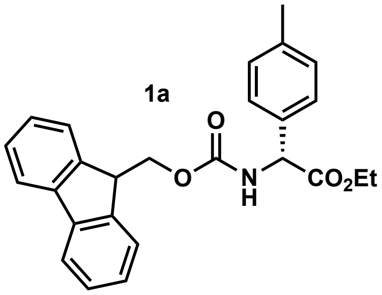

Prepared following *General Procedure D*, from *N,O*-acetal **2c** (38.4 mg, 0.1 mmol, 1.0 equiv.) and toluene (**3a**, 1.0 mL) using catalyst **4g** (11.7 mg, 5 µmol, 5 mol%) over 5 d reaction time at 15 °C. Purification by silica gel flash column chromatography (eluent: *i*-hexanes/MTBE 4:1) gave **3a** as white solid (23 mg, 55 µmol, 55%).

TLC: R_f (*i*-hexanes/MTBE 2:1) = 0.71.

^{1}H-NMR: (501 MHz, $CDCl_3$): mixture of two rotamers with a ratio of 1:0.20. δ = 7.76 (d, J = 7.6 Hz, 2H), 7.58 (d, J = 7.5 Hz, 2H), 7.40 (t, J = 7.6 Hz, 2H), 7.34–7.23 (m, 4H), 7.18 (d, J = 6.9 Hz, 2H), 5.82 (d, J = 6.8 Hz, $1H_{maj}$), 5.70 (s, $1H_{min}$), 5.33 (d, J = 6.9 Hz, $1H_{maj}$), 5.12 (s, $1H_{min}$), 4.44–4.32 (m, 2H), 4.30–4.10 (m, 3H), 2.35 (s, 3H), 1.22 (t, J = 6.7 Hz, 3H).

^{13}C-NMR: (126 MHz, CDCl3): δ = 171.15, 155.51, 144.03, 143.93, 141.43, 138.55, 133.91, 129.78, 127.83, 127.20, 125.24, 120.12, 67.26, 62.06, 57.88, 47.30, 21.31, 14.16.

ESI-HRMS: calculated for $C_{26}H_{25}NNaO_4$ ($[M+Na]^+$): 438.16813, found: 438.16767.

HPLC: (Chiralpak IG-3, *n*-heptane/*i*-PrOH 95:5, 298 K, 254 nm): t_R (major) = 32.0 min, t_R (minor) = 36.1 min, e.r. = 96:4 (92% e.e.).

$[\propto]_D^{25}$**:** –76.9 (c = 0.14, $CHCl_3$).

r.r.: >20:1 (favoring the *para* isomer), determined via HPLC analysis.

ethyl (*R*)-2-((((9H-fluoren-9-yl)methoxy)carbonyl)amino)-2-(4-ethylphenyl)acetate (1b)

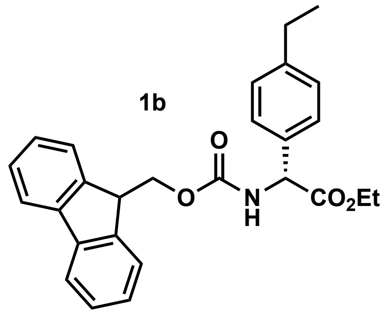

1b

Prepared following *General Procedure D*, from *N*,*O*-acetal **2c** (38.4 mg, 0.1 mmol, 1.0 equiv.) and ethylbenzene (**3b**, 1.0 mL) using catalyst **4g** (11.7 mg, 5 µmol, 5 mol%) over 5 d reaction time at 30 °C. Purification by silica gel flash column chromatography (eluent: *i*-hexanes/MTBE 4:1) gave **1b** as white solid (30 mg, 70 µmol, 70%).

TLC: R_f (*i*-hexanes/MTBE 2:1) = 0.62.

^{1}H-NMR: (501 MHz, $CDCl_3$): mixture of two rotamers with a ratio of 1:0.18. δ = 7.77 (d, J = 7.6 Hz, 2H), 7.60 (d, J = 7.5 Hz, 2H), 7.40 (t, J = 7.5 Hz, 2H), 7.35–7.26 (m, 4H), 7.21 (d, J = 7.6 Hz, 2H), 5.85 (d, J = 7.5 Hz, $1H_{maj}$), 5.73 (s, $1H_{min}$), 5.36 (d, J = 7.5 Hz, $1H_{maj}$), 5.16 (s, $1H_{min}$), 4.43–4.36 (m, 2H), 4.31–4.11 (m, 3H), 2.66 (q, J = 7.6 Hz, 2H), 1.25 (t, J = 7.6 Hz, 3 H), 1.24 (t, J = 7.0 Hz, 3H).

^{13}C-NMR: (126 MHz, $CDCl_3$): δ = 171.15, 155.52, 144.78, 144.01, 143.90, 141.41, 134.03, 128.57, 127.81, 127.23, 127.18, 125.22, 120.09, 67.23, 62.01, 57.88, 47.28, 28.65, 15.50, 14.15.

ESI-HRMS: calculated for $C_{27}H_{27}NNaO_4$ ($[M+Na]^+$): 452.18378, found: 452.18319.

HPLC: (Chiralpak IB-3 column, *n*-heptane/*i*-PrOH 95:5, 298 K, 254 nm): t_R (minor) = 11.1 min, t_R (major) = 14.7 min, e.r. = 97.5:2.5 (95% e.e.).

$[\propto]_D^{25}$**:** –64.4 (c = 0.17, $CHCl_3$).

r.r.: >20:1 (favoring the *para* isomer), determined via ^{1}H-NMR analysis.

ethyl (*R*)-2-((((9H-fluoren-9-yl)methoxy)carbonyl)amino)-2-(4-propylphenyl)acetate (1c)

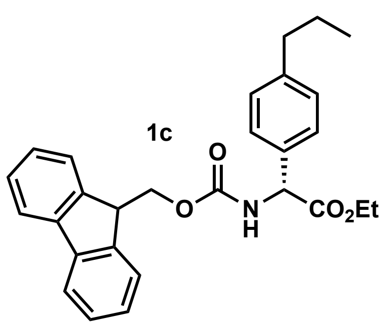

1c

Prepared following *General Procedure D*, from *N*,*O*-acetal **2c** (38.4 mg, 0.1 mmol, 1.0 equiv.) and *n*-propylbenzene (**3c**, 1.0 mL) using catalyst **4g** (11.7 mg, 5 µmol, 5 mol%) over 5 d reaction time at 30 °C. Purification by silica gel flash column chromatography (eluent: *i*-hexanes/MTBE 4:1) gave **1c** as white solid (21 mg, 47 µmol, 47%).

TLC: R_f (*i*-hexanes/EtOAc 3:1) = 0.68.

^{1}H-NMR: (501 MHz, $CDCl_3$): mixture of two rotamers with a ratio of 1:0.19. δ = 7.76 (d, J = 7.6 Hz, 2H), 7.59 (d, J = 7.5 Hz, 2H), 7.39 (t, J = 7.5 Hz, 2H), 7.33–7.20 (m, 4H), 7.18 (d, J = 7.7 Hz, 3H), 5.80 (d, J = 7.5 Hz, $1H_{maj}$), 5.67 (s, $1H_{min}$), 5.34 (d, J = 7.5 Hz,

1H$_{maj}$), 5.14 (s, 1H$_{min}$), 4.39 (qd, J = 10.7, 7.2 Hz, 2H), 4.29–4.10 (m, 3H), 2.58 (t, J = 7.7 Hz, 2H), 1.64 (h, J = 7.4 Hz, 2H), 1.23 (t, J = 7.1 Hz, 3H), 0.94 (t, J = 7.3 Hz, 3H).

^{13}C-NMR: (126 MHz, $CDCl_3$): δ = 171.18, 155.55, 144.03, 143.92, 143.32, 141.43, 134.02, 129.19, 127.84, 127.21, 127.15, 125.24, 120.12, 67.26, 62.03, 57.90, 47.30, 37.86, 24.56, 14.18, 14.00.

ESI-HRMS: calculated for $C_{28}H_{29}NNaO_4$ ($[M+Na]^+$): 466.19943, found: 466.19925.

HPLC: (Chiralpak IB-3 column, *n*-heptane/*i*-PrOH 96:4, 298 K, 254 nm): t_R (minor) = 12.4 min, t_R (major) = 17.1 min, e.r. = 95:5 (90% e.e.).

$[\alpha]_D^{25}$: –56.0 (c = 0.10, $CHCl_3$).

r.r. >20:1 (favoring the *para* isomer), determined via ^{1}H-NMR analysis.

ethyl (*R*)-2-((((9H-fluoren-9-yl)methoxy)carbonyl)amino)-2-(4-butylphenyl)acetate (1d)

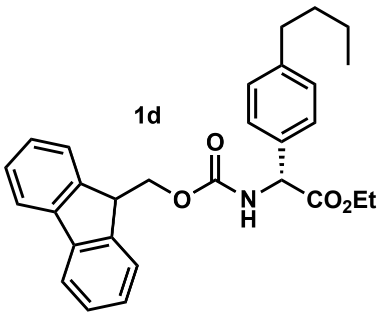

1d

Prepared following *General Procedure D*, from *N,O*-acetal **2c** (38.4 mg, 0.1 mmol, 1.0 equiv.) and *n*-butylbenzene (**3d**, 1.0 mL) using catalyst **4g** (11.7 mg, 5 µmol, 5 mol%) over 5 d reaction time at 30 °C. Purification by silica gel flash column chromatography (eluent: *i*-hexanes/MTBE 4:1) gave **1d** as white solid (22 mg, 48 µmol, 48%).

TLC: R_f (*i*-hexanes/MTBE 2:1) = 0.83.

^{1}H-NMR: (501 MHz, $CDCl_3$): mixture of two rotamers with a ratio of 1:0.19. δ = 7.76 (d, J = 7.6 Hz, 2H), 7.59 (d, J = 7.5 Hz, 2H), 7.40 (t, J = 7.5 Hz, 2H), 7.34–7.26 (m, 4H), 7.18 (d, J = 7.7 Hz, 2H), 5.81 (d, J = 7.5 Hz, 1H$_{maj}$), 5.68 (s, 1H$_{min}$), 5.35 (d, J = 7.5 Hz, 1H$_{maj}$), 5.14 (s, 1H$_{min}$), 4.39 (qd, J = 10.7, 7.2 Hz, 2H), 4.31–4.11 (m, 3H), 2.61 (t, J = 7.8 Hz, 2H), 1.60 (p, J = 7.6 Hz, 2H), 1.36 (h, J = 7.4 Hz, 2H), 1.23 (t, J = 7.1 Hz, 3H), 0.93 (t, J = 7.3 Hz, 3H).

^{13}C-NMR: (126 MHz, $CDCl_3$): δ = 171.18, 155.54, 144.03, 143.91, 143.53, 141.43, 133.98, 129.13, 127.83, 127.20, 127.16, 125.24, 120.11, 67.26, 62.02, 57.90, 47.30, 35.47, 33.61, 22.52, 14.17, 14.08.

ESI-HRMS: calculated for $C_{29}H_{31}NNaO_4$ ($[M+Na]^+$): 480.21508, found: 480.21486.

HPLC: (Chiralpak IB-3 column, *n*-heptane/*i*-PrOH 95:5, 298 K, 254 nm): t_R (minor) = 10.4 min, t_R (major) = 13.9 min, e.r. = 96:4 (92% e.e.).

$[\alpha]_D^{25}$: –63.9 (c = 0.12, $CHCl_3$).

r.r. >20:1 (favoring the *para* isomer), determined via ^{1}H-NMR analysis.

ethyl (*R*)-2-((((9H-fluoren-9-yl)methoxy)carbonyl)amino)-2-(4-isopropylphenyl)acetate (1e)

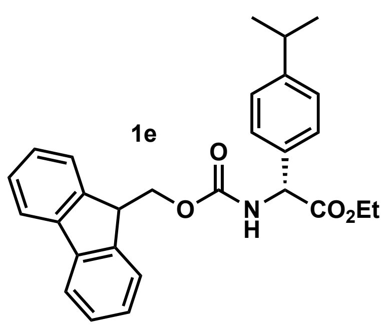

Prepared following *General Procedure D* with deviation, from *N*,*O*-acetal **2c** (38.4 mg, 0.1 mmol, 1.0 equiv.) and cumene (**3e**, 1.0 mL) using catalyst **4f** (13.4 mg, 5 µmol, 5 mol%) over 3 d reaction time in a 4 mL screwcap vial under an atmosphere of argon. Purification by silica gel flash column chromatography (eluent: *i*-hexanes/MTBE 4:1) gave **1e** as white solid (25 mg, 56 µmol, 56%).

TLC: R_f (*i*-hexanes/MTBE 2:1) = 0.69.

^{1}H-NMR: (501 MHz, $CDCl_3$): mixture of two rotamers with a ratio of 1.0:0.21. δ = 7.76 (d, J = 7.6 Hz, 2H), 7.59 (d, J = 7.5 Hz, 2H), 7.40 (t, J = 7.5 Hz, 2H), 7.34–7.24 (m, 4H), 7.23 (d, J = 7.9 Hz, 2H), 5.80 (d, J = 7.4 Hz, $1H_{maj}$), 5.67 (s, $1H_{min}$), 5.35 (d, J = 7.5 Hz, $1H_{maj}$), 5.15 (s, $1H_{min}$), 4.39 (qd, J = 10.7, 7.2 Hz, 2H), 4.31–4.08 (m, 3H), 2.91 (hept, J = 7.0 Hz, 1H), 1.31 – 1.20 (m, 9H).

^{13}C-NMR: (126 MHz, $CDCl_3$): δ = 171.18, 155.55, 149.40, 144.03, 143.91, 141.43, 134.08, 127.83, 127.21, 127.18, 125.24, 120.11, 67.26, 62.03, 57.89, 47.30, 33.96, 24.03, 14.18.

ESI-HRMS: calculated for $C_{28}H_{29}NNaO_4$ ($[M+Na]^+$): 466.19888, found: 466.19887.

HPLC: (Chiralpak IB-3 column, *n*-heptane/*i*-PrOH 95:5, 298 K, 254 nm): t_R (minor) = 10.7 min, t_R (major) = 15.4 min, e.r. = 97:3 (94% e.e.).

$[\alpha]_D^{25}$: –78.5 (c = 0.11, $CHCl_3$).

r.r. >20:1 (favoring the *para* isomer), determined via ^{1}H-NMR analysis.

ethyl (*R*)-2-((((9H-fluoren-9-yl)methoxy)carbonyl)amino)-2-(4-(tert-butyl)phenyl)acetate (1f)

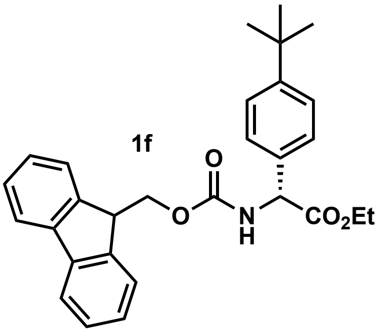

Prepared following *General Procedure D*, from *N*,*O*-acetal **2c** (38.4 mg, 0.1 mmol, 1.0 equiv.) and *t*-butylbenzene (**3f**, 1.0 mL) using catalyst **4g** (11.7 mg, 5 µmol, 5 mol%) over 5 d reaction time at 30 °C. Purification by silica gel flash column chromatography (eluent: *i*-hexanes/MTBE 4:1) gave **1f** as white solid (16 mg, 35 µmol, 35%).

TLC: R_f (*i*-hexanes/MTBE 2:1) = 0.78.

^{1}H-NMR: (501 MHz, CDCl3): mixture of two rotamers with a ratio of 1.0:0.20. δ = 7.76 (d, J = 7.6 Hz, 2H), 7.59 (d, J = 7.5 Hz, 2H), 7.42–7.36 (m, 4H), 7.34–7.28 (m, 4H), 5.80 (d, J = 7.6 Hz, $1H_{maj}$), 5.66 (s, $1H_{min}$), 5.36 (d, J = 7.5 Hz, $1H_{maj}$), 5.16 (s, $1H_{min}$), 4.39 (qd, J = 10.7, 7.2 Hz, 2H), 4.32–4.11 (m, 3H), 1.32 (s, 9H), 1.24 (t, J = 7.1 Hz, 3H).

^{13}C-NMR: (126 MHz, $CDCl_3$): δ = 171.17, 155.57, 151.67, 144.03, 143.91, 141.43, 133.68, 127.83, 127.21, 126.94, 126.06, 125.24, 120.11, 67.26, 62.03, 57.80, 47.30, 34.74, 31.43, 14.19.

ESI-HRMS: calculated for $C_{29}H_{31}NNaO_4$ ($[M+Na]^+$): 480.21508, found: 480.21474.

HPLC: (Chiralpak IB-3 column, *n*-heptane/*i*-PrOH 96:4, 298 K, 254 nm): t_R (minor) = 11.4 min, t_R (major) = 17.3 min, e.r. = 97:3 (94% e.e.).

$[\propto]_D^{25}$: –71.2 (c = 0.12, $CHCl_3$).

r.r. 93.5:6.5 (favoring the *para* isomer), determined via HPLC analysis.

ethyl (*R*)-2-((((9H-fluoren-9-yl)methoxy)carbonyl)amino)-2-(4-cyclopropylphenyl)acetate (1g)

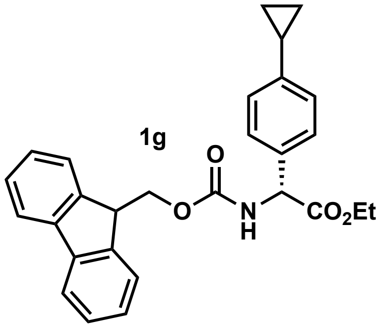

Prepared following *General Procedure D*, from *N*,*O*-acetal **2c** (38.4 mg, 0.1 mmol, 1.0 equiv.) and cyclopropylbenzene (**3g**, 1.0 mL) using catalyst **4g** (11.7 mg, 5 µmol, 5 mol%) over 6 d reaction time at 20 °C. Purification by silica gel flash column chromatography (eluent: *i*-hexanes/MTBE 4:1) gave **1g** as white solid (34 mg, 77 µmol, 77%).

TLC: R_f (*i*-hexanes/MTBE 2:1) = 0.63.

^{1}H-NMR: (501 MHz, $CDCl_3$): mixture of two regioisomers with a ratio of 1.0:0.05 and two rotamers of the main regioisomer with a ratio of 1.0:0.21. δ = 7.76 (d, J = 7.6 Hz, 2H), 7.58 (d, J = 7.6 Hz, 2H), 7.40 (t, J = 7.6 Hz, 2H), 7.34–7.15 (m, 4H), 7.06 (d, J = 7.8 Hz, 2H), 5.87 (d, J = 9.7 Hz, $1H_{min}$), 5.81 (d, J = 7.4 Hz, $1H_{maj}$), 5.70 (s, $1H_{min}$), 5.40 (d, J = 9.7 Hz, $1H_{min}$), 5.32 (d, J = 7.4 Hz, $1H_{maj}$), 5.12 (s, $1H_{min}$), 4.49 (s, $1H_{min}$), 4.38 (qd, J = 10.8, 7.2 Hz, $2H_{maj}$), 4.30–4.10 (m, $3H_{maj}$), 3.68 (s, $1H_{min}$), 1.89 (tt, J = 8.6, 4.9 Hz, 1H), 1.33 (t, J = 7.4 Hz, $3H_{min}$), 1.23 (t, J = 7.2 Hz, $3H_{maj}$), 1.01–0.94 (m, 2H), 0.73–0.66 (m, 2H).

^{13}C-NMR: (126 MHz, $CDCl_3$): δ = 171.13, 155.50, 144.72, 144.01, 143.91, 141.41, 133.78, 127.93 (C_{min}), 127.82 (C_{maj}), 127.20, 126.31, 125.23, 120.10, 67.24, 62.29 (C_{min}), 62.03 (C_{maj}), 57.84, 47.29, 15.30, 14.16, 9.54.

ESI-HRMS: calculated for $C_{28}H_{27}NNaO_4$ ($[M+Na]^+$): 464.18322, found: 464.18325.

HPLC: (Chiralpak IB-3 column, *n*-heptane/*i*-PrOH 95:5, 298 K, 254 nm): t_R (minor) = 14.4 min, t_R (major) = 17.8 min, e.r. = 96:4 (92% e.e.).

$[\propto]_D^{25}$: –82.0 (c = 0.18, $CHCl_3$).

r.r. 95:5 (favoring the *para* isomer), determined via ^{1}H-NMR analysis.

ethyl (*R*)-2-((((9H-fluoren-9-yl)methoxy)carbonyl)amino)-2-(3,4-dimethylphenyl)acetate (1h)

1h

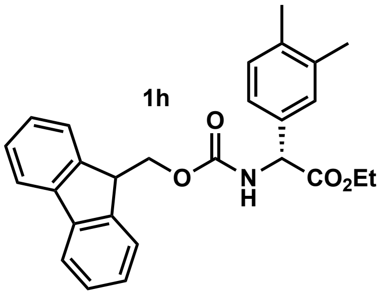

Prepared following *General Procedure D*, from *N*,*O*-acetal **2c** (38.4 mg, 0.1 mmol, 1.0 equiv.) and *o*-xylene (**3h**, 1.0 mL) using catalyst **4g** (11.7 mg, 5 µmol, 5 mol%) over 6 d reaction time at 0 °C. Purification by silica gel flash column chromatography (eluent: *i*-hexanes/MTBE 4:1) gave **3h** as white solid (34 mg, 77 µmol, 77%).

TLC: R_f (*i*-hexanes/MTBE 2:1) = 0.64.

^{1}H-NMR: (501 MHz, $CDCl_3$): mixture of two rotamers with a ratio of 1:0.20. δ = 7.76 (d, J = 7.6 Hz, 2H), 7.59 (d, J = 7.4 Hz, 2H), 7.39 (t, J = 7.5 Hz, 2H), 7.34–7.23 (m, 2H), 7.18–7.02 (m, 3H), 5.80 (d, J = 7.5 Hz, $1H_{maj}$), 5.67 (s, $1H_{min}$), 5.30 (d, J = 7.4 Hz, $1H_{maj}$), 5.12 (s, $1H_{min}$), 4.44–4.32 (m, 2H), 4.30–4.09 (m, 3H), 2.27 (s, 3H), 2.25 (s, 3H), 1.23 (t, J = 7.1 Hz, 3H).

^{13}C-NMR: (126 MHz, $CDCl_3$): δ = 171.25, 155.52, 144.03, 143.94, 141.41, 137.43, 137.24, 134.22, 130.31, 128.51, 127.82, 127.19, 125.24, 124.66, 120.10, 67.26, 62.01, 57.92, 47.28, 19.97, 19.64, 14.18.

ESI-HRMS: calculated for $C_{27}H_{27}NNaO_4$ ($[M+Na]^+$): 452.18323, found: 452.18340.

HPLC: (Chiralpak IB-3 column, *n*-heptane/*i*-PrOH 96:4, 298 K, 254 nm): t_R (minor) = 13.3 min, t_R (major) = 18.1 min, e.r. = 94.5:4.5 (89% e.e.).

$[\propto]_D^{25}$**:** –81.0 (c = 0.11, $CHCl_3$).

r.r. >20:1 (favoring the *para* isomer), determined via HPLC analysis and ^{1}H-NMR analysis.

ethyl (*R*)-2-((((9H-fluoren-9-yl)methoxy)carbonyl)amino)-2-(3,4-diethylphenyl)acetate (1i)

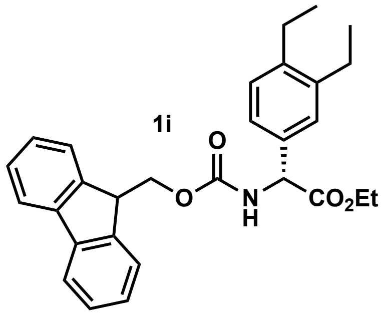

Prepared following *General Procedure D*, from *N,O*-acetal **2c** (38.4 mg, 0.1 mmol, 1.0 equiv.) and 1,2-diethylbenzene (**3i**, 1.0 mL) using catalyst **4g** (11.7 mg, 5 µmol, 5 mol%) over 6 d reaction time at 0 °C. Purification by silica gel flash column chromatography (eluent: *i*-hexanes/MTBE 4:1) gave **1i** as white solid (38 mg, 79 µmol, 79%).

TLC: R_f (*i*-hexanes/MTBE 2:1) = 0.74.

^{1}H-NMR: (501 MHz, $CDCl_3$): mixture of two rotamers with a ratio of 1:0.21. δ 7.76 (d, J = 7.7 Hz, 2H), 7.60 (d, J = 7.5 Hz, 2H), 7.40 (t, J = 7.5 Hz, 2H), 7.34–7.28 (m, 2H), 7.23–7.09 (m, 3H), 5.79 (d, J = 7.5 Hz, $1H_{maj}$), 5.66 (s, $1H_{min}$), 5.34 (d, J = 7.6 Hz, $1H_{maj}$), 5.18 (s, $1H_{min}$), 4.39 (dt, J = 18.2, 9.1 Hz, 2H), 4.31–4.12 (m, 3H), 2.70–2.61 (m, 4H), 1.29–1.19 (m, 9H).

^{13}C-NMR: (126 MHz, $CDCl_3$): δ = 171.28, 155.57, 144.03, 143.93, 142.60, 142.32, 141.41, 134.10, 128.96, 127.81, 127.19, 125.27, 125.24, 124.71, 120.10, 67.27, 61.95, 57.98, 47.28, 25.65, 25.31, 15.29, 15.19, 14.18.

ESI-HRMS: calculated for $C_{29}H_{31}NNaO_4$ ($[M+Na]^+$): 480.21453, found: 480.21494.

HPLC: (Chiralpak IB-3 column, *n*-heptane/*i*-PrOH 95:5, 298 K, 254 nm): t_R (minor) = 9.7 min, t_R (major) = 12.7 min, e.r. = 96.5:3.5 (93% e.e.).

$[\propto]_D^{25}$**:** –74.8 (c = 0.11, $CHCl_3$).

r.r. >20:1 (favoring the *para* isomer), determined via HPLC analysis and ^{1}H-NMR analysis.

ethyl (*R*)-2-((((9H-fluoren-9-yl)methoxy)carbonyl)amino)-2-phenylacetate (1j)

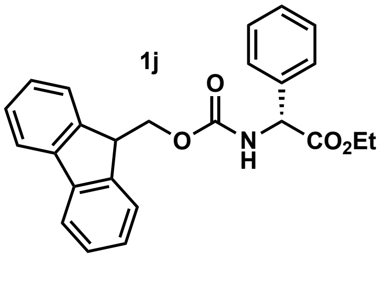

Prepared following *General Procedure D* with some deviations, from *N,O*-acetal **2c** (9.6 mg, 0.025 mmol, 1.0 equiv.) and benzene (**1j**, 0.25 mL) using catalyst **4i** (6.9 mg, 2.5 µmol, 10 mol%) over 5 d reaction time at 60 °C in a 1 mL pressure vial. Purification by preparative TLC (eluent: *i*-hexanes/EtOAc 3:1) gave **1j** as white solid (5.3 mg, 13 µmol, 53%).

TLC: R_f (*i*-hexanes/EtOAc 3:1) = 0.63.

^{1}H-NMR: (501 MHz, $CDCl_3$): mixture of two rotamers with a ratio of 1:0.19. δ 7.76 (d, J = 7.6 Hz, 2H), 7.59 (d, J = 7.5 Hz, 2H), 7.44–7.28 (m, 9H), 5.86 (d, J = 7.4 Hz, $1H_{maj}$), 5.75 (s, $1H_{min}$), 5.38 (d, J = 7.5 Hz, $1H_{maj}$), 5.15 (s, $1H_{min}$), 4.40 (tt, J = 8.1, 7.4 Hz, 2H), 4.31–4.09 (m, 3H), 1.23 (t, J = 7.1 Hz, 3H).

^{13}C-NMR: (126 MHz, $CDCl_3$): δ = 170.98, 155.51, 144.00, 143.88, 141.43, 136.87, 129.09, 128.67, 127.84, 127.26, 127.21, 125.22, 120.12, 67.27, 62.12, 58.13, 47.30, 14.14.

ESI-HRMS: calculated for $C_{25}H_{23}NNaO_4$ ($[M+Na]^+$): 424.15193, found: 424.15206.

HPLC: (Chiralpak IB-3 column, *n*-heptane/*i*-PrOH 95:5, 298 K, 254 nm): t_R (minor) = 13.3 min, t_R (major) = 19.3 min, e.r. = 81:19 (62% e.e.).

$[\alpha]_D^{25}$: –79.4 (c = 0.19, $CHCl_3$).

5.2 Friedel–Crafts Reaction of Anisoles and Heteroarenes

ethyl (*R*)-2-((((9H-fluoren-9-yl)methoxy)carbonyl)amino)-2-(4-methoxyphenyl)acetate (1k)

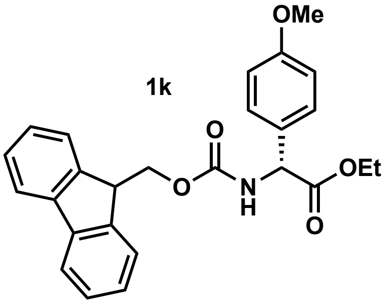

Prepared following *General Procedure E*, from *N,O*-acetal **2c** (38.4 mg, 0.1 mmol, 1.0 equiv.) and anisole (**3k**, 22 µL, 0.2 mmol, 2.0 equiv.) in *n*-pentane (1.0 mL) using catalyst **4f** (13.4 mg, 5 µmol, 5 mol%) over 20 h reaction time at 30 °C. Purification by silica gel flash column chromatography (eluent: *i*-hexanes/EtOAc 10:1 ⟶ 3:1) gave **1k** as white solid (40 mg, 92 µmol, 92%).

TLC: R_f (*i*-hexanes/EtOAc 3:1) = 0.55.

^{1}H-NMR: (501 MHz, CD_2Cl_2): mixture of two regioisomers with a ratio of 1:0.02 and two rotamers of the main regioisomer with a ratio of 1:0.17. δ = 7.78 (d, J = 7.6 Hz, 2H), 7.61 (d, J = 7.5 Hz, 2H), 7.41 (t, J = 7.5 Hz, 2H), 7.35–7.17 (m, 4H), 6.90 (d, J = 8.3 Hz, 2H), 5.95 (d, J = 8.6 Hz, 1Hmin), 5.86 (d, J = 7.3 Hz, $1H_{maj}$), 5.64 (s, $1H_{min}$), 5.46 (d, J = 8.6 Hz, $1H_{min}$), 5.26 (d, J = 7.2 Hz, $1H_{maj}$), 5.06 (s, $1H_{min}$), 4.44–4.33 (m, 2H), 4.26–4.09 (m, 3H), 3.84 (s, $3H_{min}$), 3.80 (s, $3H_{maj}$), 1.21 (t, J = 7.2 Hz, 3H).

Exemplary experiment at decreased temperature for detailed analysis of rotamers:

(600 MHz, CD_2Cl_2, 233 K): mixture of two regioisomers with a ratio of 1.0:0.02 and two rotamers of the main regioisomer with a ratio of 1.0:0.13. δ = 7.78 (dt, J = 7.6, 1.0 Hz, $2H_{maj}$), 7.75 (dt, J = 7.6, 0.9 Hz, $2H_{min}$) 7.59 (ddq, J = 7.4, 1.9, 0.9 Hz, 2H), 7.40 (t, J = 7.4 Hz, $2H_{maj}$), 7.37 (t, J = 7.5 Hz, $2H_{min}$), 7.30 (tdd, J = 7.3, 4.5, 1.0 Hz, $2H_{maj}$), 7.29–

7.26 (m, $2H_{maj}$), 7.25–7.21 (m, $2H_{min}$), 7.20–7.16 (m, $2H_{min}$), 6.95 (td, J = 7.4, 1.0 Hz, $2H_{min}$) 6.91–6.83 (m, $2H_{maj}$), 6.02 (d, J = 7.0 Hz, $1H_{maj}$), 5.87 (d, J = 6.6 Hz, $1H_{min}$), 5.37 (d, J = 8.7 Hz, $1H_{min}$), 5.21 (d, J = 6.9 Hz, $1H_{maj}$), 5.09 (d, J = 7.5 Hz, $1H_{min}$), 5.06 (d, J = 6.6 Hz, $1H_{min}$), 4.39–4.34 (m, 1H), 4.33–4.28 (m, 1H), 4.22 – 4.12 (m, 2H), 4.06 (dq, J = 10.8, 7.1 Hz, 1H), 3.78 (s, $3H_{min}$), 3.76 (s, $3H_{maj}$), 1.16 (t, J = 7.2 Hz, $3H_{maj}$), 1.15 (t, J = 7.1 Hz, $3H_{min}$).

^{13}C-NMR: (126 MHz, CD_2Cl_2): δ = 171.30, 160.20, 155.64, 144.41, 144.33, 141.68, 129.36, 128.77, 128.06, 127.44, 125.45, 120.33, 114.59, 67.27, 62.27, 57.88, 55.69, 47.60, 14.22.

Exemplary experiment at decreased temperature for detailed analysis of rotamers:

(151 MHz, CD_2Cl_2, 233 K): δ = 170.93 (C_{maj}), 170.64 (C_{min}), 159.41 (C_{maj}), 159.37 (C_{min}), 155.06 (C_{maj}), 154.70 (C_{min}), 143.75 (d, J = 19.0 Hz, C_{maj}), 143.70 (d, J = 19.1 Hz, C_{min}), 141.06 (d, J = 2.6 Hz, C_{maj}), 140.99 (d, J = 14.4 Hz, C_{min}), 129.02 (C_{min}), 128.60 (C_{min}), 128.42 (C_{maj}), 128.25 (C_{min}), 127.65 (C_{maj}), 127.60 (d, J = 5.5 Hz, C_{min}), 127.02 (d, J = 2.3 Hz, C_{maj}), 126.97 (C_{min}), 125.05 (d, J = 2.9 Hz, C_{maj}), 124.94 (d, J = 11.0 Hz, C_{min}), 119.98 (C_{maj}), 119.96 (C_{min}), 119.88 (C_{min}), 113.93 (C_{min}), 113.91 (C_{maj}), 67.19 (C_{min}), 66.61 (C_{maj}), 62.22 (C_{min}), 62.09 (C_{maj}), 57.52 (C_{min}), 57.15 (C_{maj}), 55.30, 46.77 (C_{maj}), 46.67 (C_{min}), 13.83 (C_{maj}), 13.82 (C_{min}).

ESI-HRMS: calculated for $C_{26}H_{25}NNaO_5$ ($[M+Na]^+$): 454.16249, found: 454.16295.

2D-LC: (1. dimension: 100 mm Zorbax RX-SIL, *n*-heptane/*i*-PrOH 99:1, 308 K, 220 nm): t_R (major regioisomer) = 3.2 min, t_R (minor regioisomer) = 3.8 min.

(2. dimension: Chiralpak IC-3, *n*-heptane/*i*-PrOH 70:30, 298 K, 220 nm): t_R (minor enantiomer) = 8.0 min, t_R (major enantiomer) = 12.6 min, e.r. = 97.5:2.5 (95% e.e.).

$[\alpha]_D^{25}$: –86.6 (c = 0.13, $CHCl_3$).

r.r. 72:1 (favoring the *para* isomer), determined via HPLC analysis.

ethyl (*R*)-2-((((9H-fluoren-9-yl)methoxy)carbonyl)amino)-2-(4-(methoxy-*d*$_3$)phenyl)acetate (1l)

1l

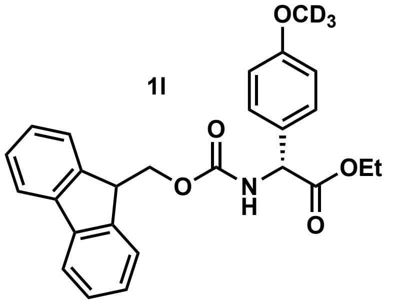

Prepared following *General Procedure E*, from *N*,*O*-acetal **2c** (38.4 mg, 0.1 mmol, 1.0 equiv.) and (methoxy-*d*$_3$)benzene (**3l**, 22 µL, 0.2 mmol, 2.0 equiv.) in *n*-pentane (1.0 mL) using catalyst **4f** (13.4 mg, 5 µmol, 5 mol%) over 20 h reaction time at 30 °C. Purification by silica gel flash column chromatography (eluent: *i*-hexanes/EtOAc 10:1 ⟶ 3:1) gave **1l** as white solid (41 mg, 95 µmol, 95%).

TLC: R_f (*i*-hexanes/EtOAc 3:1) = 0.55.

^{1}H-NMR: (501 MHz, $CDCl_3$): mixture of two rotamers with a ratio of 1:0.18. δ = 7.76 (d, J = 7.6 Hz, 2H), 7.59 (d, J = 7.5 Hz, 2H), 7.40 (t, J = 7.5 Hz, 2H), 7.35–7.17 (m, 4H), 6.89 (d, J = 8.3 Hz, 2H), 5.81 (d, J = 7.2 Hz, $1H_{maj}$), 5.68 (s, $1H_{min}$), 5.31 (d, J = 7.3 Hz, $1H_{maj}$), 5.08 (s, $1H_{min}$), 4.45–4.34 (m, 2H), 4.30–4.10 (m, 3H), 1.23 (t, J = 7.2 Hz, 3H).

^{13}C-NMR: (126 MHz, CDCl3): δ 171.22, 159.86, 155.50, 144.02, 143.91, 141.43, 128.94, 128.53, 127.83, 127.20, 125.23, 120.11, 114.46, 67.23, 62.03, 57.57, 47.30, 14.17.

ESI-HRMS: calculated for $C_{26}H_{22}D_3NNaO_5$ ($[M+Na]^+$): 457.18187, found: 457.18131.

2D-LC: (1. dimension: 100 mm Zorbax RX-SIL, *n*-heptane/*i*-PrOH 99:1, 308 K, 220 nm): t_R (major regioisomer) = 3.2 min, t_R (minor regioisomer) = 3.8 min.

(2. dimension: Chiralpak IC-3, *n*-heptane/*i*-PrOH 65:35, 298 K, 220 nm): t_R (minor enantiomer) = 7.1 min, t_R (major enantiomer) = 11.0 min, e.r. = 97.5:2.5 (95% e.e.).

$[\propto]_D^{25}$**:** –85.3 (c = 0.12, $CHCl_3$).

r.r. 68:1 (favoring the *para* isomer), determined via HPLC analysis.

ethyl (*R*)-2-((((9H-fluoren-9-yl)methoxy)carbonyl)amino)-2-(4-(methylthio)phenyl)acetate (1m)

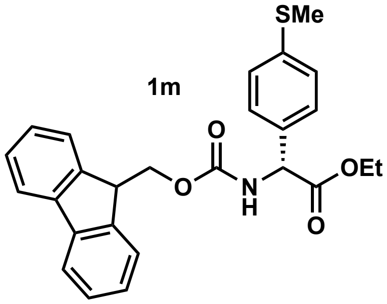

Prepared following *General Procedure E*, from *N*,*O*-acetal **2c** (38.4 mg, 0.1 mmol, 1.0 equiv.) and methyl(phenyl)sulfane (**3m**, 24 µL, 0.2 mmol, 2.0 equiv.) in *n*-pentane (1.0 mL) using catalyst **4f** (13.4 mg, 5 µmol, 5 mol%) over 36 h reaction time at 30 °C. Purification by silica gel flash column chromatography (eluent: *i*-hexanes/EtOAc 10:1 ⟶ 3:1) gave **1m** as white solid (33 mg, 74 µmol, 74%).

TLC: R_f (*i*-hexanes/MTBE 2:1) = 0.58.

^{1}H-NMR: (501 MHz, $CDCl_3$): mixture of two rotamers with a ratio of 1:0.22. δ = 7.76 (d, J = 7.6 Hz, 2H), 7.58 (d, J = 7.6 Hz, 2H), 7.40 (t, J = 7.5 Hz, 2H), 7.34–7.27 (m, 4H), 7.26–7.16 (m, 4H), 5.87 (d, J = 7.3 Hz, $1H_{maj}$), 5.76 (s, $1H_{min}$), 5.33 (d, J = 7.3 Hz, $1H_{maj}$), 5.07 (s, $1H_{min}$), 4.48–4.33 (m, 2H), 4.29–4.10 (m, 3H), 2.48 (s, 3H), 1.23 (t, J = 7.1 Hz, 3H).

^{13}C-NMR: (126 MHz, $CDCl_3$): δ = 170.86, 155.45, 143.96, 143.86, 141.42, 139.32, 133.59, 127.84, 127.69, 127.19, 126.88, 125.19, 120.12, 67.24, 62.17, 57.67, 47.28, 15.73, 14.14.

ESI-HRMS: calculated for $C_{26}H_{25}NNaO_4S$ ($[M+Na]^+$): 470.13965, found: 470.13952.

2D-LC: (1. dimension: 250 mm PVA-SIL, *n*-heptane/*i*-PrOH 99:1, 308 K, 220 nm): t_R (major regioisomer) = 16.3 min, t_R (minor regioisomer) = 17.5 min.

(2. dimension: Chiralpak IC-3, *n*-heptane/*i*-PrOH 65:35, 298 K, 220 nm): t_R (minor enantiomer) = 7.1 min, t_R (major enantiomer) = 9.2 min, e.r. = 97:3 (94% e.e.).

$[\alpha]_D^{25}$: –99.0 (c = 0.10, $CHCl_3$).

r.r. 42:1 (favoring the *para* isomer), determined via HPLC analysis.

ethyl (*R*)-2-((((9H-fluoren-9-yl)methoxy)carbonyl)amino)-2-(4-methoxy-3-methylphenyl)acetate (1n)

1n

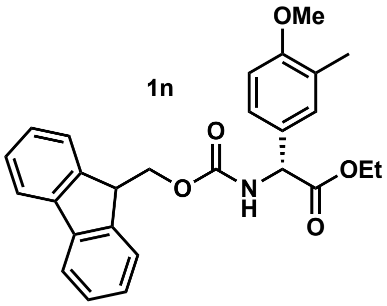

Prepared following *General Procedure E*, from *N,O*-acetal **2c** (38.4 mg, 0.1 mmol, 1.0 equiv.) and 1-methoxy-2-methylbenzene (**1n**, 25 µL, 0.2 mmol, 2.0 equiv.) in *n*-pentane (1.0 mL) using catalyst **4f** (13.4 mg, 5 µmol, 5 mol%) over 16 h reaction time at 30 °C. Purification by silica gel flash column chromatography (eluent: *i*-hexanes/EtOAc 10:1 ⟶ 3:1) gave **1n** as white solid (42 mg, 94 µmol, 94%).

TLC: R_f (*i*-hexanes/EtOAc 3:1) = 0.55.

^{1}H-NMR: (501 MHz, $CDCl_3$): mixture of two rotamers with a ratio of 1:0.20. δ = 7.76 (d, J = 7.6 Hz, 2H), 7.59 (d, J = 7.5 Hz, 2H), 7.39 (t, J = 7.5 Hz, 2H), 7.34–7.27 (m, 2H), 7.23–7.07 (m, 2H), 6.80 (d, J = 8.4 Hz, 1H), 5.77 (d, J = 7.3 Hz, $1H_{maj}$), 5.65 (s, $1H_{min}$), 5.27 (d, J = 7.4 Hz, $1H_{maj}$), 5.09 (s, $1H_{min}$), 4.46–4.33 (m, 2H), 4.31–4.09 (m, 3H), 3.83 (s, 3H), 2.22 (s, 3H), 1.23 (t, J = 7.1 Hz, 3H).

^{13}C-NMR: (126 MHz, $CDCl_3$): δ = 171.38, 158.08, 155.52, 144.05, 143.95, 141.43, 129.52, 128.39, 127.83, 127.50, 127.20, 125.91, 125.24, 120.11, 110.22, 67.24, 61.98, 57.66, 55.51, 47.30, 16.45, 14.19.

ESI-HRMS: calculated for $C_{27}H_{27}NNaO_5$ ($[M+Na]^+$): 468.17814, found: 468.17859.

HPLC: (Chiralpak AD-3 column, *n*-heptane/*i*-PrOH 94:6, 298 K, 254 nm): t_R (minor) = 19.1 min, t_R (major) = 20.5 min, e.r. = 95.5:4.5 (91% e.e.).

$[\alpha]_D^{25}$: –90.8 (c = 0.13, $CHCl_3$).

r.r. >20:1 (favoring the *para* isomer), determined via ^{1}H-NMR analysis.

ethyl (*R*)-2-((((9H-fluoren-9-yl)methoxy)carbonyl)amino)-2-(benzo[*d*][1,3]dioxol-5-yl)acetate (1o)

1o

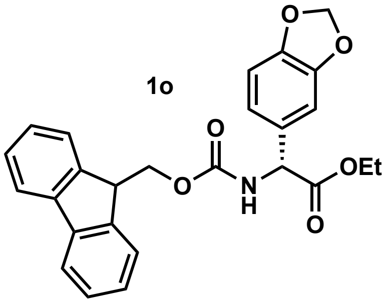

Prepared following *General Procedure E*, from *N,O*-acetal **2c** (38.4 mg, 0.1 mmol, 1.0 equiv.) and benzo[*d*][1,3]dioxole (**3o**, 23 µL, 0.2 mmol, 2.0 equiv.) in *n*-pentane (1.0 mL) using catalyst **4f** (13.4 mg, 5 µmol, 5 mol%) over 36 h reaction time at 30 °C. Purification by silica gel flash column chromatography (eluent: *i*-hexanes/EtOAc 10:1 ⟶ 3:1) gave **1o** as white solid (42 mg, 94 µmol, 94%).

TLC: R_f (*i*-hexanes/EtOAc 3:1) = 0.53.

^{1}H-NMR: (501 MHz, $CDCl_3$): mixture of two rotamers with a ratio of 1:0.20. δ = 7.76 (d, J = 7.6 Hz, 2H), 7.58 (d, J = 7.5 Hz, 2H), 7.40 (t, J = 7.5 Hz, 2H), 7.31 (t, J = 7.5 Hz, 2H), 6.90–6.69 (m, 3H), 5.97 (s, 2H), 5.83 (d, J = 7.2 Hz, $1H_{maj}$), 5.67 (s, $1H_{min}$), 5.26 (d, J = 7.2 Hz, $1H_{maj}$), 4.99 (s, $1H_{min}$), 4.51–4.33 (m, 2H), 4.29–4.08 (m, 3H), 1.24 (t, J = 7.2 Hz, 3H).

^{13}C-NMR: (126 MHz, $CDCl_3$): δ = 170.96, 155.44, 148.21, 147.95, 143.98, 143.87, 141.43, 130.66, 127.84, 127.20, 125.21, 120.96, 120.12, 108.70, 107.65, 101.45, 67.25, 62.16, 57.81, 47.28, 14.16.

ESI-HRMS: calculated for $C_{26}H_{23}NNaO_6$ ($[M+Na]^+$): 468.14175, found: 468.14216.

HPLC: (Chiralpak IB-3 column, *n*-heptane/*i*-PrOH 95:5, 298 K, 254 nm): t_R (minor) = 20.6 min, t_R (major) = 24.1 min, e.r. = 96:4 (92% e.e.).

$[\propto]_D^{25}$: –70.5 (c = 0.11, $CHCl_3$).

r.r. >20:1 (favoring the *para* isomer), determined via ^{1}H-NMR analysis.

ethyl (*R*)-2-((((9H-fluoren-9-yl)methoxy)carbonyl)amino)-2-(3-iodo-4-methoxyphenyl)acetate (1p)

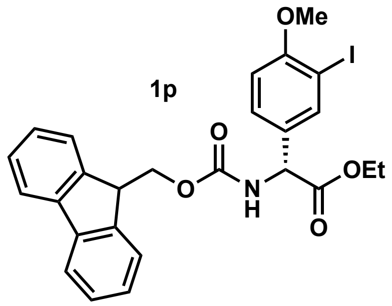

Prepared following *General Procedure E*, from *N*,*O*-acetal **2c** (38.4 mg, 0.1 mmol, 1.0 equiv.) and 1-iodo-2-methoxybenzene (**3p**, 26 µL, 0.2 mmol, 2.0 equiv.) in CyMe (1.0 mL) using catalyst **4f** (13.4 mg, 5 µmol, 5 mol%) over 6 d reaction time at 30 °C. Purification by silica gel flash column chromatography (eluent: *i*-hexanes/MTBE 4:1 ⟶ 2:1) gave **1p** as white solid (42 mg, 75 µmol, 75%).

TLC: R_f (*i*-hexanes/EtOAc 3:1) = 0.52.

^{1}H-NMR: (501 MHz, $CDCl_3$): mixture of two regioisomers with a ratio of 1:0.02 and two rotamers of the main regioisomer with a ratio of 1:0.17. δ = 7.84–7.70 (m, 3H), 7.59 (d, J = 7.5 Hz, 2H), 7.40 (t, J = 7.5 Hz, 2H), 7.36–7.15 (m, 1H), 7.32 (q, J = 7.8 Hz, 2H), 6.79 (d, J = 8.4 Hz, 1H), 5.95 (d, J = 7.4 Hz, $1H_{min}$), 5.87 (d, J = 7.2 Hz, $1H_{maj}$), 5.71 (s, $1H_{min}$), 5.44 (d, J = 7.2 Hz, $1H_{min}$), 5.27 (d, J = 7.2 Hz, $1H_{maj}$), 4.99 (s, $1H_{min}$), 4.53–4.33 (m, 2H), 4.32–4.09 (m, 3H), 3.88 (s, 3H), 1.23 (t, J = 7.1 Hz, 3H).

^{13}C-NMR: (126 MHz, CDCl3): δ = 170.70, 158.40, 155.40, 143.93, 143.84, 141.42, 138.06, 131.06, 128.70, 127.85, 127.23, 125.20, 120.12, 111.03, 86.47, 67.30, 62.28, 56.84, 56.57, 47.26, 14.16.

ESI-HRMS: calculated for $C_{26}H_{24}NNaO_5I$ ($[M+Na]^+$): 580.05914, found: 580.05978.

**HPLC:**	(Chiralpak IB-3 column, *n*-heptane/*i*-PrOH 97:3, 298 K, 254 nm): t_R (minor) = 36.1 min, t_R (major) = 40.1 min, e.r. = 95.5:4.5 (91% e.e.).
$[\alpha]_D^{25}$:	–66.7 (c = 0.11, $CHCl_3$).
**r.r.**	>20:1 (favoring the *para* isomer), determined via ^{1}H-NMR analysis.

ethyl (*R*)-2-((((9H-fluoren-9-yl)methoxy)carbonyl)amino)-2-(4-methoxy3 (trimethylsilyl)-phenyl)acetate (1q)

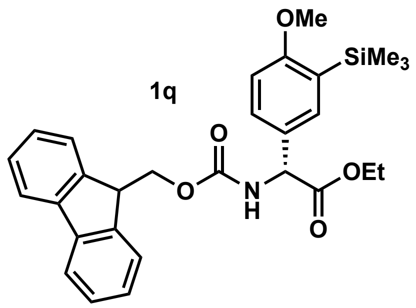

Prepared following *General Procedure E*, from *N,O*-acetal **2c** (38.4 mg, 0.1 mmol, 1.0 equiv.) and (2-methoxyphenyl)trimethylsilane (**3q**, 38 µL, 0.2 mmol, 2.0 equiv.) in CyMe (1.0 mL) using catalyst **4f** (13.4 mg, 5 µmol, 5 mol%) over 20 h reaction time at 30 °C. Purification by silica gel flash column chromatography (eluent: *i*-hexanes/MTBE 3:1) gave **1q** as white solid (42 mg, 83 µmol, 83%). *Since usage of bistriflimide as catalyst for the synthesis of a racemic sample exclusively gave the protodesilylated product* ***1k****, HOTf was used as achiral acid catalyst.*

**TLC:**	R_f (*i*-hexanes/MTBE 2:1) = 0.55.
**^{1}H-NMR:**	(501 MHz, $CDCl_3$): mixture of two rotamers with a ratio of 1:0.19. δ = 7.77 (d, J = 7.6 Hz, 2H), 7.60 (dd, J = 7.5, 2.7 Hz, 2H), 7.40 (t, J = 7.5 Hz, 2H), 7.37–7.28 (m, 4H), 6.81 (d, J = 9 Hz, 1H), 5.77 (d, J = 7.5 Hz, $1H_{maj}$), 5.64 (s, $1H_{min}$), 5.34 (d, J = 7.5 Hz, $1H_{maj}$), 5.19 (s, $1H_{min}$), 4.40 (qd, J = 10.7, 7.2 Hz, 2H), 4.30–4.13 (m, 3H), 3.81 (s, 3H), 1.25 (t, J = 7.1 Hz, 3H), 0.28 (s, 9H).
**^{13}C-NMR:**	(126 MHz, $CDCl_3$): δ = 171.37, 164.61, 155.56, 144.03, 143.93, 141.41, 133.99, 129.65, 129.05, 128.32, 127.82, 127.19, 125.26, 125.23, 120.10, 109.84, 67.26, 61.92, 57.72, 55.29, 47.28, 14.19, –0.91.
**ESI-HRMS:**	calculated for $C_{29}H_{33}NNaO_5Si$ ($[M+Na]^+$): 526.20202, found: 526.20223.
**HPLC:**	(Chiralpak IB-3 column, *n*-heptane/*i*-PrOH 95:5, 298 K, 254 nm): t_R (minor) = 9.7 min, t_R (major) = 11.9 min, e.r. = 96:4 (92% e.e.).
$[\alpha]_D^{25}$:	–71.3 (c = 0.12, $CHCl_3$).
**r.r.**	>20:1 (favoring the *para* isomer), determined via ^{1}H-NMR analysis.

ethyl (*R*)-2-((((9H-fluoren-9-yl)methoxy)carbonyl)amino)-2-(4-(2-bromoethoxy)phenyl)acetate (1r)

1r

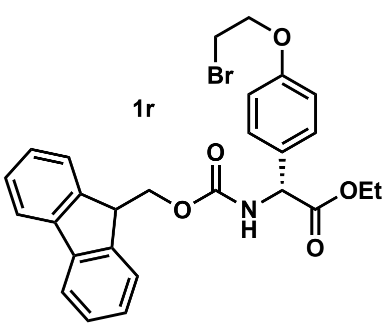

Prepared following *General Procedure E*, from *N*,*O*-acetal **2c** (38.4 mg, 0.1 mmol, 1.0 equiv.) and (2-bromoethoxy)benzene (**3r**, 40 mg, 0.2 mmol, 2.0 equiv.) in *n*-pentane (1.0 mL) using catalyst **4f** (13.4 mg, 5 µmol, 5 mol%) over 3 d reaction time at 30 °C. Purification by silica gel flash column chromatography (eluent: *i*-hexanes/MTBE 3:1 ⟶ 2:1) gave **1r** as white solid (50 mg, 95 µmol, 95%).

TLC: R_f (*i*-hexanes/EtOAc 2:1) = 0.68.

^{1}H-NMR: (501 MHz, $CDCl_3$): mixture of two rotamers with a ratio of 1:0.23. δ = 7.76 (d, J = 7.6 Hz, 2H), 7.58 (d, J = 7.5 Hz, 2H), 7.40 (t, J = 7.5 Hz, 2H), 7.35–7.17 (m, 4H), 6.90 (d, J = 8.4 Hz, 2H), 5.83 (d, J = 7.3 Hz, $1H_{maj}$), 5.70 (s, $1H_{min}$), 5.31 (d, J = 7.2 Hz, $1H_{maj}$), 5.06 (s, $1H_{min}$), 4.45–4.34 (m, 2H), 4.29 (t, J = 6.2 Hz, 2H), 4.27–4.10 (m, 3H), 3.63 (t, J = 6.2 Hz, 2H), 1.22 (t, J = 7.1 Hz, 3H).

^{13}C-NMR: (126 MHz, $CDCl_3$): δ = 171.09, 158.36, 155.47, 143.99, 143.89, 141.43, 129.84, 128.64, 127.84, 127.20, 125.21, 120.12, 115.25, 68.03, 67.24, 62.10, 57.51, 47.29, 29.10, 14.16.

ESI-HRMS: calculated for $C_{27}H_{26}BrNNaO_5$ ($[M+Na]^+$): 546.08866, found: 546.08899.

HPLC: (Chiralpak AD-3 column, *n*-heptane/*i*-PrOH 92:8, 298 K, 254 nm): t_R (minor) = 40.0 min, t_R (major) = 44.7 min, e.r. = 98:2 (96% e.e.).

$[\alpha]_D^{25}$: –61.2 (c = 0.24, $CHCl_3$).

r.r. >20:1(favoring the *para* isomer), determined via ^{1}H-NMR analysis.

ethyl (R)-2-((((9H-fluoren-9-yl)methoxy)carbonyl)amino)-2-(4-(2-azidoethoxy)phenyl)acetate (1s)

1s

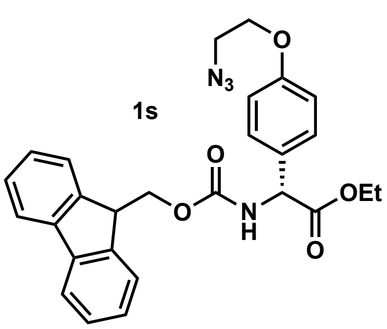

Prepared following *General Procedure E*, from *N,O*-acetal **2c** (38.4 mg, 0.1 mmol, 1.0 equiv.) and (2-azidoethoxy)benzene (**3s**, 32.6 mg, 0.2 mmol, 2.0 equiv.) in *n*-pentane (1.0 mL) using catalyst **4f** (13.4 mg, 5 µmol, 5 mol%) over 3 d reaction time at 30 °C. Purification by silica gel flash column chromatography (eluent: *i*-hexanes/MTBE 3:1) gave **1s** as white solid (42 mg, 86 µmol, 86%).

TLC: R_f (*i*-hexanes/EtOAc 2:1) = 0.53.

^{1}H-NMR: (501 MHz, $CDCl_3$): mixture of two rotamers with a ratio of 1:0.28. δ = 7.76 (d, J = 7.7 Hz, 2H), 7.58 (d, J = 7.6 Hz, 2H), 7.40 (t, J = 7.5 Hz, 2H), 7.36–7.17 (m, 4H), 6.91

(d, J = 8.4 Hz, $2H_{maj}$), 6.16 (s, $2H_{min}$), 5.83 (d, J = 7.3 Hz, $1H_{maj}$), 5.69 (s, $1H_{min}$), 5.31 (d, J = 7.3 Hz, $1H_{maj}$), 5.07 (s, $1H_{min}$), 4.48–4.34 (m, 2H), 4.31–4.17 (m, 3H), 4.15 (t, J = 5.0 Hz, 2H), 3.60 (t, J = 4.9 Hz, 2H), 1.33 (t, J = 7.2 Hz, $3H_{min}$), 1.22 (t, J = 7.1 Hz, $3H_{maj}$).

^{13}C-NMR: (126 MHz, $CDCl_3$): δ = 171.12, 158.51, 155.49, 144.00, 143.90, 141.44, 129.79, 128.63, 127.85, 127.21, 125.22, 120.13, 115.13, 67.25, 67.19, 62.10, 57.53, 50.26, 47.30, 14.17.

ESI-HRMS: calculated for $C_{27}H_{26}N_4NaO_5$ ($[M+Na]^+$): 509.17954, found: 509.17994.

HPLC: *achiral HPLC (for the determination of the regioisomeric ratio):*

(Eclipse Plus C18, MeOH/H_2O – gradient: 60:40 – 5' – 90:10, 308 K, 220 nm): t_R (minor) = 4.60 min, t_R (major) = 4.7 min, r.r. = 98.5:1.5.

chiral HPLC (for the determination of the enantiomeric ratio):

(chiralpak OJ-3 column, MeOH 100%, 298 K, 2204 nm): t_R (major) = 6.9 min, t_R (minor) = 10.4 min, e.r. = 98:2 (96% e.e.).

$[\propto]_D^{25}$: –81.1 (c = 0.11, $CHCl_3$).

r.r. 98.1:1.5 (favoring the *para* isomer), determined via HPLC analysis.

ethyl (*R*)-2-((((9H-fluoren-9-yl)methoxy)carbonyl)amino)-2-(4-(allyloxy)phenyl)acetate (1t)

1t

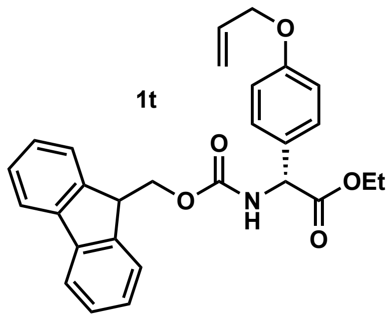

Prepared following *General Procedure E*, from *N,O*-acetal **2c** (38.4 mg, 0.1 mmol, 1.0 equiv.) and (allyloxy)benzene (**3t**, 27 µL, 0.2 mmol, 2.0 equiv.) in *n*-pentane (1.0 mL) using catalyst **4f** (13.4 mg, 5 µmol, 5 mol%) over 16 h reaction time at 30 °C. Purification by silica gel flash column chromatography (eluent: *i*-hexanes/MTBE 5:1 ⟶ 3:1) gave **1t** as white solid (42 mg, 92 µmol, 92%).

TLC: R_f (*i*-hexanes/MTBE 2:1) = 0.67.

^{1}H-NMR: (501 MHz, $CDCl_3$): mixture of two rotamers with a ratio of 1:0.21. δ = 7.76 (d, J = 7.6 Hz, 2H), 7.59 (d, J = 7.5 Hz, 2H), 7.40 (t, J = 7.5 Hz, 2H), 7.34–7.16 (m, 4H), 6.91 (d, J = 8.3 Hz, 2H), 6.05 (ddt, J = 16.2, 10.5, 5.3 Hz, 1H), 5.80 (d, J = 7.3 Hz, $1H_{maj}$), 5.67 (s, $1H_{min}$), 5.41 (d, J = 17.3 Hz, 1H), 5.35–5.26 (m, 2H), 5.08 (s, $1H_{min}$), 4.54 (dt, J = 5.3, 1.6 Hz, 2H), 4.47–4.34 (m, 2H), 4.32–4.07 (m, 3H), 1.22 (t, J = 7.1 Hz, 3H).

^{13}C-NMR: (126 MHz, $CDCl_3$): δ = 171.21, 158.91, 155.51, 144.02, 143.91, 141.43, 133.21, 129.10, 128.51, 127.83, 127.21, 125.23, 120.12, 117.97, 115.26, 69.00, 67.23, 62.03, 57.55, 47.30, 14.17.

ESI-HRMS: calculated for $C_{28}H_{27}N_1NaO_5$ ($[M+Na]^+$): 480.17814, found: 480.17845.

HPLC: (Chiralpak AD-3 column, *n*-heptane/*i*-PrOH 92:8, 298 K, 254 nm): t_R (minor) = 21.4 min, t_R (major) = 23.2 min, e.r. = 96.5:3.5 (93% e.e.).

$[\propto]_{D}^{25}$: –80.0 (c = 0.14, $CHCl_3$).

r.r. >20.1 (favoring the *para* isomer), determined via ^{1}H-NMR analysis.

ethyl (R)-2-((((9H-fluoren-9-yl)methoxy)carbonyl)amino)-2-(4-(prop-2-yn-1-yloxy)phenyl)acetate (1u)

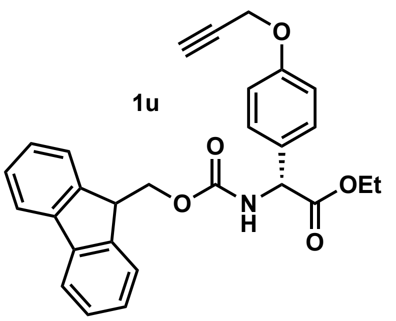

Prepared following *General Procedure E*, from *N*,*O*-acetal **2c** (38.4 mg, 0.1 mmol, 1.0 equiv.) and (prop-2-yn-1-yloxy)benzene)benzene (**3u**, 25.7 µL, 0.2 mmol, 2.0 equiv.) in *n*-pentane (1.0 mL) using catalyst **4f** (13.4 mg, 5 µmol, 5 mol%) over 3 d reaction time at 30 °C. Purification by silica gel flash column chromatography (eluent: *i*-hexanes/MTBE 3:1) gave **1u** as white solid (45 mg, 98 µmol, 98%).

TLC: R_f (*i*-hexanes/EtOAc 2:1) = 0.65.

^{1}H-NMR: (501 MHz, $CDCl_3$): mixture of two rotamers with a ratio of 1:0.20. δ = 7.76 (d, J = 7.6 Hz, 2H), 7.58 (d, J = 7.5 Hz, 2H), 7.40 (t, J = 7.5 Hz, 2H), 7.36–7.17 (m, 4H), 6.97 (d, J = 8.3 Hz, 2H), 5.82 (d, J = 7.3 Hz, $1H_{maj}$), 5.69 (s, $1H_{min}$), 5.32 (d, J = 7.3 Hz, $1H_{maj}$), 5.08 (s, $1H_{min}$), 4.69 (d, J = 2.4 Hz, 2H), 4.50–4.33 (m, 2H), 4.31–4.07 (m, 3H), 2.53 (t, J = 2.4 Hz, 1H), 1.23 (t, J = 7.1 Hz, 3H).

^{13}C-NMR: (126 MHz, $CDCl_3$): δ = 171.10, 157.83, 155.49, 144.00, 143.88, 141.43, 129.92, 128.55, 127.84, 127.21, 125.22, 120.12, 115.40, 78.49, 75.86, 67.23, 62.09, 57.52, 55.97, 47.30, 14.16.

ESI-HRMS: calculated for $C_{28}H_{25}N_1NaO_5$ ($[M+Na]^+$): 478.16249, found: 478.16271.

HPLC: (Chiralpak AD-3 column, *n*-heptane/*i*-PrOH 92:8, 298 K, 254 nm): t_R (minor) = 35.0 min, t_R (major) = 41.2 min, e.r. = 98:2 (96% e.e.).

$[\propto]_{D}^{25}$: –78.6 (c = 0.20, $CHCl_3$).

r.r. >20:1 (favoring the *para* isomer), determined via HPLC analysis.

ethyl (*R*)-2-((((9H-fluoren-9-yl)methoxy)carbonyl)amino)-2-(thipohen-2-yl)acetate (1v)

1v

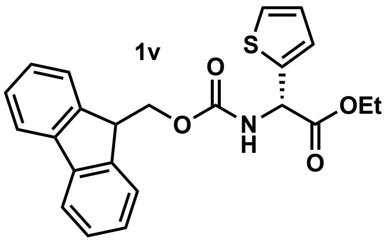

Prepared following *General Procedure E*, from *N*,*O*-acetal **2c** (38.4 mg, 0.1 mmol, 1.0 equiv.) and thiophene (**3v**, 16 µL, 0.2 mmol, 2.0 equiv.) in CyMe (1.0 mL) using catalyst **4f** (13.4 mg, 5 µmol, 5 mol%) over 5 d reaction time at 30 °C. Purification by silica gel flash column chromatography (eluent: *i*-hexanes/MTBE 4:1) gave **1v** as white solid (39 mg, 96 µmol, 96%).

TLC: R_f (*i*-hexanes/EtOAc 2:1) = 0.74.

^{1}H-NMR: (600 MHz, $CDCl_3$, 253 K): mixture of two regioisomers with a ratio of 1:0.15 and two rotamers of the main regioisomer with a ratio of 1:0.19. δ = 7.78 (d, J = 7.6 Hz, 2H), 7.62–7.57 (m, 2H), 7.42 (t, J = 7.5 Hz, 2H), 7.33 (dddd, J = 8.5, 7.5, 2.0, 1.2 Hz, 2H), 7.29 (dd, J = 5.2, 1.2 Hz, 1H), 7.07 (dt, J = 3.6, 1.1 Hz, 1H), 6.99 (dd, J = 5.1, 3.6 Hz, 1H), 5.92 (d, J = 7.7 Hz, $1H_{maj}$), 5.82 (d, J =7.7 Hz, $1H_{min}$), 5.80 (d, J = 7.2 Hz, $1H_{min}$), 5.74 (d, J = 7.2 Hz, $1H_{min}$), 5.65 (dd, J = 7.7, 0.9 Hz, $1H_{maj}$), 5.49 (d, J = 7.0 Hz, $1H_{min}$), 5.48 (d, J = 7.7 Hz, $1H_{min}$), 5.28 (d, J = 7.1 Hz, $1H_{min}$), 4.48–4.34 (m, 2H), 4.32–4.15 (m, 3H), 1.28 (t, J = 7.1 Hz, $3H_{maj}$), 1.26 (t, J = 7.3 Hz, $3H_{min}$).

^{13}C-NMR: (126 MHz, $CDCl_3$ at 253 K): δ = 170.65 (C_{min}), 170.05, 169.45 (C_{min}), 155.45 (C_{min}), 155.33, 154.69 (C_{min}), 143.75 (C_{min}), 143.67, 143.60, 143.59 (C_{min}), 143.54 (C_{min}), 141.27 (C_{min}), 141.25, 141.24, 141.22 (C_{min}), 139.51 (C_{min}), 138.82, 136.63 (C_{min}), 127.83, 127.79 (C_{min}), 127.77 (C_{min}), 127.31 (C_{min}), 127.28, 127.17, 126.99 (C_{min}), 126.26, 126.20 (C_{min}), 126.06, 126.04 (C_{min}), 125.98 (C_{min}), 125.22, 125.19, 125.17 (C_{min}), 125.15 (C_{min}), 125.06 (C_{min}), 125.03 (C_{min}), 123.26 (C_{min}), 120.13, 120.08 (C_{min}), 67.72 (C_{min}), 67.23, 67.02 (C_{min}), 62.73 (C_{min}), 62.60, 62.31 (C_{min}), 54.12 (C_{min}), 53.72 (C_{min}), 53.46, 46.97 (C_{min}), 46.91, 46.83 (C_{min}), 14.18 (C_{min}), 14.15.

ESI-HRMS: calculated for $C_{23}H_{21}N_1NaO_4S$ ($[M+Na]^+$): 430.10835, found: 430.10874.

HPLC: (Chiralpak IB-3 column, *n*-heptane/*i*-PrOH 95:5, 298 K, 254 nm): t_R (minor) = 14.1 min, t_R (major) = 19.2 min, e.r. = 94.5:5.5 (89% e.e.).

$[\propto]_D^{25}$: –51.9 (c = 0.13, $CHCl_3$).

r.r. 86.5:13.5 (favoring the 2-position), determined via HPLC analysis. The connectivity of the thiophene ring of the major regioisomer to the glycine scaffold was determined based on the coupling of the signals of the thiophene ring in the ^{1}H-NMR (signals at 7.29, 7.07 and 6.99 ppm).

ethyl (*R*)-2-((((9H-fluoren-9-yl)methoxy)carbonyl)amino)-2-(benzo[*b*]thiophen-2-yl)acetate (1w)

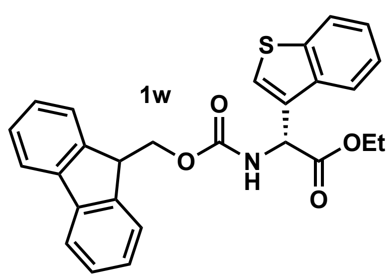

Prepared following *General Procedure E*, from *N*,*O*-acetal **2c** (38.4 mg, 0.1 mmol, 1.0 equiv.) and benzo[*b*]thiophen (**3w**, 23 µL, 0.2 mmol, 2.0 equiv.) in *n*-pentane (1.0 mL) using catalyst **4f** (13.4 mg, 5 µmol, 5 mol%) over 4 d reaction time at 30 °C. Purification by silica gel flash column chromatography (eluent: *i*-hexanes/MTBE 4:1) gave **1w** as white solid (45 mg, 98 µmol, 98%).

TLC: R_f (*i*-hexanes/EtOAc 3:1) = 0.58.

^{1}H-NMR: (600 MHz, $CDCl_3$, 233 K): mixture of two regioisomers with a ratio of 1:0.12 and two rotamers of the main regioisomer with a ratio of 1:0.16. δ = 7.92 (ddd, J = 7.9, 1.4, 0.7 Hz, 1H), 7.90 (ddd, J = 7.7, 1.3, 0.6 Hz, 1H), 7.77 (dd, J = 7.6, 0.9 Hz, 2H), 7.72 (t, J = 7.8 Hz, $1H_{min}$), 7.61 (td, J = 7.7, 0.9 Hz, $1H_{min}$), 7.58 (ddd, J = 7.5, 1.9, 1.0 Hz, 2H), 7.47–7.43 (m, $2H_{min}$), 7.42 (d, J = 0.7 Hz, 1H), 7.39–7.33 (m, $2H_{min}$), 7.30 (dtd, J = 10.8, 7.5, 1.1 Hz, 2H), 7.22–7.20 (m, $1H_{min}$), 7.11–7.07 (m, 1 H_{min}), 6.92 (td, J = 7.5, 1.1 Hz, $1H_{min}$), 6.80 (td, J = 7.5, 1.1 Hz, $1H_{min}$), 6.10 (d, J = 7.5 Hz, $1H_{min}$), 6.04 (d, J = 6.5 Hz, $1H_{min}$), 5.92 (d, J = 6.8 Hz, $1H_{min}$), 5.89 (d, J = 7.7 Hz, $1H_{maj}$), 5.79 (dd, J = 7.7, 0.7 Hz, 1H, $_{maj}$), 5.73 (dd, 7.5, 0.9 Hz, $1H_{min}$), 5.53 (dd, J = 6.5, 1.0 Hz, $1H_{min}$), 5.45 (dd, J = 6.7, 0.8 Hz, $1H_{min}$), 4.51–4.43 (m, $2H_{min}$), 4.43–4.35 (m, 2H), 4.29 (dd, J = 10.8, 7.2 Hz, 1H), 4.23–4.20 (m, 1H), 4.16 (dd, J = 10.8, 7.1 Hz, 1H), 4.06 (dq, J = 10.8, 7.1 Hz, $1H_{min}$), 1.29 (t, J = 7.2 Hz, $3H_{min}$), 1.22 (t, J = 7.1 Hz, 3H, 27) 1.16 (t, J = 7.1 Hz, $3H_{min}$).

^{13}C-NMR: (151 MHz, $CDCl_3$ at 233 K): δ = 170.70, 169.76 (C_{min}), 169.61 (C_{min}), 155.51, 155.26 (C_{min}), 154.84 (C_{min}), 143.62, 143.51 (C_{min}), 143.50 (C_{min}), 143.42, 143.39 (C_{min}), 143.38 (C_{min}), 141.15, 141.13, 141.09 (C_{min}), 141.05 (C_{min}), 140.58 (C_{min}), 140.36, 139.62 (C_{min}), 139.38 (C_{min}), 139.04 (C_{min}), 136.73, 136.37 (C_{min}), 131.01 (C_{min}), 130.53, 127.79, 127.78, 127.70 (C_{min}), 127.67 (C_{min}), 127.13, 127.11, 127.03 (C_{min}), 125.74, 125.66 (C_{min}), 125.16, 125.21 (C_{min}), 125.16, 124.95, 124.85 (C_{min}), 124.82 (C_{min}), 124.77 (C_{min}), 124.73 (C_{min}), 124.68, 124.62 (C_{min}), 124.55 (C_{min}), 123.90 (C_{min}), 123.08 (C_{min}), 123.05, 123.01 (C_{min}), 122.44 (C_{min}), 122.14 (C_{min}), 122.00, 120.11, 120.14 (C_{min}), 120.09, 120.06 (C_{min}), 67.16 (C_{min}), 67.12 (C_{min}), 67.09, 62.91 (C_{min}), 62.68 (C_{min}), 62.50, 54.05 (C_{min}), 53.28 (C_{min}), 52.48, 46.75, 46.69 (C_{min}), 14.13, 14.07 (C_{min}).

ESI-HRMS: calculated for $C_{27}H_{23}N_1NaO_4S$ ($[M+Na]^+$): 480.12400, found: 480.12392.

2D-LC (1. dimension: 250 mm PVA-SIL, *n*-heptane/*i*-PrOH 95:5, 308 K, 220 nm): t_R (minor regioisomer) = 7.2 min, t_R (major regioisomer) = 7.7 min.

(2. dimension: Chiralpak IB-3, *n*-heptane/*i*-PrOH 80:20, 298 K, 220 nm): t_R (minor enantiomer) = 7.3 min, t_R (major enantiomer) = 8.4 min, e.r. = 96.5:3.5 (93% e.e.).

$[\alpha]_D^{25}$: –84.1 (c = 0.18, $CHCl_3$).

r.r. 85.5:15.5 (favoring the 3-position), determined via ^{1}H-NMR analysis and HPLC analysis. The connectivity of the benzothiophene ring of the major regioisomer to the glycine scaffold was determined based on the following characteristic cross peaks: HMBC (C19 to H16); NOESY (H16 to H20), see section 11 for further details.

ethyl (*R*)-2-((((9H-fluoren-9-yl)methoxy)carbonyl)amino)-2-(benzofuran-2-yl)acetate (1x)

1x

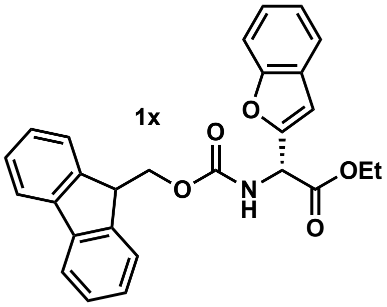

Prepared following *General Procedure E*, from *N*,*O*-acetal **2c** (38.4 mg, 0.1 mmol, 1.0 equiv.) and benzofuran (**3x**, 22 µL, 0.2 mmol, 2.0 equiv.) in CyMe (1.0 mL) using catalyst **4f** (13.4 mg, 5 µmol, 5 mol%) over 3 d reaction time at 30 °C. Purification by silica gel flash column chromatography (eluent: *i*-hexanes/MTBE 4:1) gave **1x** as off-white solid (42 mg, 95 µmol, 95%).

TLC: R_f (*i*-hexanes/MTBE 2:1) = 0.44.

^{1}H-NMR: (600 MHz, $CDCl_3$, 233 K): mixture of two regioisomers with a ratio of 1:0.13 and two rotamers of the main regioisomer with a ratio of 1:0.09. δ 7.78 (dd, J = 7.6, 0.9 Hz, 2H), 7.62 – 7.58 (m, 3H), 7.57–7.52 (m, $2H_{min}$), 7.52–7.45 (m, 1H), 7.43–7.39 (m, 2H), 7.39–7.36 (m, $1H_{min}$), 7.35–7.29 (m, 3H), 7.29–7.24 (m, $1H_{min}$), 7.17 (td, J = 7.5, 1.1 Hz, $1H_{min}$), 7.09 (td, J = 7.5, 1.1 Hz, $1H_{min}$), 6.81 (s, 1H), 6.24 (s, $1H_{min}$), 6.08 (d, J = 8.0 Hz, 1H), 5.94 (d, J = 7.4 Hz, $1H_{min}$), 5.88 (d, J = 7.1 Hz, $1H_{min}$), 5.65 (d, J= 7.9 Hz, 1H), 5.62 (dd, J = 7.4, 0.7 Hz, $1H_{min}$), 5.25 (d, J = 7.1 Hz, $1H_{min}$), 4.53–4.46 (m, $2H_{min}$), 4.43–4.36 (m, 2H), 4.34–4.29 (m, 1H), 4.23 (t, J = 6.7 Hz, 1H), 4.21–4.17 (m, 1H), 1.25 (t, J = 7.2 Hz, 3H).

^{13}C-NMR: (151 MHz, $CDCl_3$ at 233 K): δ = 170.35 (C_{min}), 168.54, 167.89 (C_{min}), 155.43 (C_{min}), 155.39, 155.29 (C_{min}), 154.67, 154.64 (C_{min}), 154.60 (C_{min}), 151.31 (C_{min}), 150.88, 143.58 (C_{min}), 143.54, 143.52 (C_{min}), 143.45, 143.43 (C_{min}), 143.40 (C_{min}), 143.38 (C_{min}), 141.19 (C_{min}), 141.15, 141.14, 141.07 (C_{min}), 127.80, 127.75 (C_{min}), 127.73 (C_{min}), 127.63, 127.13, 127.09 (C_{min}), 125.19, 125.16, 125.11 (C_{min}), 125.04 (C_{min}), 124.92, 124.86 (C_{min}), 124.73 (C_{min}), 123.18, 123.15 (C_{min}), 121.53, 120.17 (C_{min}), 120.12, 120.10, 116.13 (C_{min}), 111.93 (C_{min}), 111.57 (C_{min}), 111.52, 105.94, 105.38 (C_{min}), 67.23, 67.19

(C_{min}), 67.03 (C_{min}), 63.12 (C_{min}), 62.96, 62.62 (C_{min}), 52.58 (C_{min}), 52.16, 49.67 (C_{min}), 46.76 (C_{min}), 46.71, 46.62 (C_{min}).

ESI-HRMS: calculated for $C_{27}H_{23}N_1NaO_5$ ($[M+Na]^+$): 464.14684, found: 464.14681.

2D-LC: (1. dimension: 100 mm Zorbax RX-Sil, *n*-heptane/*i*-PrOH 99.5:0.5, 308 K, 220 nm): t_R (major regioisomer) = 3.1 min, t_R (minor regioisomer) = 3.3 min, r.r. = 82:18.

(2. dimension: Chiralpak IB-3, *n*-heptane/*i*-PrOH 80:20, 298 K, 220 nm): t_R (minor enantiomer) = 5.9 min, t_R (major enantiomer) = 6.9 min, e.r. = 97.5:2.5 (95% e.e.).

$[\propto]_D^{25}$: –95.8 (c = 0.12, $CHCl_3$).

r.r. 82:18 (favoring the 2-position), determined via ^{1}H-NMR analysis and HPLC analysis. The connectivity of the benzofurane ring of the major regioisomer to the glycine scaffold was determined based on the following characteristic cross peak: HMBC (C20 to H18), see section 11 for further details.

5.3 Friedel–Crafts Reaction with Differing N,O-Acetals

ethyl (*R*)-2-(((benzyloxy)carbonyl)amino)-2-(p-tolyl)acetate (S8-a)

S8-a

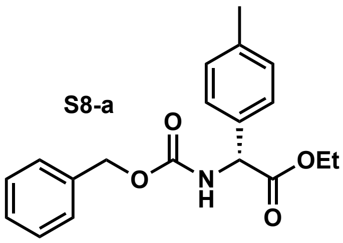

Prepared following *General Procedure D*, from *N,O*-acetal **2a** (29.5 mg, 0.1 mmol, 1.0 equiv.) and toluene (**3a**, 1.0 mL) using catalyst **4b** (8.9 mg, 5 µmol, 5 mol%) over 24 h reaction time at 30 °C. Purification by silica gel flash column chromatography (eluent: *i*-hexanes/MTBE 4:1) gave **S8-a** as colorless oil (15.7 mg, 48.0 µmol, 48%).

TLC: R_f (*i*-hexanes/EtOAc 3:1) = 0.65.

^{1}H-NMR: (501 MHz, $CDCl_3$): mixture of two regioisomers with a ratio of 1:0.05 and two rotamers of the main regioisomer with a ratio of 1:0.27. δ 7.39 – 7.28 (m, 5H), 7.25 (d, J = 7.5 Hz, 2H), 7.16 (d, J = 7.7 Hz, 2H), 5.79 (d, J = 7.4 Hz, $1H_{maj}$), 5.60 (s, $1H_{min}$), 5.32 (d, J = 7.4 Hz, $1H_{maj}$), 5.18 (s, $1H_{min}$), 5.09 (q, J = 12.3 Hz, 2H), 4.30–4.05 (m, 2H), 2.49 (s, $3H_{min}$), 2.34 (s, $3H_{maj}$), 1.21 (t, J = 7.1 Hz, 3H).

^{13}C-NMR: (126 MHz, $CDCl_3$): δ = 171.08, 155.47, 138.50, 136.35, 133.96, 129.74, 128.67, 128.33, 128.31, 127.15, 67.20, 62.01, 57.88, 21.29, 14.14.

ES-HRMS: calculated for $C_{19}H_{21}N_1NaO_4$ ($[M+Na]^+$): 350.13627, found: 350.13632.

HPLC: (Chiralpak IB-3 column, *n*-heptane/*i*-PrOH 96:4, 1 mL/min, 25 °C, 206 nm): t_R (minor) = 7.9 min, t_R (major) = 8.7 min, e.r. = 83.5:16:5 (67% e.e.).

$[\propto]_D^{25}$= –66.7 (c = 0.11, $CHCl_3$).

r.r. 20:1 (favoring the *para* isomer), determined via ^{1}H-NMR analysis.

methyl (*R*)-2-((((9H-fluoren-9-yl)methoxy)carbonyl)amino)-2-(p-tolyl)acetate (S8-b)

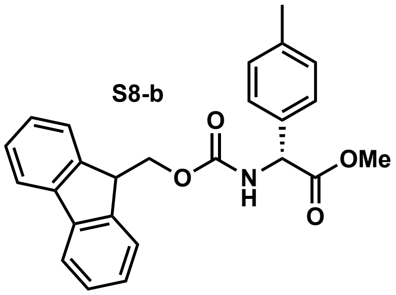

Prepared following *General Procedure D* with deviations, from *N*,*O*-acetal **2e** (36.9 mg, 0.1 mmol, 1.0 equiv.) and toluene (1.0 mL) using catalyst **4b** (8.9 mg, 5 µmol, 5 mol%) over 24 h reaction time at 30 °C. Purification by silica gel flash column chromatography (eluent: *i*-hexanes/MTBE 4:1) gave **S8-b** as white solid (15.1 mg, 37.6 µmol, 38%).

TLC: R_f (*i*-hexanes/MTBE 2:1) = 0.62.

^{1}H-NMR: (501 MHz, $CDCl_3$): mixture of two regioisomers with a ratio of 1:0.07 and two rotamers of the main regioisomer with a ratio of 1:0.18. δ 7.76 (d, J = 7.6 Hz, 2H), 7.58 (d, J = 7.5 Hz, 2H), 7.40 (t, J = 7.5 Hz, 2H), 7.35–7.21 (m, 4H), 7.19 (d, J = 8.0 Hz, 2H), 5.81 (d, J = 7.4 Hz, $1H_{maj}$), 5.74 (d, J = 7.3 Hz, $1H_{min}$), 5.68 (s, $1H_{min}$), 5.62 (d, J = 7.5 Hz, $1H_{min}$), 5.35 (d, J = 7.3 Hz, $1H_{maj}$), 5.11 (s, $1H_{min}$), 4.47–4.32 (m, 2H), 4.22 (t, J = 7.2 Hz, $1H_{maj}$), 4.15 (s, $1H_{min}$), 3.74 (s, $3H_{maj}$), 3.70 (s, $3H_{min}$), 2.49 (s, $1H_{min}$), 2.35 (s, $3H_{maj}$).

^{13}C-NMR: (126 MHz, $CDCl_3$): δ = 171.65, 155.51, 144.00 (C_{min}), 143.90 (C_{maj}), 141.43, 138.70, 133.73, 129.85, 127.84, 127.24, 127.21, 125.22, 120.12, 67.28, 57.83, 52.96, 47.29, 21.31.

ESI-HRMS: calculated for $C_{25}H_{23}N_1NaO_4$ ($[M+Na]^+$): 424.15192, found: 424.15216.

HPLC: (Chiralpak IB-3 column, *n*-heptane/*i*-PrOH 96:4, 1 mL/min, 25 °C, 254 nm): t_R (minor) = 15.6 min, t_R (major) = 33.4 min, e.r. = 90:10 (80% e.e.).

$[\propto]_D^{25}$= –67.9 (c = 0.11, $CHCl_3$).

r.r. 93.5:6.5 (favoring the *para* isomer), determined via ^{1}H-NMR analysis.

isopropyl (*R*)-2-((((9H-fluoren-9-yl)methoxy)carbonyl)amino)-2-(p-tolyl)acetate (S8-c)

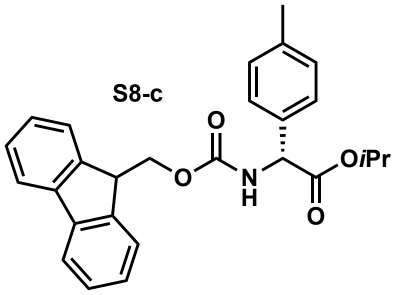

Prepared following *General Procedure D* with deviations, from *N*,*O*-acetal **2d** (39.7 mg, 0.1 mmol, 1.0 equiv.) and toluene (1.0 mL) using catalyst **4b** (8.9 mg, 5 µmol, 5 mol%) over 24 h reaction time at 30 °C. Purification by silica gel flash column chromatography (eluent: *i*-hexanes/MTBE 4:1) afforded **S8-c** as white solid (16.8 mg, 39.1 µmol, 39%).

TLC: R_f (*i*-hexanes/MTBE 2:1) = 0.75.

^{1}H-NMR: (501 MHz, $CDCl_3$): mixture of two regioisomers with a ratio of 1.0:0.03 and two rotamers of the main regioisomer with a ratio of 1.0:0.23. δ 7.76 (d, J = 7.6 Hz, 2H), 7.59 (d, J = 7.5 Hz, 2H), 7.40 (t, J = 7.6 Hz, 2H), 7.35–7.19 (m, 4H), 7.17 (d, J = 7.7 Hz, 2H), 5.83 (d, J = 7.4 Hz, $1H_{maj}$), 5.73 (s, $1H_{min}$), 5.57 (d, J = 7.5 Hz, $1H_{min}$), 5.30 (d, J = 7.4 Hz, $1H_{maj}$), 5.12 (d, J = 5.4 Hz, $1H_{min}$), 5.06 (p, J = 6.2 Hz, 1H), 4.38 (qd, J = 10.7, 7.3 Hz, 2H), 4.22 (t, J = 7.2 Hz, 1H), 4.15 (s, $1H_{min}$), 2.50 (s, $3H_{min}$), 2.35 (s, $3H_{maj}$), 1.28 (d, J = 6.2 Hz, 3H), 1.12 (d, J = 6.2 Hz, 3H).

^{13}C-NMR: (126 MHz, $CDCl_3$): δ = 170.64, 155.51, 144.04, 143.94, 141.42, 138.38, 134.04, 129.70, 127.82, 127.19, 127.10, 125.25, 120.10, 69.80, 67.23, 57.95, 47.30, 21.86, 21.50, 21.30.

ESI-HRMS: calculated for $C_{27}H_{27}N_1NaO_4$ ($[M+Na]^+$): 452.18322, found: 452.18347.

HPLC: (Chiralpak IC-3 column, *n*-heptane/*i*-PrOH 90:10, 1 mL/min, 25 °C, 254 nm): t_R (minor) = 10.9 min, t_R (major) = 19.9 min, e.r. = 89.5:10.5 (79% e.e.).

$[\propto]_D^{25}$= –55.2 (c = 0.14, $CHCl_3$).

r.r. 97:3 (favoring the *para* isomer), determined via ^{1}H-NMR analysis.

6. Gram-Scale Synthesis of Arylglycine Derivates

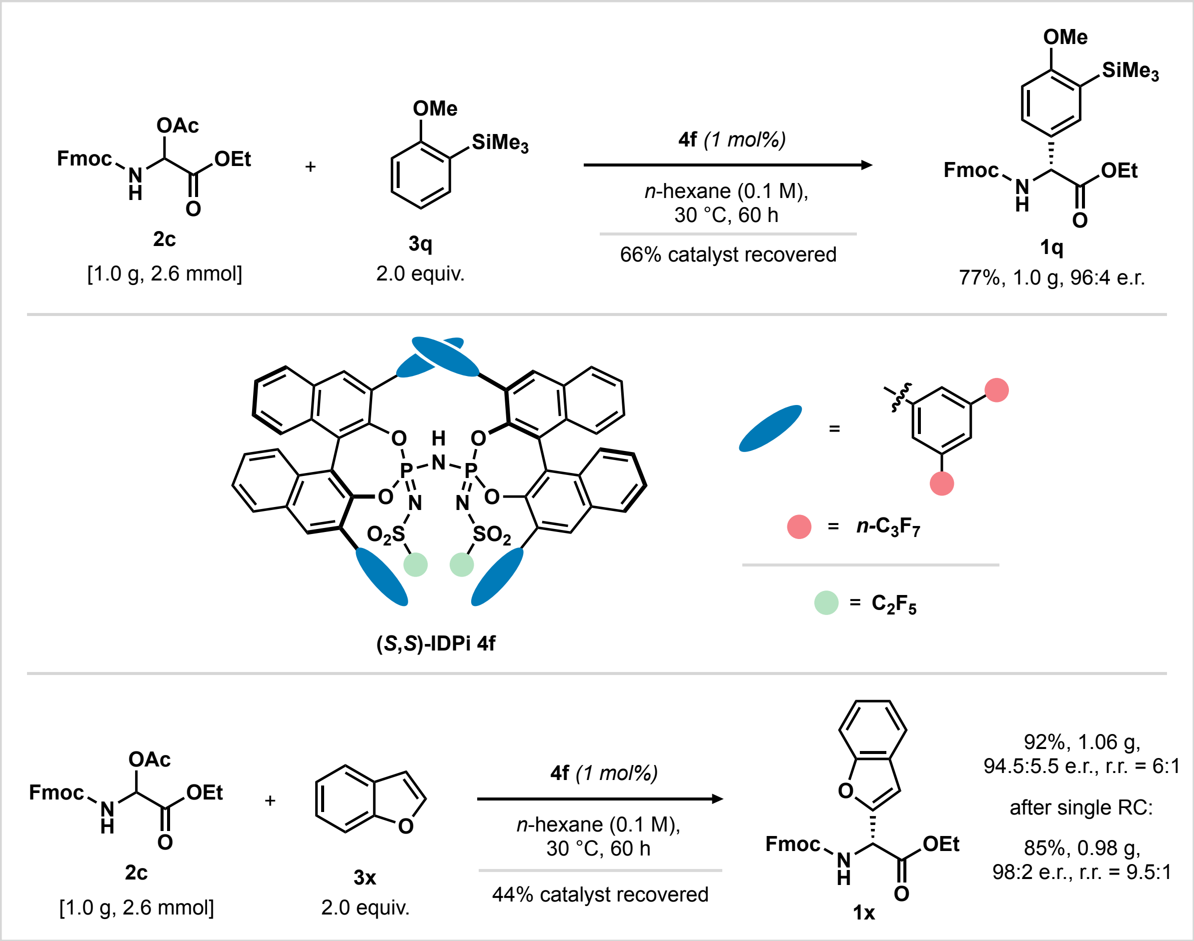

Figure SI-2: Gram-scale synthesis of arylglycine derivates.

Gram-Scale Synthesis of: ethyl (*R*)-2-((((9*H*-fluoren-9-yl)methoxy)carbonyl)amino)-2-(4-methoxy-3-(trimethylsilyl)phenyl)acetate (1q)

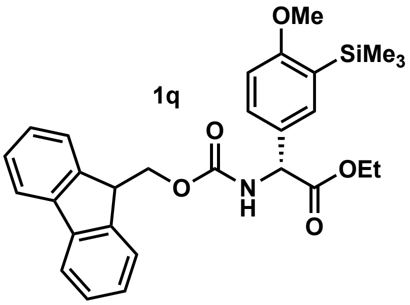

N,*O*-acetal **2c** (1.0 g, 2.60 mmol, 1.0 equiv) was given to a flame-dried 100 mL Schlenk tube under an atmosphere of argon followed by IDPi-catalyst **4f** (70 mg, 26.1 µmol, 1 mol%). The tube was cooled to –78 °C and *n*-hexane (27 mL) was added followed by the dropwise addition of (2-methoxyphenl)trimethylsilane (**3q**, 0.98 mL, 5.22 mmol, 2.0 equiv.). The heterogeneous mixture was allowed to warm to room temperature and then stirred at 30 °C for 60 h whereupon complete conversion of *N*,*O*-acetal **2c** was observed. The reaction was quenched with triethylamine (30 µL), adsorbed on celite® and purified via flash column chromatography on silica gel (eluent: *i*-hexanes/MTBE 5:1 ⟶ 2:1) to yield the desired arylglycine **1q** (1.0 g, 1.99 mmol, 77%) as white solid.

For the analytical data of ***1q*** *isolated herein, see the corresponding entry at section 5.2 of this supporting information.*

To reisolate IDPi catalyst **4f**, the silica gel column was flushed (eluent: CH_2Cl_2/EtOAc 99:1) after complete elution of arylglycine **1q**. The obtained solution was concentrated and the residue was

purified via flash column chromatography on silica gel (eluent: *i*-hexanes/CH_2Cl_2 1:1 ⟶ CH_2Cl_2/EtOAc 99.5:0.5) to yield catalyst **4f** as salt. After acidification over DOWEX 50WX8 (H-form, eluted with CH_2Cl_2), the reisolated catalyst **4f** (46 mg, 17.1 µmol, 66%) was obtained spectroscopically pure as off-white solid.

Gram-Scale Synthesis of ethyl (*R*)-2-((((9H-fluoren-9-yl)methoxy)carbonyl)amino)-2-(benzofuran-2-yl)acetate (1x)

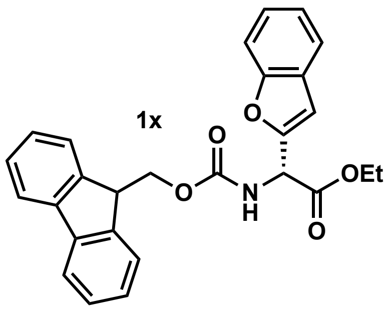

N,*O*-acetal **2c** (1.0 g, 2.60 mmol, 1.0 equiv) was given to a flame-dried 100 mL Schlenk tube under an atmosphere of argon followed by IDPi-catalyst **4f** (70 mg, 26.1 µmol, 1 mol%). The tube was cooled to –78 °C and CyMe (27 mL) was added followed by the dropwise addition of benzofuran (**3x**, 0.57 mL, 5.22 mmol, 2.0 equiv.). The heterogeneous mixture was allowed to warm to room temperature and then stirred at 30 °C for 5 d whereupon complete conversion of the *N,O*-acetal **2c** was observed. The reaction was quenched with triethylamine (30 µL), adsorbed on celite® and purified via flash column chromatography on silica gel (eluent: *i*-hexanes/MTBE 5:1 to 3:1) to yield the desired arylglycine **1X** (1.06 g, 2.40 mmol, 92%) as white solid with an enantiomeric ratio of 94.5:5.5 and a regioisomeric ratio of 86:14. The compound **1x** can be recrystallized from *i*-hexanes and CH_2Cl_2 to yield arylglycine **1x** (0.98 g, 2.22 mmol, 85%) as white solid with an increased enantiomeric ratio of 98:2 and a regioisomeric ratio of 91:9 after a single recrystallization.

*For the analytical data of **1x** isolated herein, see the corresponding entry at section 5.2 of this supporting information. LC-data of the recrystallized compound as to be found below:*

2D-LC: (1. dimension: 100 mm Zorbax RX-Sil, *n*-heptane/*i*-PrOH 99.5:0.5, 308 K, 220 nm): t_R (major regioisomer) = 3.1 min, t_R (minor regioisomer) = 3.3 min, r.r. = 91:9.
(2. dimension: Chiralpak IB-3, *n*-heptane/*i*-PrOH 80:20, 298 K, 220 nm): t_R (minor enantiomer) = 5.9 min, t_R (major enantiomer) = 6.9 min, e.r. = 97.5:2.5 (95% e.e.).

To reisolate IDPi catalyst **4f**, the silica gel column was flushed (eluent: CH_2Cl_2/EtOAc 99:1) after complete elution of arylglycine **1q**. The obtained solution was concentrated and the residue was purified via flash column chromatography on silica gel (eluent: *i*-hexanes/CH_2Cl_2 1:1 ⟶ CH_2Cl_2/EtOAc 99.5:0.5) to yield catalyst **4f** as salt. After acidification over DOWEX 50WX8 (H-form, eluted with CH_2Cl_2), the reisolated catalyst **4f** (31 mg, 11.5 µmol, 44%) was obtained spectroscopically pure as off-white solid.

7. Determination of the Absolute Configuration of Arylglycine Products

The absolute configuration of the produced arylglycine products was determined via comparison with an enantiopure sample as described below: commercially available enantiopure *D*-(–)-phenylglycin-ethylester hydrochloride (**S10**, purchased from https://www.sigmaaldrich.com/DE/de, CAS registry number = 17609-48-2) was transformed to the *N*-Fmoc protected amino acid ester **1j** (**figure SI-3**). HPLC-analysis and subsequent comparison with arylglycine **1j** prepared following General Procedure D using (S,S)-IDPi **4h** shows formation of the (*R*)-arylglycine **1j** as predominant enantiomer.

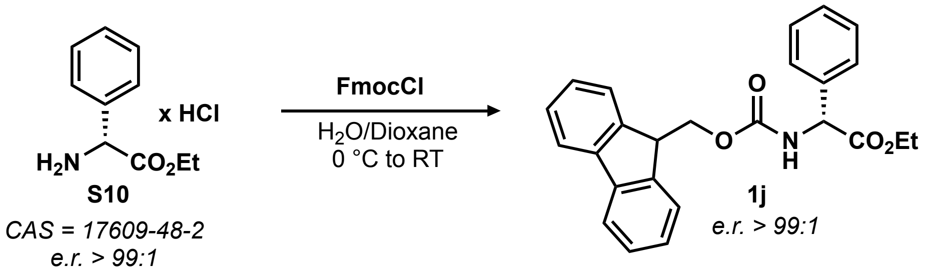

Figure SI-3: Synthesis of phenylglycine **1j** from commercial enantiopure amino acid **S9**.

ethyl (R)-2-((((9H-fluoren-9-yl)oxy)carbonyl)amino)-2-phenylacetate (1j)

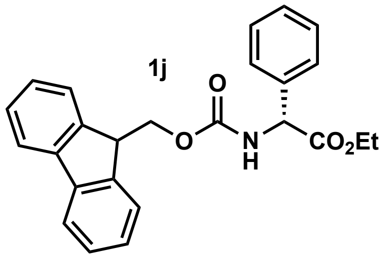

Following a procedure reported by Hulme, ethyl (*R*)-2-amino-2-phenylacetate hydrochloride (**S10**, e.r. > 99:1, 500 mg, 2.31 mmol, 1.0 equiv.) was given to a round bottom flask, dissolved in dioxane (7 mL) and a 15% aqueous solution of Na_2CO_3 (7 mL) was added. The mixture was cooled to 0 °C and stirred for 10 min, then 9-fluorenylmethoxycarbonylchlorid (600 mg, 2.31 mmol, 1.0 equiv.) was added in one portion, the mixture was allowed to warm to room temperature and stirred for 18 h. Water (20 mL) and CH_2Cl_2 (20 mL) was added, the layers were separated and the organic layer was washed with water (3 x 20 mL) and brine (20 mL) and then dried (Na_2SO_4). The solution was concentrated under reduced pressure to yield the desired carbamate (915 mg, 2.30 mmol, 98%) as white solid which was used as reference without further purification.

For the analytical data of ***1j*** *isolated herein, see the corresponding entry at section 5.1 of this supporting information. LC-data of compound* ***1j*** *obtained as described above to be found below:*

HPLC: (Chiralpak IB-3 column, *n*-heptane/*i*-PrOH 95:5, 298 K, 254 nm): t_R (minor) = 13.3 min, t_R (major) = 19.2 min, e.r. > 99.5:0.5 (>99% e.e.).

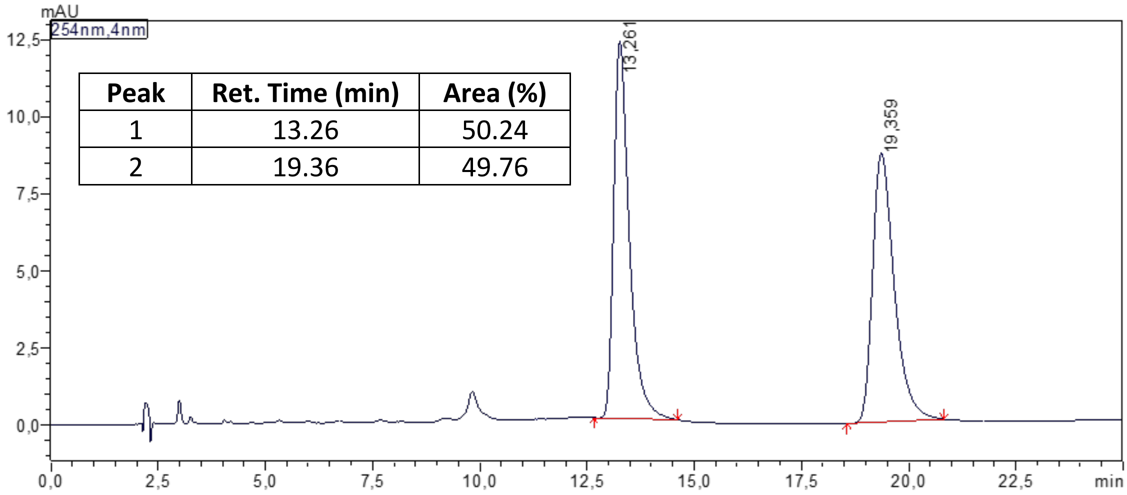

Peak	Ret. Time (min)	Area (%)
1	13.26	50.24
2	19.36	49.76

Figure SI-4: HPLC-traces of racemic **1j**, obtained using $HNTf_2$ as achiral catalyst.

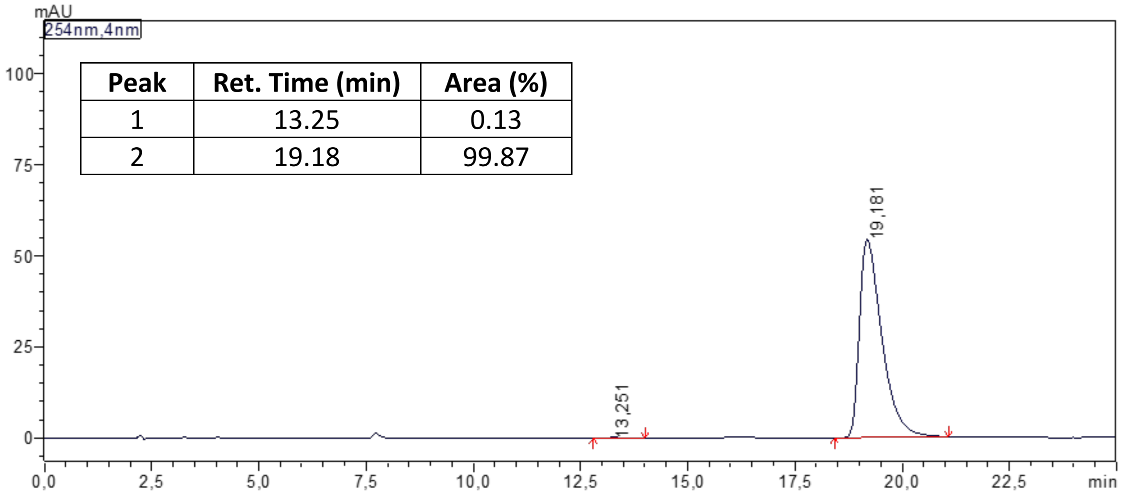

Peak	Ret. Time (min)	Area (%)
1	13.25	0.13
2	19.18	99.87

Figure SI-5: HPLC-traces of enantioenriched (*R*)-**1j**, obtained from commercial enantiopure **S9**.

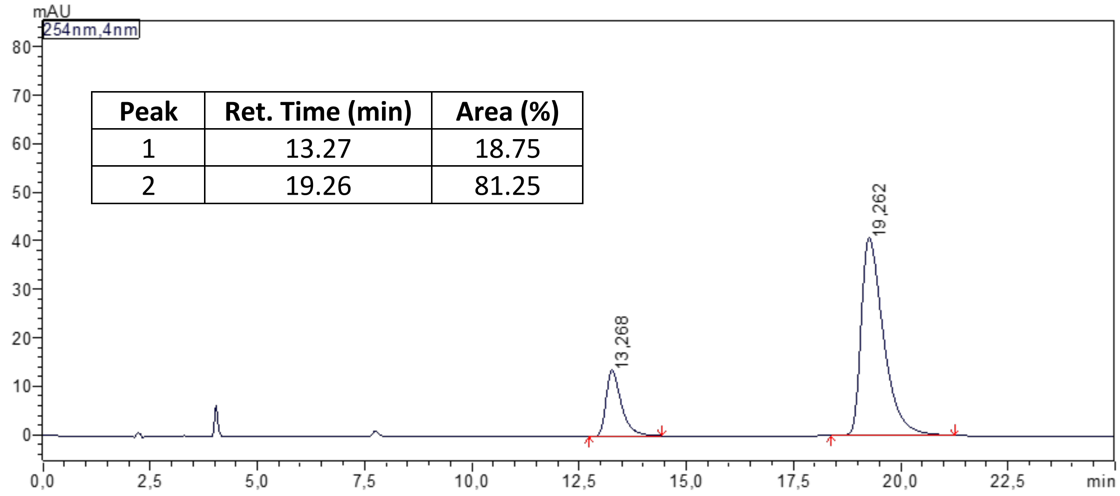

Peak	Ret. Time (min)	Area (%)
1	13.27	18.75
2	19.26	81.25

Figure SI-6: HPLC-traces of enantioenriched **1j**, obtained using **4h** as chiral catalyst.

8. Mechanistic Investigations

8.1 Reactivity Assessment via Acetate Scrambling Experiments

During initial reaction optimization studies, we noticed that only a small choice of highly acidic (*S,S*)-IDPi catalysts **4** are able to promote C–C bond formation in the Friedel–Crafts reaction between *N,O*-acetals **2** and toluene (**3a**). To gain insights in the limiting factors that prevent product formation using less acidic catalysts, we were interested in the development of tools that enable the monitoring of the interaction between the used *N,O*-acetals **2** and (*S,S*)-IDPi catalysts **4**. Based on earlier studies, we expected iminium ion formation upon reaction of **2** with acid catalysts **4** followed by ion pair **I** formation with the corresponding anion of **4** to be a possible reaction pathway.[13] To investigate the latter, we designed an "acetate scrambling" experiment (exchange of acetate-h_3 and acetate-d_3 on *N*,*O*-acetal **2c** as described in **figure SI-7**) promoted by Brønsted acid catalysts adding deuterated acetic acid as mechanistic probe:

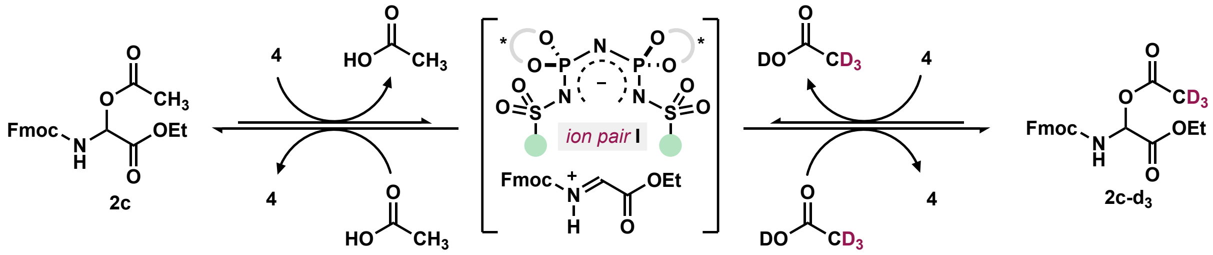

Figure SI-7: Design and mechanistic concept of the acetate scrambling experiments.

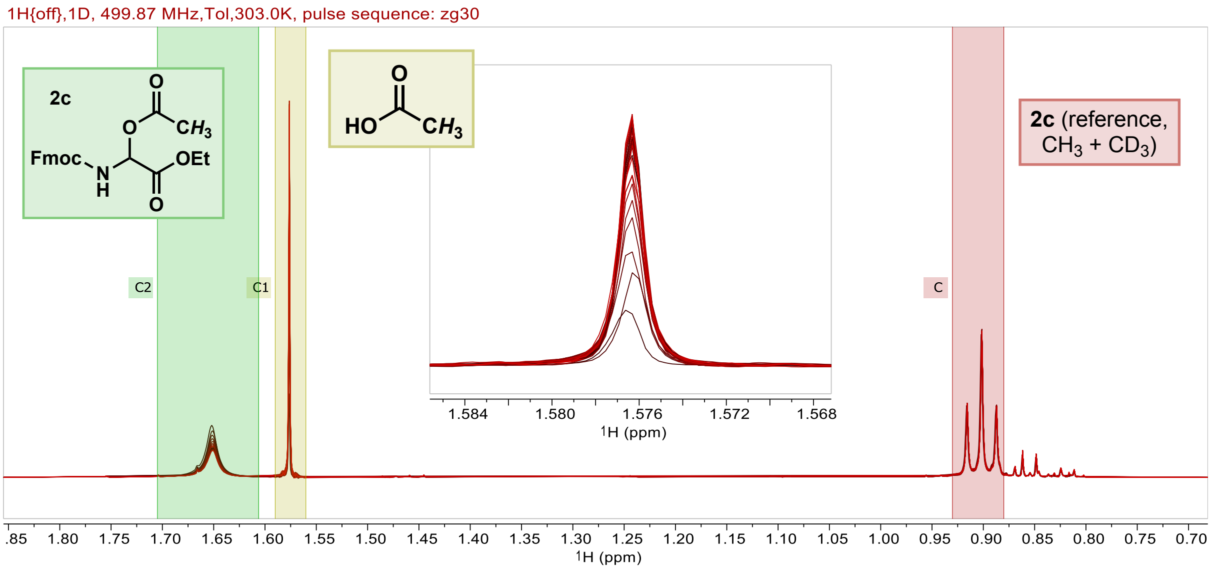

Figure SI-8: Quantification of the acetate scrambling experiment via ^{1}H-NMR analysis (exemplified spectroscopic data collected for the reaction with catalyst **4b**).

The respective catalyst (2.5 µmol, 5 mol%) and *N,O*-acetal **2c** (19.2 mg, 50 µmol, 1.0 equiv.) were given to an oven dried NMR tube, the tube was placed under an atmosphere of argon and a solution of acetic acid-d_4 in PhMe-d_8 (0.071 M solution, 0.7 mL, 50 µmol acetic acid-d_4, 1.0 equiv.) was added. The tube was shaken and the homogeneous mixture was directly monitored via ^{1}H-NMR analysis. The

equilibration between **2c**-h_3 and **2c**-d_3 was observed through the formation of free acetic acid-h_3 (signal at ca 1.58 ppm) and the decrease of the signal corresponding to the acetate group of **2c**-h_3 (signal at ca 1.65 ppm) using the signal of the methyl group of the ethyl ester terminus as relatively constant reference (signal at ca 0.9 ppm, sum of **2c**-h_3 and **2c**-d_3).

General Remark for the Evaluation of Kinetic NMR Experiments: Kinetic ^{1}H-NMR experiments were generally acquired with single scans (30° pulses) until an appropriate conversion was reached. The data was then imported with the Reaction Monitoring Plugin into MNOVA 14.3.2 and processed therein (phase correction, baseline correction, integration). Unless noted otherwise, the first NMR spectrum of each kinetic series was used as absolute concentration reference using total CH3COOX integral (AcOH + AcOX) as reference signal (= 100 mol%). This integral was found to be constant for all reactions performed. For the reaction with additional AcOH, the acetate signal of the starting material was used as reference signal (= 100 mol%). As this point no significant conversion was observed.

The observed rates are shown in the diagrams below:

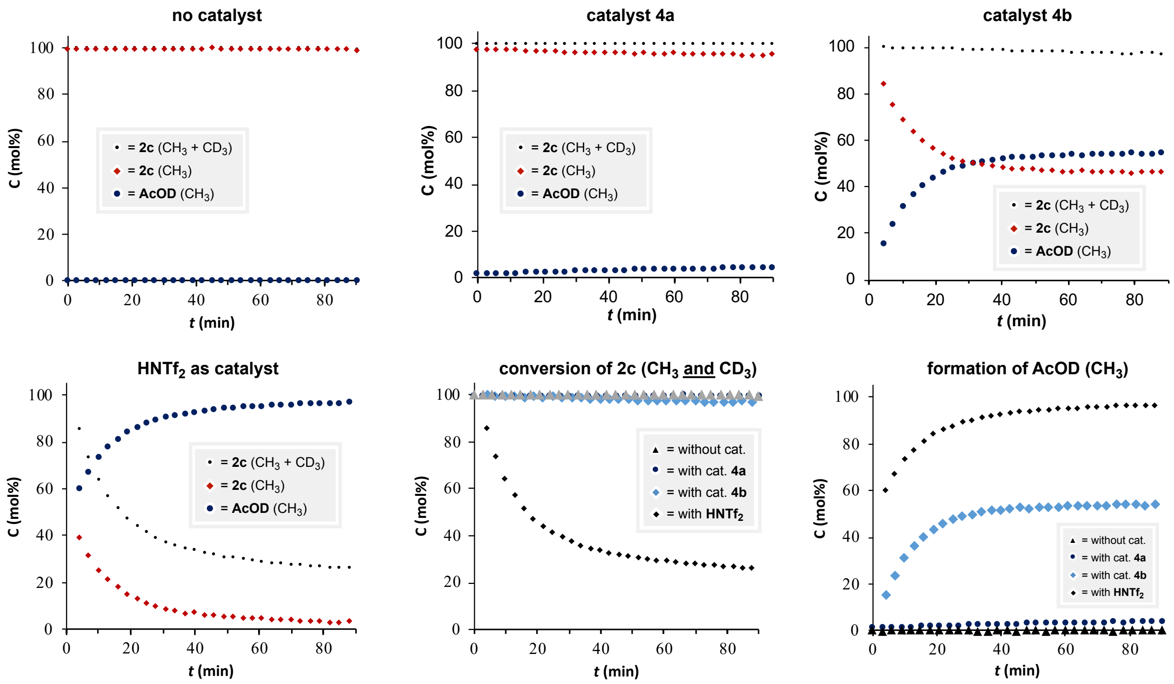

Figure SI-9: Observed rates of equilibration in the acetate scrambling experiments.

Without the presence of an acid catalyst, no acetate scrambling can be observed in the chosen timescale (left diagram upper row). Similarly, using catalyst **4a** (inactive in the Friedel–Crafts reaction towards **1a**), only extremely slow exchange of acetate-h_3 with acetate-d_3 in **2c** can be found (middle diagram upper row). When catalyst **4b** (active in the Friedel–Crafts reaction towards **1a**) however is used, complete equilibration between **2c**-h_3 and **2c**-d_3 is observed within 40 minutes (right diagram upper row). This significant difference in the rate of equilibration observed for **4b** in comparison with **4a** is in agreement with the respective catalyst's activity (or inactivity) in the Friedel–Crafts reaction between **2c** and the arenes **3** investigated herein.

Based on these experiments as well as previous investigations using reactive iminium ion intermediates, we propose the formation of ion pair **I** from **2c** and **4b** as crucial intermediate for the subsequent Friedel–Crafts reaction.[13] The less acidic catalyst **4a** however is not able to sufficiently

activate *N,O*-acetal **2c** towards the formation of ion pair **I** (or to sufficiently stabilize ion pair **I** and to therefore prolonging its lifetime so that the subsequent nucleophilic attack of arene substrates **3** is possible).

Intriguingly, using $HNTf_2$ as catalyst, complete equilibration between **2c**-h_3 and **2c**-d_3 can be observed before the first NMR measurement was performed (left diagram bottom row). Furthermore, consumption of **2c** towards arylglycin **1a** can be observed in the investigated timescale, leading to additional release of AcOD-h_3 (see middle and right diagram bottom row).

8.2 *Investigation of Inhibitory Effects of Acetic Acid*

As described in section 8.1 of this SI, we propose the formation of ion pair **I** from *N,O*-acetal **2c** and (*S,S*)-IDPi catalysts **4** in an equilibrium state to be a crucial intermediate towards the subsequent Friedel–Crafts type formation of arylglycine products **1**. Consequentially, the concentration of free acetic acid in the system, released from the leaving group of converted *N,O*-acetal **2c**, should increase with the proceeding of the reaction, shifting the equilibrium away from ion pair **I** and therefore slowing down the reaction or even inhibiting it completely at higher levels of conversion (**figure SI-10**).

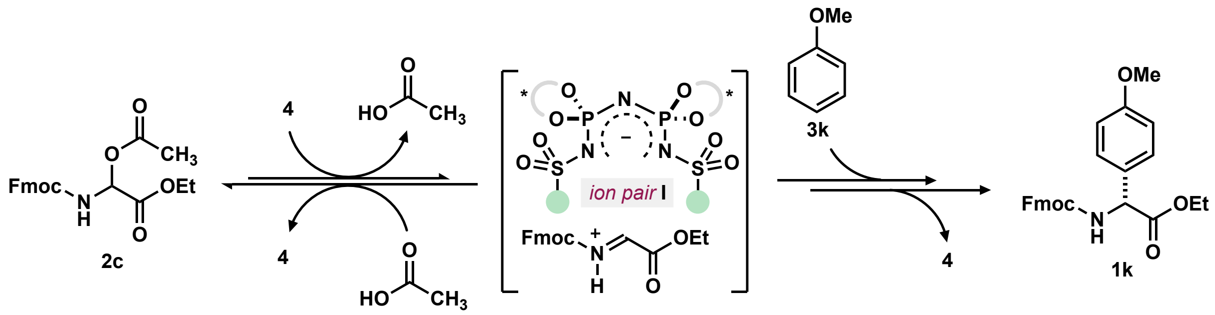

Figure SI-10: Ion pair **I** formation towards the formation of arylglycine **1k**.

To further investigate the inhibitory effects of acetic acid in the Friedel–Crafts reaction reported herein, the rates of reaction of **2c** with anisole (**3k**) as mechanistic probe towards arylglycine **1k** using (*S,S*)-IDPi **4b** were measured with an optional addition of acetic acid:

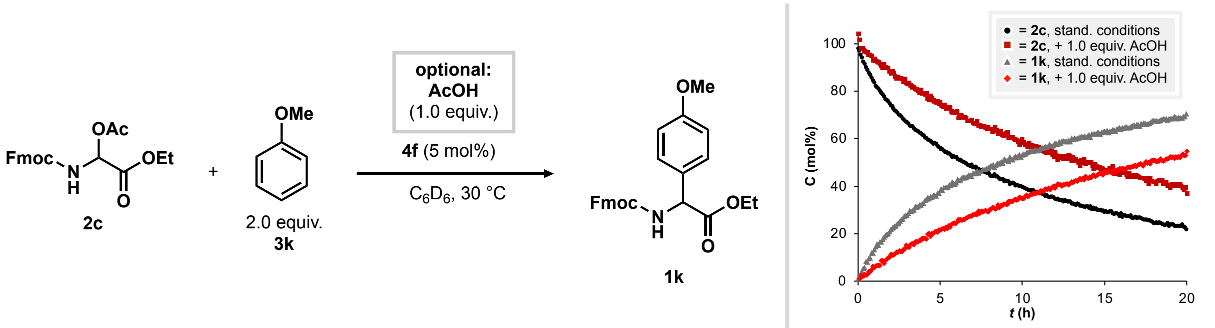

Figure SI-11: Concept and measured rates for the acetic acid inhibition experiment.

(*S,S*)-IDPi **4f** (6.7 mg, 2.5 µmol, 5 mol%) and *N,O*-acetal **2c** (19.2 mg, 50 µmol, 1.0 equiv.) were given to an oven dried NMR tube, the tube was placed under an atmosphere of argon and a stock solution of anisole (**3k**) in C_6D_6 (0.143 M solution, 0.7 mL, 0.1 mmol of **3k**, 2.0 equiv.) or a stock solution of anisole (**3k**) and acetic acid (0.143 M solution regarding **3k**, 0.0712 M regarding acetic acid, 0.7 mL, 0.1 mmol of **3k**, 2.0 equiv. of **3k**; 0.05 mmol of acetic acid, 1.0 equiv. of acetic acid) was added. The tube was shaken and the homogeneous mixture was directly monitored via ^{1}H-NMR analysis. The conversion of starting material **2c** was monitored through the decrease of **2c**'s acetate signals (signals at 1.56 ppm) and the formation of the Friedel–Crafts product **1k** was monitored through the product **1k**'s Ar–O–C*H*$_3$ signal (signals at 3.17 ppm, **figure SI-12**).

General Remark for the Evaluation of Kinetic NMR Experiments: Kinetic ^{1}H-NMR experiments were generally acquired with single scans (30° pulses) until an appropriate conversion was reached. The data was then imported with the Reaction Monitoring Plugin into MNOVA 14.3.2 and processed therein (phase correction, baseline correction, integration). Unless noted otherwise, the first NMR spectrum of each kinetic series was used as absolute concentration reference using total CH3COOX integral

(AcOH + AcOX) as reference signal (= 100 mol%). This integral was found to be constant for all reactions performed. For the reaction with additional AcOH, the acetate signal of the starting material was used as reference signal (= 100 mol%). As this point no significant conversion was observed.

As displayed above (**figure SI-11**), a significantly slower rate of product **1k** formation can be observed upon addition of 1.0 equivalent of acetic acid compared to the reference reaction without additional acetic acid. This observation supports the suggested inhibitory properties of acetic acid for the formation of product **1k** under the described reaction conditions.

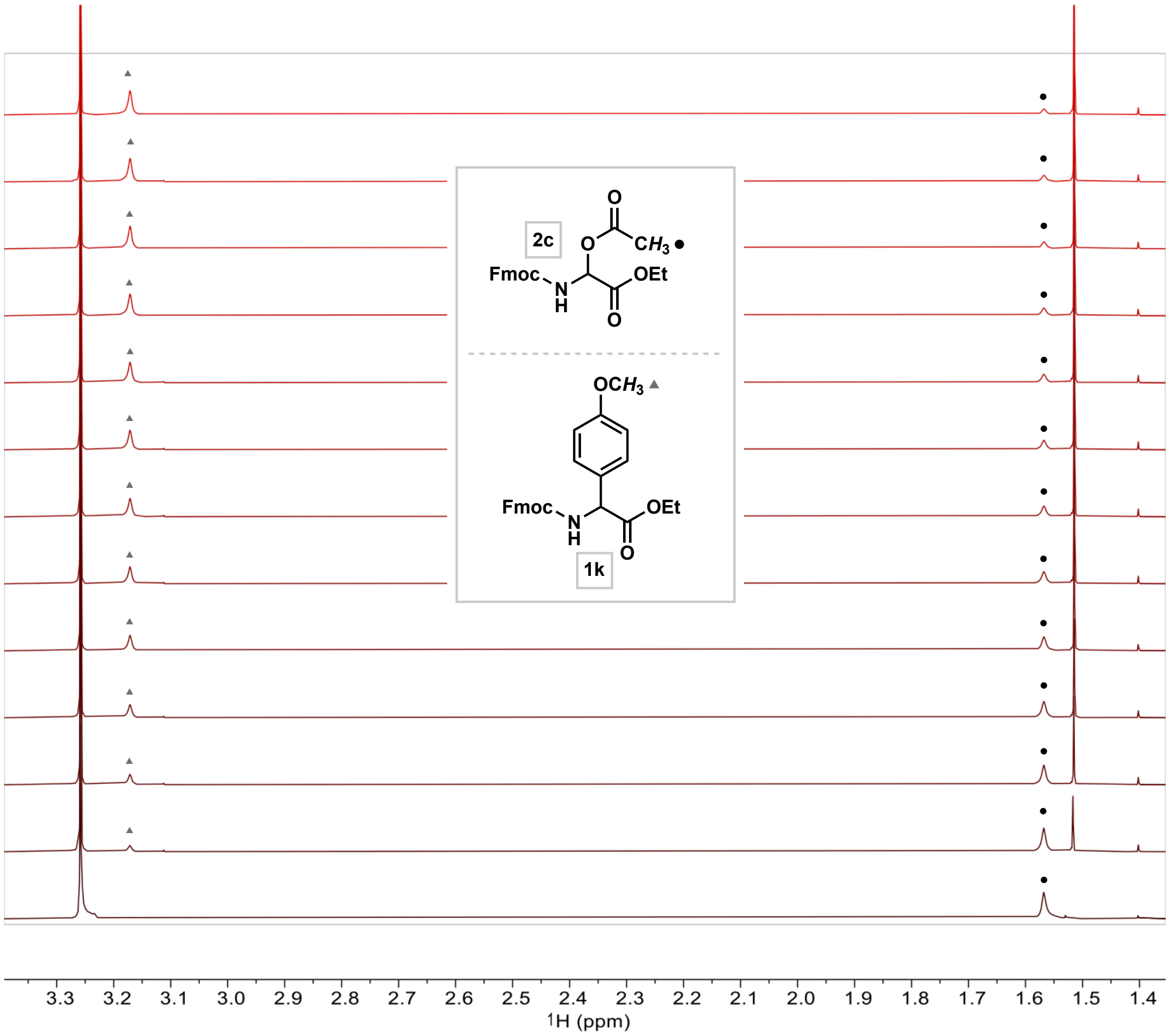

Figure SI-12: Determination of reaction rates via ^{1}H-NMR (exemplified in the reaction without AcOH).

To investigate the effect of additional acetic acid on the enantioselectivities of products **1**, the following experiments have been carried out:

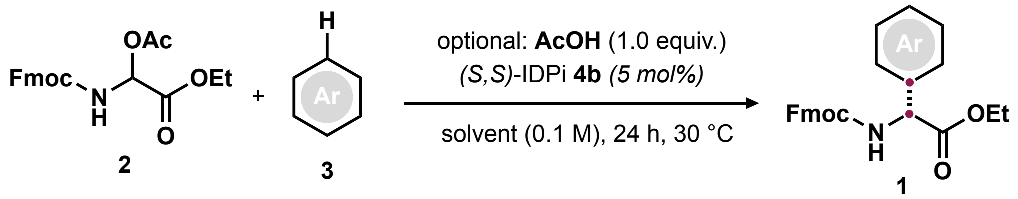

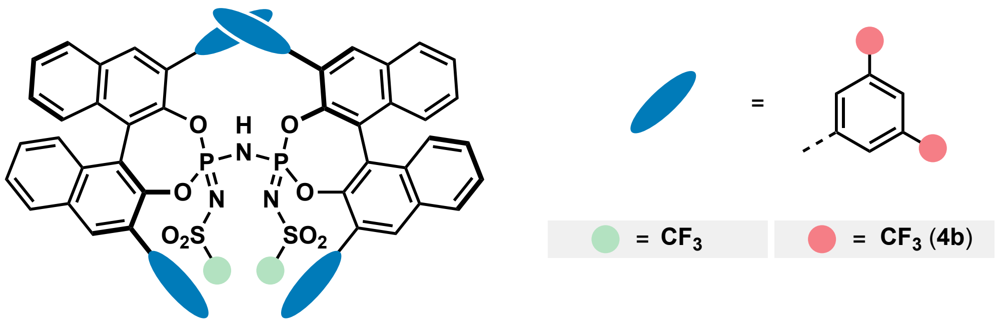

entry	arene	solvent	AcOH	yield (%)	e.r.
1	PhMe (**3a**)	neat	–	55	90:10
2	PhMe (**3a**)	neat	1.0 equiv.	35	90:10
3	anisole (**3k**)	CyMe	–	67	87:13
4	anisole (**3k**)	CyMe	1.0 equiv.	63	88:12

Figure SI-13: Effect of additional AcOH on the enantiomeric ratios of products **1**.

An oven dried 1 mL screwcap vial equipped with a magnetic stirring bar was charged with the catalyst (5 mol%) and *N*,*O*-acetal **2c** (9.6 mg, 1.0 equiv., 0.025 mmol). The vial was evacuated under high vacuum and subsequently placed under an atmosphere of argon. Solvent (0.3 mL) was added quickly followed by anisole (*optional for entries 3 and 4*, 5.4 µL, 2.0 equiv., 0.05 mmol), the vial was sealed and the mixture was stirred at 30 °C for 24 h. The reaction was then quenched via addition of triethylamine (0.3 M solution in PhMe, 50 µL), dimethylsulfone (2.0 M solution in MeCN, 12.5 µL, 1.0 equiv.) was added as internal standard and the mixture was diluted with $CHCl_3$ (0.2 mL). An aliquot of the reaction was diluted with $CDCl_3$ (0.6 mL) and analyzed via ^{1}H-NMR to determine the reaction yield. The remaining mixture was purified via preparative thin layer chromatography (PTLC) for the subsequent HPLC analysis for the determination of the enantiomeric ratio.

For toluene (**3a**), addition of AcOH significantly slows down the reaction which leads to visibly lower yields for product **1a** (entries 1 and 2). The enantiomeric ratio of the formed product **1a** however was found to be identical for both reactions.

For the reaction of anisole (**3k**), the yield on product **1k** is very similar for both reactions (entries 3 and 4, with or without additional AcOH), which is most likely due to the increased nucleophilicity of **3k**. The enantiomeric ratio was found to be very similar for both reactions with a slightly increased enantiomeric ratio for the reaction with additional AcOH.

As described above, the influence of additional AcOH on the observed enantiomeric ratio is only minimal. On the basis of these experiments we therefore conclude that a potential kinetic resolution of starting material **2c** does not play a relevant role in the process investigated herein.

8.3 *Kinetic Isotope Effect Studies*

As a mechanistic probe towards the determination of the rate limiting step of the Friedel–Crafts reaction between *N,O*-acetal **2c** and toluene (**3a**), direct competition kinetic isotope effect (KIE) studies were performed as described below (**figure SI-14**):

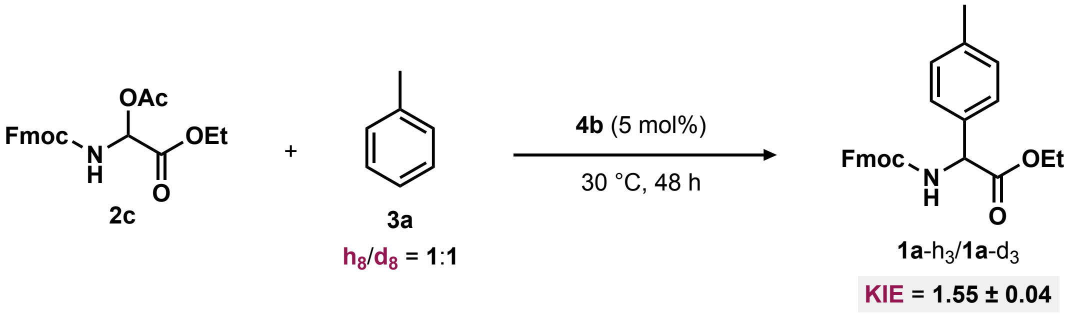

Figure SI-14: Direct competition KIE studies with a 1:1 mixture of toluene-h_8 and toluene-d_8.

(*S,S*)-IDPi **4b** (8.9 mg, 5 µmol, 5 mol%) was given to a flame dried Schlenk tube under an atmosphere of argon followed by *N,O*-acetal **2c** (38.3 mg, 0.1 mmol, 1.0 equiv.). 1 mL of a 1:1 (*v/v*) mixture of toluene-h_8 and toluene-d_8 (premixed, equals 4.694 mmol toluene-h_8 and 4.706 mmol toluene-d_8, ratio PhMe-h_8/PhMe-d_8 = 0.998:1) was added and the mixture was stirred at 30 °C for 48 h. The reaction was then stopped via the addition of NEt_3 (stock solution, 0.3 M in CH_2Cl_2, 200 µL) and applied directly on a silica gel column equilibrated with *i*-hexanes. The mixture was flushed with *i*-hexanes (100 mL) and then purified via flash column chromatography on silica gel (*i*-hexanes/MTBE 4:1) to yield the mixture of arylglycine **1a**-h_7 and **1a**-d_7. The experiment was performed twice, and the average of both experiments was used for the determination of the KIE. The KIE was determined via NMR analysis (ratio of **1a**-h_3 to **1a**-d_3) of the isolated product **1a** as described below:

With regards to the high excess of toluene in the system (**3a**-h_8 as well as **3a**-d_8), changes in concentration of arene **3a** were neglected for the determination of the KIE at hand. The average of three different signal sets was used, taking into account both rotameric species if present:

1.) Ratio of *H* at C6 (**1a**-h_7 + **1a**-d_7, 5.25 ppm and 5.10 ppm) to C*H*$_3$ at C5 (**1a**-h_7 only, 2.31 ppm and 2.35 ppm), determined via ^{1}H-NMR.
2.) Ratio of C6 (C_α) in **1a**-h_7 (57.5 ppm and 57.9 ppm) to C6 (C_α) in **1a**-d_7 (57.4 ppm and 57.8 ppm) via 13C-NMR (integration of the two separate peaks).
3.) Ratio of C1 in **1a**-h_7 (C_{ipso}, 170.82 ppm) to C1 in **1a**-d_7 (C_{ipso}, 170.85 ppm) via 13C-NMR (integration of the two separate peaks).

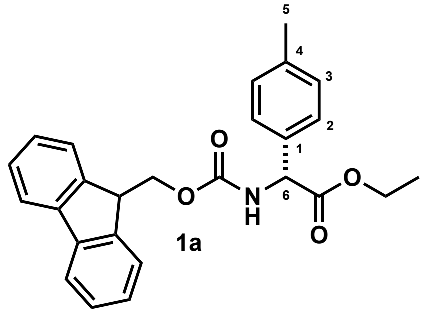

The corresponding spectra are shown below (**figures SI-15-18**).

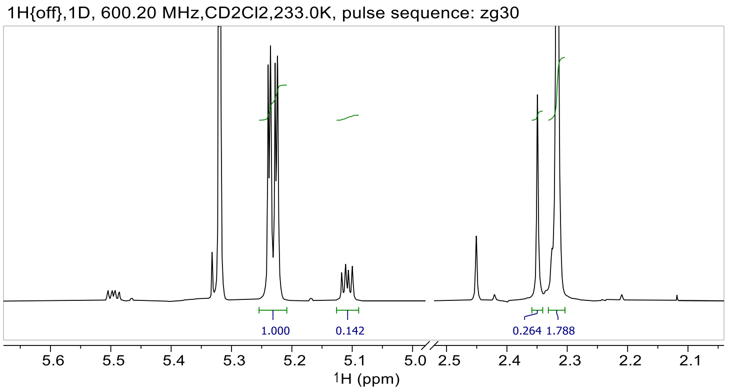

	^{1}H		
	Int (C_a)	Ar-Me	KIE
major	1	1.79	1.48
minor	0.14	0.26	1.63

	^{13}C		
	Int(H-7)	Int (D7)	KIE
$C_{a, major}$	0.48	0.29	1.66
$C_{a, minor}$	3.73	2.33	1.60
$C_{ipso, major}$	1.64	1.00	1.64

KIE (average)	1.60 ± 0.07

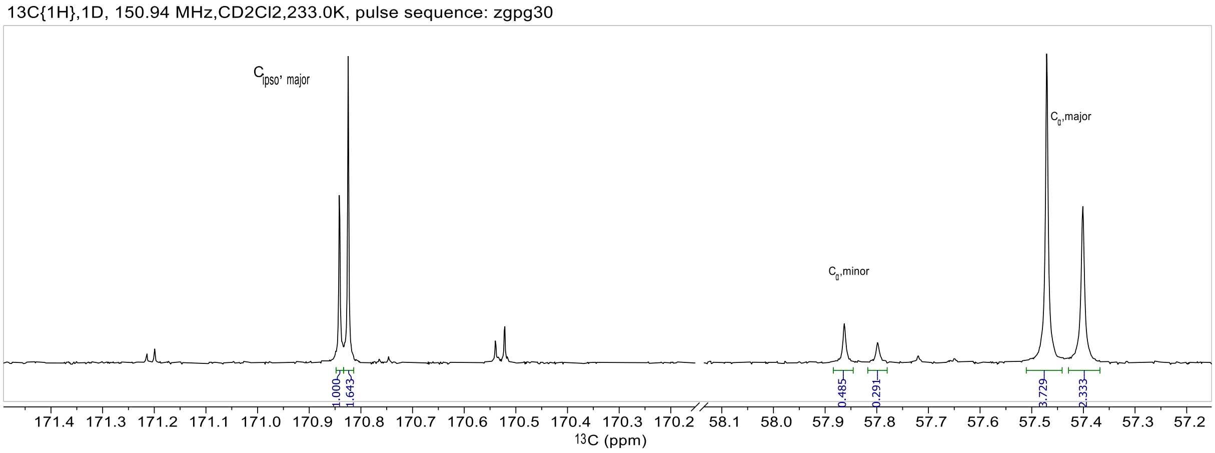

Figure SI-15: Experiment 1 for the determination of the KIE.

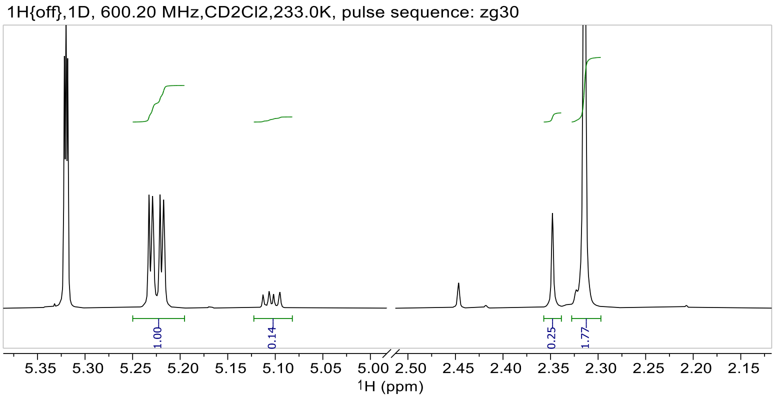

	^{1}H		
	Int (C_a)	Ar-Me	KIE
major	1	1.77	1.44
minor	0.14	0.25	1.47

	^{13}C		
	Int(H-7)	Int (D7)	KIE
$C_{a, major}$	0.52	0.34	1.53
$C_{a, minor}$	4.16	2.75	1.51
$C_{ipso, major}$	1.52	1.00	1.52

KIE (average)	1.49 ± 0.04

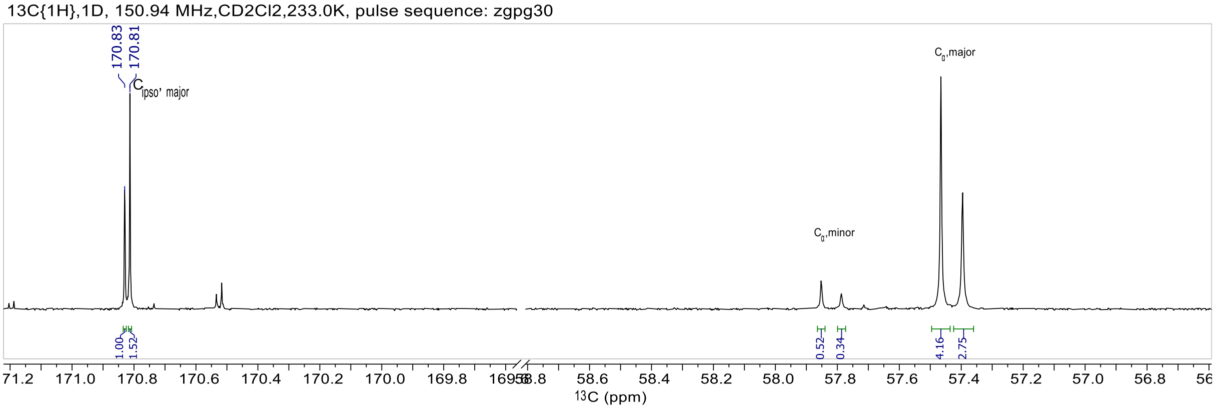

Figure SI-16: Experiment 2 for the determination of the KIE.

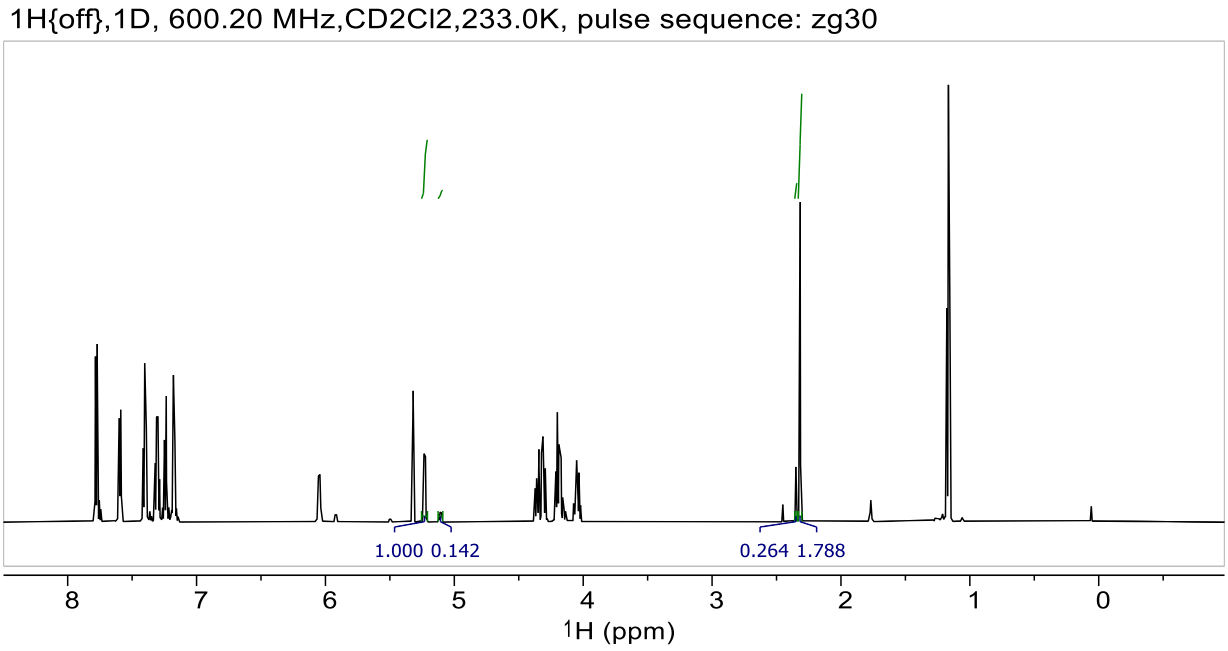

Figure SI-17: Full ^{1}H-NMR spectrum (exemplarily for experiment 1) for the determination of the KIE.

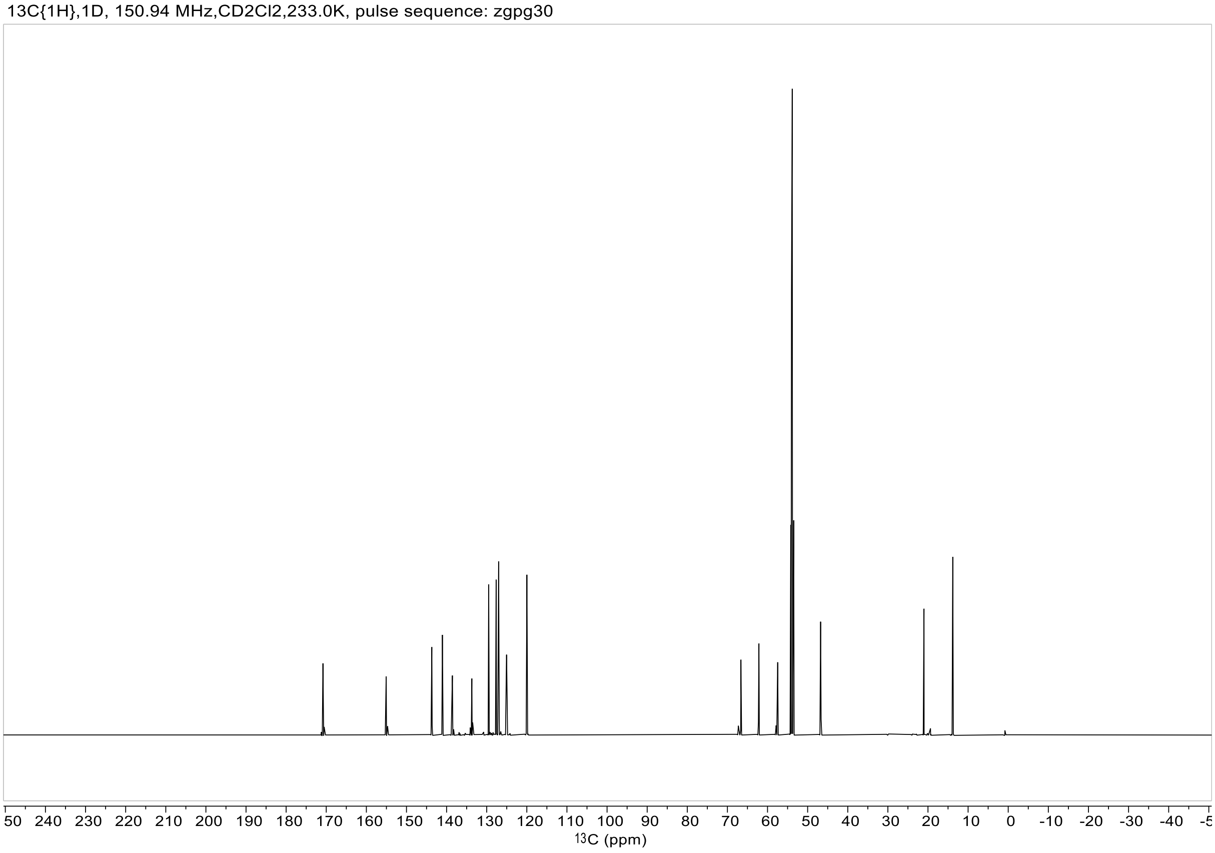

Figure SI-18: Full ^{13}C-NMR spectrum (exemplarily for experiment 1) for the determination of the KIE.

Based on the above measurements (**figures SI-15** and **SI-16**), the KIE was determined as average of both experiments:

$$\mathrm{KIE} = \frac{(\mathrm{KIE}_1 + \mathrm{KIE}_2)}{2} = 1.545$$

The corresponding standard deviation was determined as follows:

$$\sigma_{KIE} = \sqrt{\frac{{\sigma_1}^2}{2^2} + \frac{{\sigma_2}^2}{2^2}} = \sqrt{\frac{0.07^2}{2^2} + \frac{0.04^2}{2^2}} = 0.040$$

On the basis of the above calculations, a resulting KIE of **1.545 ± 0.040** could be determined.

Discussion of the Experimentally Determined KIE:

As the estimated maximum theoretical value for normal secondary KIEs is around 1.4 and typical values range from 1.1 to 1.2, the KIE observed herein is more in the range of a (small) primary KIE.[14] Primary KIEs are regularly observed in Friedel–Crafts acylation reactions (examples thereof are shown below in figure SI-19):[15–19]

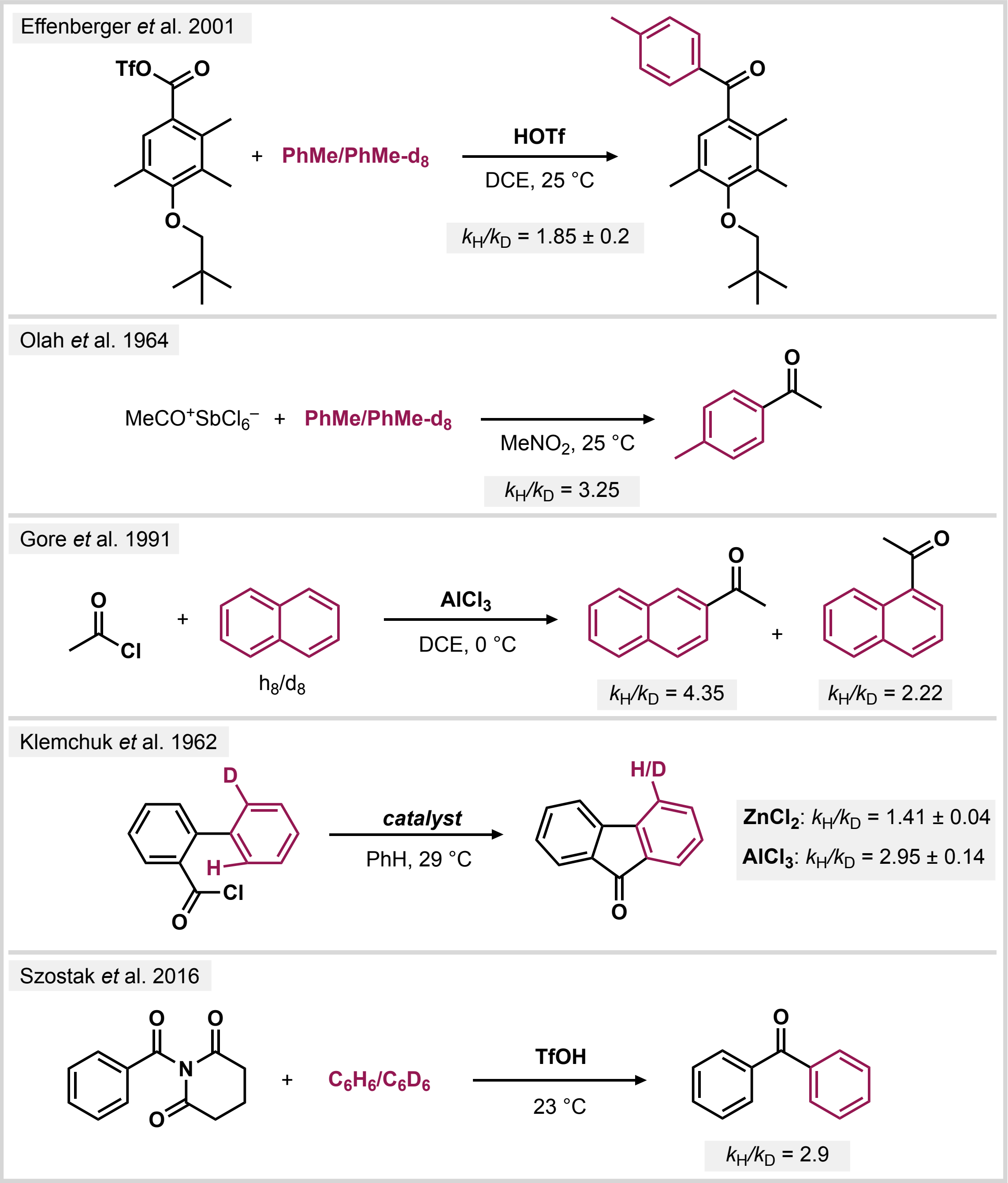

Figure SI-19: Precedents of KIE experiments in Friedel–Crafts acylations.

These primary KIEs are usually explained to originate from relatively stable σ-complexes[15] and the reversible formation thereof[18] which renders the subsequent deprotonation at least partially rate limiting.

For Friedel–Crafts alkylation reactions or nitration reactions however, secondary normal or inverse KIEs can often be observed (see figure SI-20):[20–22]

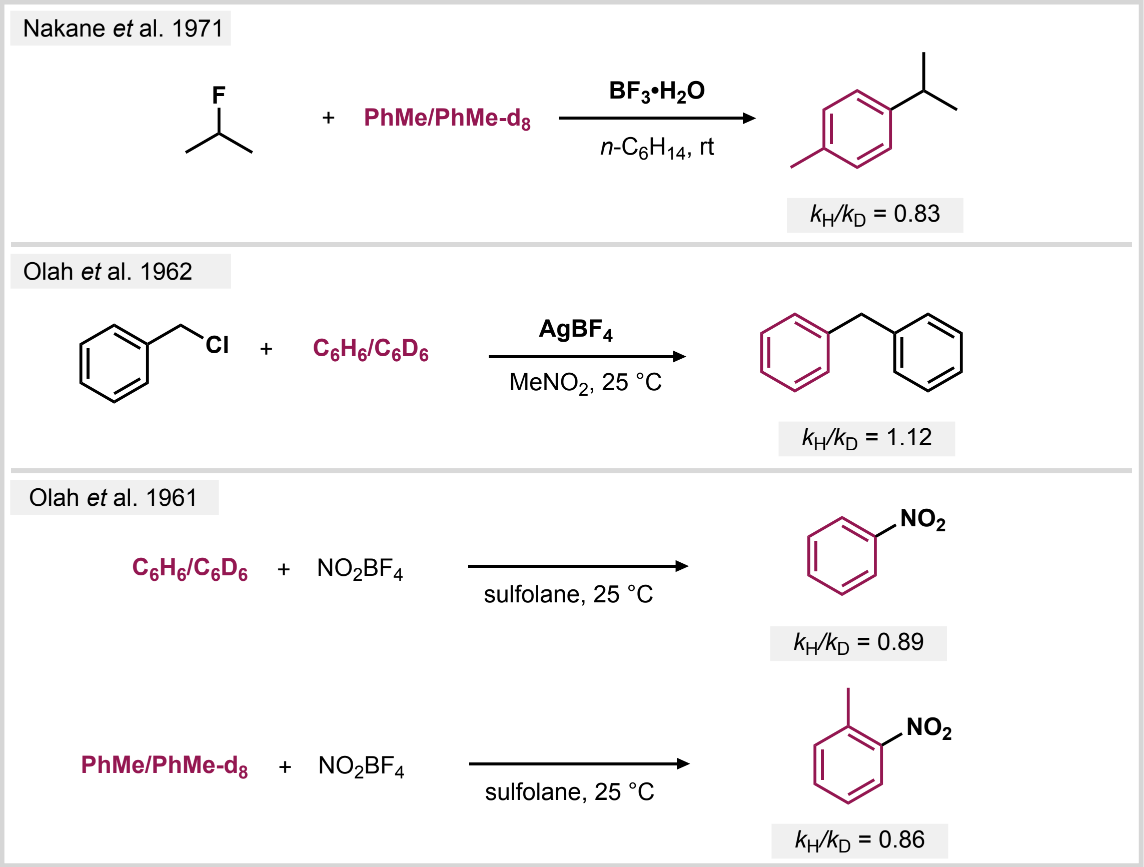

Figure SI-20: Precedents of KIE experiments in Friedel–Crafts alkylations and nitrations.

The rate limiting step in these reactions, unlike those shown in figure SI-19, is not to be identified in the rearomatization process but rather in the formation of the σ-complex which can lead to either normal or inverse KIEs.[21,22]

Based on the abovementioned precedents we interpretate the measured primary KIE of 1.545±0.04 to originate from an at least partially rate determining deprotonation of Wheland-type complex **II**. We furthermore propose an interaction with the electron rich carbamate moiety that could stabilize complex **II** as well as the transition state leading toward its formation as shown below (figure SI-21). Additional stabilization of **II** as described might furthermore contribute to the reversibility of its formation which would support the discrimination between toluene-h_8 and toluene-d_8 in the subsequent deprotonation step.

As the measured KIE is rather small for a primary KIE, we furthermore envision a preceding inverse secondary equilibrium isotope effect (EIE) in the formation of Wheland-type complex **II** from **I** which could additionally contributes to the measured KIE.[14,23]

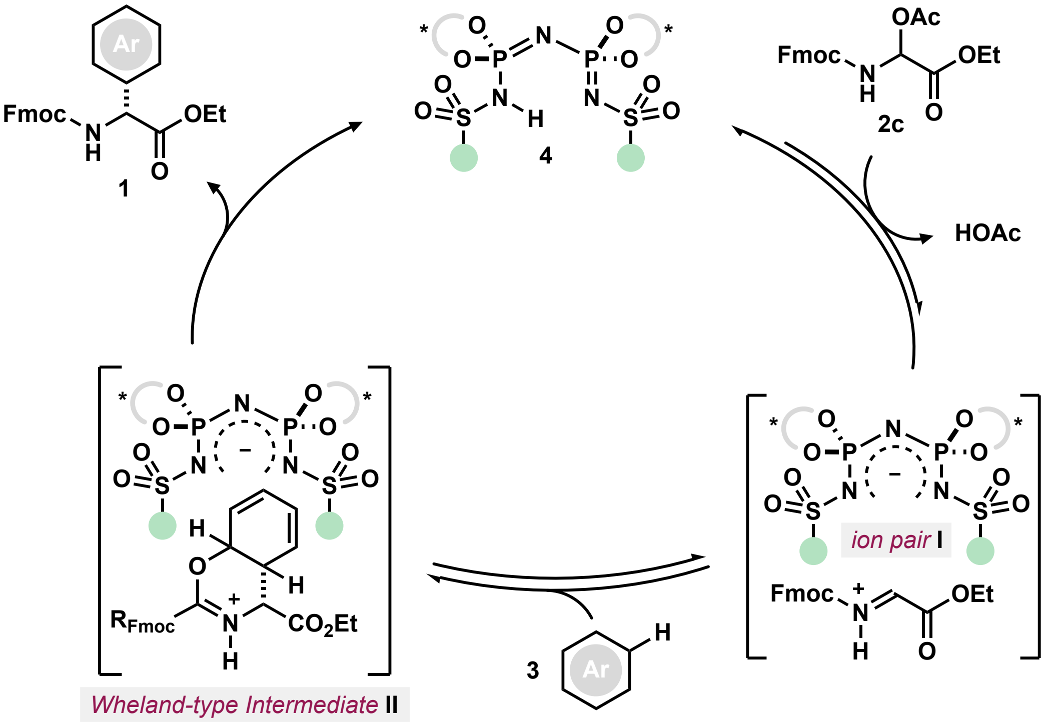

Figure SI-21: Proposed reaction mechanism for the Friedel–Crafts reaction.

9. Catalyst Stability Studies

Initial studies on the Friedel–Crafts reaction of *N,O*-acetal **2c** with toluene (**3a**) quickly revealed that the highly reactive nature of **2** (or rather its corresponding iminium ion) leads to visible hydrolysis of the (*S,S*)-IDPi's **4** phosphoramide core which results in a stepwise degradation of **4** to the corresponding imino-imidodiphosphate (*i*IDP, **S11**) and finally to the corresponding imidodiphosphate (IDP, **S12**) (**figure SI-22**).

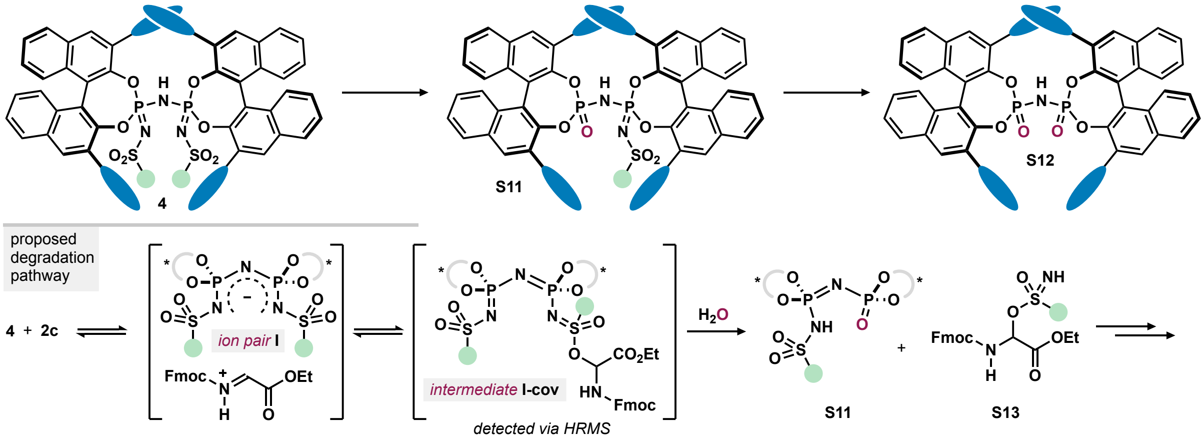

Figure SI-22: Stepwise degradation of (*S,S*)-IDPis **4**.

As *i*IDP's **S10** as well as IDP's **S11** proved to be inactive in the Friedel–Crafts reaction investigated herein (see table **S-1**), hydrolysis of the catalytically active IDPi catalysts **4** represents a problem that hinders successful C–C bond formation. To avoid this problem, we therefore optimized our (*S,S*)-IDPi **4** catalysts to not only yield the desired arylglycine products **1** with increased enantiomeric- and regioisomeric ratios, but also to be more resistant towards hydrolytic degradation which, consequentially, correlates with increased yields.

Finally, optimized catalysts **4f** and **4g**, used for the reaction scope (see **figure 2**) were found to be significantly more resilient towards hydrolytic degradation compared to benchmark catalyst **4b** as determined via ^{31}P-NMR studies (**figure SI-23**) described as follows:

<u>Evaluation of the stability of catalyst **4g** compared to benchmark catalyst **4b**:</u>

The respective catalyst (1.25 µmol, 5 mol%) and *N,O*-acetal **2c** (9.6 mg, 25 µmol, 1.0 equiv.) were given to an oven dried screwcap vial. The vial was placed under an atmosphere of argon and PhMe (0.3 mL) was added. The mixture was stirred at 30 °C for 20 h and then quenched via with NEt_3 (0.3 M solution in PhMe, 50 µL). The solution was transferred to an NMR tube and analyzed via ^{31}P-NMR to determine the relative amount of catalyst degradation towards **S11** and **S12** (see **figure SI-23**).

<u>Evaluation of the stability of catalyst **4f** compared to benchmark catalyst **4b**:</u>

The respective catalyst (1.25 µmol, 5 mol%) and *N,O*-acetal **2c** (9.6 mg, 25 µmol, 1.0 equiv.) were given to an oven dried screwcap vial. The vial was placed under an atmosphere of argon and *n*-pentane (0.3 mL) was added quickly followed by anisole (**3k**, 5.4 µL, 50 µmol, 2.0 equiv.). The mixture was stirred at 30 °C for 20 h and then quenched via with NEt_3 (0.3 M solution in PhMe, 50 µL). The solution was transferred to an NMR tube and analyzed via ^{31}P-NMR to determine the relative amount of catalyst degradation towards **S11** and **S12** (see **figure SI-23**).

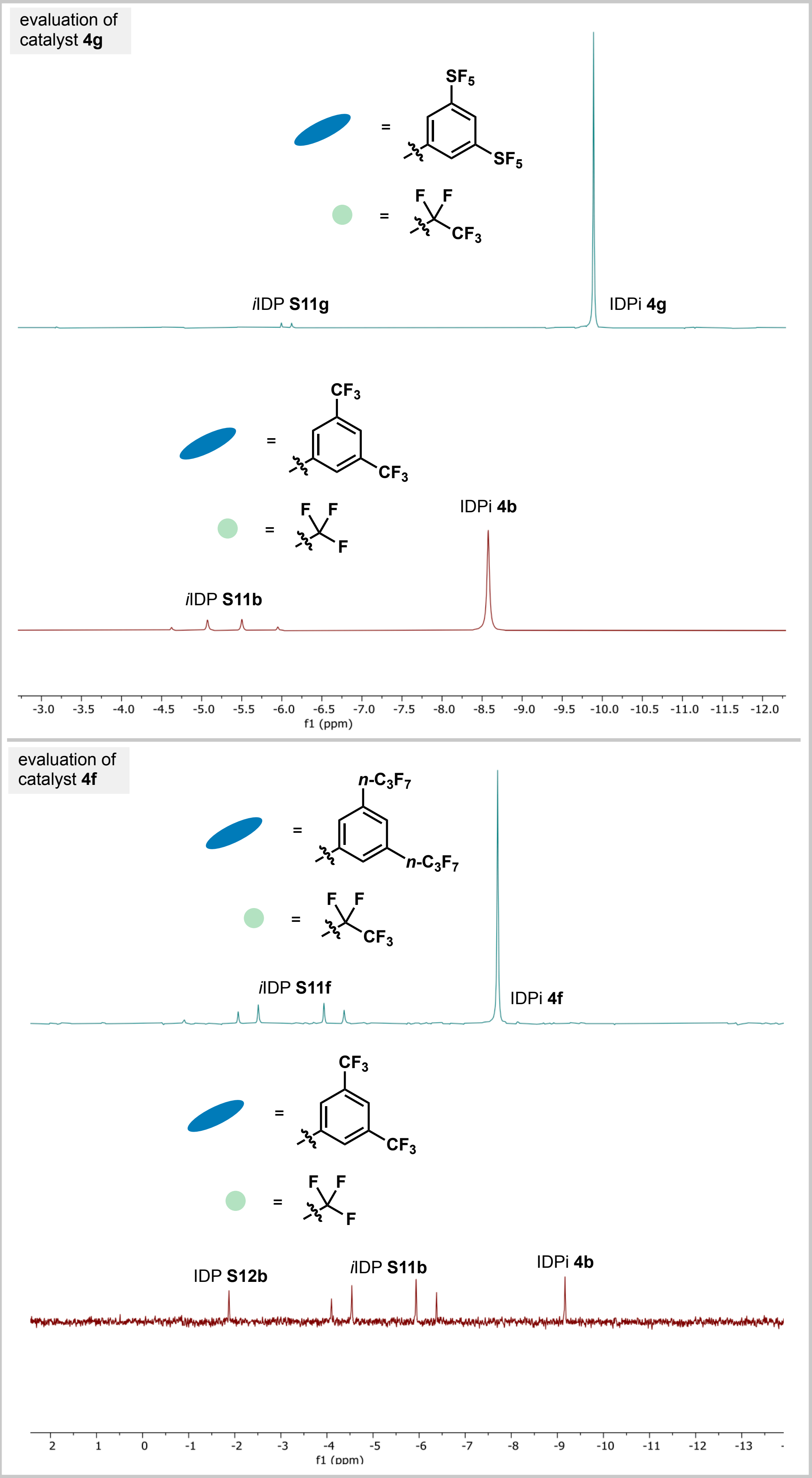

Figure SI-23: ^{31}P-NMR spectra to evaluate the stability of IDPi-catalysts **4g** and **4f**.

10. Computational Methods

Preliminary structures were generated at the GFN0-xTB[24] level using the xtb program version 6.6.0, followed by exploration of the conformational landscape at the same level of theory using Grimme's Conformer–Rotamer Ensemble Sampling Tool (CREST)[25], version 2.12. All resulting conformers were subsequently optimized at the GFN2-xTB level and, eventually, subjected to DFT geometry optimizations.[26]
All DFT calculations were conducted using ORCA version 5.0.3.[27] GFN2-xTB conformers were subjected to DFT-level geometry optimizations using the PBE exchange-correlation functional[28] and Grimme's DFT-D3 scheme[29] with Becke–Johnson damping[30] along with the def2-SVP basis set[31] (VeryTightSCF convergence criterion) in Cartesian coordinates (COPT keyword). The Resolution of Identity (RI) approximation[32] in the Split-RI-J variant[33] using a corresponding auxiliary basis set[34] was used. True ground-state structures were verified by subsequent vibrational frequency calculation at the same level of theory via absence of imaginary frequencies.
Refined electronic energies were obtained using the M06-2X exchange-correlation functional[35] in conjunction with the def2-TZVP basis set.
Gibbs free energies were calculated using the M06-2X/def2-TZVP energies in combination with thermochemical corrections from the vibrational frequency calculations acquired by using Duarte's otherm.py in a 1 M solution standard state at 303 K.[36,37]

Following this protocol, at total number of 41 ion pair conformers were evaluated at the M06-2X/def2-TZVP | PBE-D3/def2-SVP level of theory. The conformer with the lowest Gibbs free energy was used for discussion.

10.1 Calculated Structure of Iminium Ion Pair I

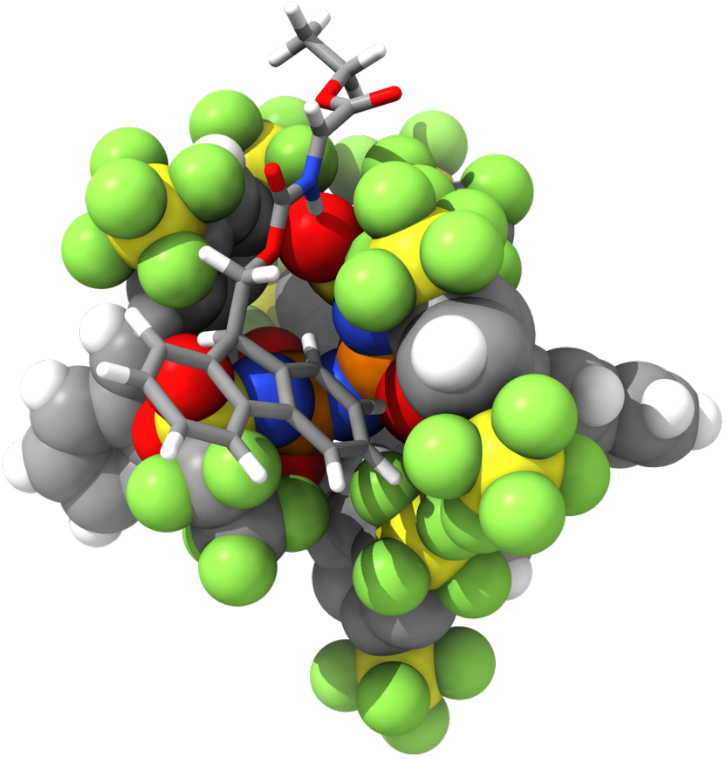

Figure SI-24: Calculated structure of iminium ion pair **I** (white = hydrogen; grey = carbon; red = oxygen; blue = nitrogen; yellow = sulfur; green = fluorine; orange = phosphorus).

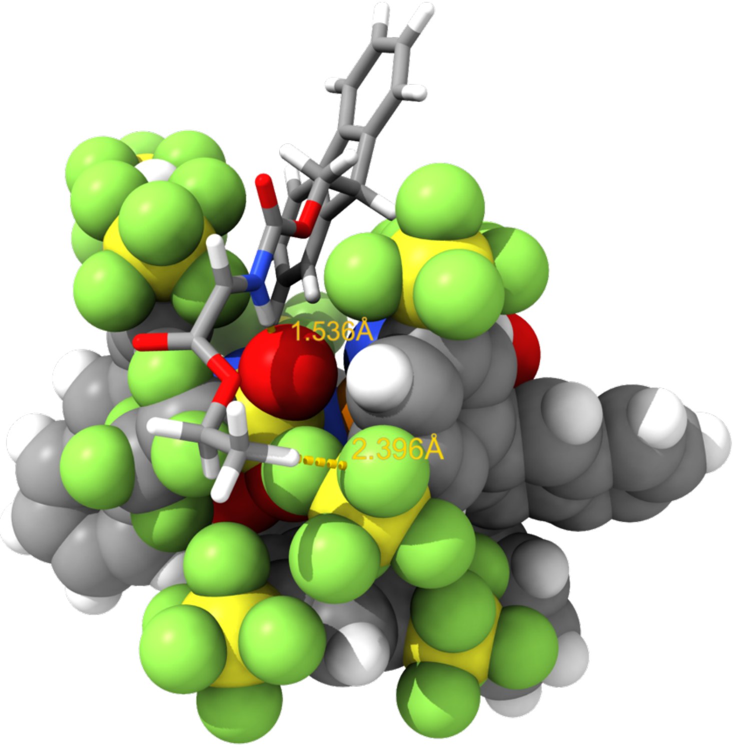

Figure SI-25: Calculated H–Bond interactions within ion pair **I**.

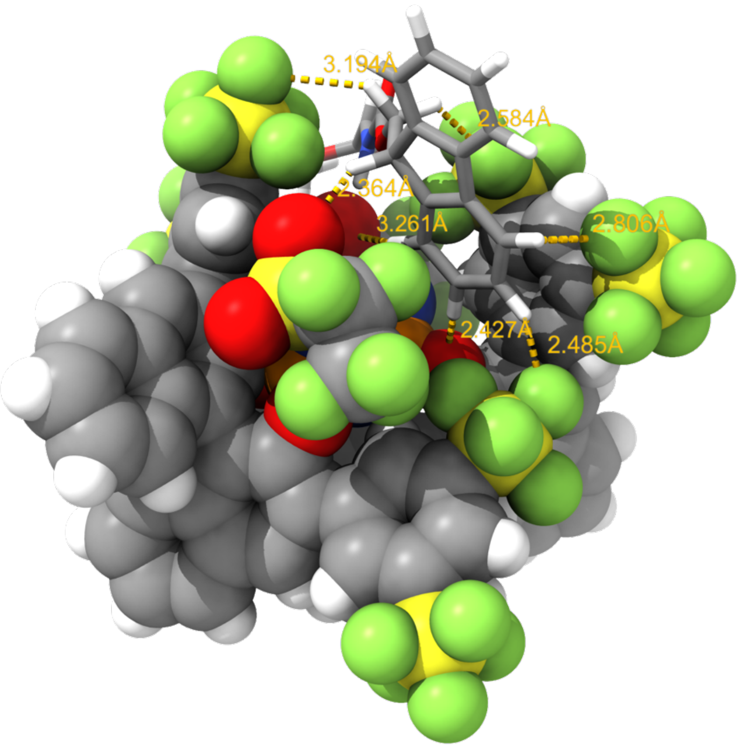

Figure SI-26: Calculated H–Bond interactions within ion pair **I**, interactions with the fluorenyl backbone of electrophile **2c**.

10.2 Molecular Electrostatic Potential

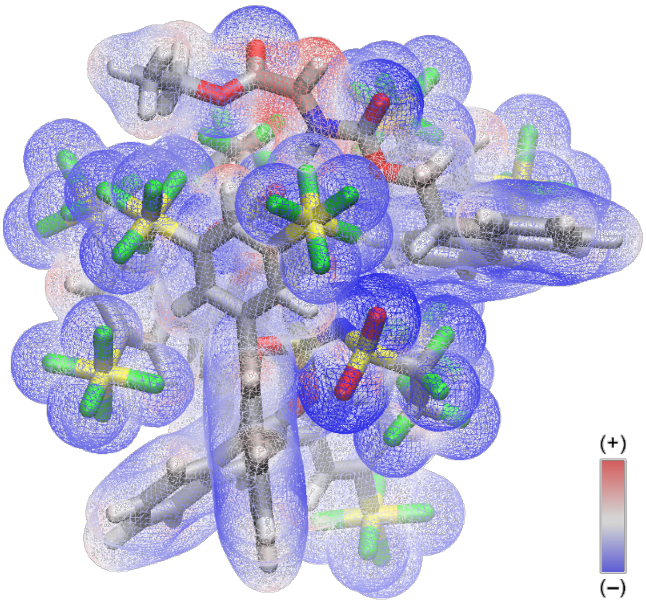

Figure SI-27: Isosurface of the electron density ($\boldsymbol{\rho}$ = 0.015) colored by electrostatic potential.

10.3 XYZ Structures

Electronic energy (a. u.) | M06-2X/def2-TZVP	–14123.9575606678
Thermochemical Corrections (a. u.) | PBE-D3(BJ)/def2-SVP	1.149237608
Imaginary Frequencies	Zero
Gibbs Free Energy (a. u.)	–14122.808323060

```
215

  C      -4.603982     -3.976447      6.420440
  C      -3.894899     -4.974413      5.701380
  C      -4.725026     -2.701054      5.892636
  C      -3.339889     -4.693191      4.461645
  C      -4.152680     -2.375437      4.628409
  C      -3.463289     -3.396966      3.880877
  H      -2.781364     -5.469984      3.922072
  H      -5.042245     -4.214132      7.401221
  H      -3.775758     -5.978669      6.135106
  H      -5.252549     -1.911092      6.449020
  C      -2.882926     -3.057067      2.608800
  C      -4.241793     -1.060756      4.097379
  C      -3.628141     -0.715973      2.900515
  C      -2.912537     -1.725897      2.197307
  O      -2.188412     -1.356256      1.063825
  H      -4.799847     -0.295075      4.657507
  C      -2.249093     -4.083142      1.738170
  C      -3.008591     -5.216994      1.274686
  C      -0.906655     -3.954359      1.360497
  C      -2.339987     -6.221676      0.487907
  C      -0.951913     -6.090862      0.237299
  C      -0.204581     -4.990971      0.659172
  O      -0.196048     -2.844007      1.778140
  H      -0.451679     -6.891133     -0.324918
  C      -4.399802     -5.384739      1.538031
  C      -5.089304     -6.485035      1.051844
  C      -4.428617     -7.473422      0.275882
  C      -3.078350     -7.340028     -0.002867
  H      -4.991417     -8.337188     -0.108110
  H      -2.549849     -8.097153     -0.603163
  H      -4.935992     -4.611419      2.100992
  H      -6.167469     -6.576680      1.249816
  P      -0.532931     -1.309445      1.242440
  N       0.031395     -0.248493      2.285618
  N       0.029722     -1.190224     -0.240984
  O       2.007961     -0.330537     -1.648393
  C       2.586421     -0.527625     -2.891564
  C       2.212417     -1.613305     -3.691265
  C       1.120195     -2.544260     -3.288518
  C      -0.143559     -2.023208     -3.011325
  O      -0.256409     -0.648971     -2.813610
  C      -1.314834     -2.822540     -2.853022
  C      -1.151462     -4.204365     -2.860895
  H      -2.033994     -4.854733     -2.760233
  C       0.133901     -4.803262     -2.984073
  C       0.301108     -6.216213     -2.879717
  H      -0.597434     -6.841096     -2.762402
  C       1.563881     -6.786862     -2.887110
  H       1.688027     -7.871454     -2.755114
  C       2.710953     -5.962766     -3.036864
```

H	3.711779	-6.417005	-2.992315
C	2.579304	-4.593838	-3.220582
H	3.471922	-3.965294	-3.332409
C	1.295307	-3.972798	-3.196061
C	2.891207	-1.807803	-4.949981
C	2.489842	-2.793314	-5.900649
H	1.615854	-3.422076	-5.685428
C	3.177939	-2.958422	-7.094160
H	2.843147	-3.722025	-7.812268
C	4.301750	-2.147659	-7.402557
H	4.840471	-2.291187	-8.351056
C	4.704750	-1.165158	-6.512906
H	5.561914	-0.514463	-6.745540
C	4.012520	-0.964167	-5.282307
C	4.397212	0.065507	-4.382631
H	5.260083	0.700152	-4.637151
C	3.691184	0.318639	-3.212301
P	0.400457	-0.153256	-1.370609
N	0.162545	1.440334	-1.228737
S	-0.138781	-0.192808	3.902157
C	1.566553	-0.687205	4.599614
S	-1.273099	2.119097	-1.381384
C	-1.063166	3.170580	-2.960427
O	-1.056074	-1.201681	4.479566
O	-0.280819	1.223429	4.314658
O	-1.466571	3.187925	-0.344158
O	-2.410860	1.208013	-1.608041
C	2.095688	-2.094310	4.202901
C	-0.507224	2.470811	-4.234297
F	1.401968	-0.662962	5.941829
F	2.485061	0.230010	4.248699
F	3.202536	-2.370270	4.919929
F	1.183726	-3.045160	4.446226
F	2.423739	-2.121535	2.898942
F	-0.542168	3.346407	-5.251679
F	-1.245703	1.404509	-4.557753
F	0.770240	2.081911	-4.055375
F	-0.233027	4.195743	-2.654422
F	-2.281725	3.674170	-3.257233
C	-3.750554	0.668739	2.374621
C	-4.463519	0.903342	1.180887
C	-3.258695	1.756681	3.123682
C	-4.696460	2.219241	0.776882
C	-4.226256	3.319928	1.499311
C	-3.519968	3.054366	2.673707
H	-2.664514	1.574155	4.029399
H	-4.866239	0.055856	0.614740
H	-4.415294	4.343787	1.165220
C	1.274947	-5.034226	0.503915
C	2.097809	-3.911435	0.250458
H	1.645976	-2.927058	0.081212
C	3.488855	-4.080690	0.206296
C	4.107539	-5.328534	0.337753
H	5.194013	-5.443194	0.292152
C	3.269750	-6.429454	0.516769
C	1.889503	-6.300147	0.634802
H	1.278379	-7.183503	0.850650
C	-2.662448	-2.203398	-2.781447
C	-3.629757	-2.681809	-1.877457
C	-4.930291	-2.173064	-1.953177
C	-5.317823	-1.209348	-2.884660
H	-6.345312	-0.838534	-2.919404

C	-4.329051	-0.713042	-3.741023
C	-3.015527	-1.184368	-3.695077
H	-2.259574	-0.795308	-4.382721
C	4.153273	1.408180	-2.307922
C	5.472027	1.360046	-1.812301
H	6.097156	0.484697	-2.023855
C	5.960668	2.417657	-1.041298
C	5.189706	3.543826	-0.744574
H	5.587304	4.365650	-0.140358
C	3.887890	3.566411	-1.251713
C	3.344170	2.521266	-2.003695
H	-3.359166	-3.435923	-1.127228
H	2.314957	2.573772	-2.374979
S	-6.189305	-2.768538	-0.770780
S	-4.807701	0.572200	-4.969047
F	-5.682536	-0.521309	-5.818547
F	-3.502815	0.258071	-5.911930
F	-3.967367	1.731780	-4.177675
F	-6.153265	0.993934	-4.134231
F	-5.232666	1.701495	-6.073454
F	-6.732647	-1.245051	-0.467184
F	-7.322753	-2.930385	-1.939364
F	-5.132404	-2.621183	0.490189
F	-5.729575	-4.326009	-1.001333
F	-7.288883	-3.317006	0.305480
S	-2.978778	4.476887	3.689594
S	-5.700265	2.538644	-0.719371
F	-2.538887	5.346594	2.336016
F	-2.481868	5.765238	4.569381
F	-3.385999	3.737287	5.084382
F	-1.456663	3.891775	3.805580
F	-4.459998	5.172073	3.615880
F	-6.984547	1.732979	-0.109570
F	-6.590223	2.863482	-2.054943
F	-5.196087	1.175149	-1.456134
F	-4.468542	3.380352	-1.414664
F	-6.298109	3.946175	-0.088695
H	0.800616	3.321817	4.196383
H	1.108709	2.286032	1.441724
H	0.633794	5.802492	3.865508
H	1.445251	5.474542	6.283672
H	-0.983726	4.608030	-0.013422
O	0.433151	4.937527	1.983896
C	1.685147	3.883234	3.825734
C	1.249096	5.190095	3.177107
C	2.135802	2.239245	1.826325
C	2.435494	5.037994	6.078296
N	-0.819664	5.653998	0.230215
C	2.524863	2.978293	2.945166
C	2.648191	4.241364	4.949796
C	-0.008182	6.026509	1.405106
H	2.786982	0.773799	0.367305
H	3.354415	5.887762	7.857127
C	3.078765	1.385401	1.229191
C	3.507736	5.266596	6.961841
H	2.122728	5.786607	2.848386
C	-1.346723	6.615623	-0.447156
C	3.840996	2.870888	3.466753
C	3.923306	3.670800	4.694877
O	-3.335927	6.019151	-1.584975
C	-2.106829	6.478356	-1.731046
O	0.146424	7.184563	1.708370

C	4.387659	1.283307	1.734550
C	4.772737	4.704567	6.710121
C	4.783748	2.031591	2.853448
C	4.990302	3.902039	5.578334
H	5.101474	0.596342	1.261273
H	5.600598	4.894446	7.410262
O	-1.540816	6.871558	-2.733947
H	5.980714	3.462054	5.385966
H	5.806953	1.942891	3.248114
C	-4.211618	6.010545	-2.770062
C	-5.588154	6.470731	-2.349952
H	-4.218544	4.965916	-3.129876
H	-3.744606	6.655941	-3.539131
H	-6.016556	5.794771	-1.587138
H	-6.255870	6.452424	-3.234278
H	-5.570250	7.505373	-1.954359
H	-1.100047	7.638500	-0.096644
S	4.589158	-2.628717	-0.025970
S	4.008771	-8.100613	0.559433
F	3.465978	-1.587959	0.534032
F	5.577038	-1.325806	-0.239941
F	5.812109	-3.537577	-0.638820
F	5.210308	-2.791378	1.472736
F	4.046573	-2.388391	-1.560301
F	2.834467	-8.698548	-0.429927
F	5.226930	-7.609370	1.536160
F	4.934906	-7.773806	-0.760486
F	4.664012	-9.593206	0.580056
F	3.120257	-8.528820	1.867266
S	2.878777	5.059985	-0.956472
F	4.123101	6.063853	-1.295032
F	2.401347	5.126094	-2.520968
F	1.952790	6.406133	-0.693936
F	1.545053	4.169855	-0.590941
F	3.277620	5.114893	0.636711
S	7.677221	2.339884	-0.406596
F	8.225575	1.732430	-1.828730
F	7.463116	0.816748	0.161492
F	9.210135	2.273842	0.145121
F	7.990102	3.859607	-0.938572
F	7.236248	2.946246	1.051753

11. NMR Spectra

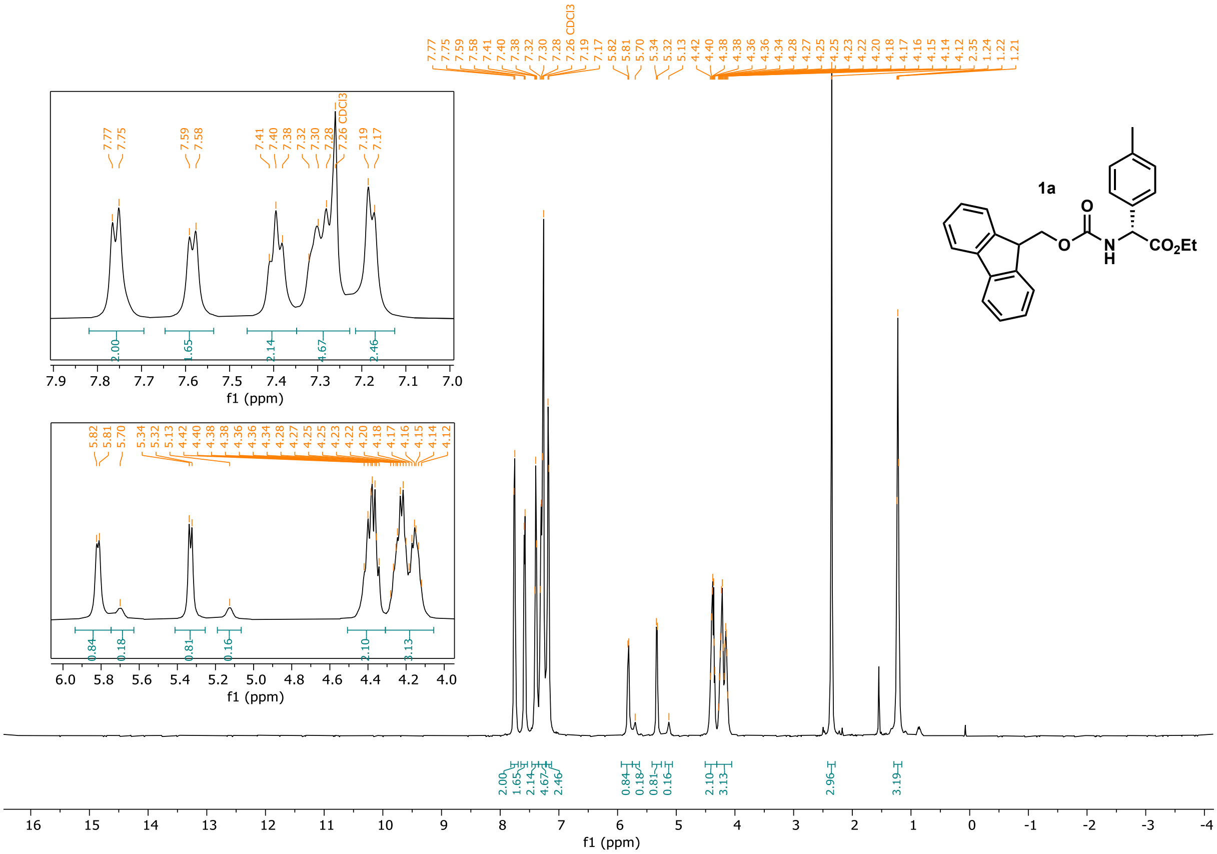

^{1}**H-NMR** spectrum of compound **1a**.

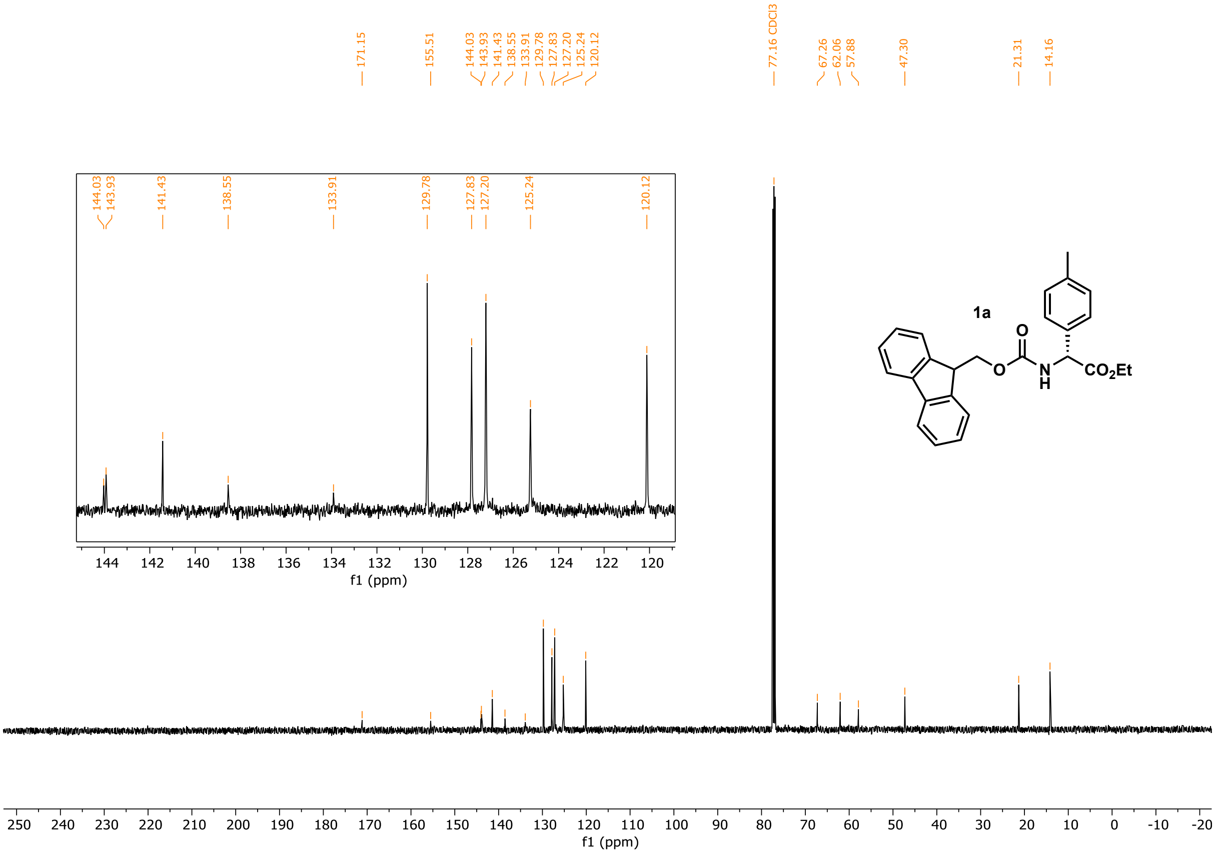

^{13}**C-NMR** spectrum of compound **1a**.

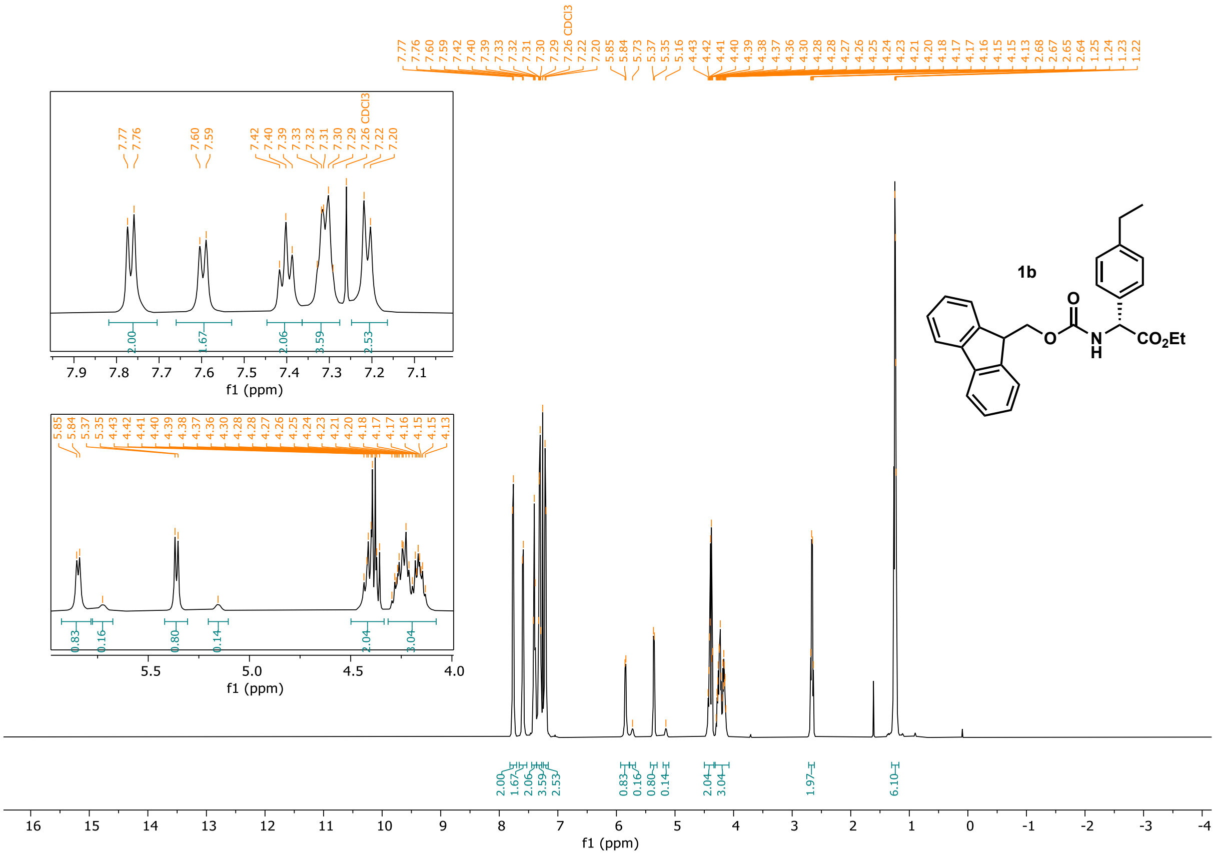

^{1}**H-NMR** spectrum of compound **1b**.

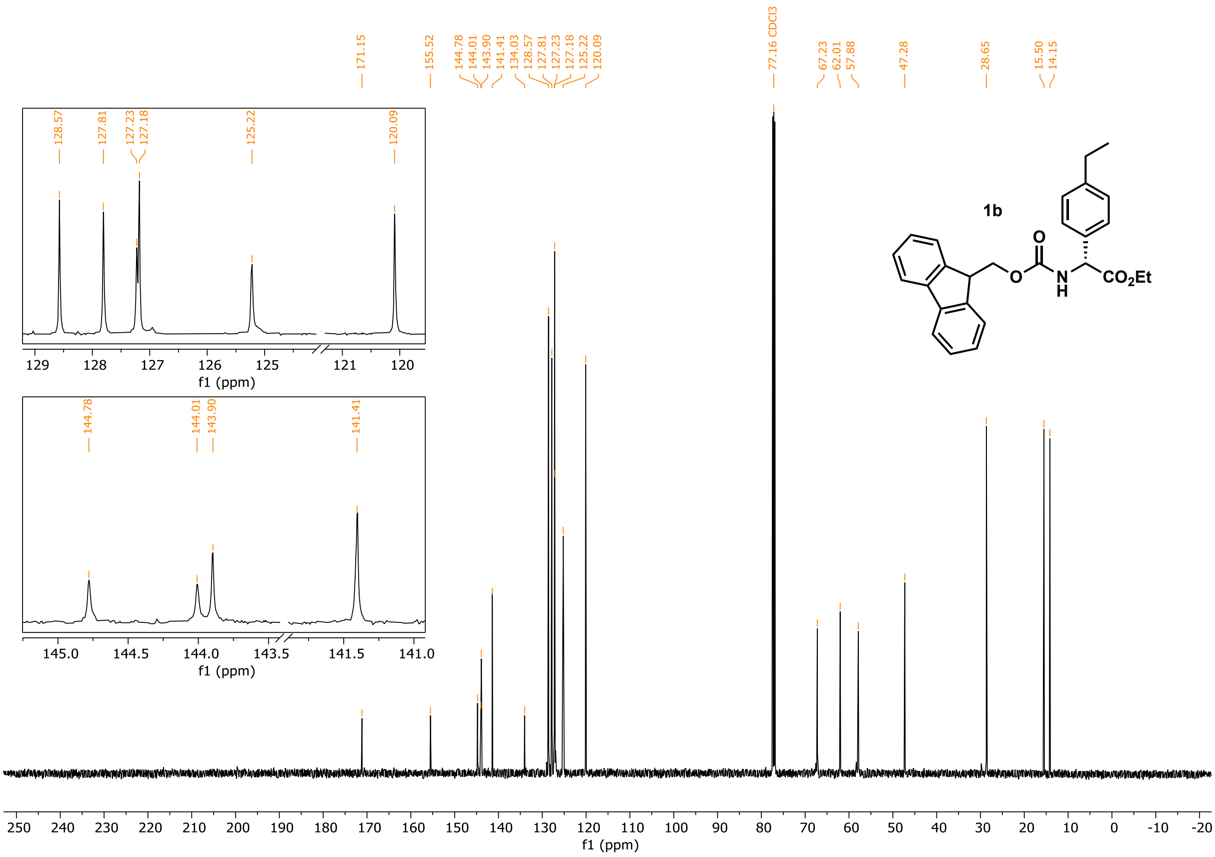

^{13}**C-NMR** spectrum of compound **1b**.

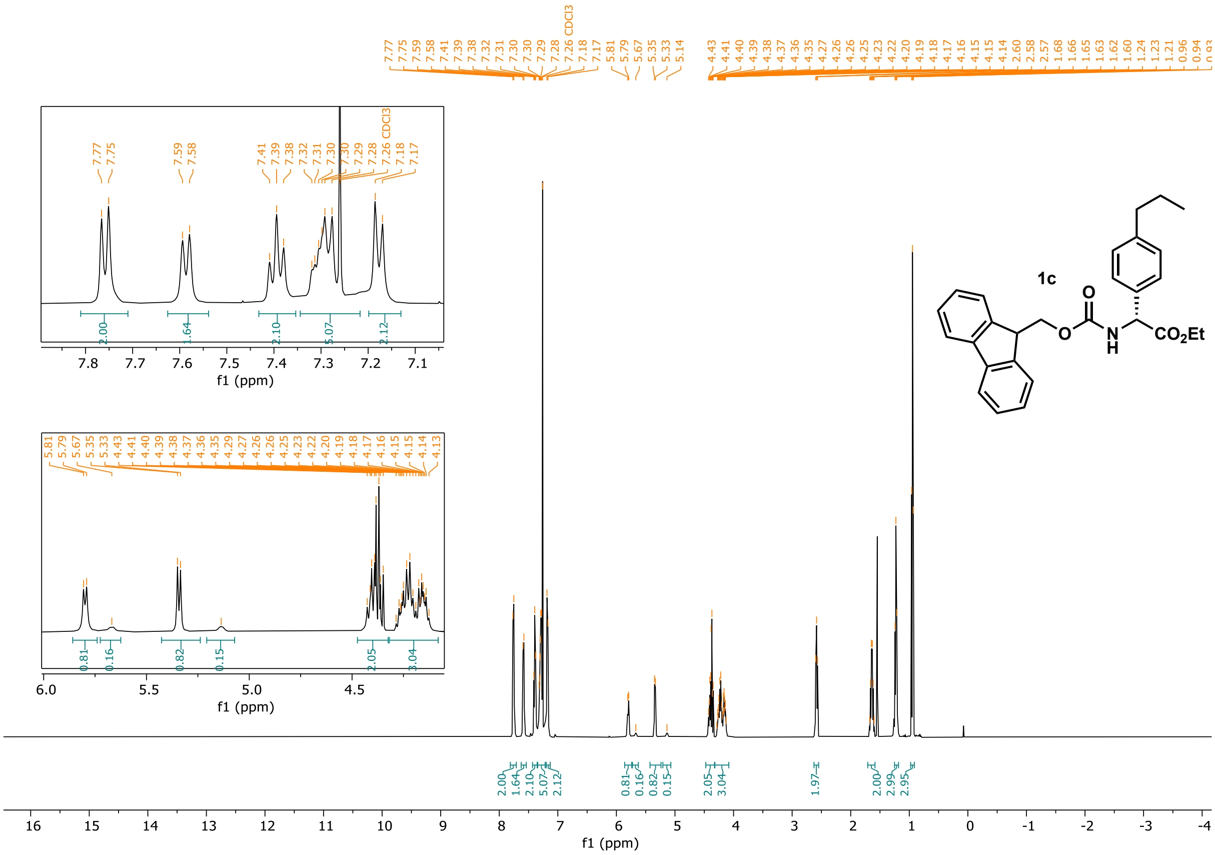

^{1}H-NMR spectrum of compound **1c**.

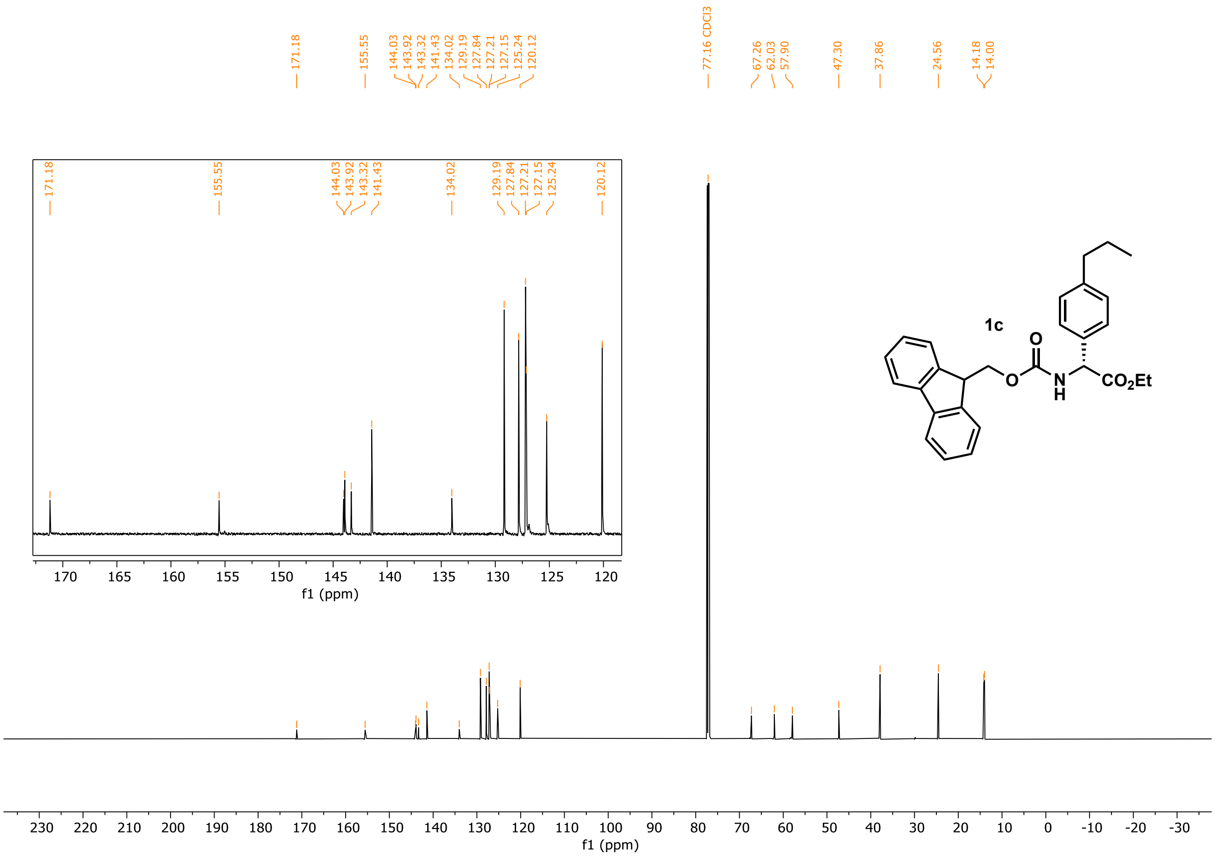

^{13}C-NMR spectrum of compound **1c**.

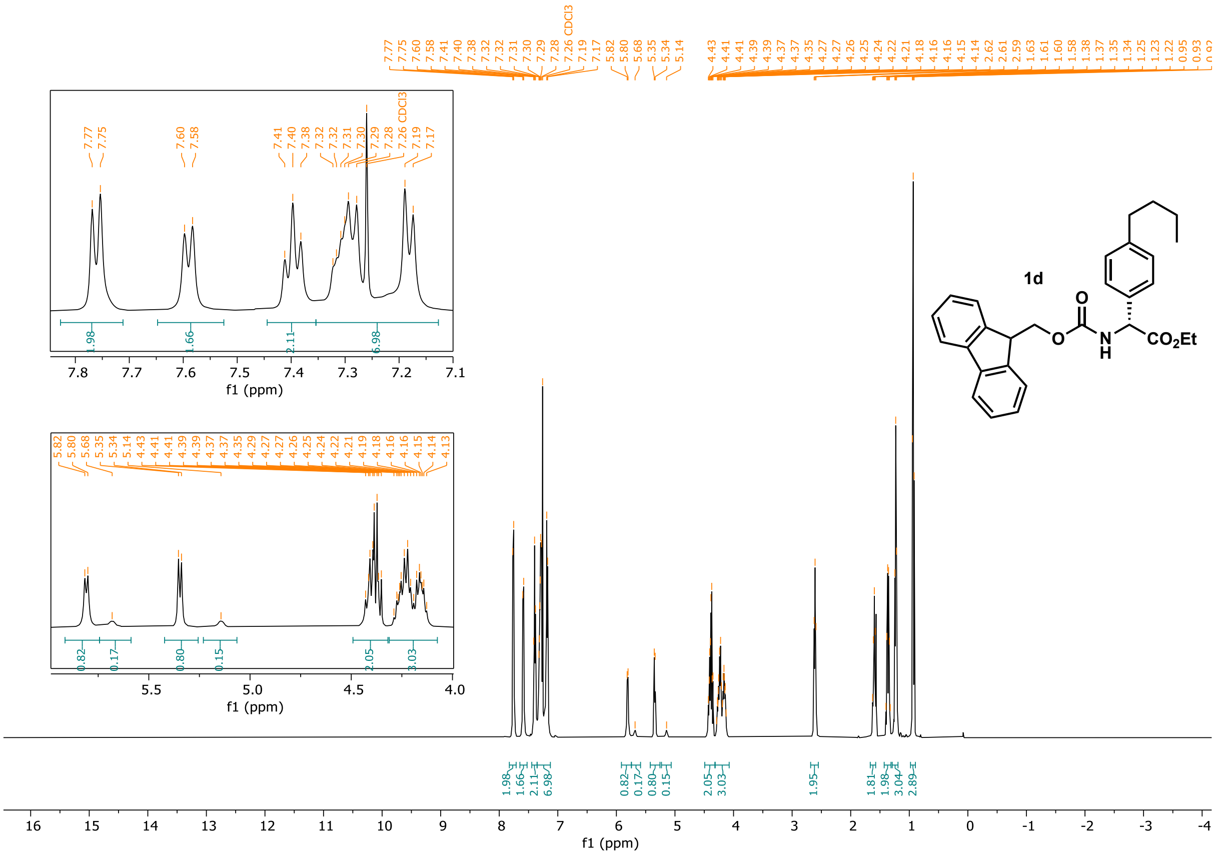

^{1}H-NMR spectrum of compound **1d**.

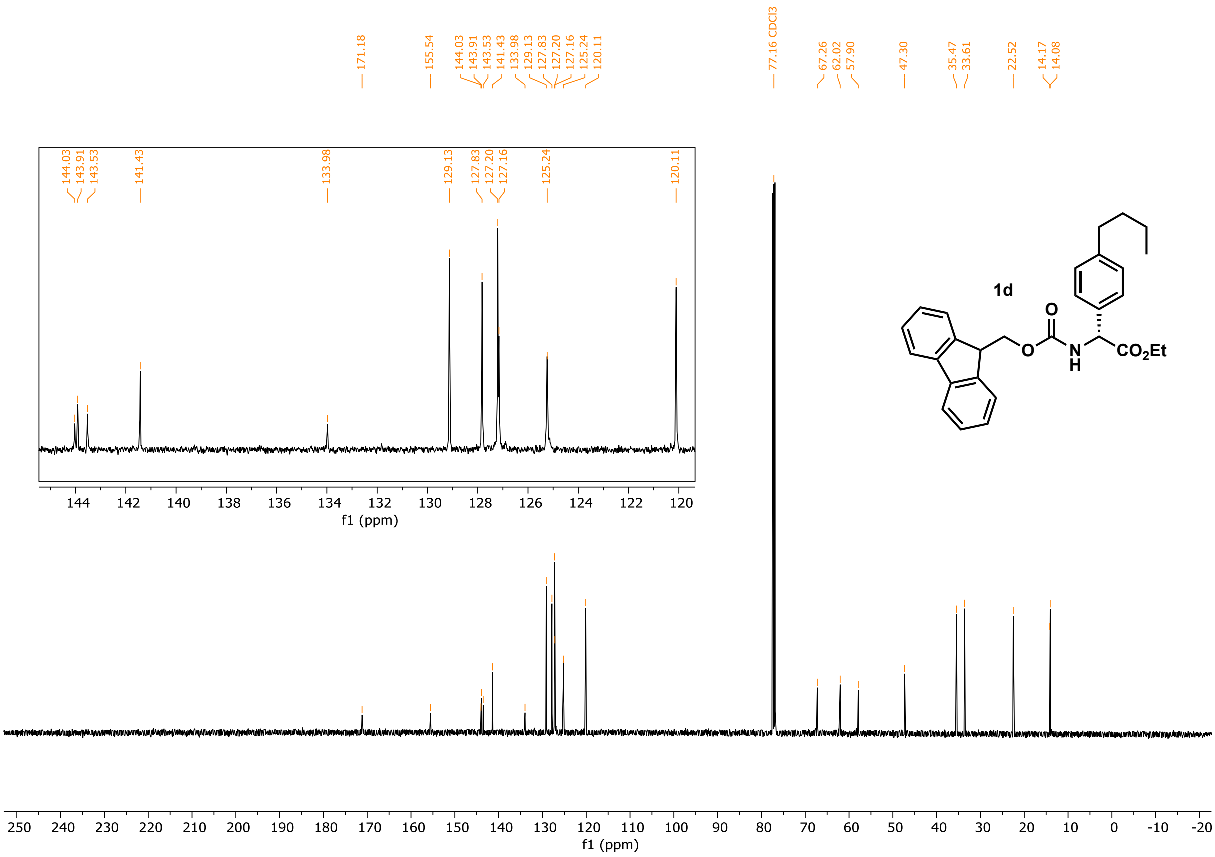

^{13}C-NMR spectrum of compound **1d**.

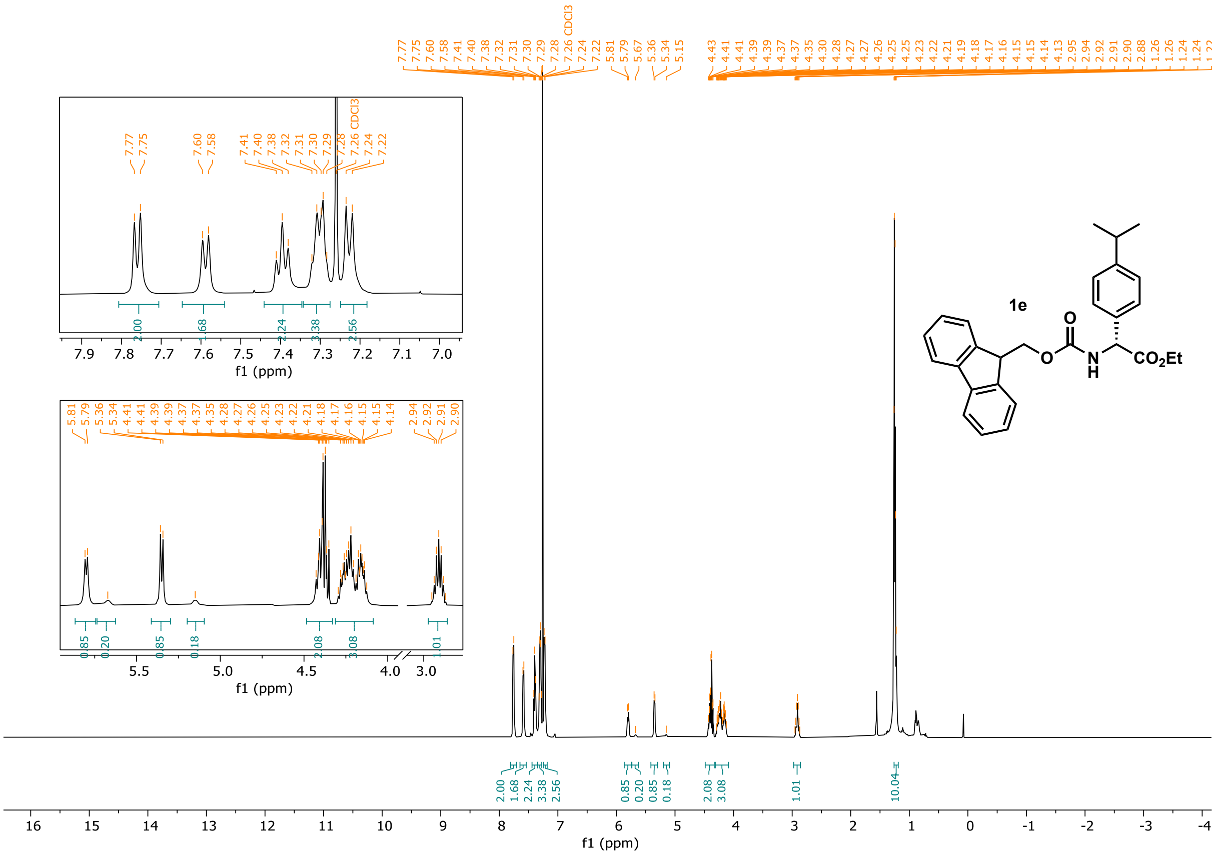

^{1}**H-NMR** spectrum of compound **1e**.

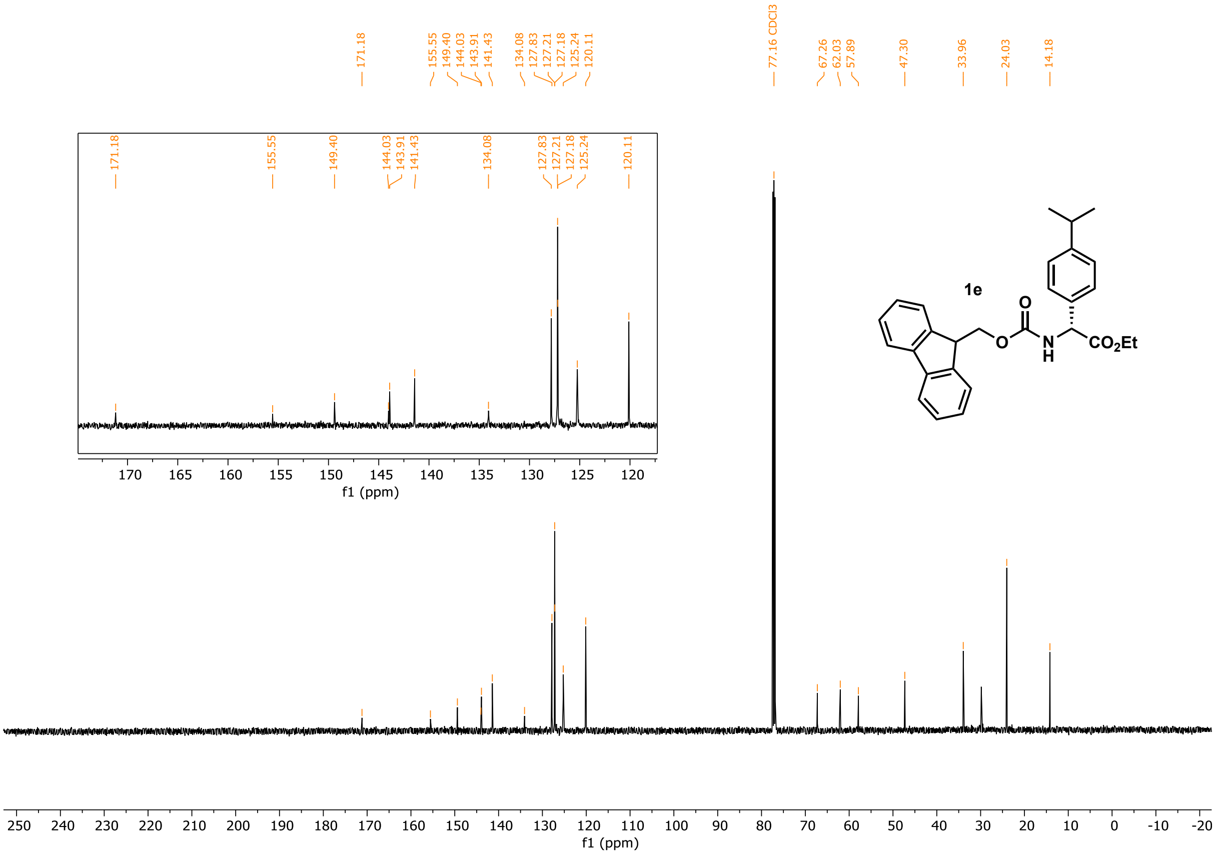

^{13}**C-NMR** spectrum of compound **1e**.

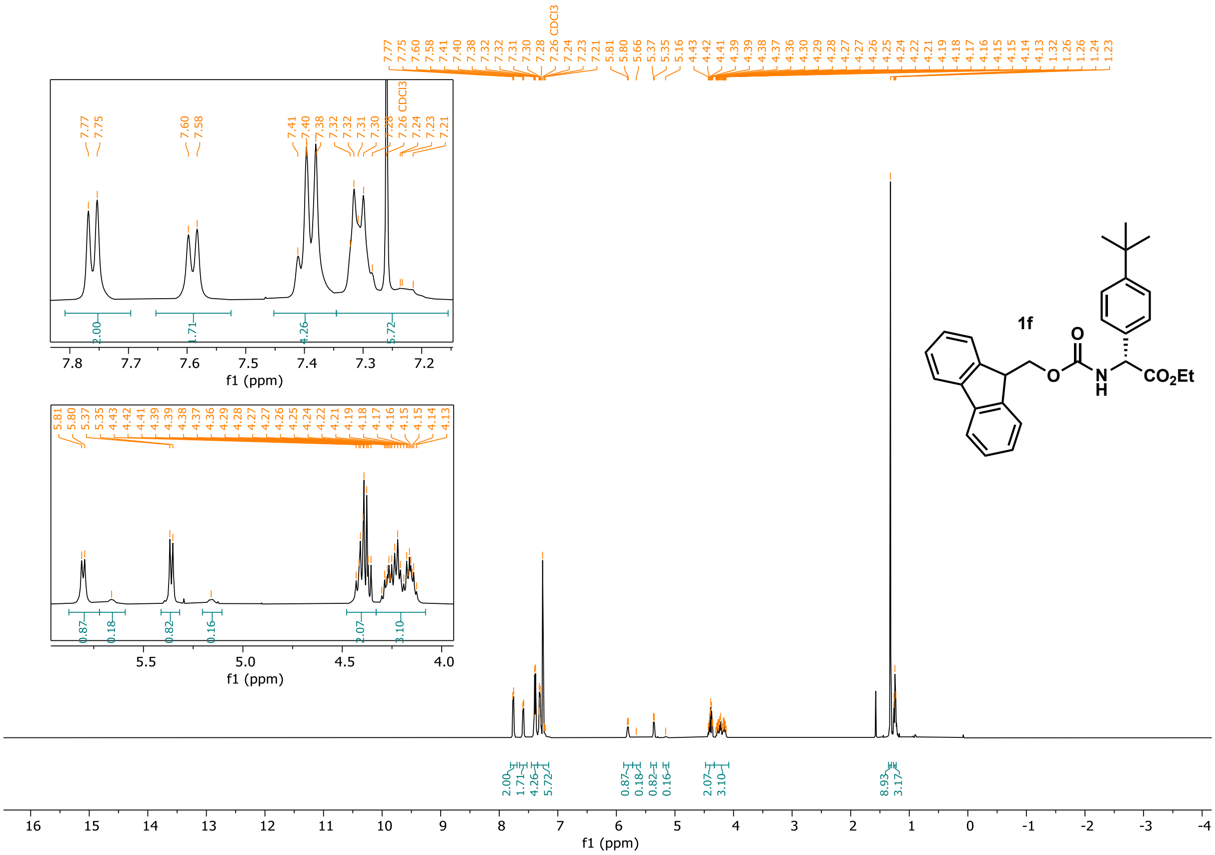

^{1}H-NMR spectrum of compound **1f**.

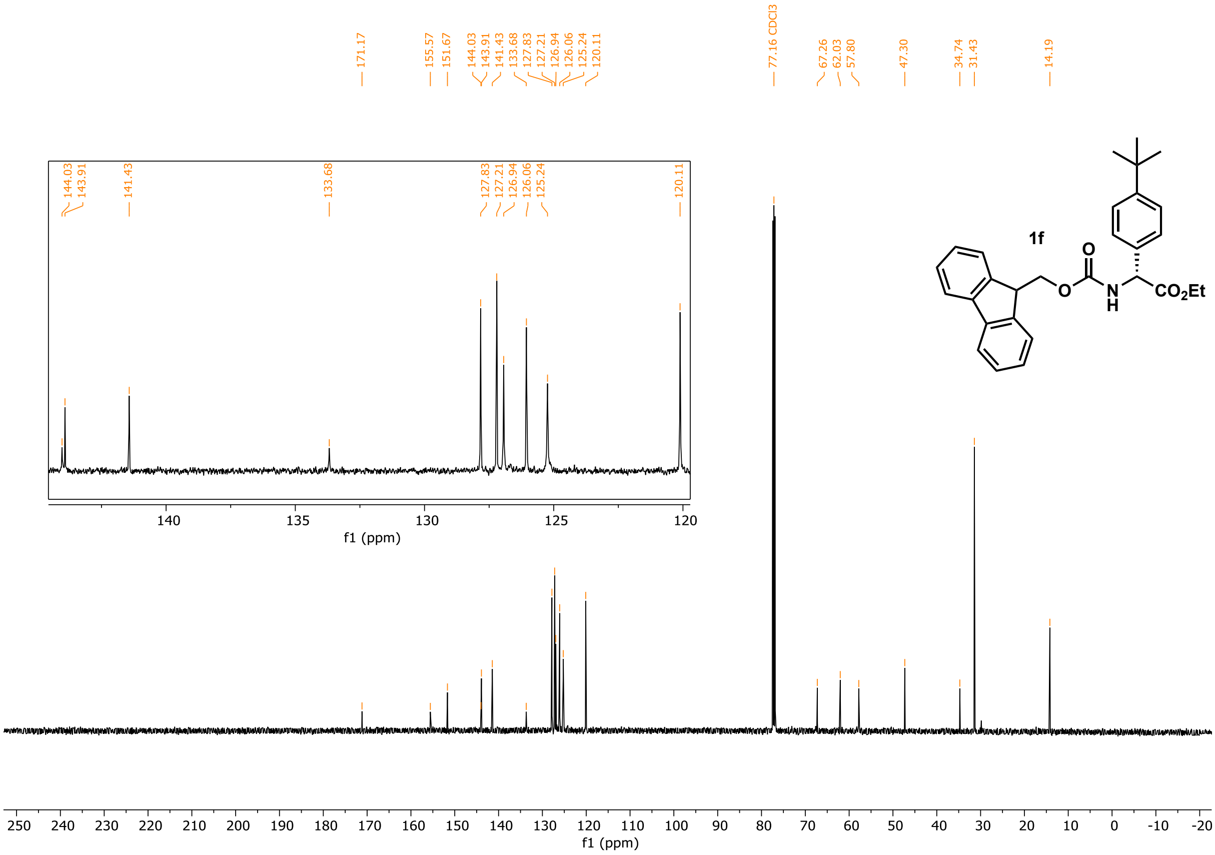

^{13}C-NMR spectrum of compound **1f**.

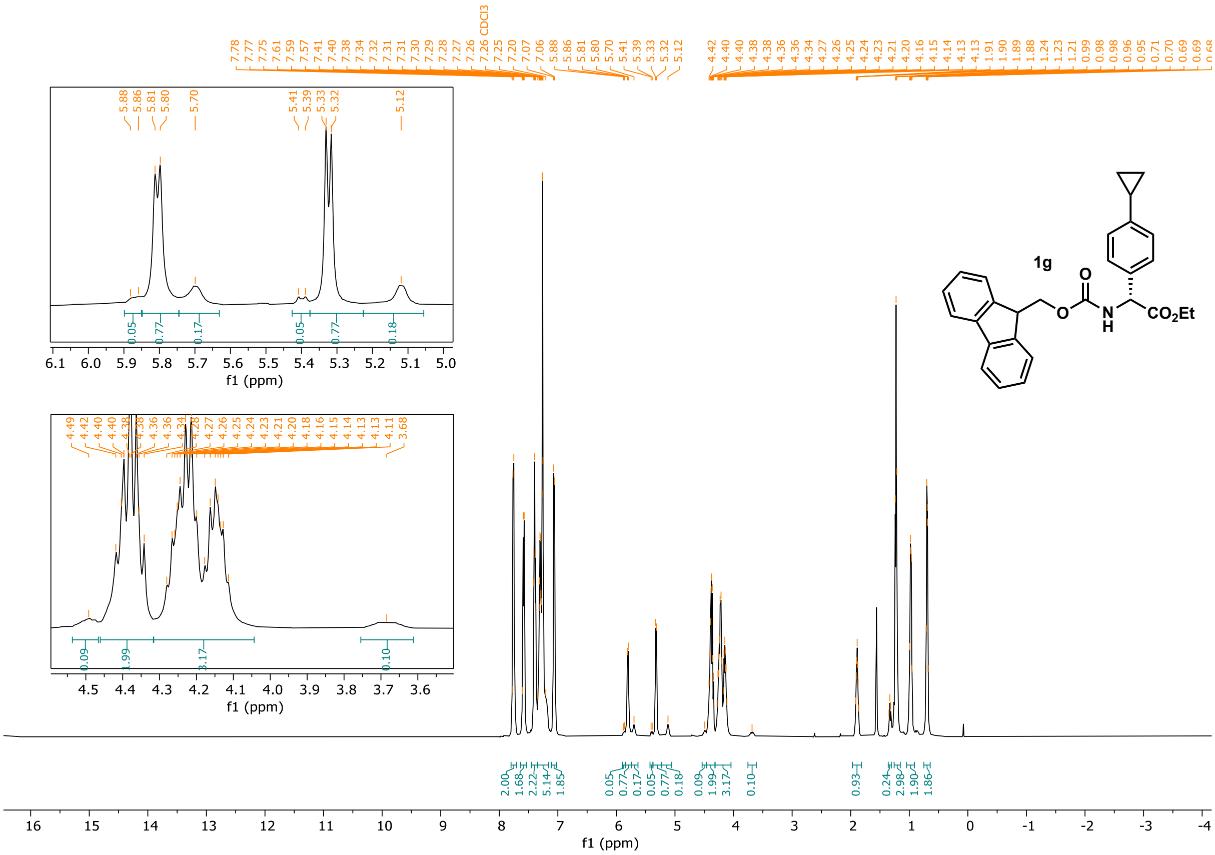

^{1}**H-NMR** spectrum of compound **1g**.

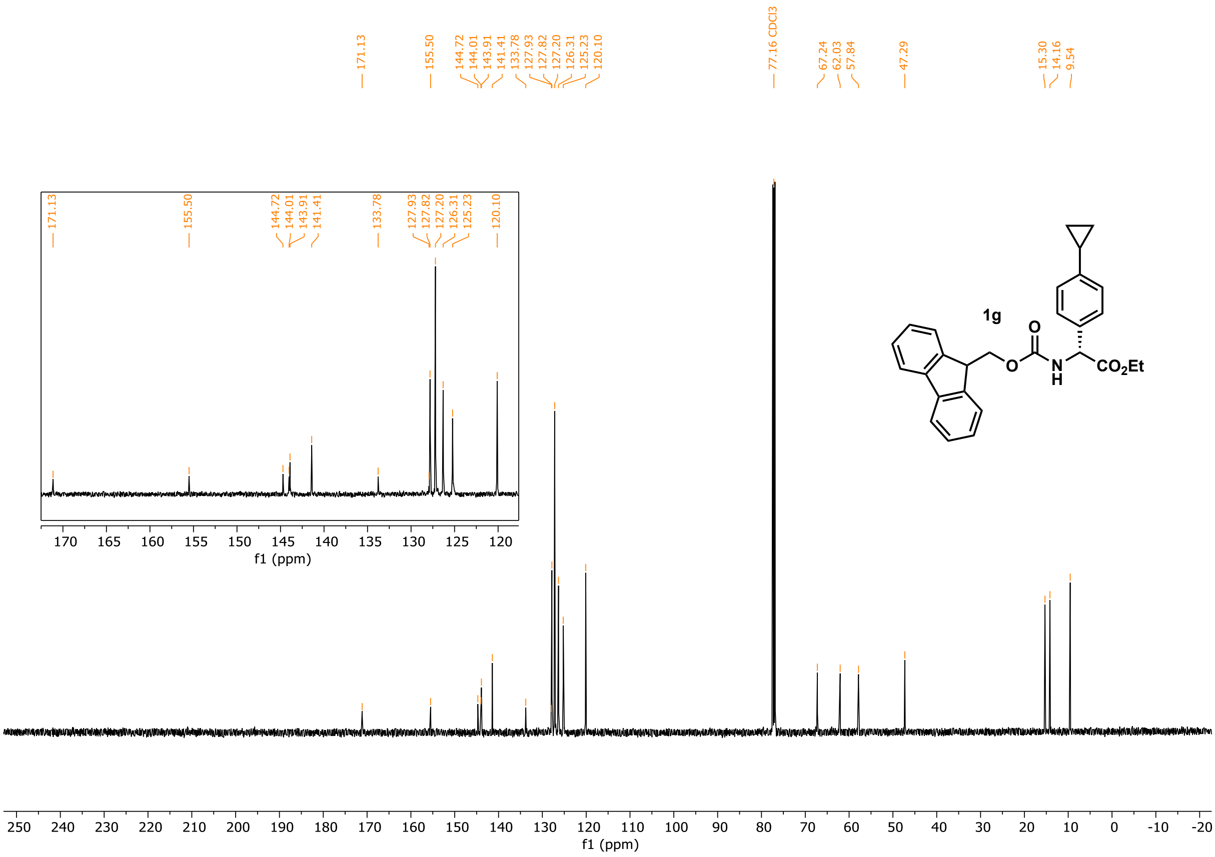

^{13}**C-NMR** spectrum of compound **1g**.

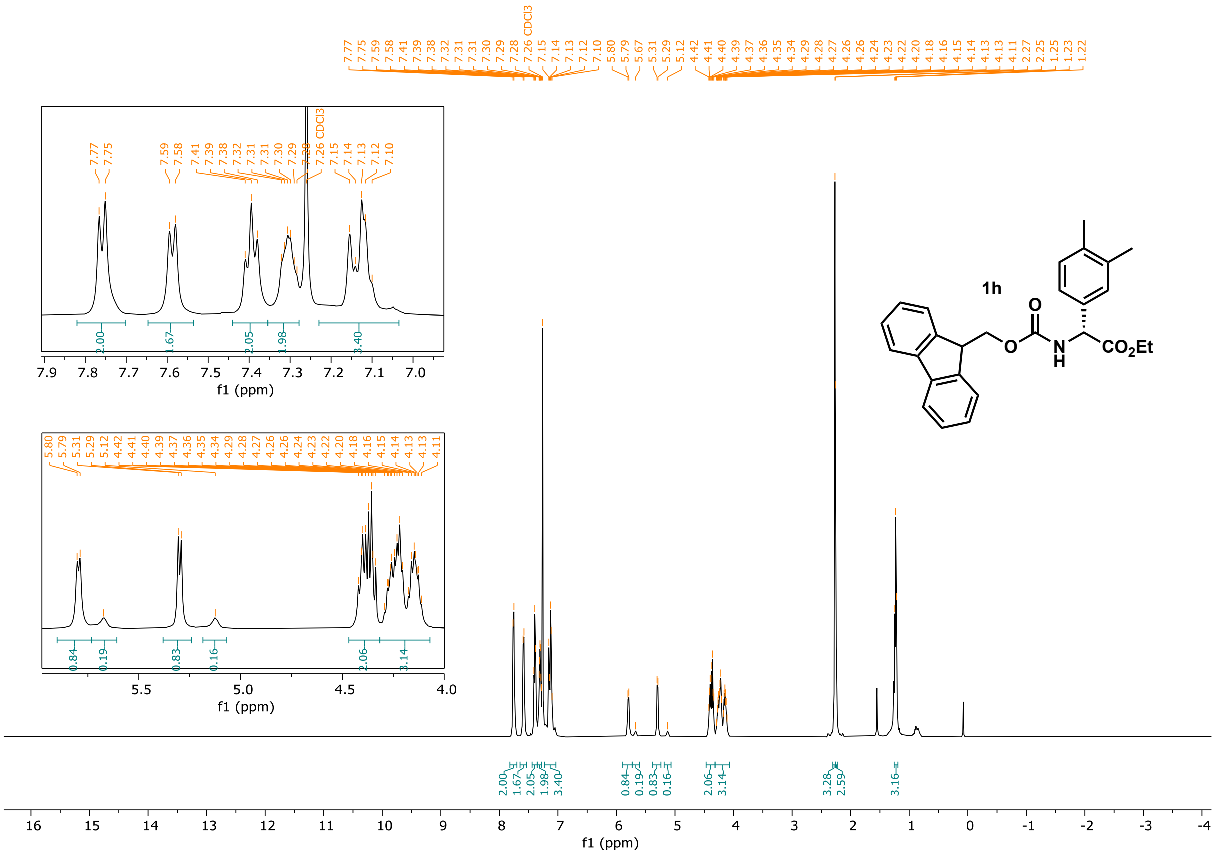

[1]**H-NMR** spectrum of compound **1h**.

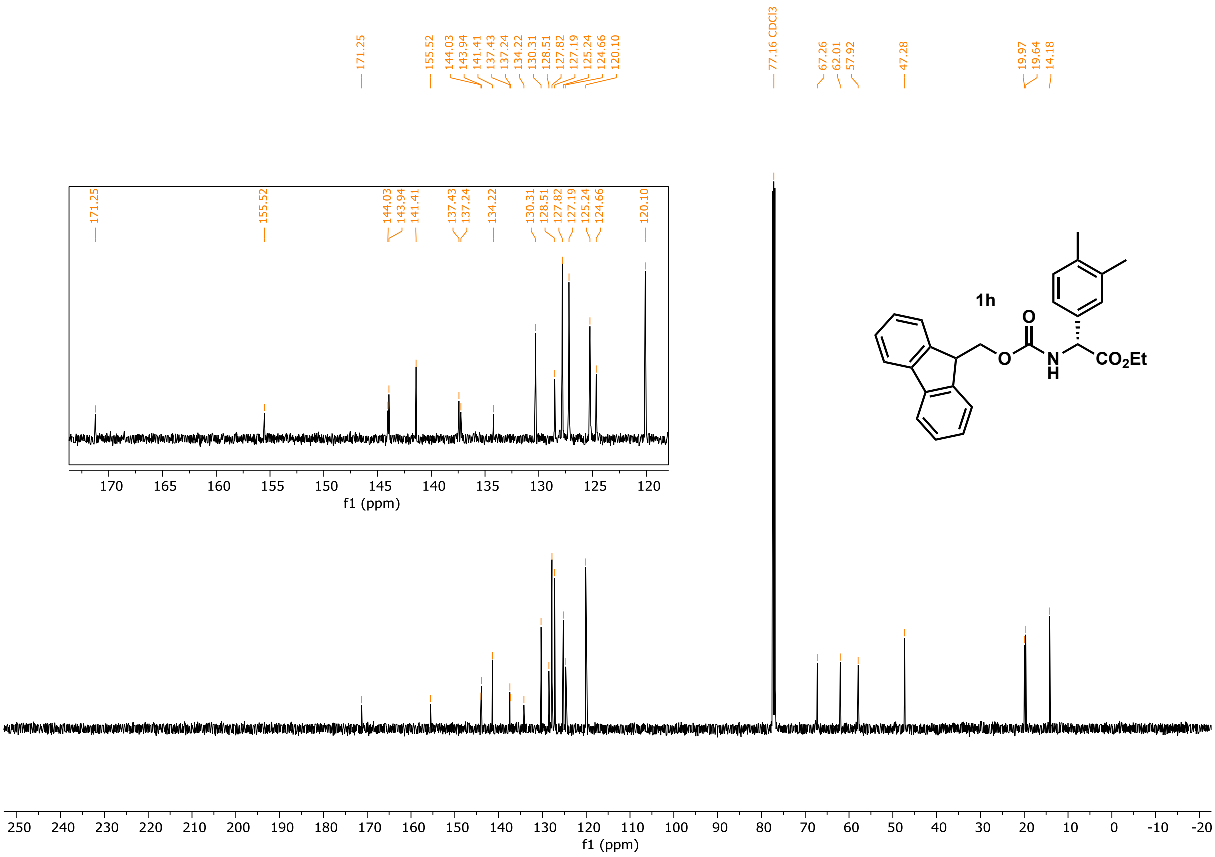

[13]**C-NMR** spectrum of compound **1h**.

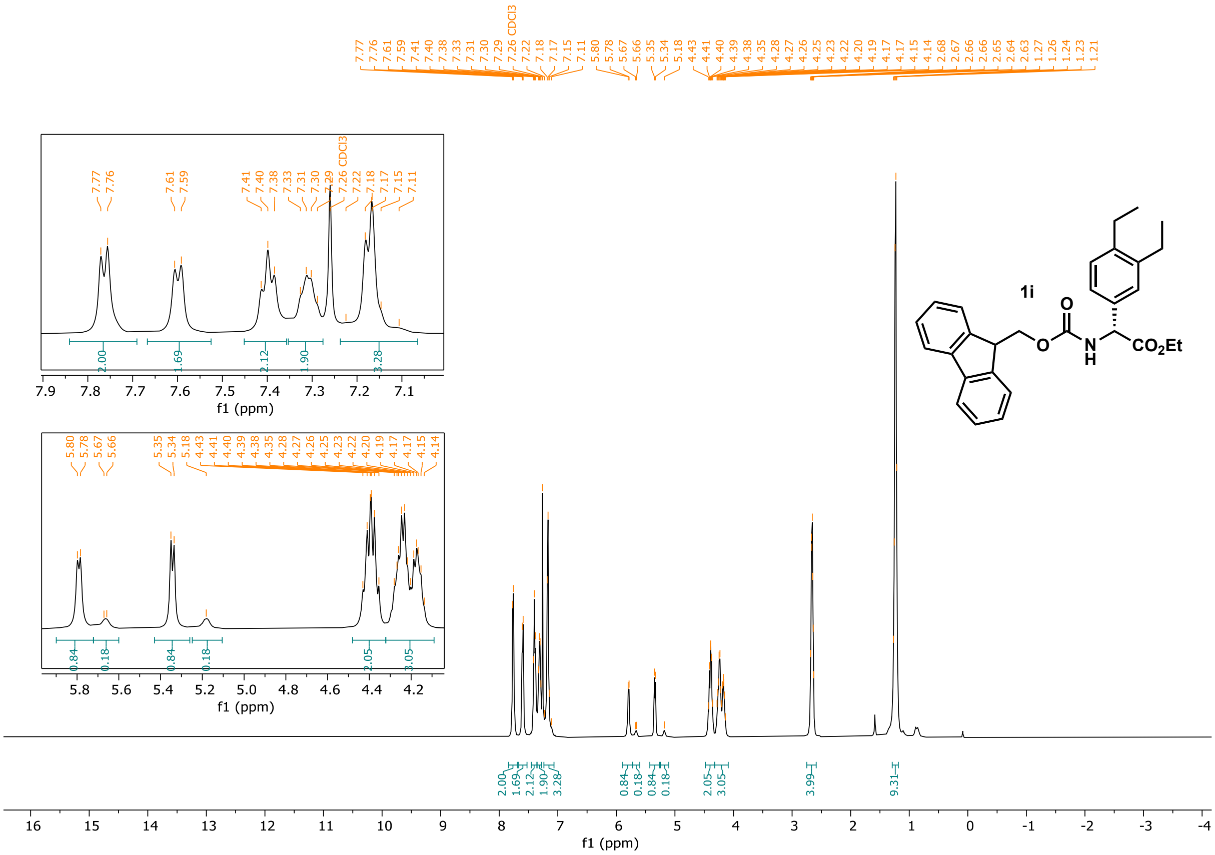

^{1}**H-NMR** spectrum of compound **1i**.

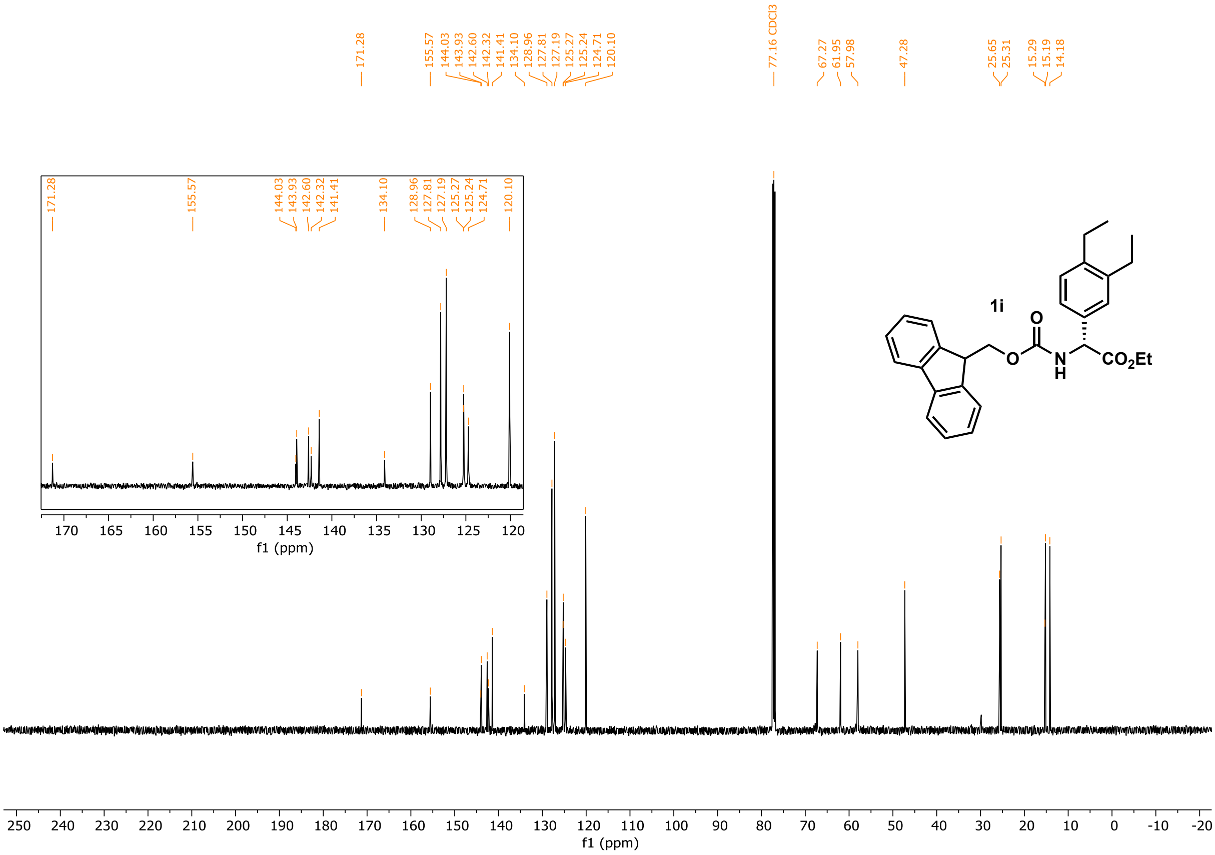

^{13}**C-NMR** spectrum of compound **1i**.

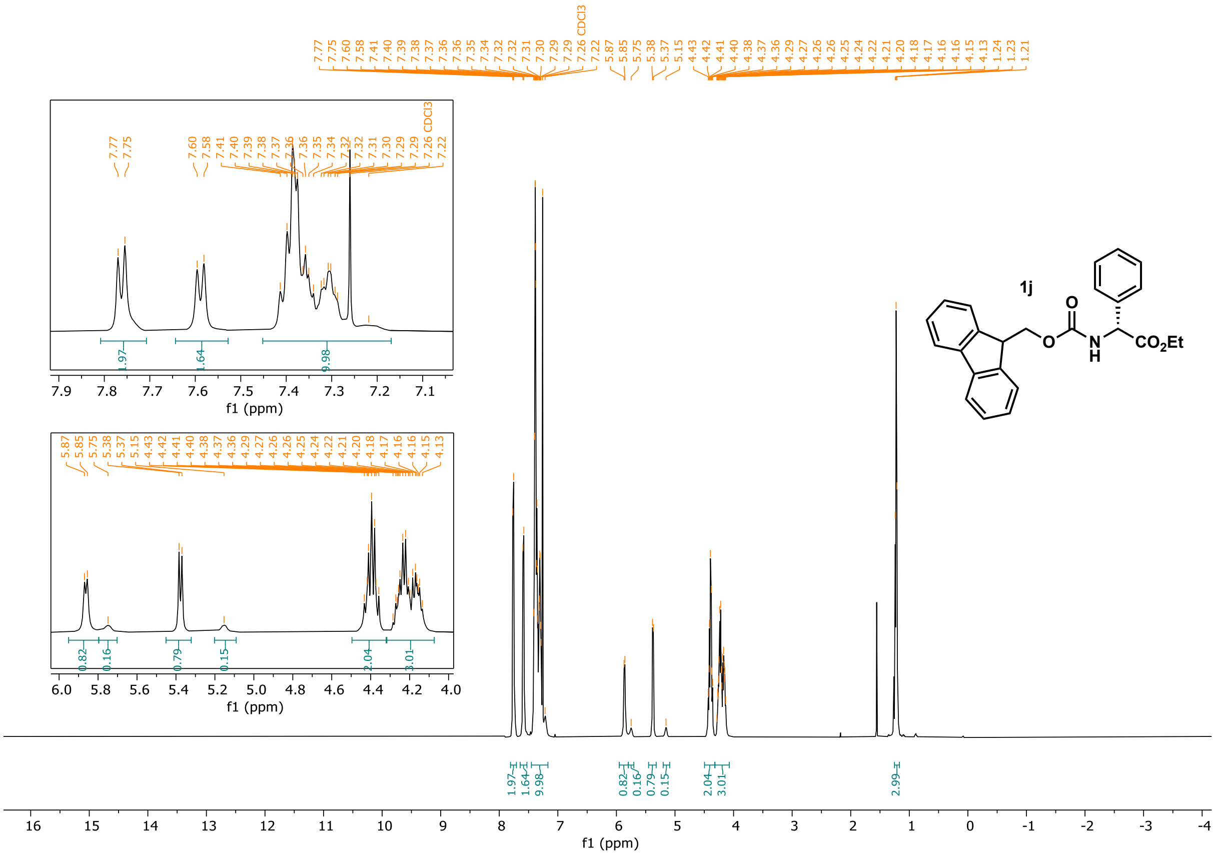

¹H-NMR spectrum of compound **1j**.

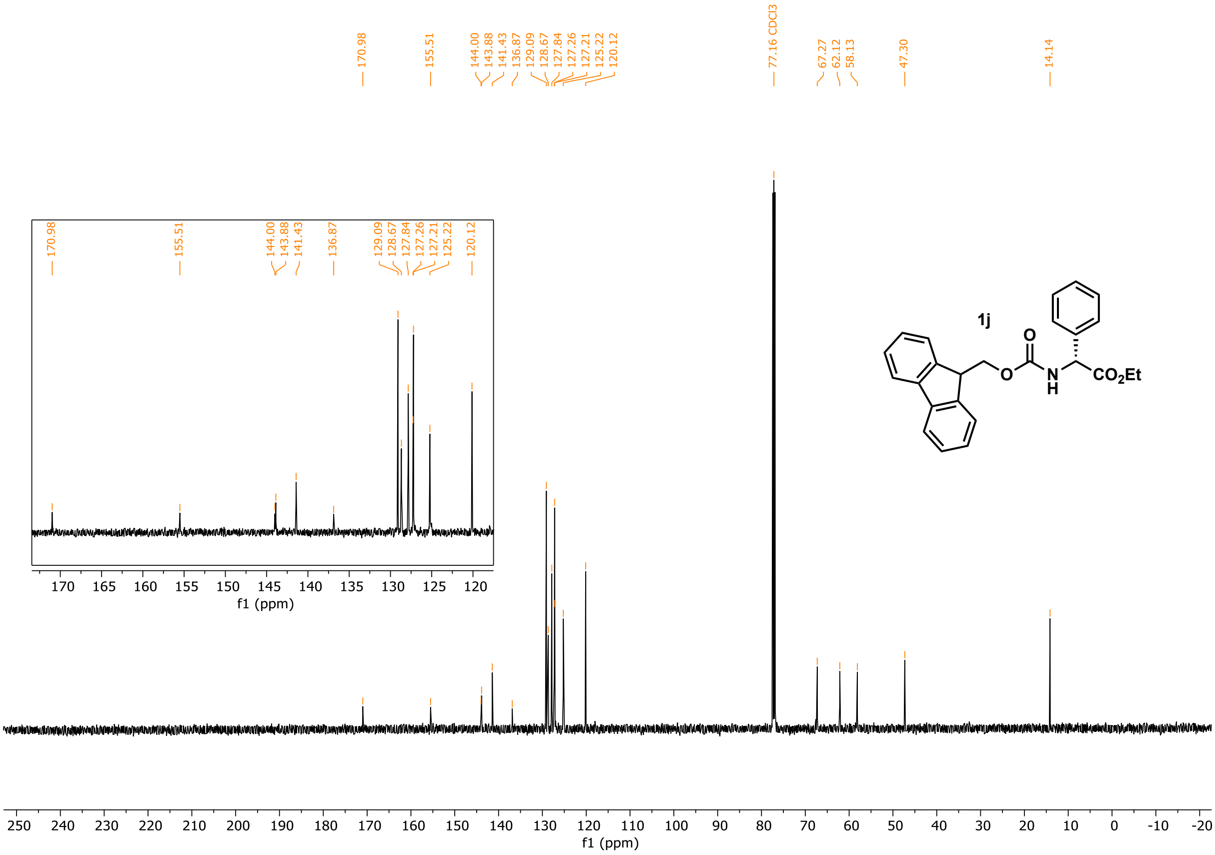

¹³C-NMR spectrum of compound **1j**.

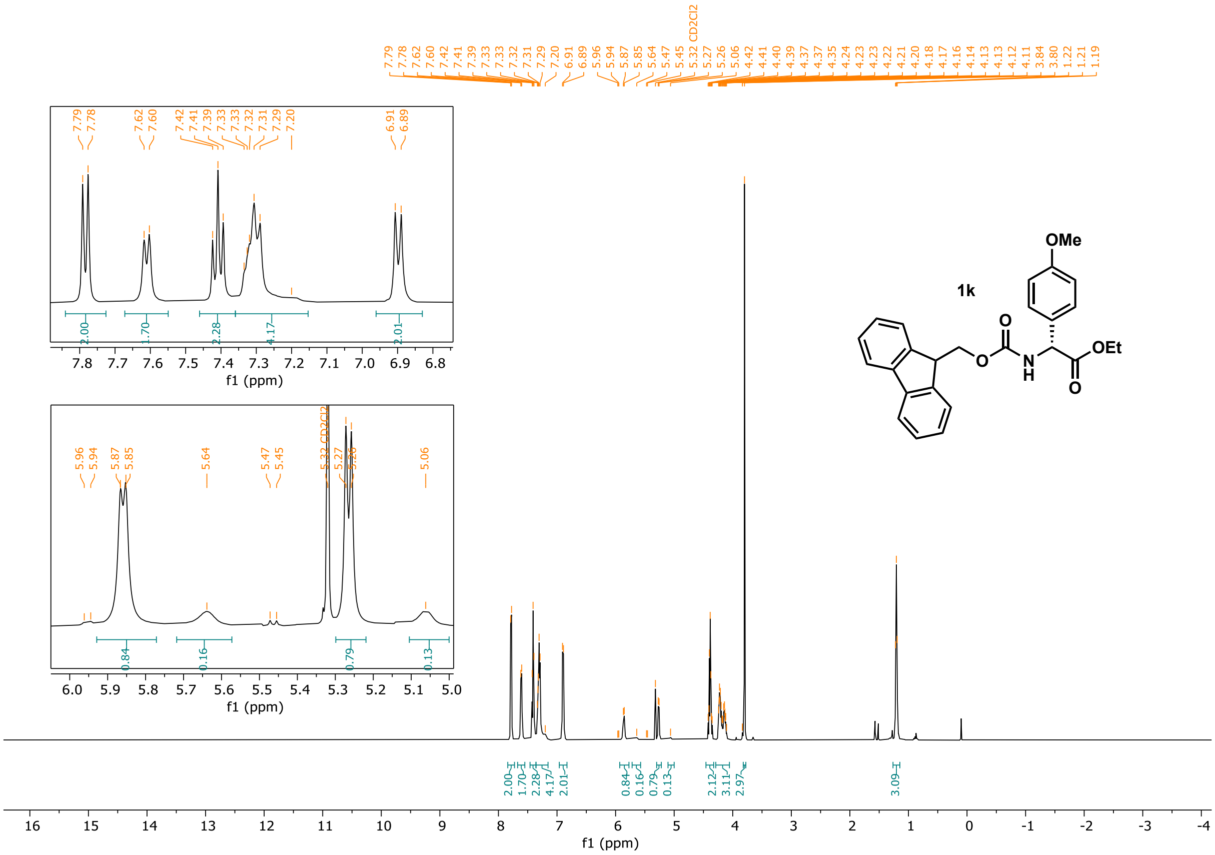

^{1}H-NMR spectrum of compound **1k**.

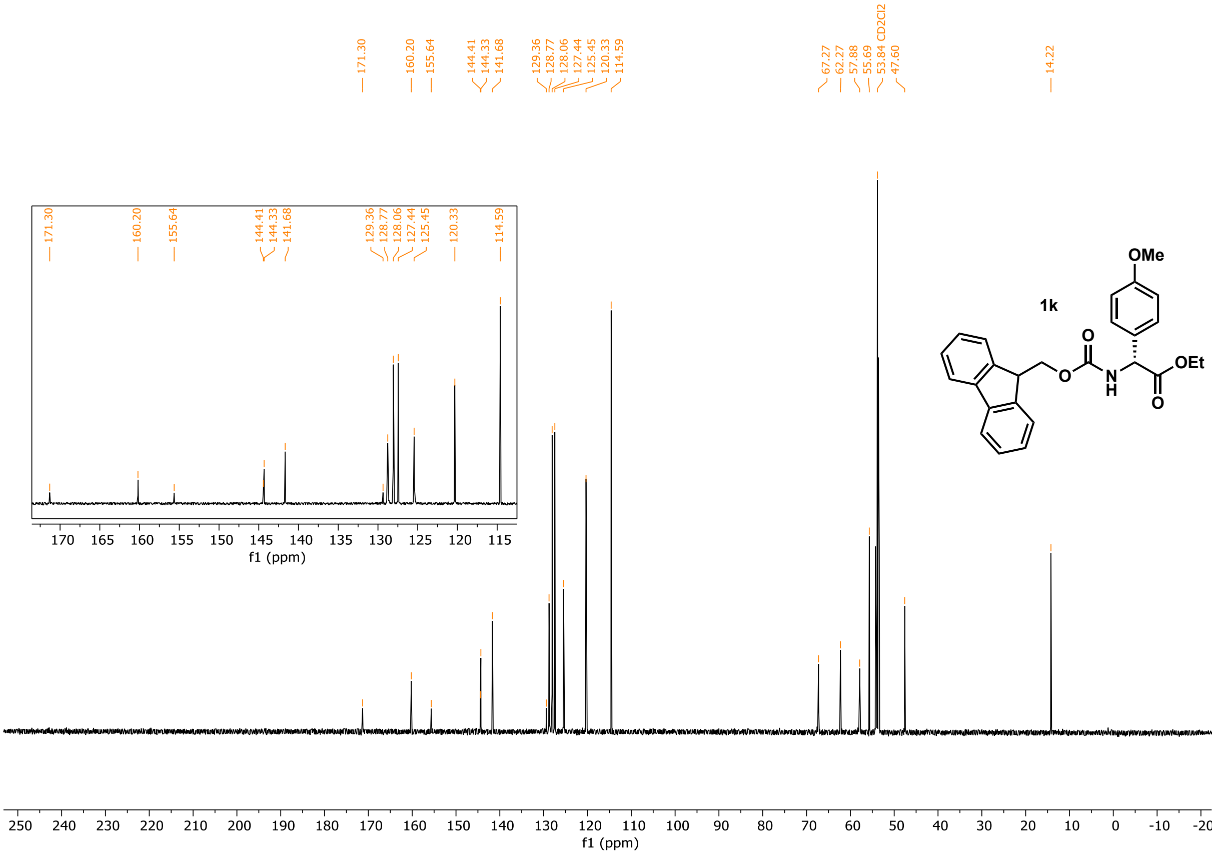

^{13}C-NMR spectrum of compound **1k**.

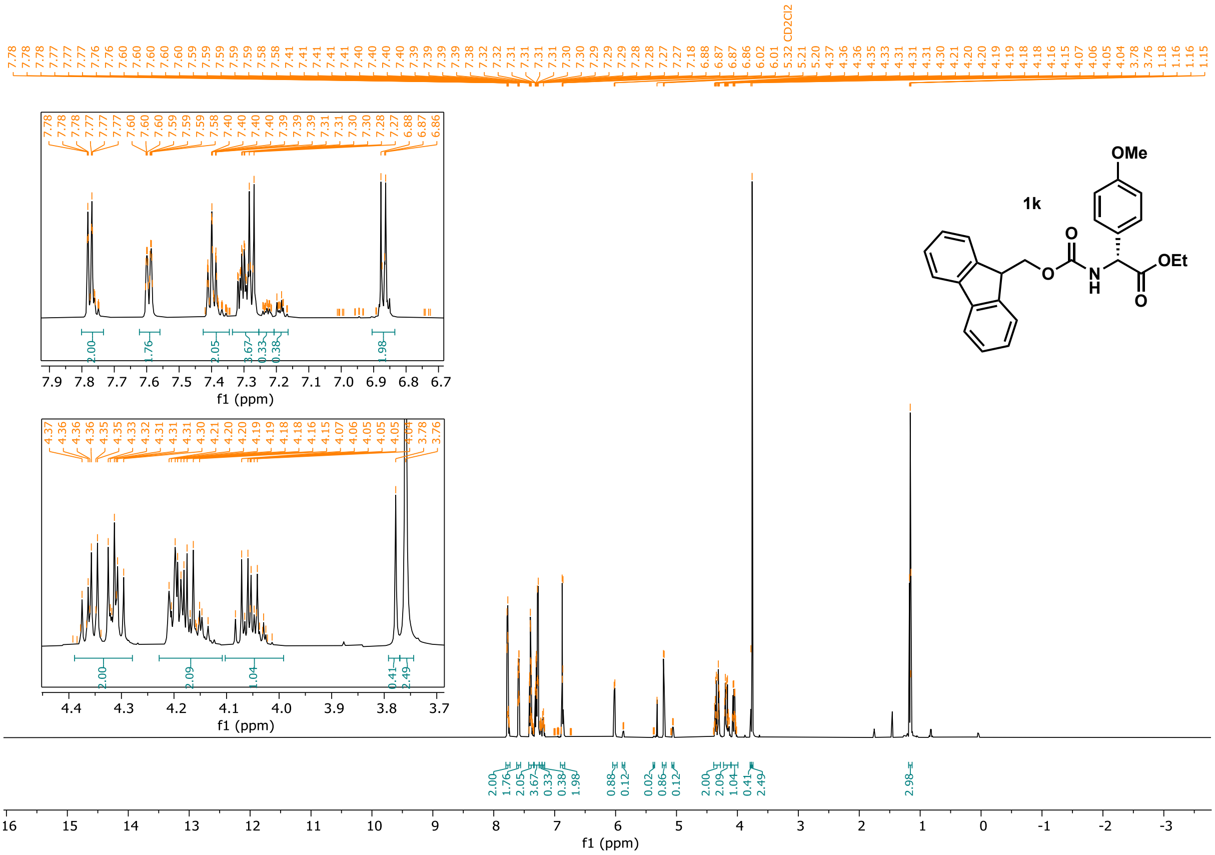

¹H-NMR spectrum of compound **1k**, data collected at 233 K.

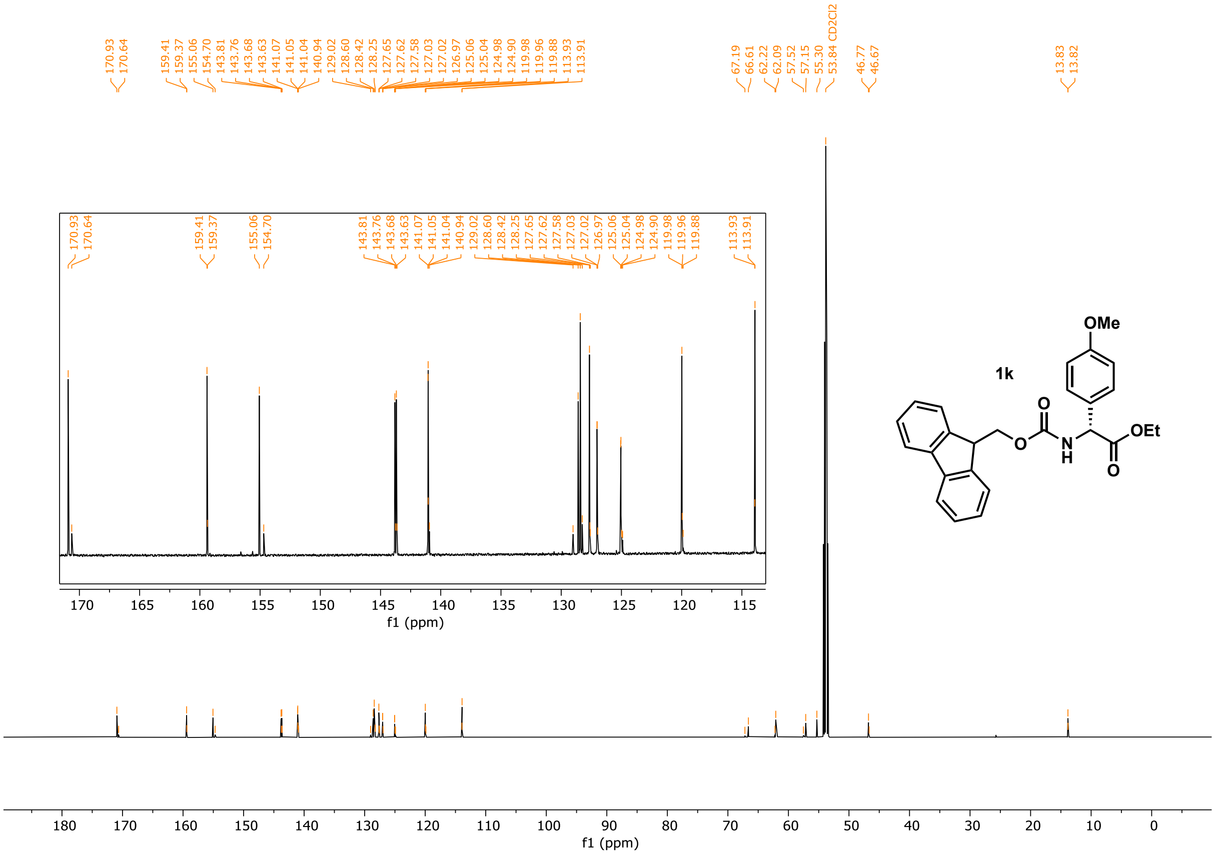

¹³C-NMR spectrum of compound **1k**, data collected at 233 K.

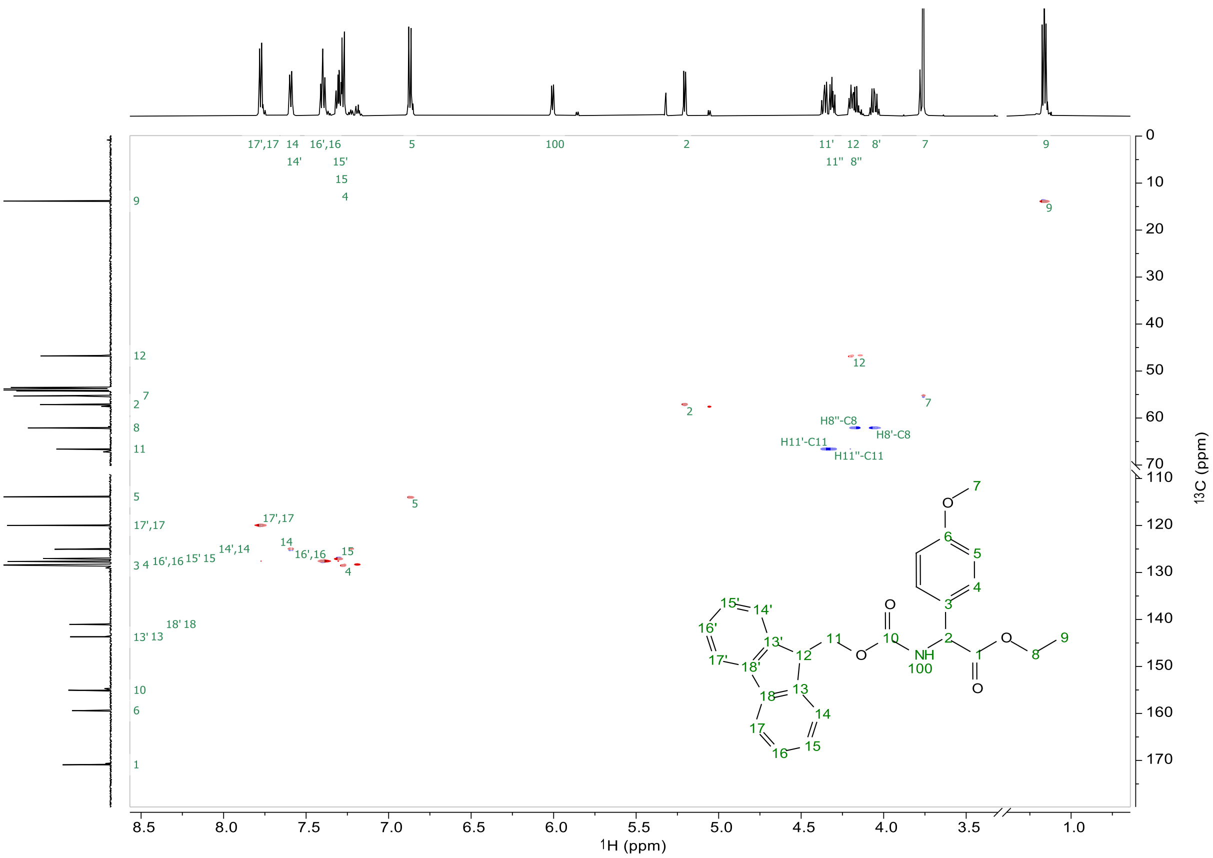

^{1}H{^{13}C}-HSQC spectrum of compound **1k**.

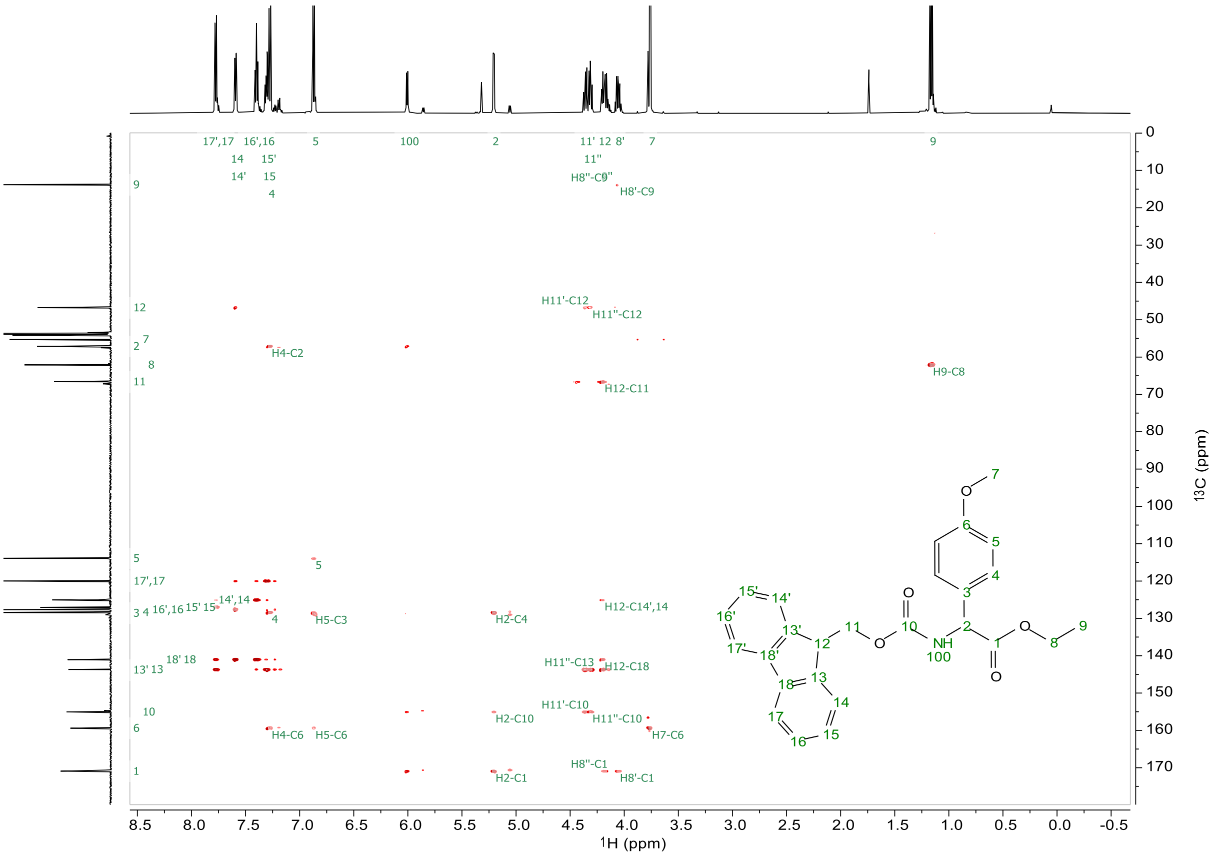

^{1}H{^{13}C}-HMBC spectrum of compound **1k**.

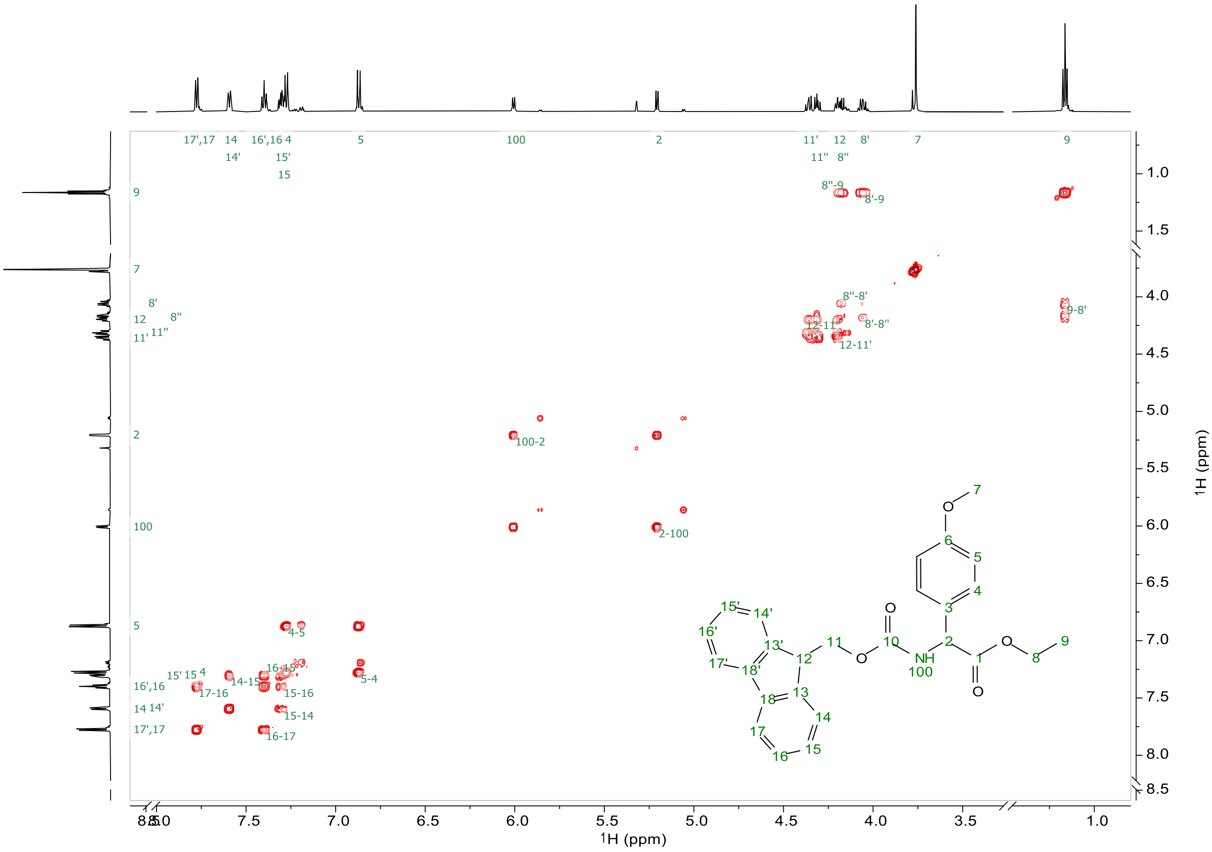

[1]**H-COSY** spectrum of compound **1k**.

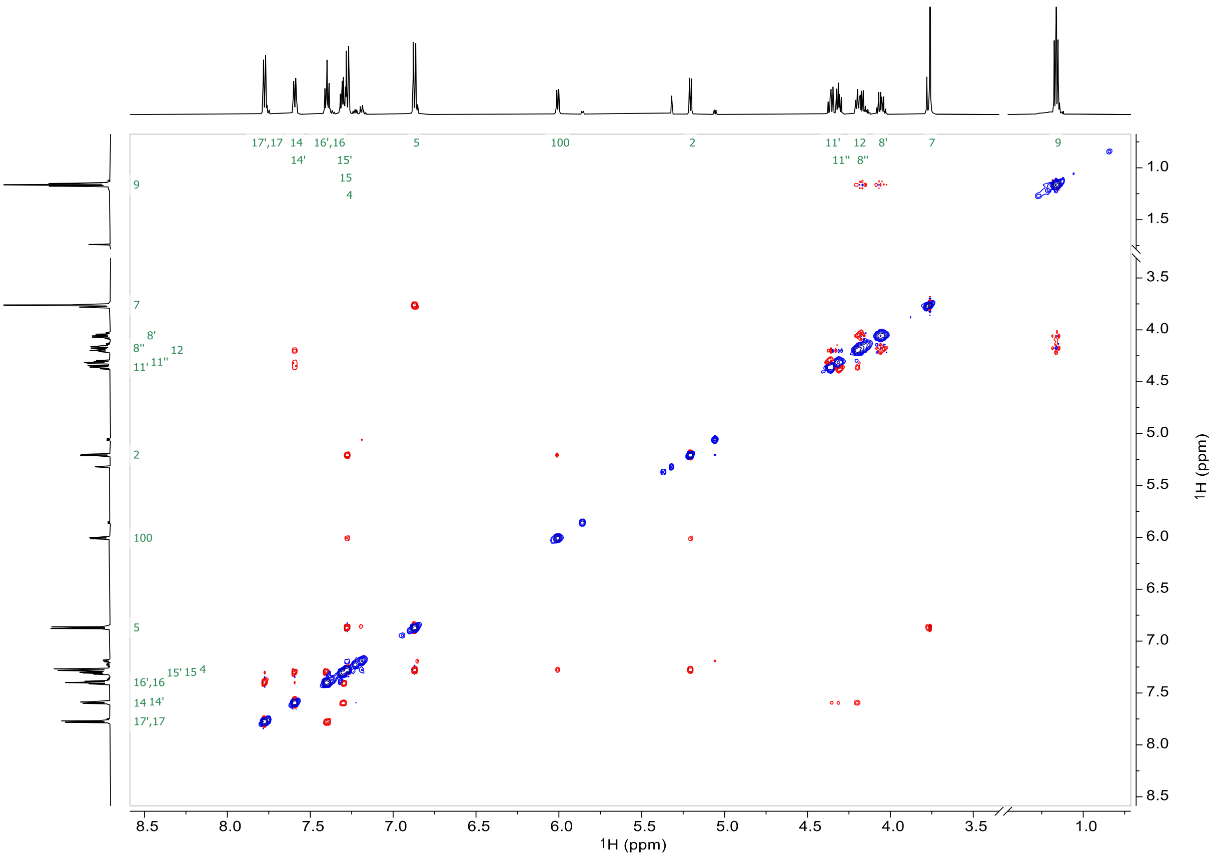

[1]**H-NOESY** spectrum of compound **1k**.

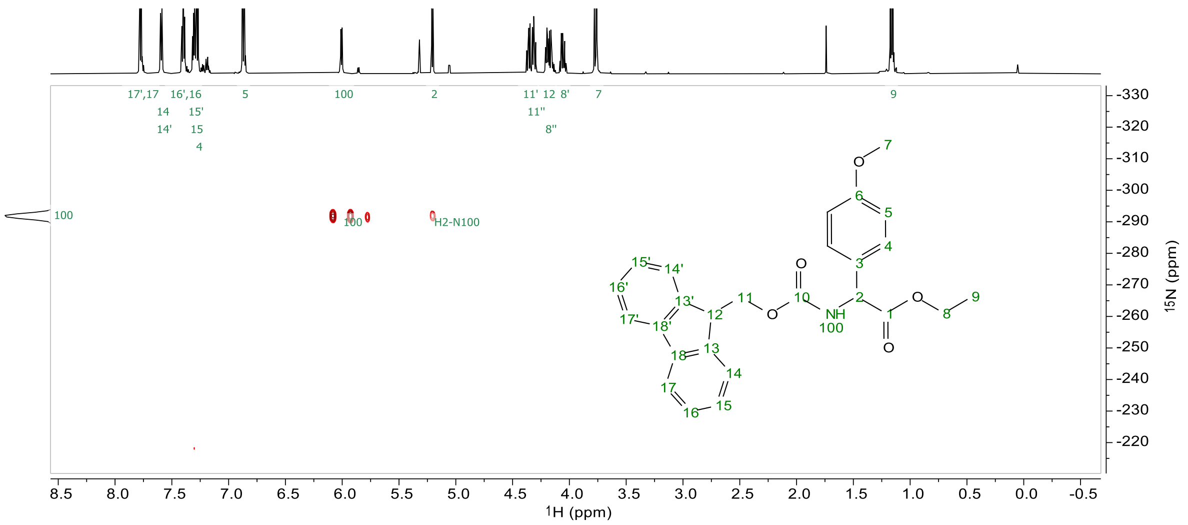

$^1H\{^{15}N\}$-HMBC spectrum of compound **1k**.

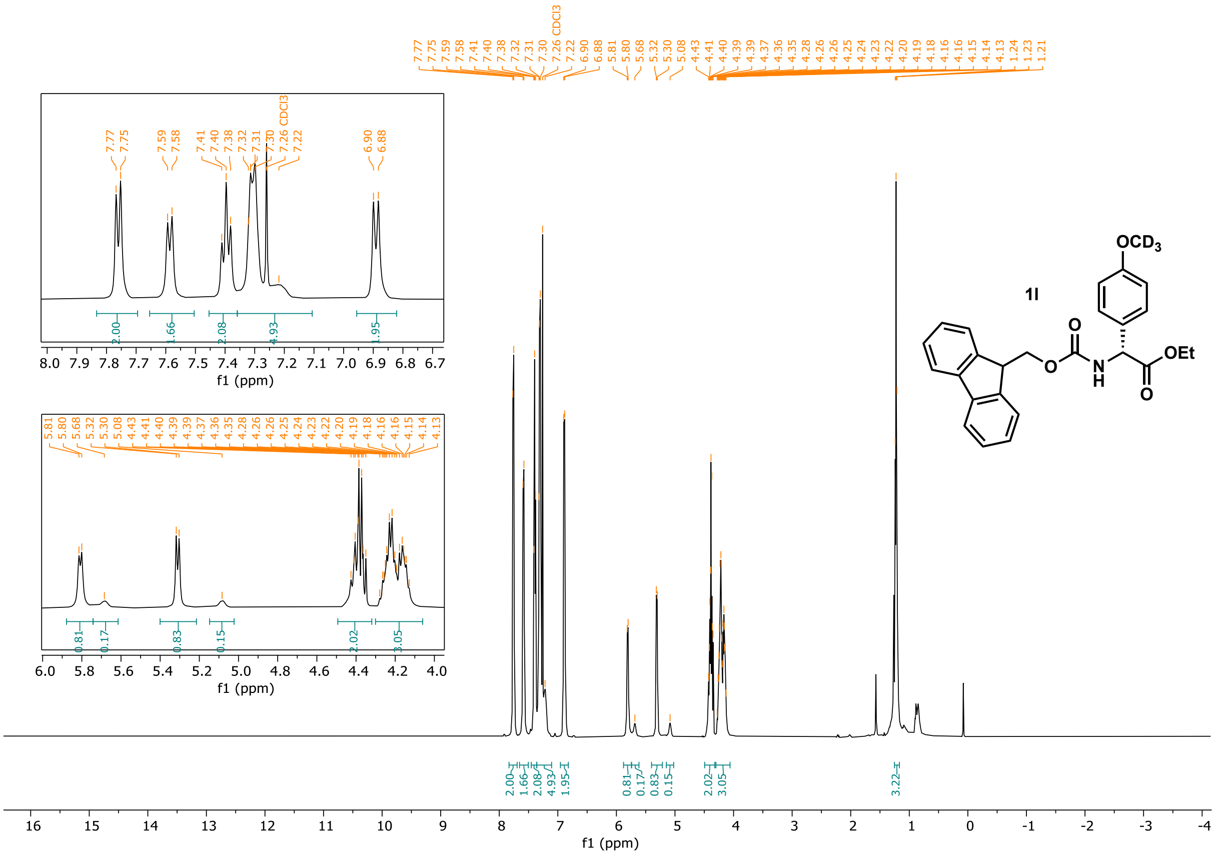

^{1}**H-NMR** spectrum of compound **1l**.

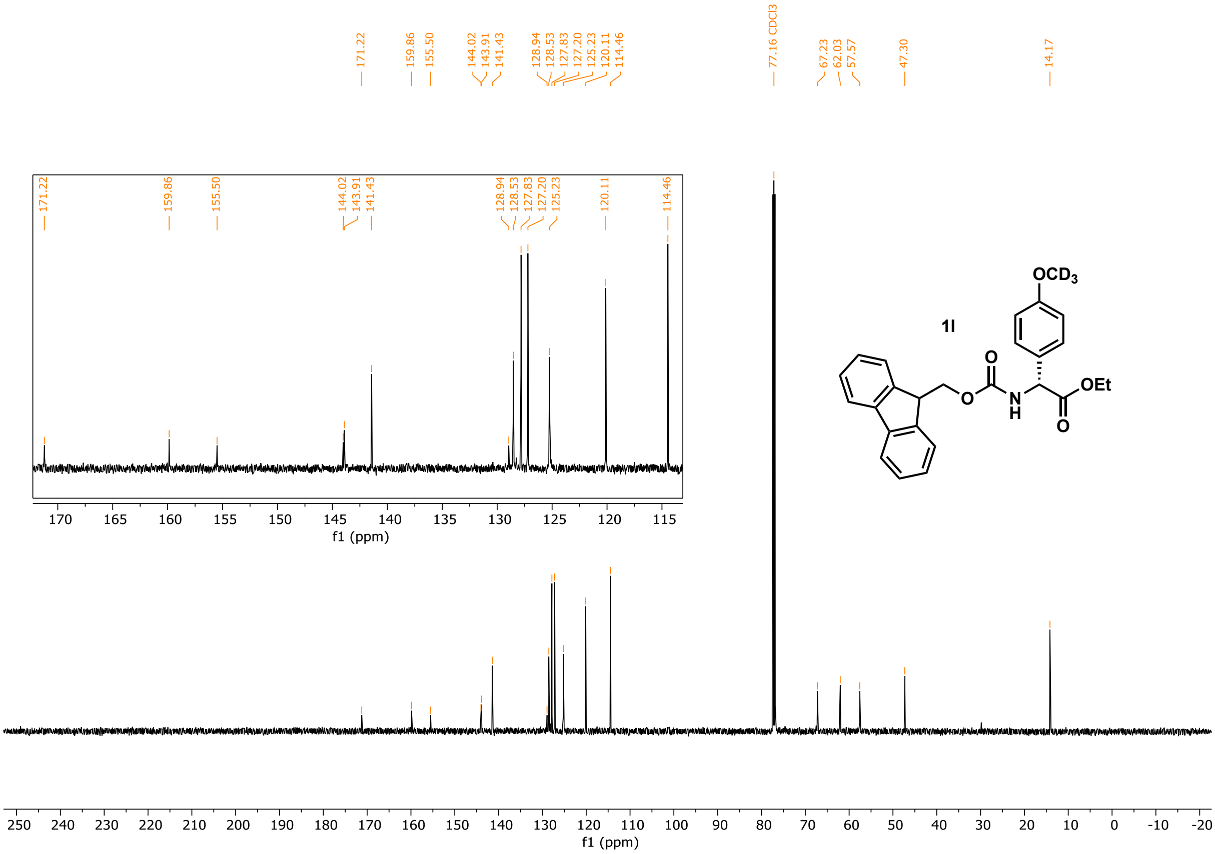

^{13}**C-NMR** spectrum of compound **1l**.

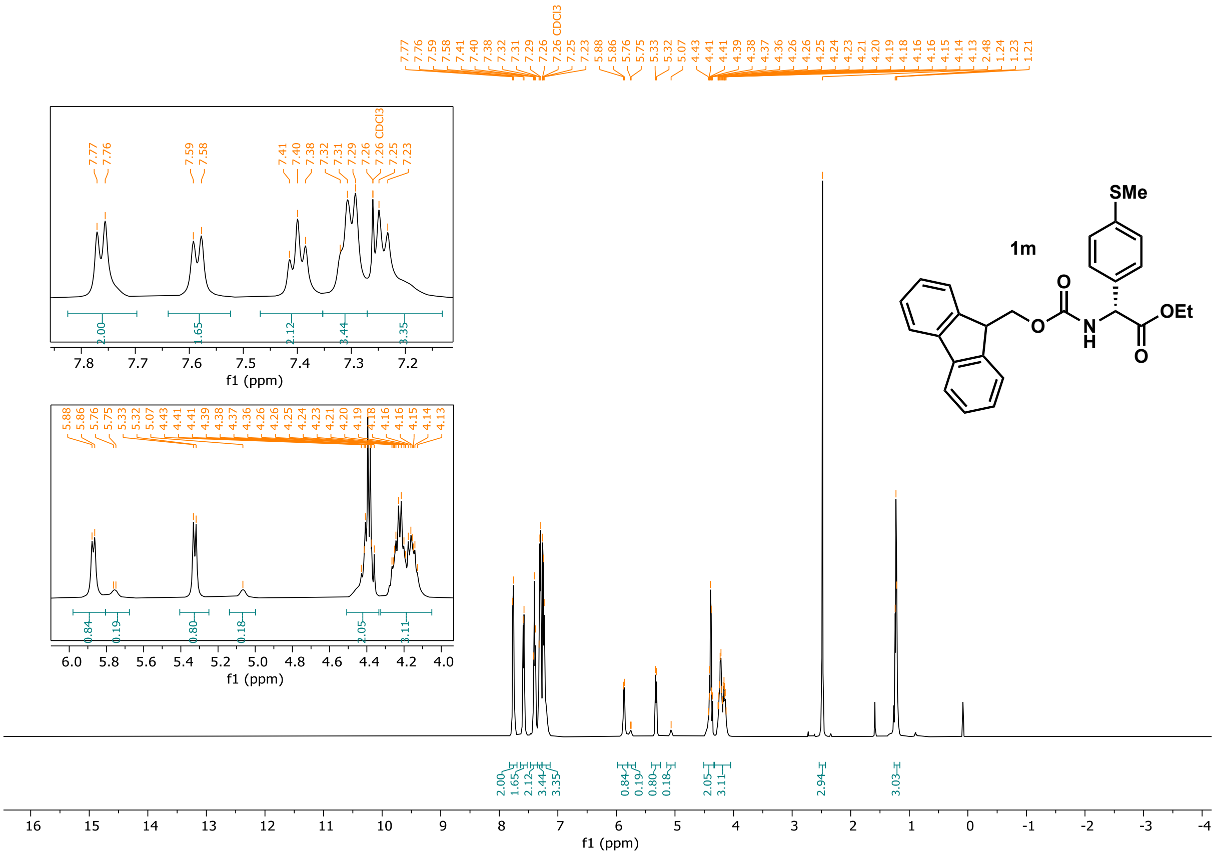

^{1}H-NMR spectrum of compound **1m**.

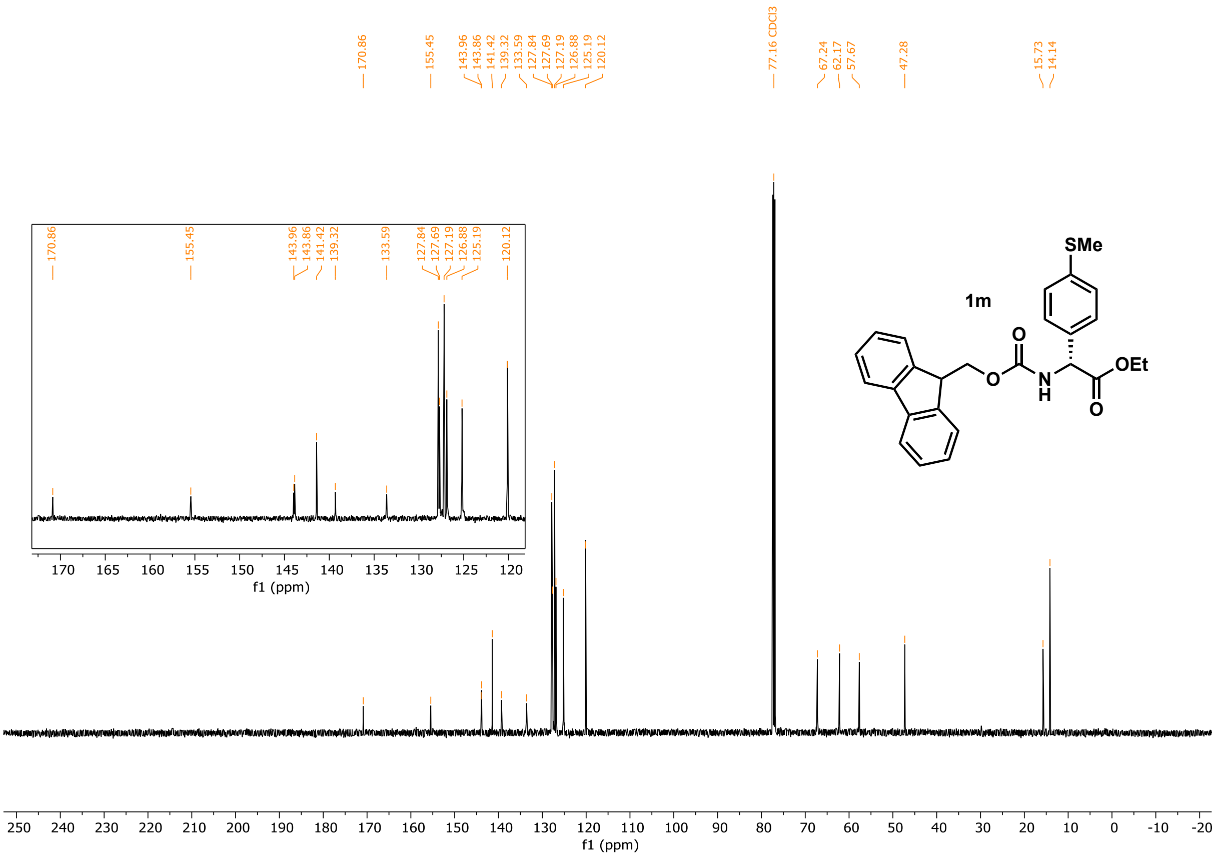

^{13}C-NMR spectrum of compound **1m**.

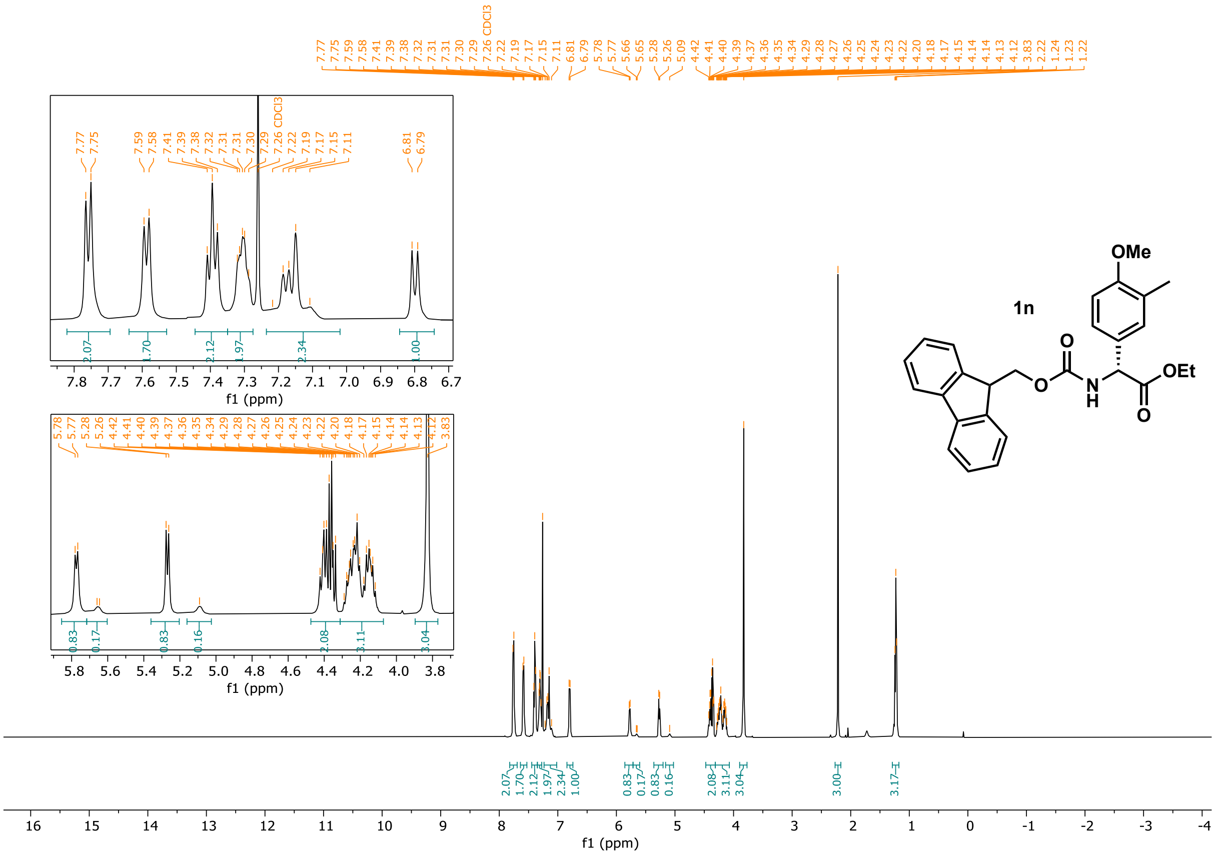

^{1}H-NMR spectrum of compound **1n**.

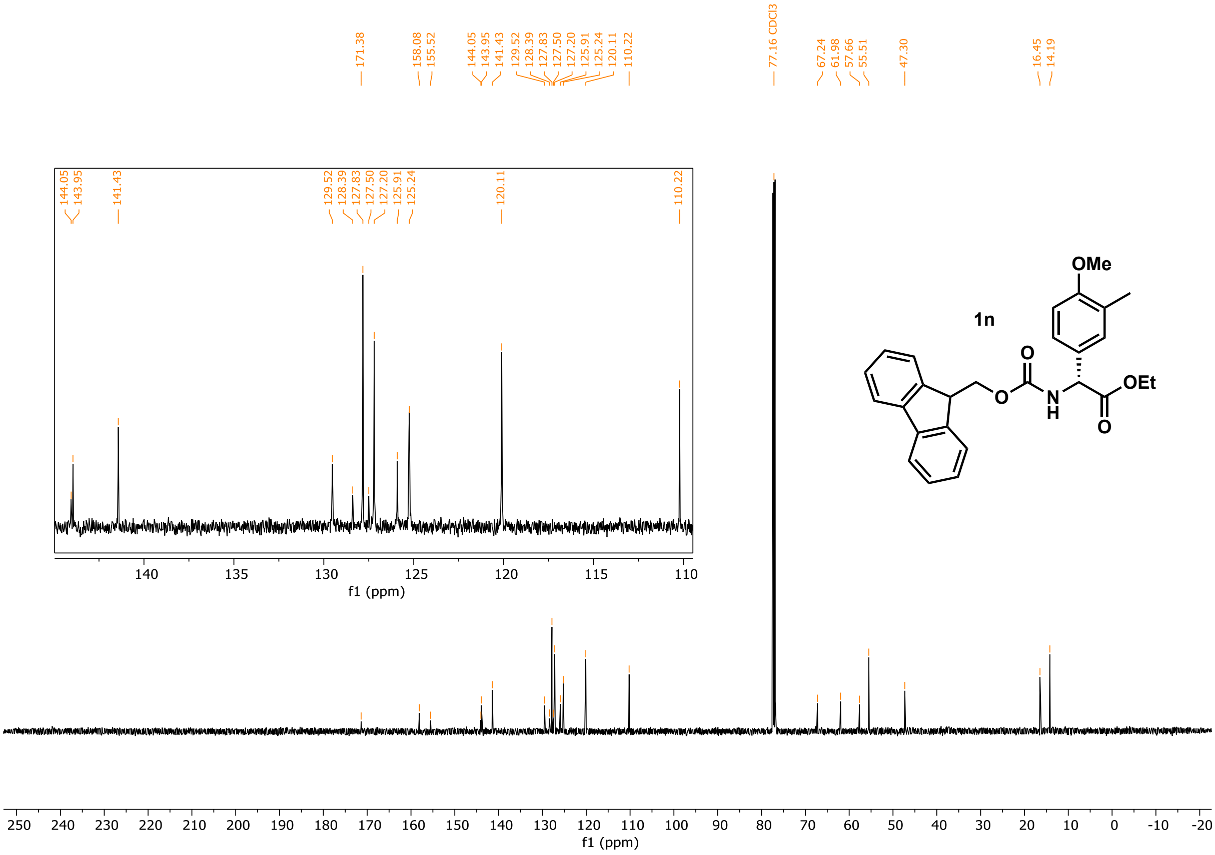

^{13}C-NMR spectrum of compound **1n**.

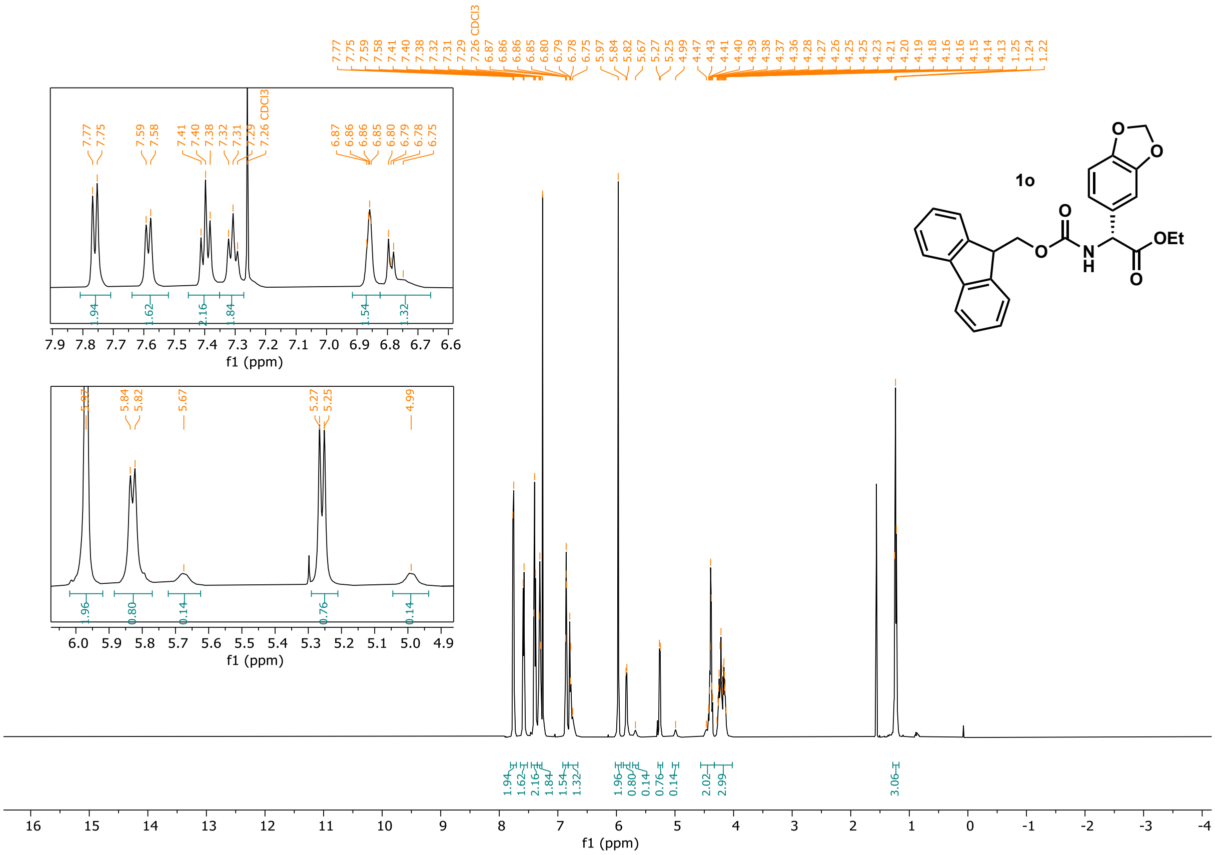

^{1}H-NMR spectrum of compound **1o**.

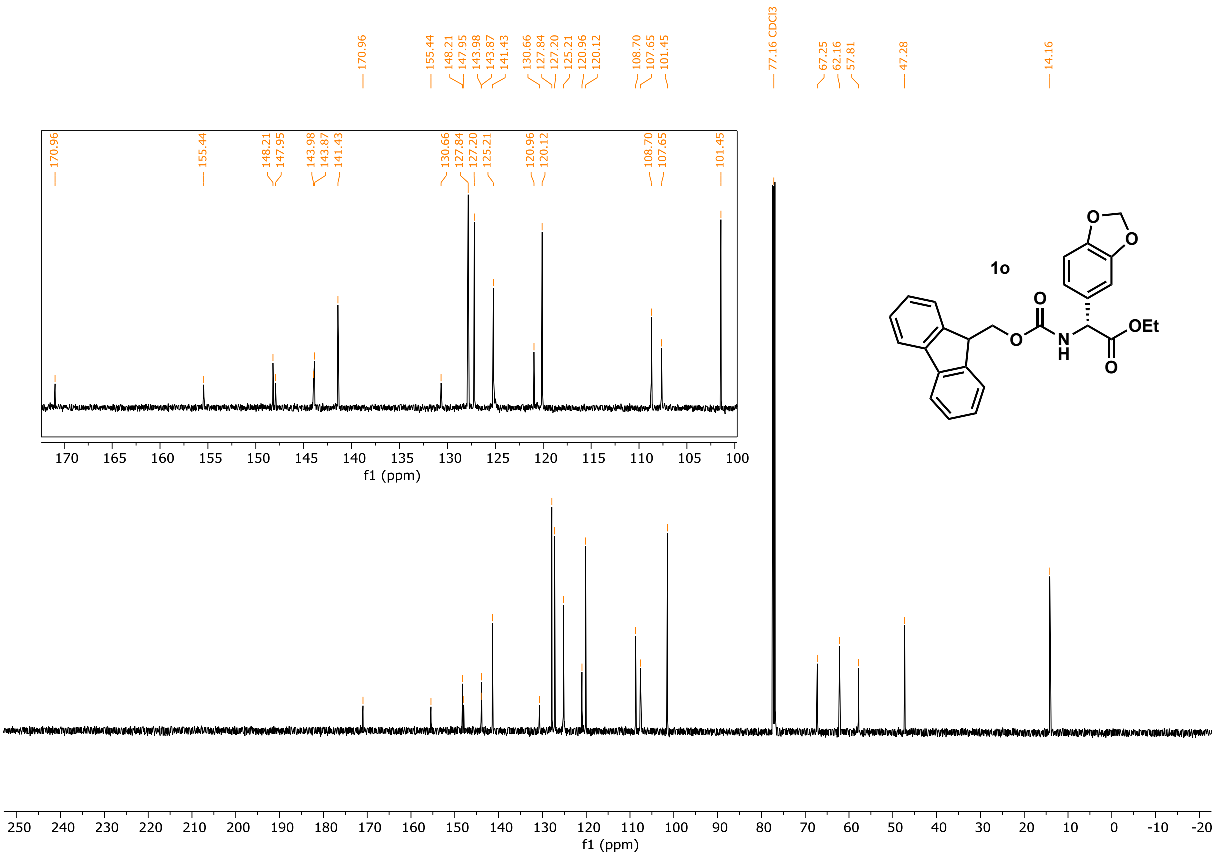

^{13}C-NMR spectrum of compound **1o**.

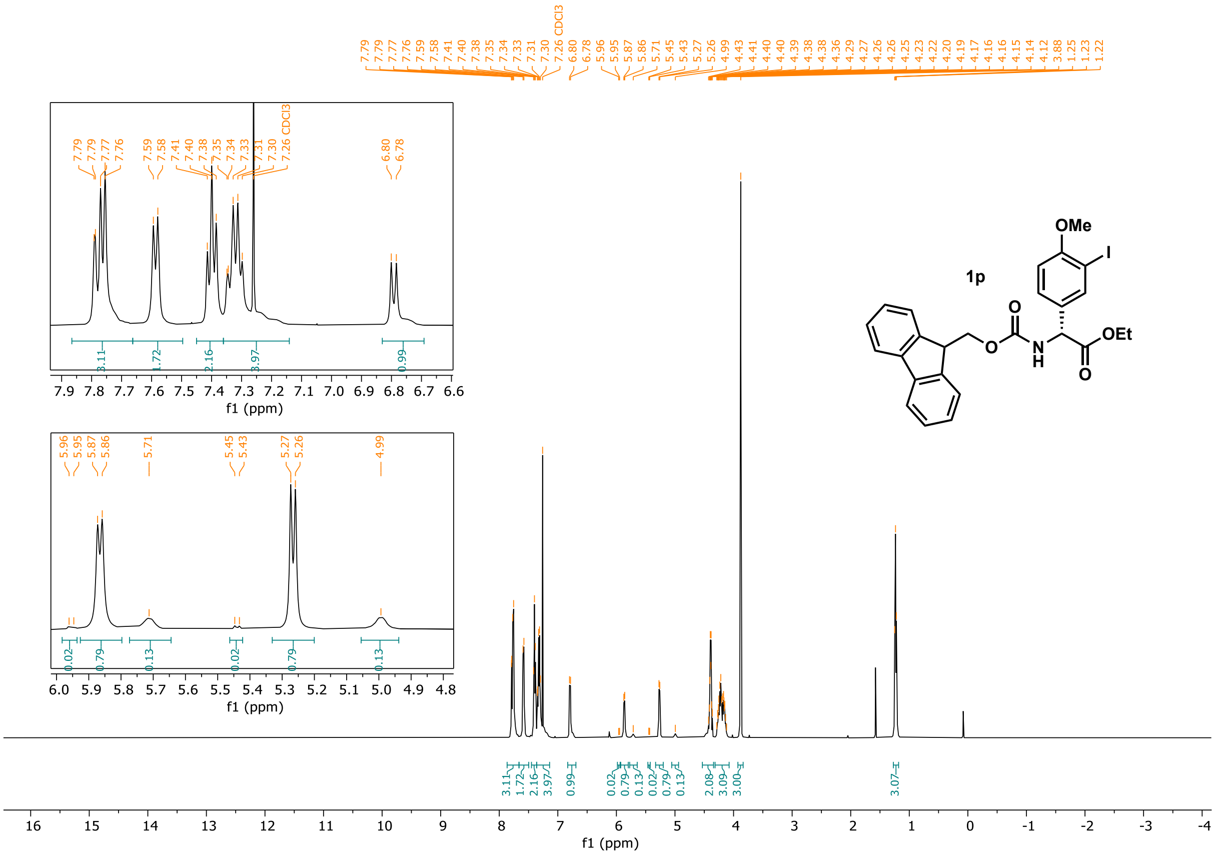

^{1}**H-NMR** spectrum of compound **1p**.

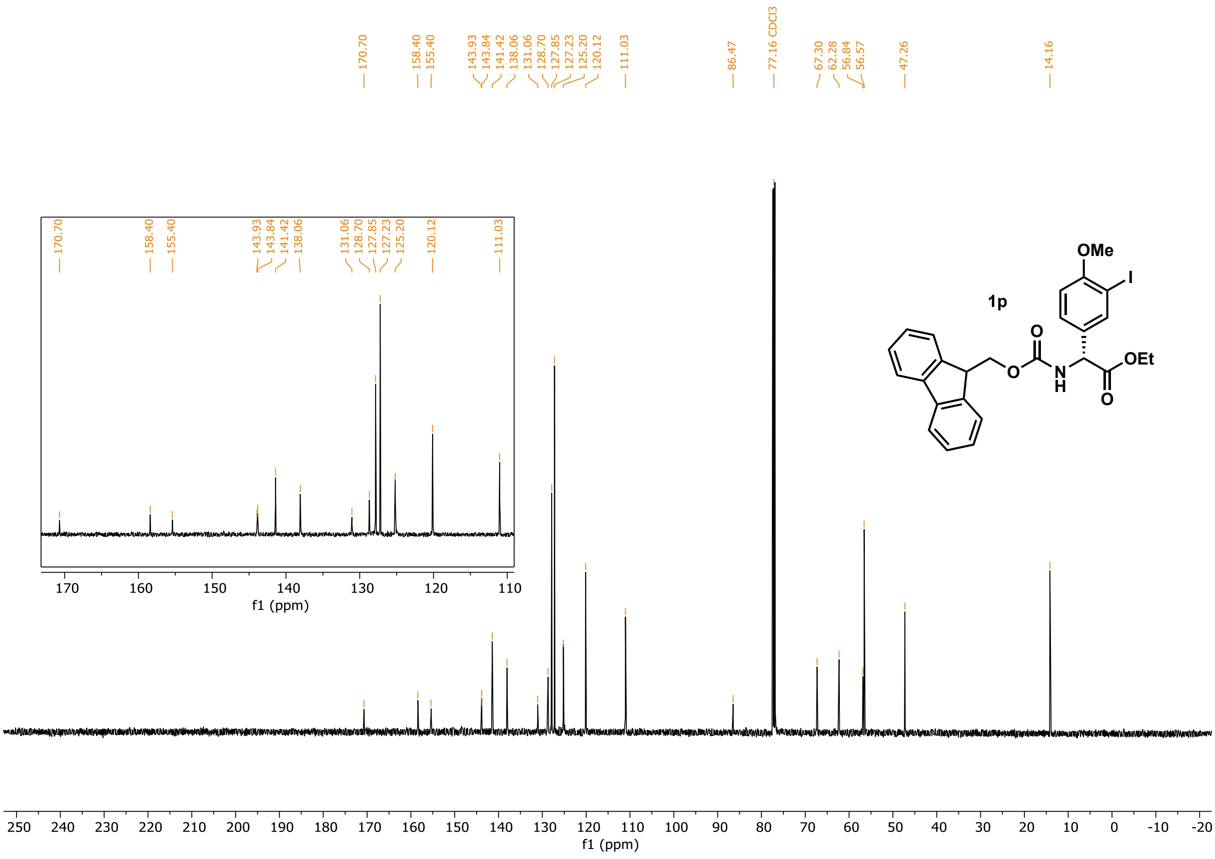

^{13}**C-NMR** spectrum of compound **1p**.

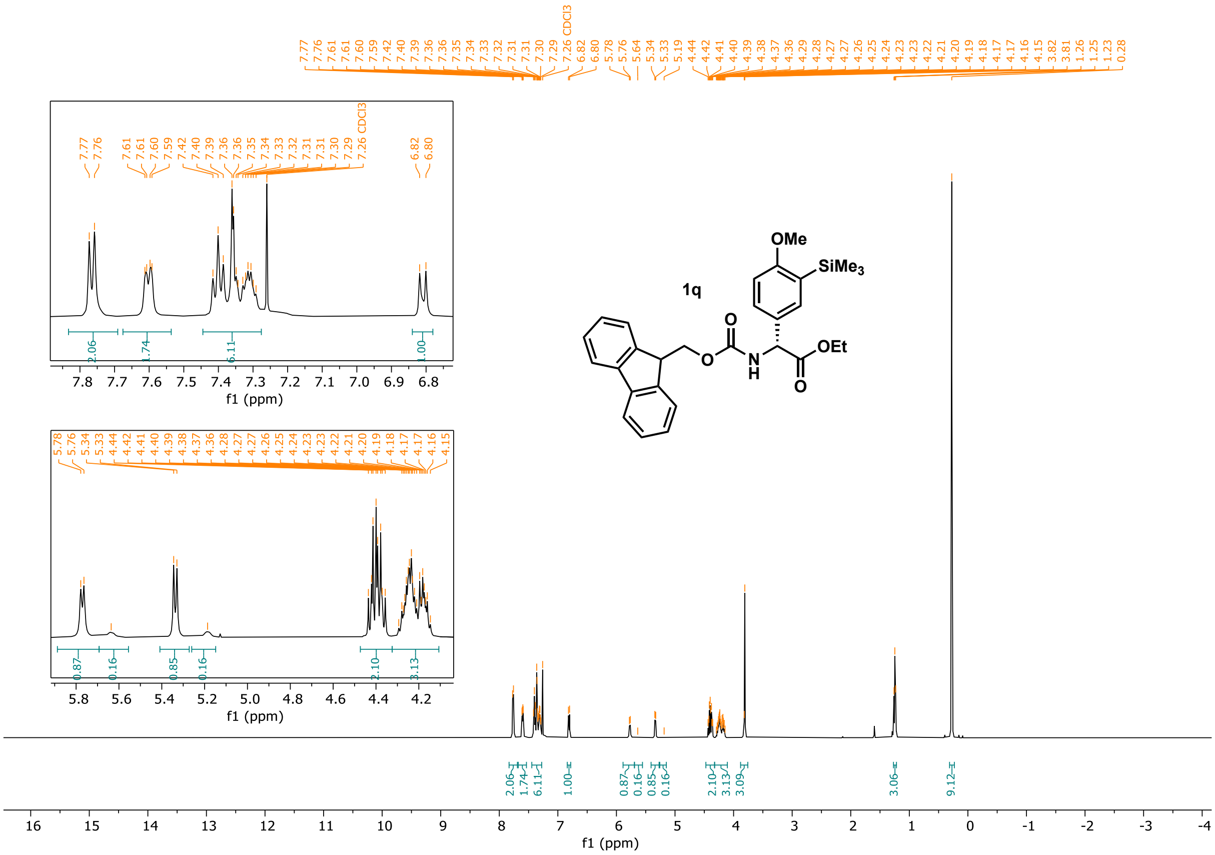

^{1}H-NMR spectrum of compound **1q**.

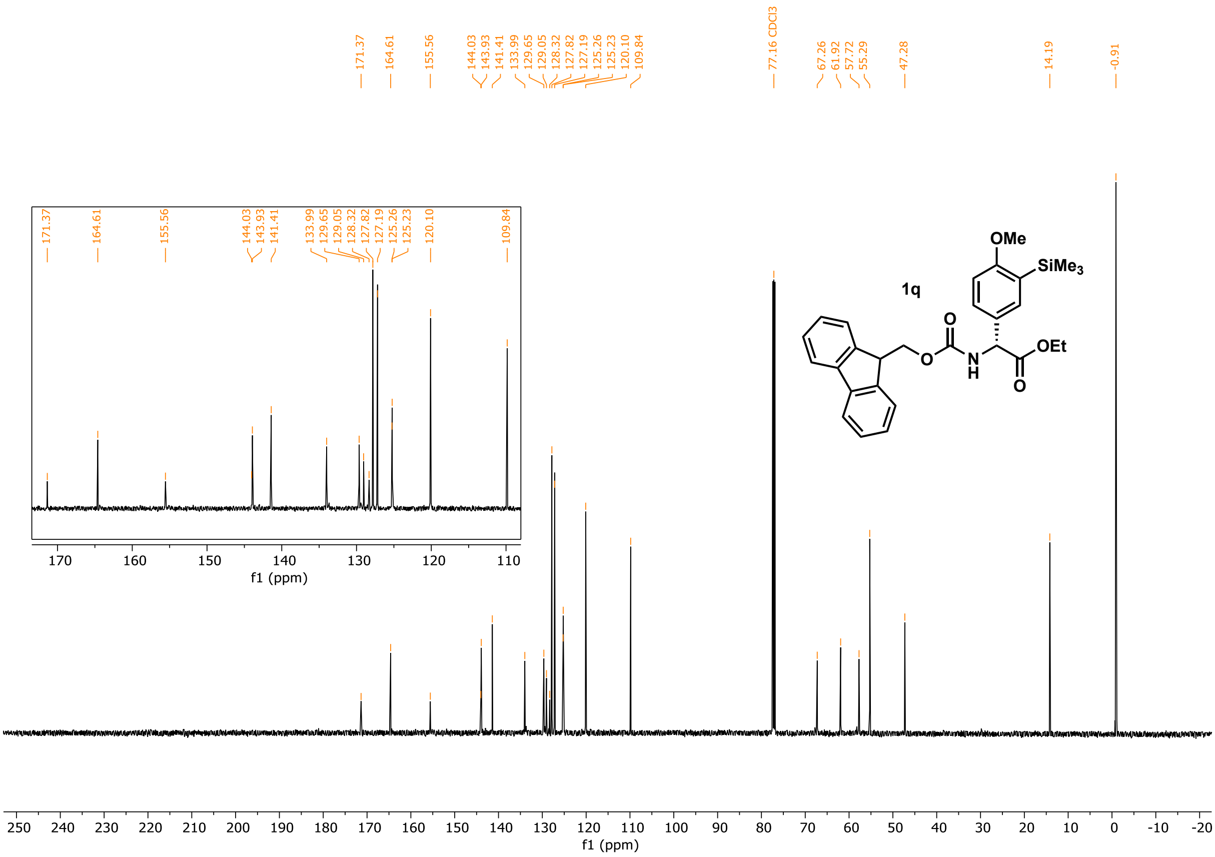

^{13}C-NMR spectrum of compound **1q**.

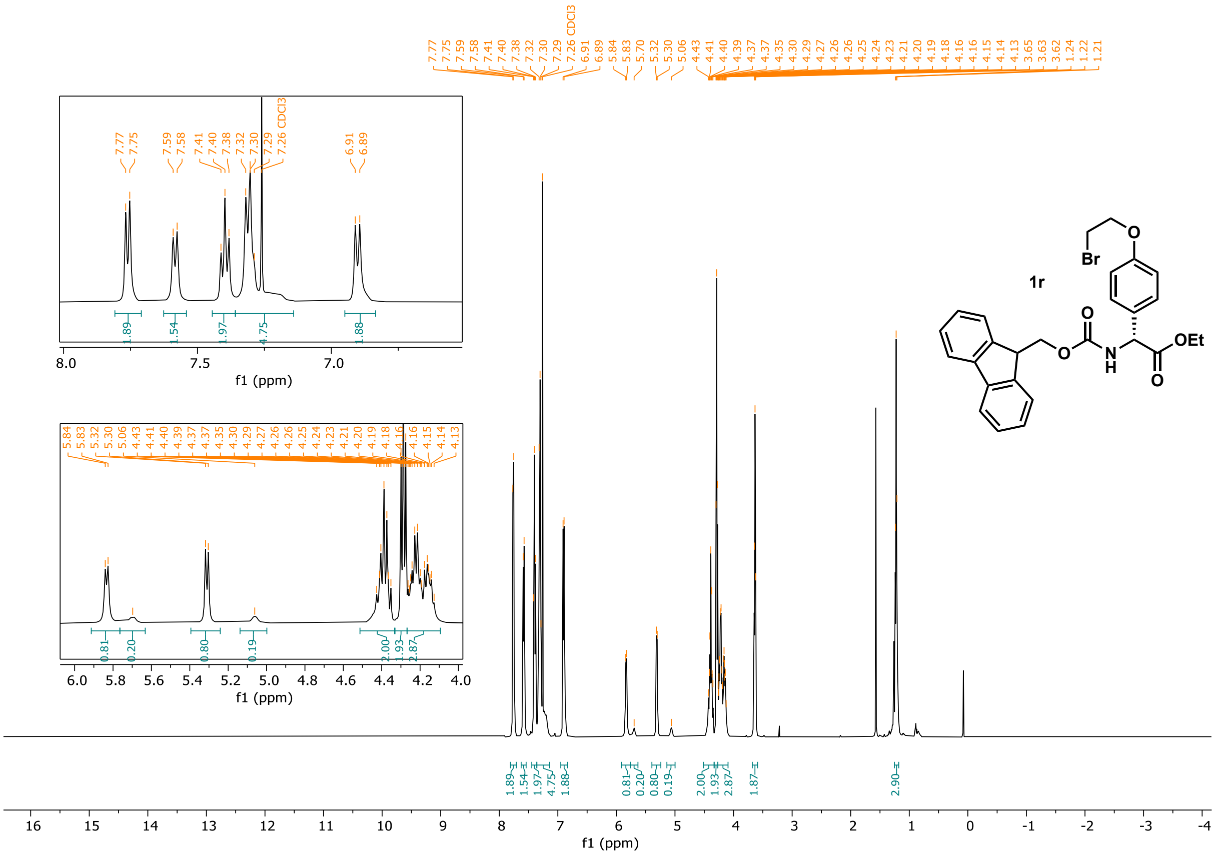

^{1}H-NMR spectrum of compound **1r**.

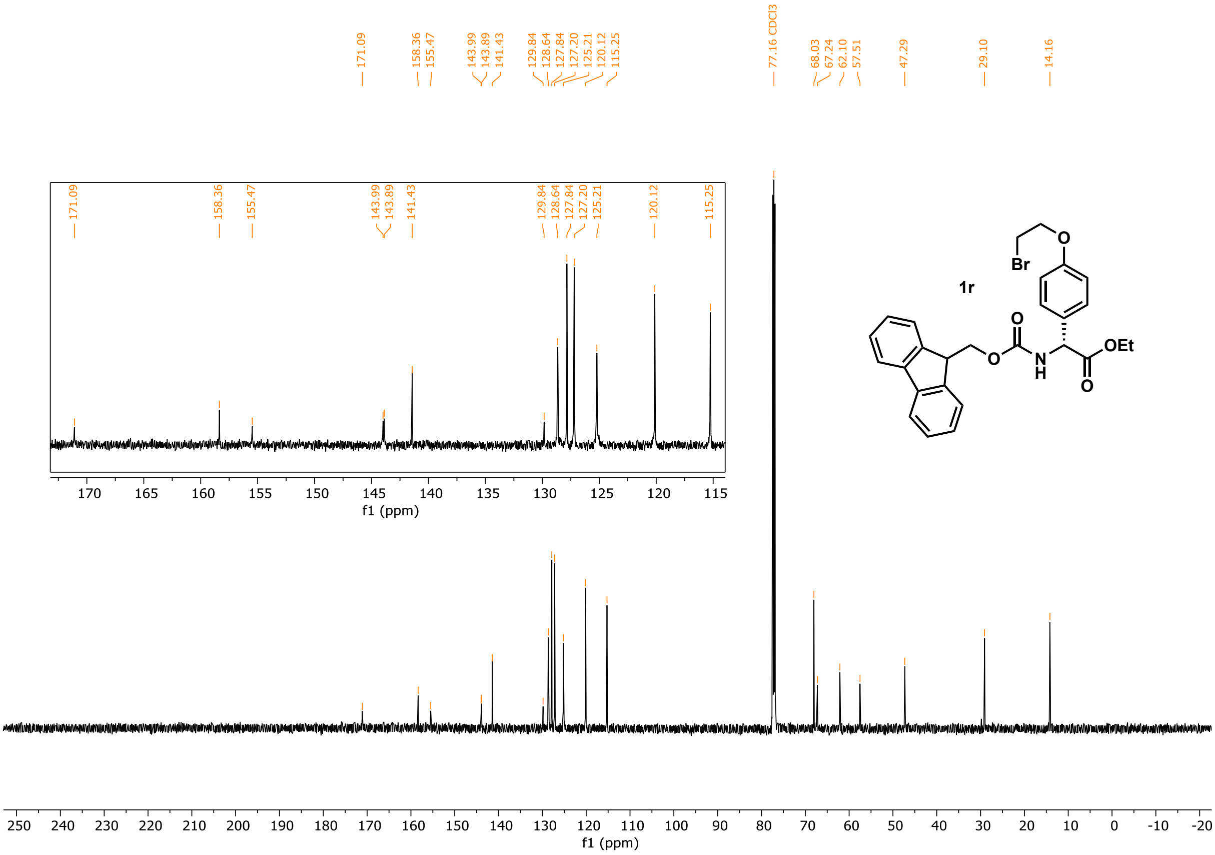

^{13}C-NMR spectrum of compound **1r**.

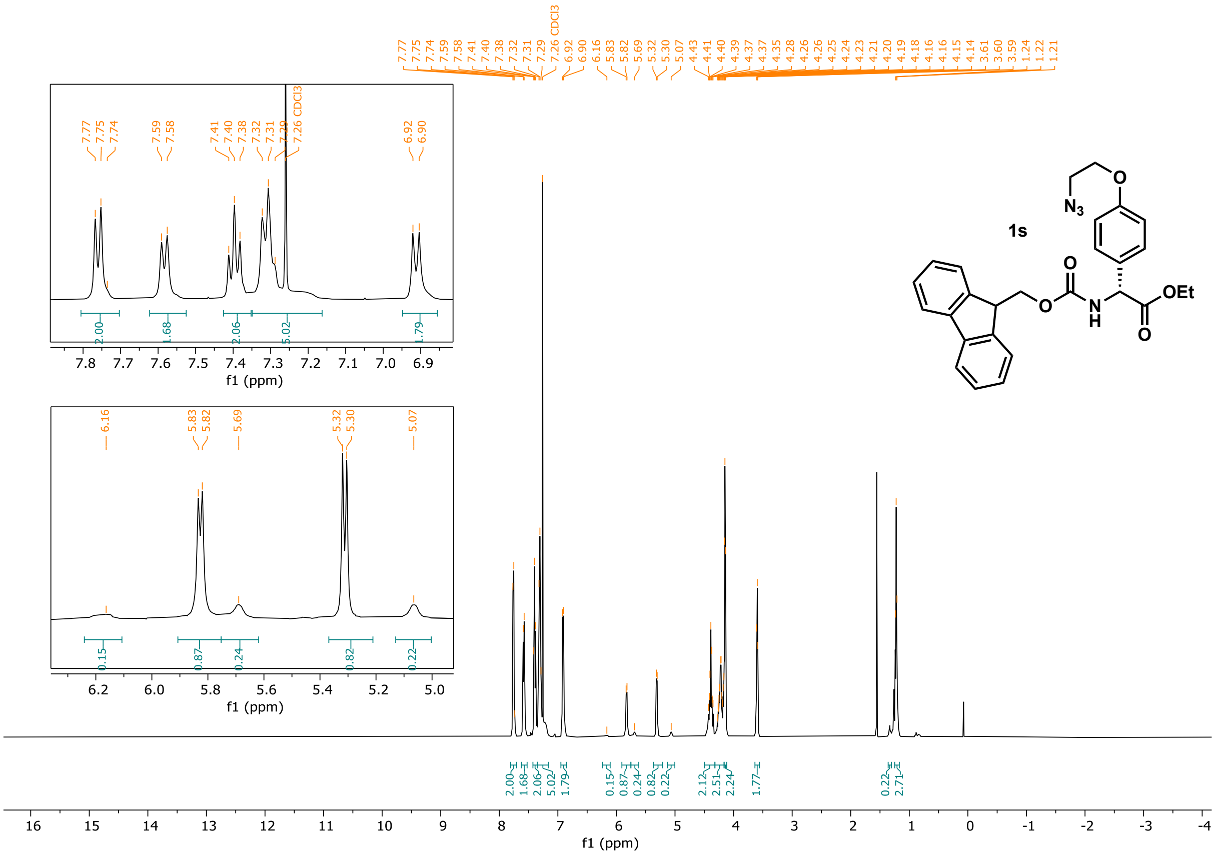

^{1}**H-NMR** spectrum of compound **1s**.

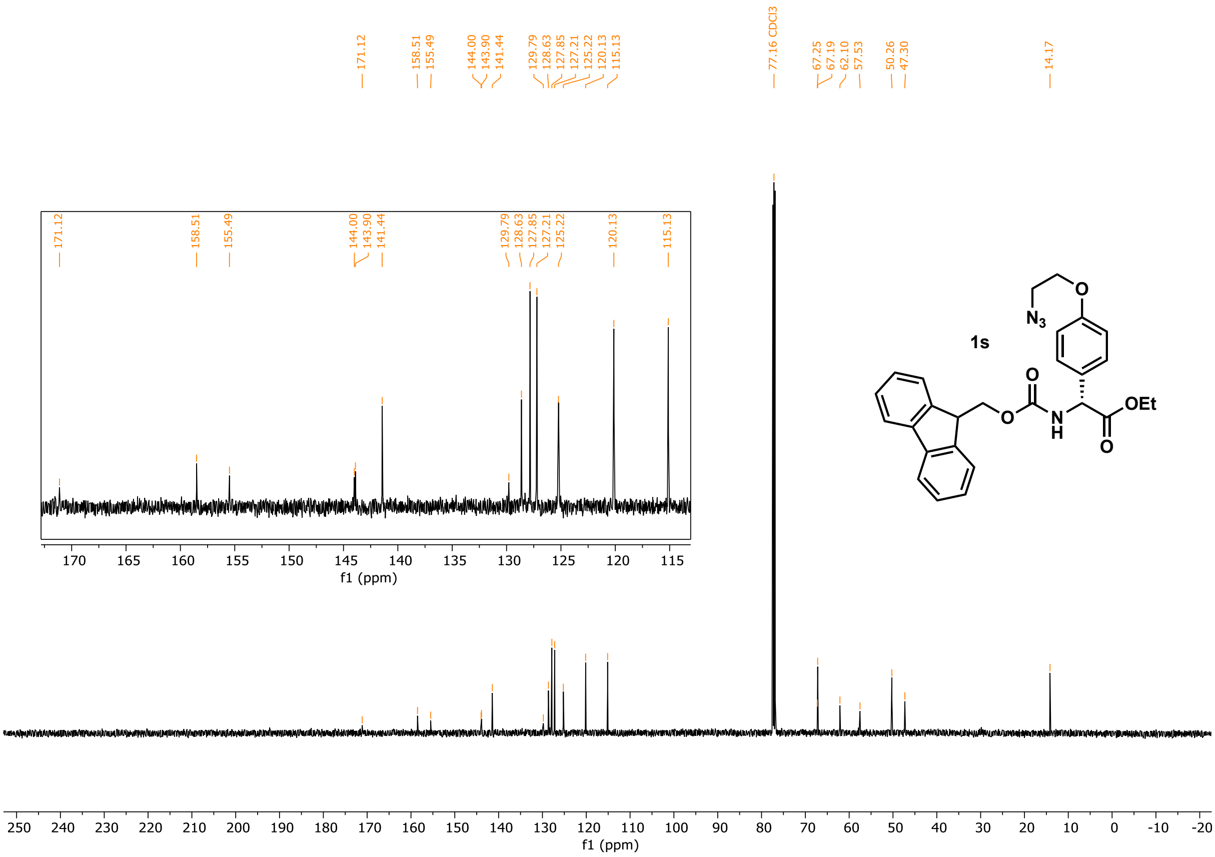

^{13}**C-NMR** spectrum of compound **1s**.

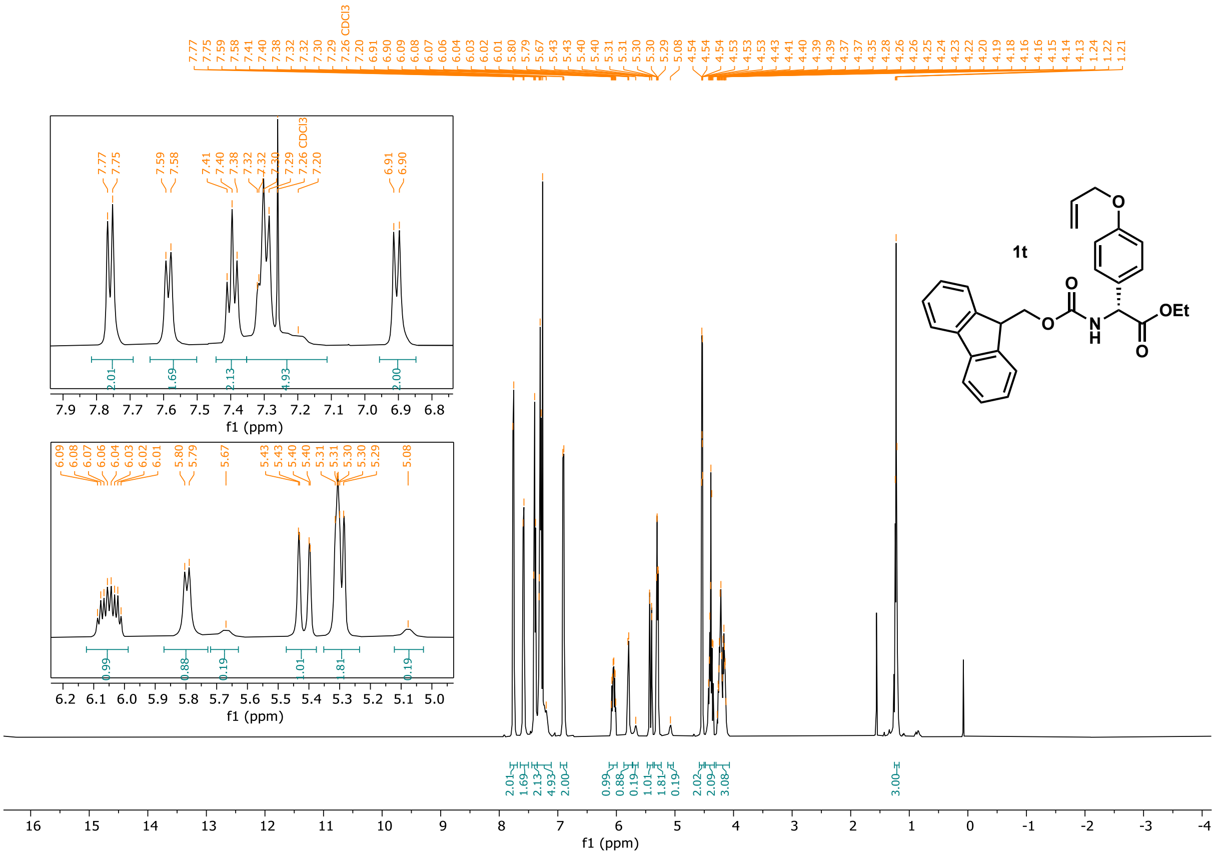

^{1}H-NMR spectrum of compound **1t**.

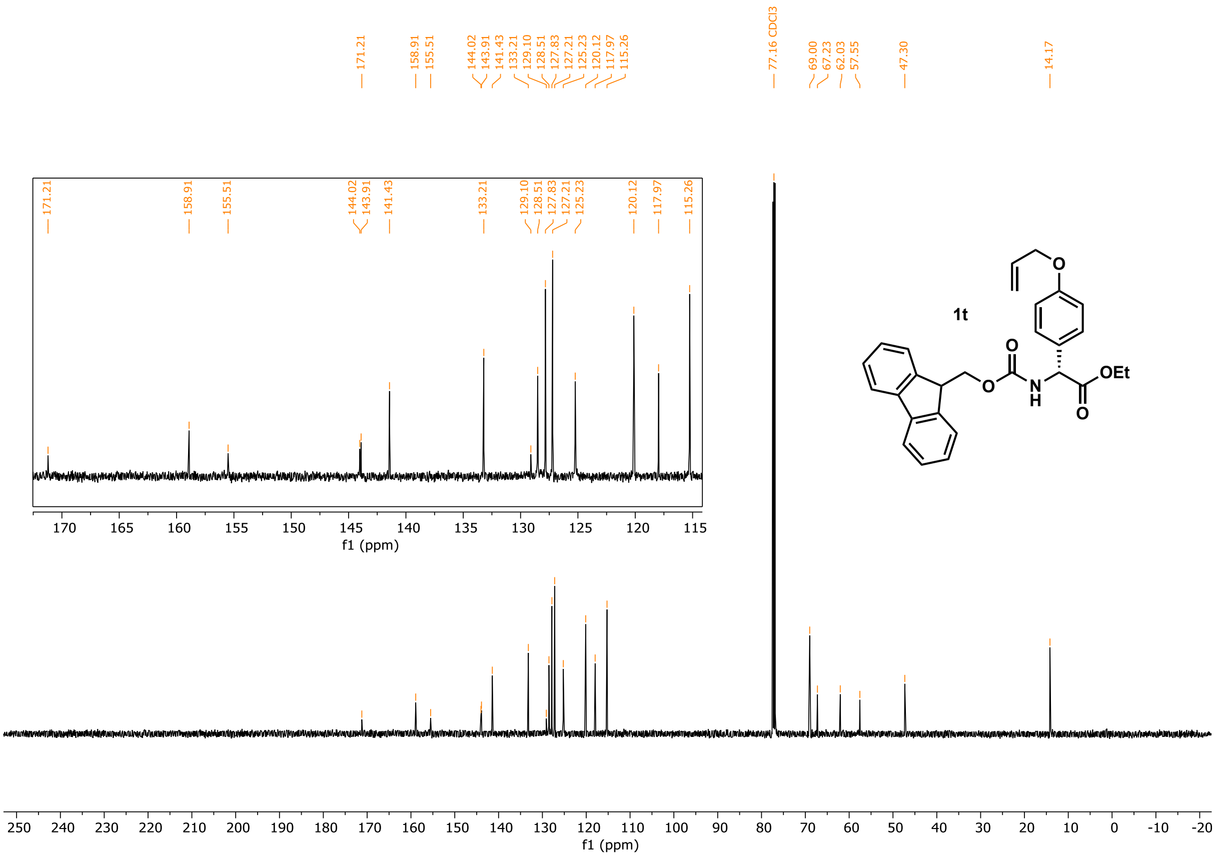

^{13}C-NMR spectrum of compound **1t**.

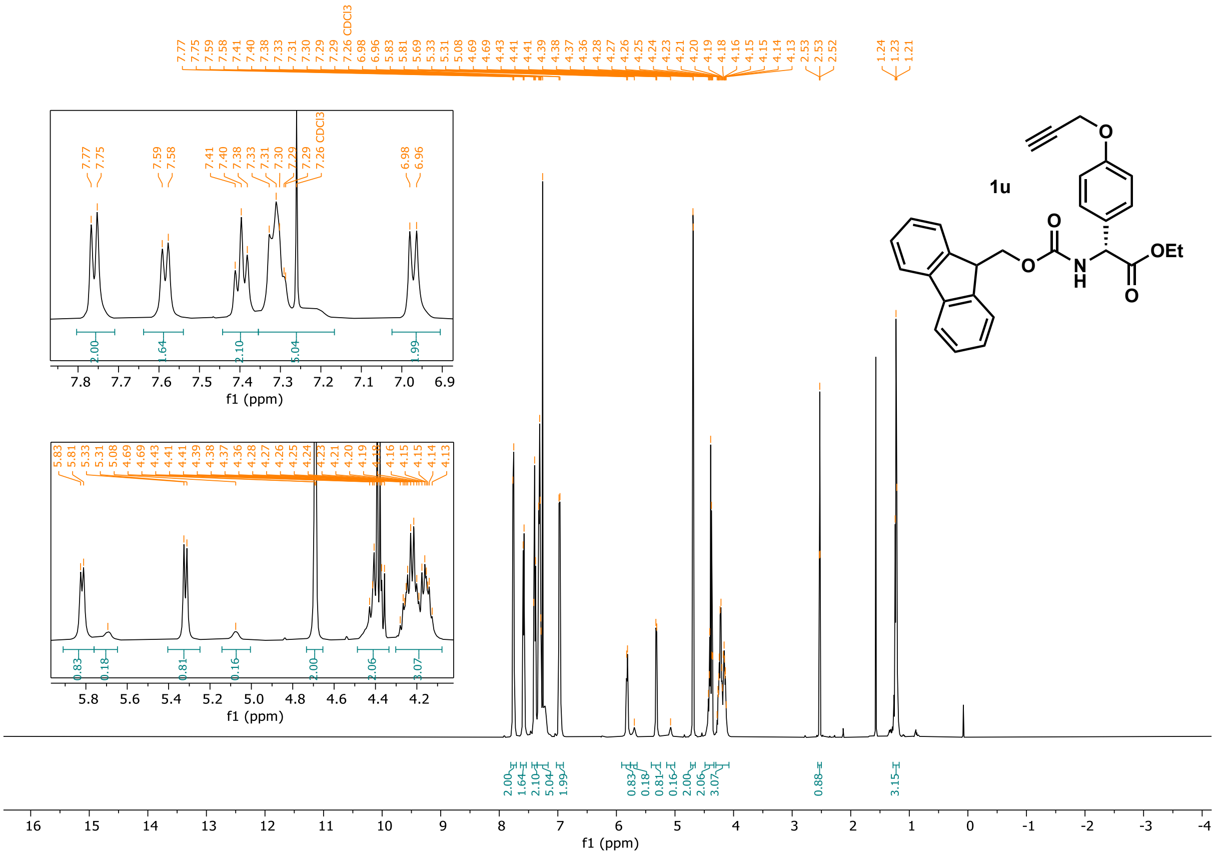

[1]**H-NMR** spectrum of compound **1u**.

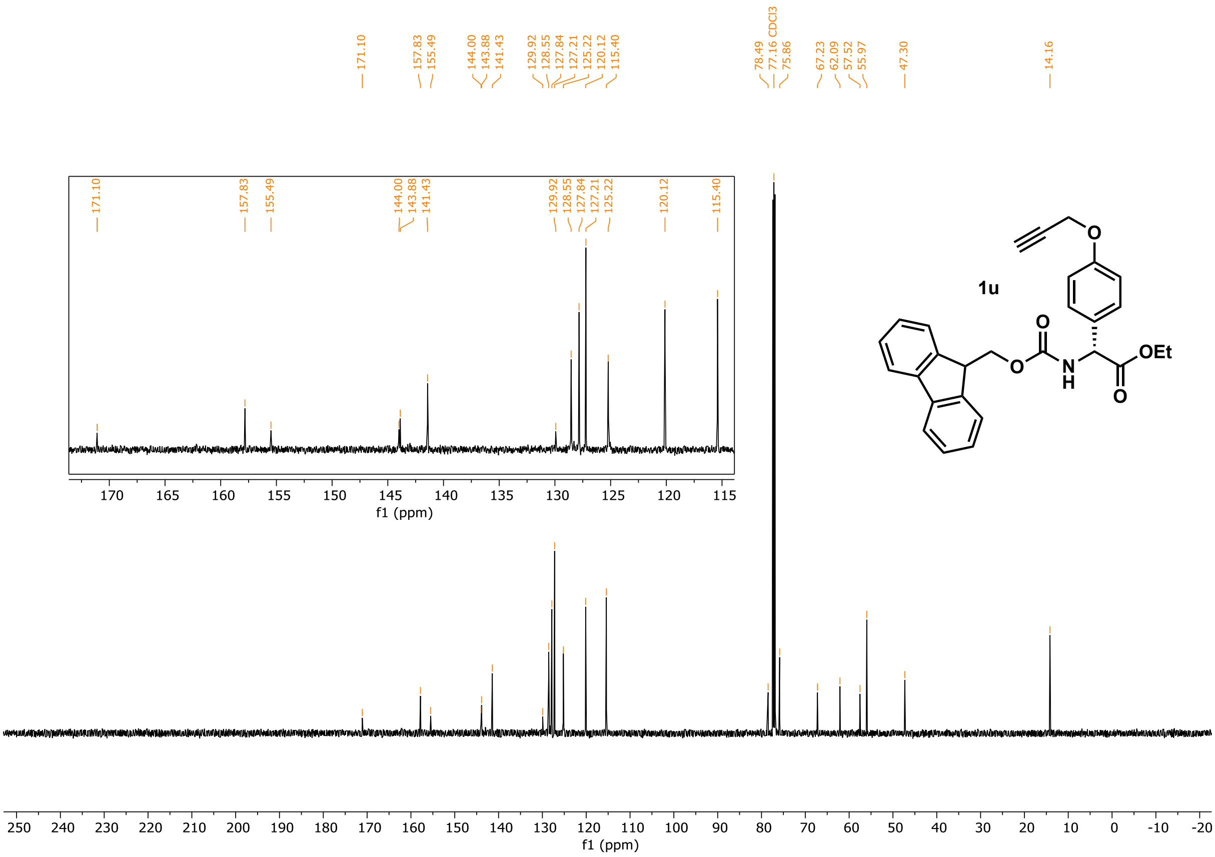

[13]**C-NMR** spectrum of compound **1u**.

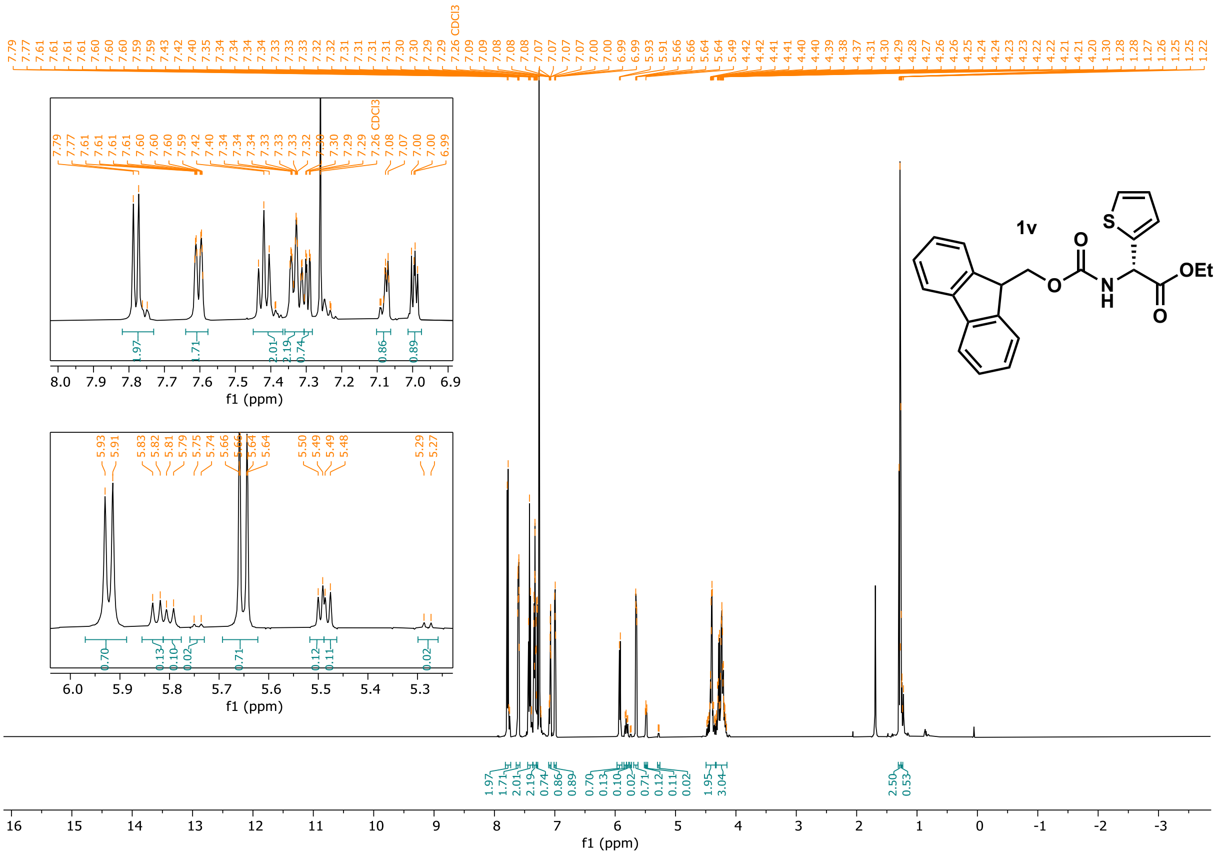

[1]**H-NMR** spectrum of compound **1v**.

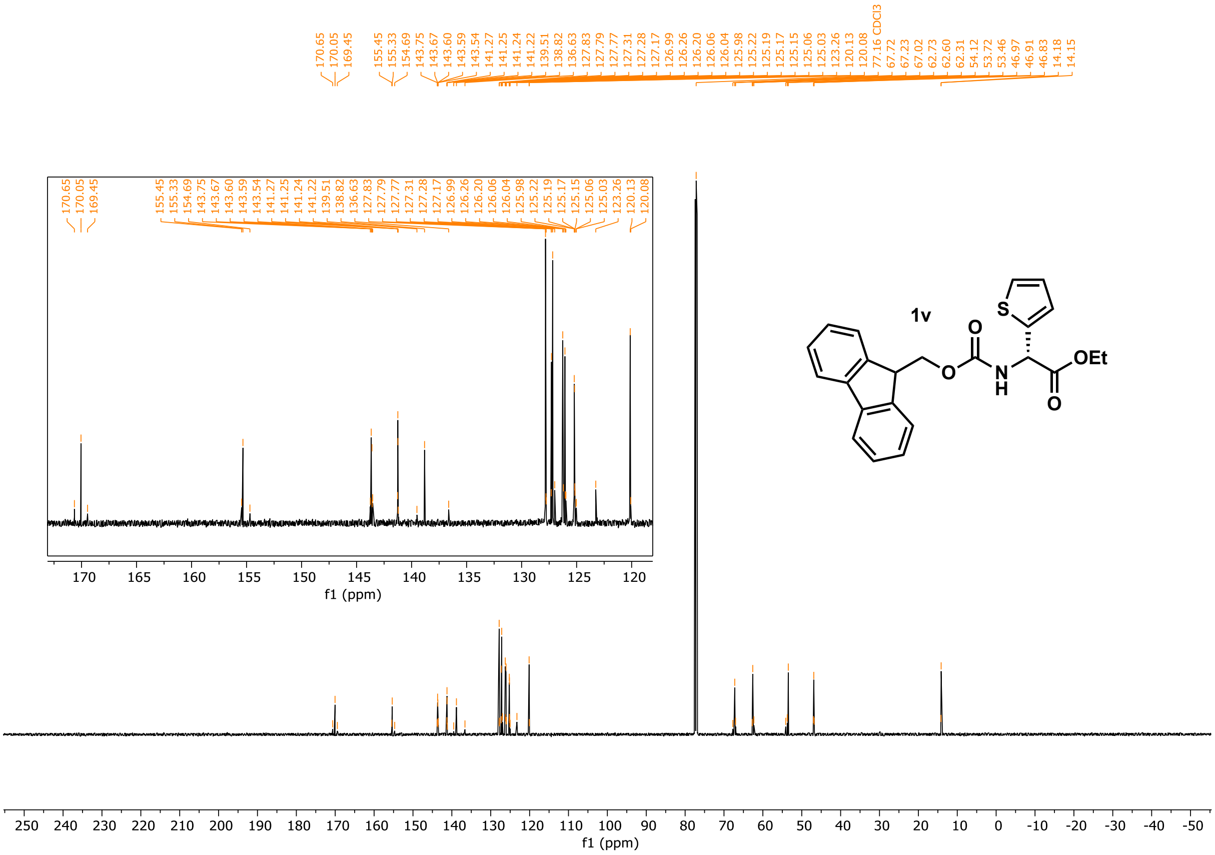

[13]**C-NMR** spectrum of compound **1v**.

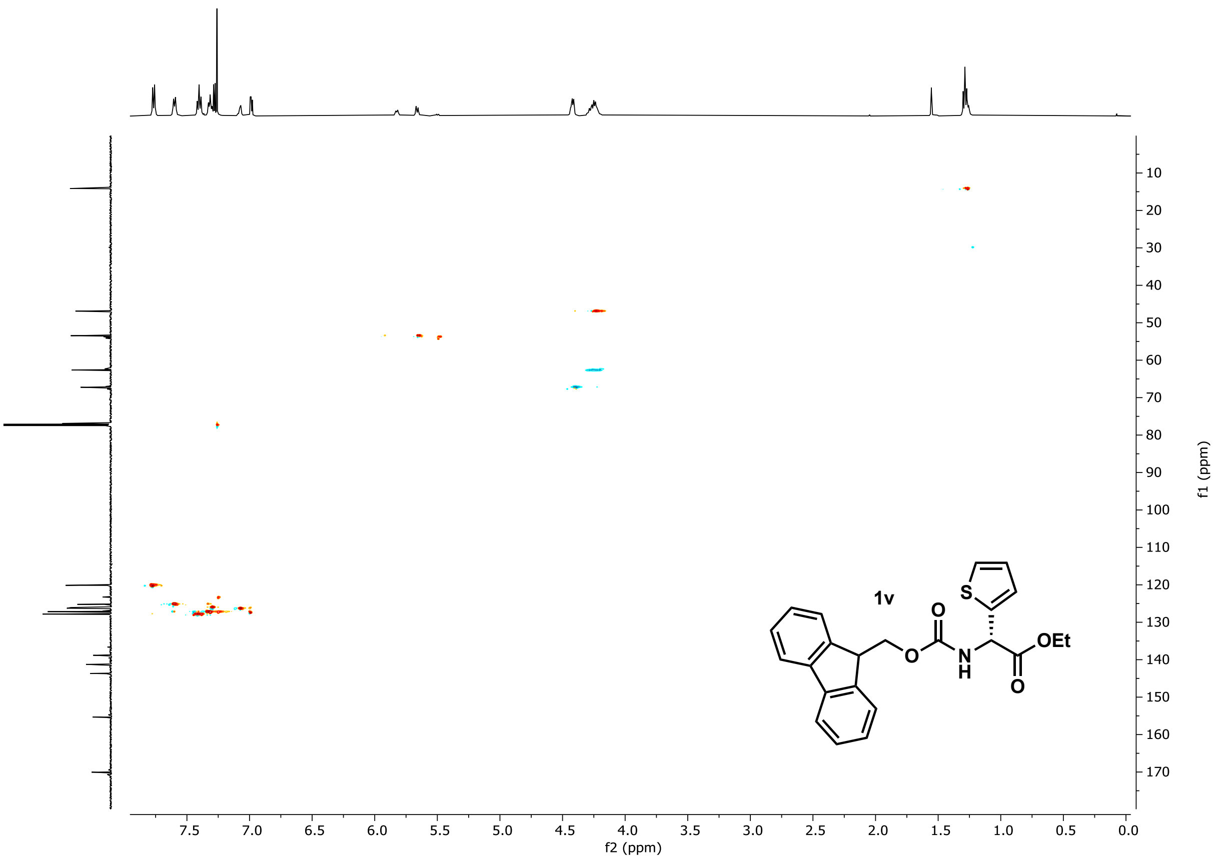

$^1H\{^{13}C\}$-**HSQC** spectrum of compound **1v**.

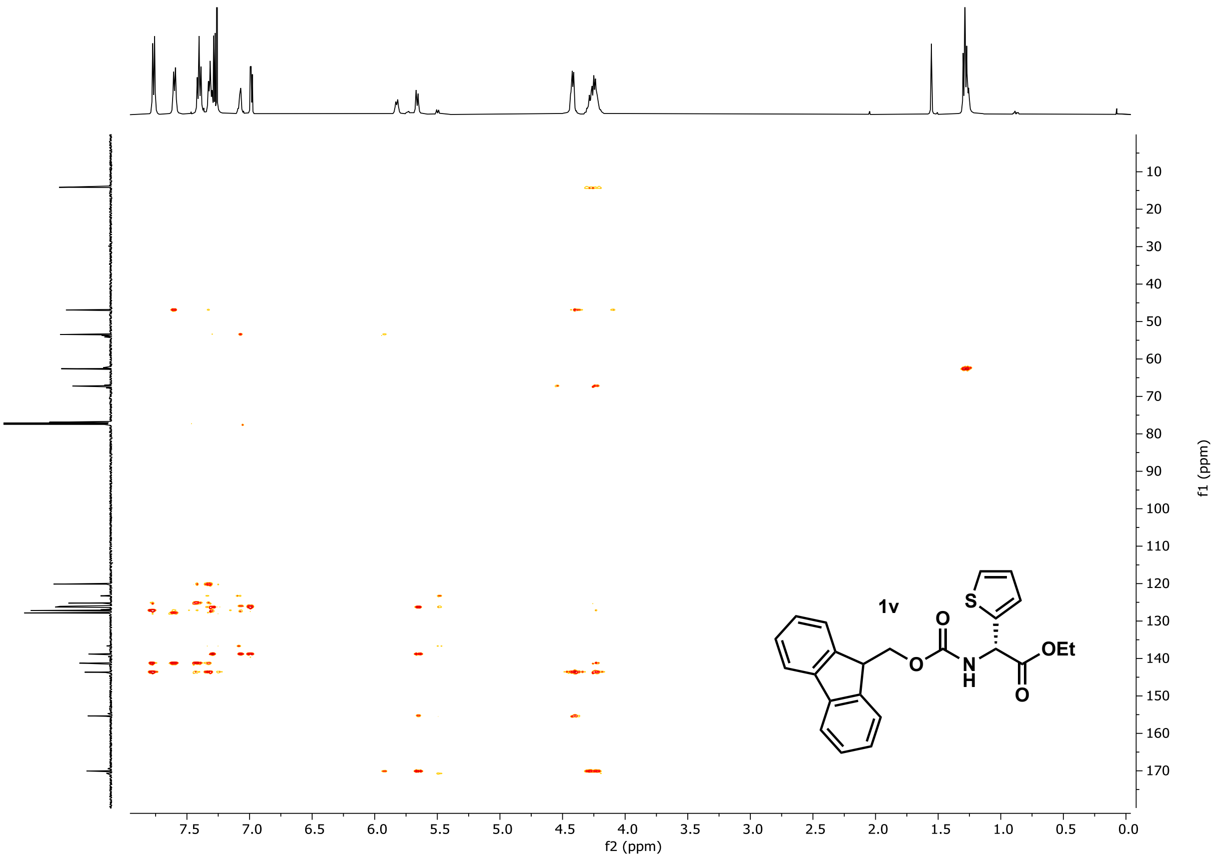

$^1H\{^{13}C\}$-**HMBC** spectrum of compound **1v**.

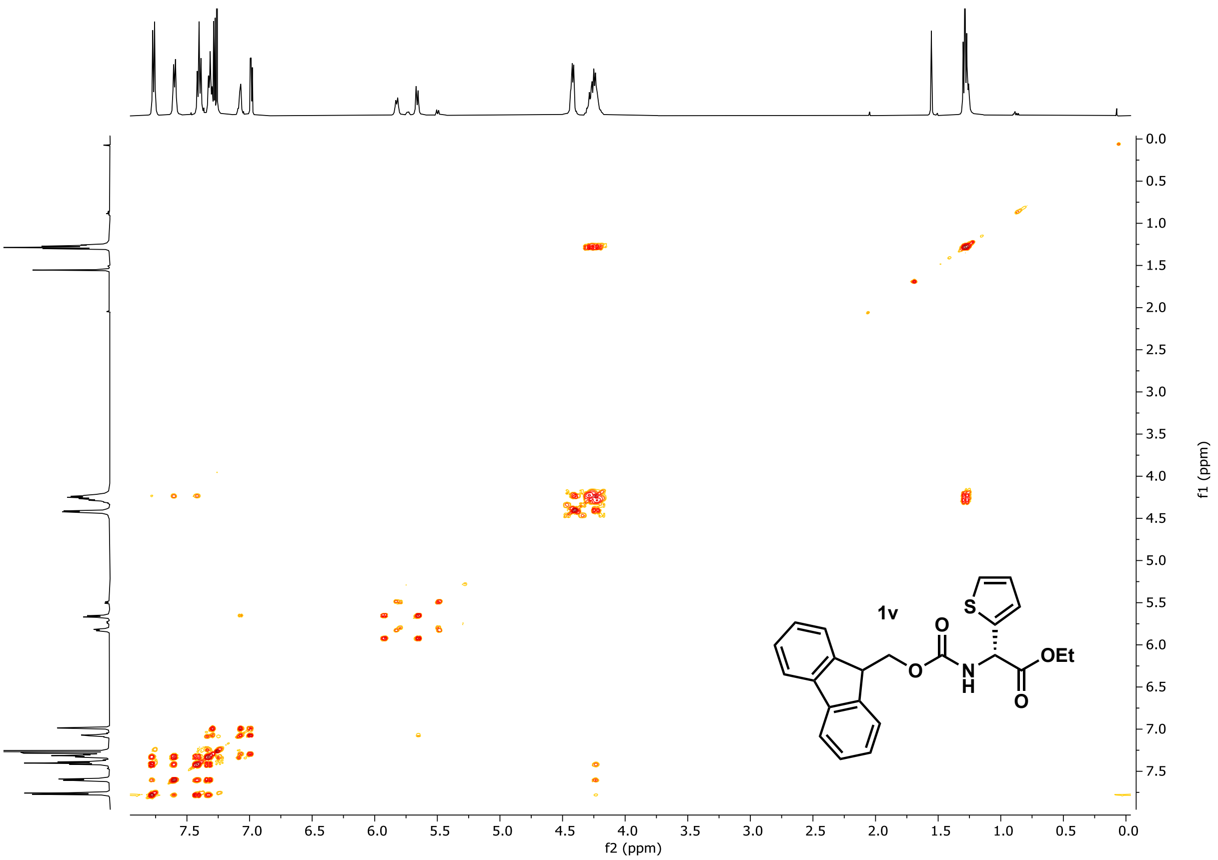

^{1}H-COSY spectrum of compound **1v**.

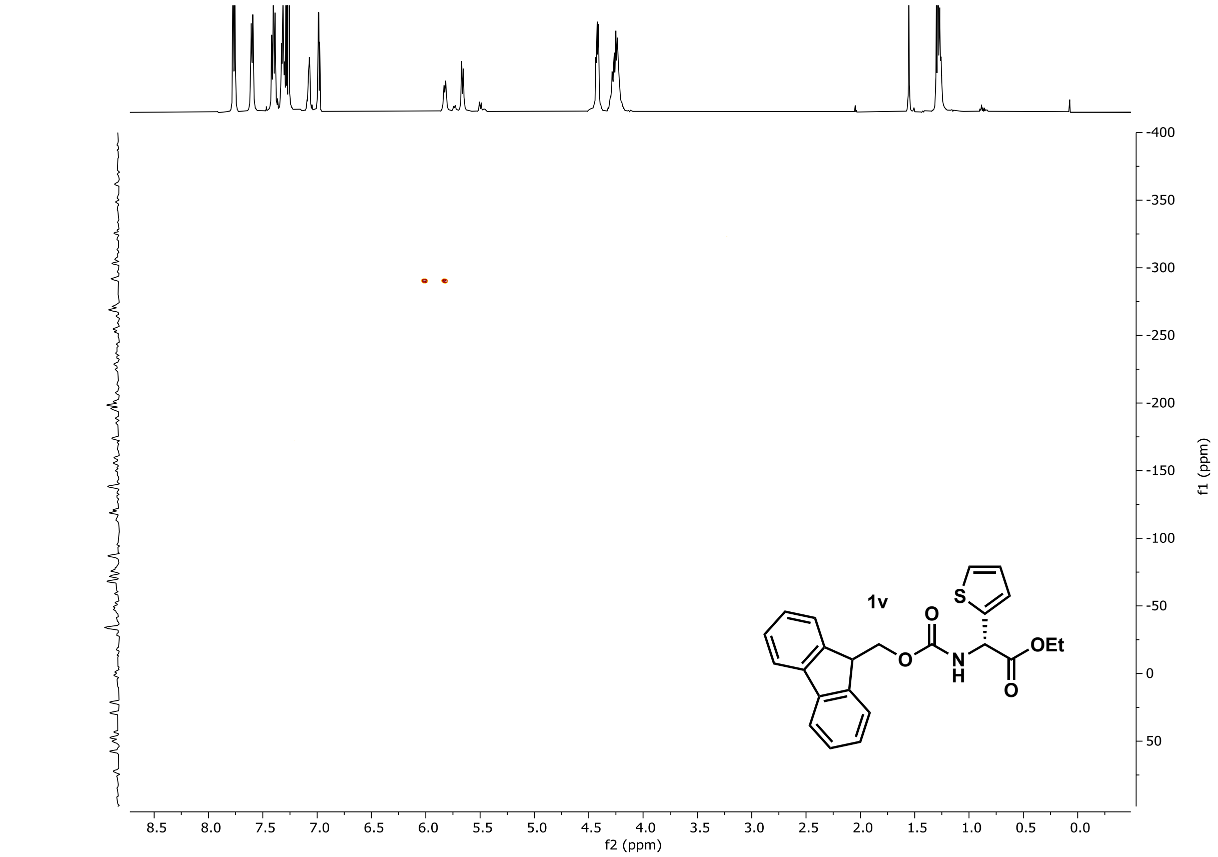

^{1}H{^{15}N}-HMBC spectrum of compound **1v**.

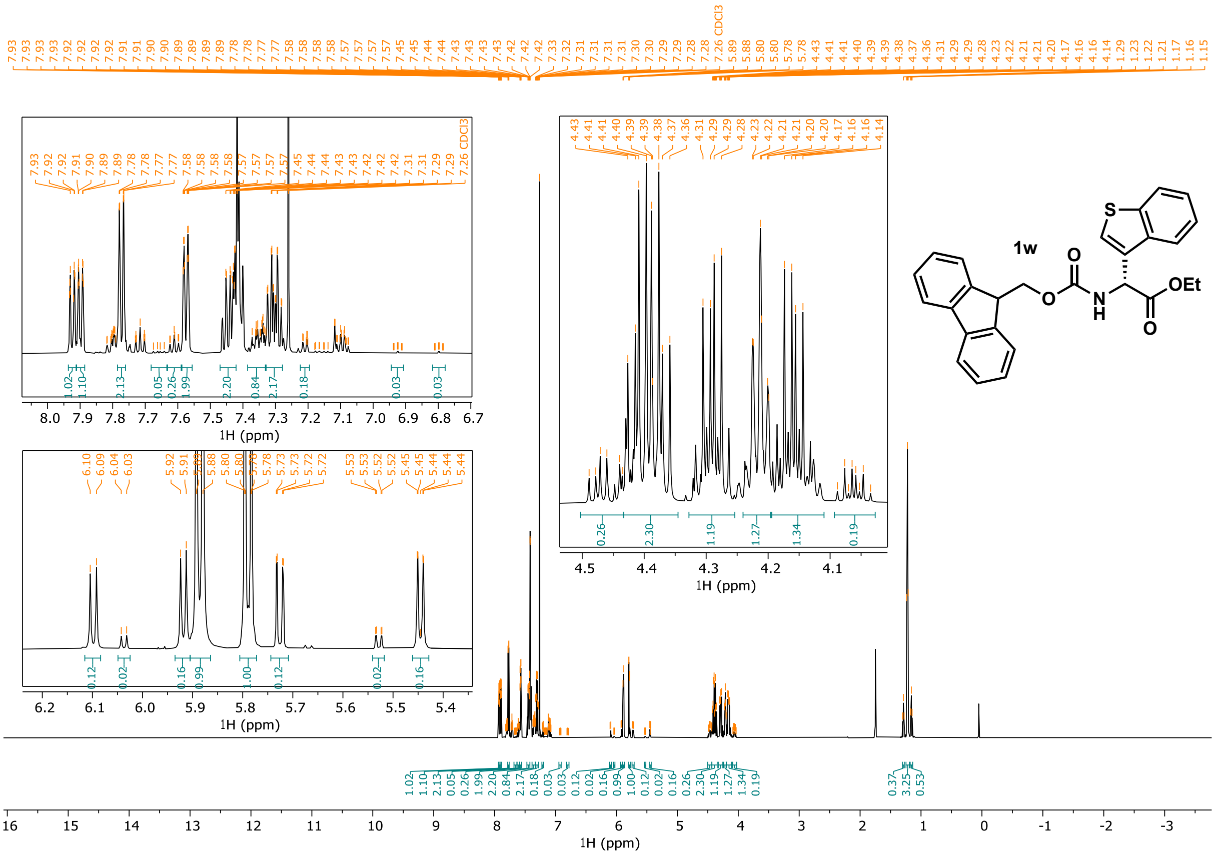

^{1}H-NMR spectrum of compound **1w**.

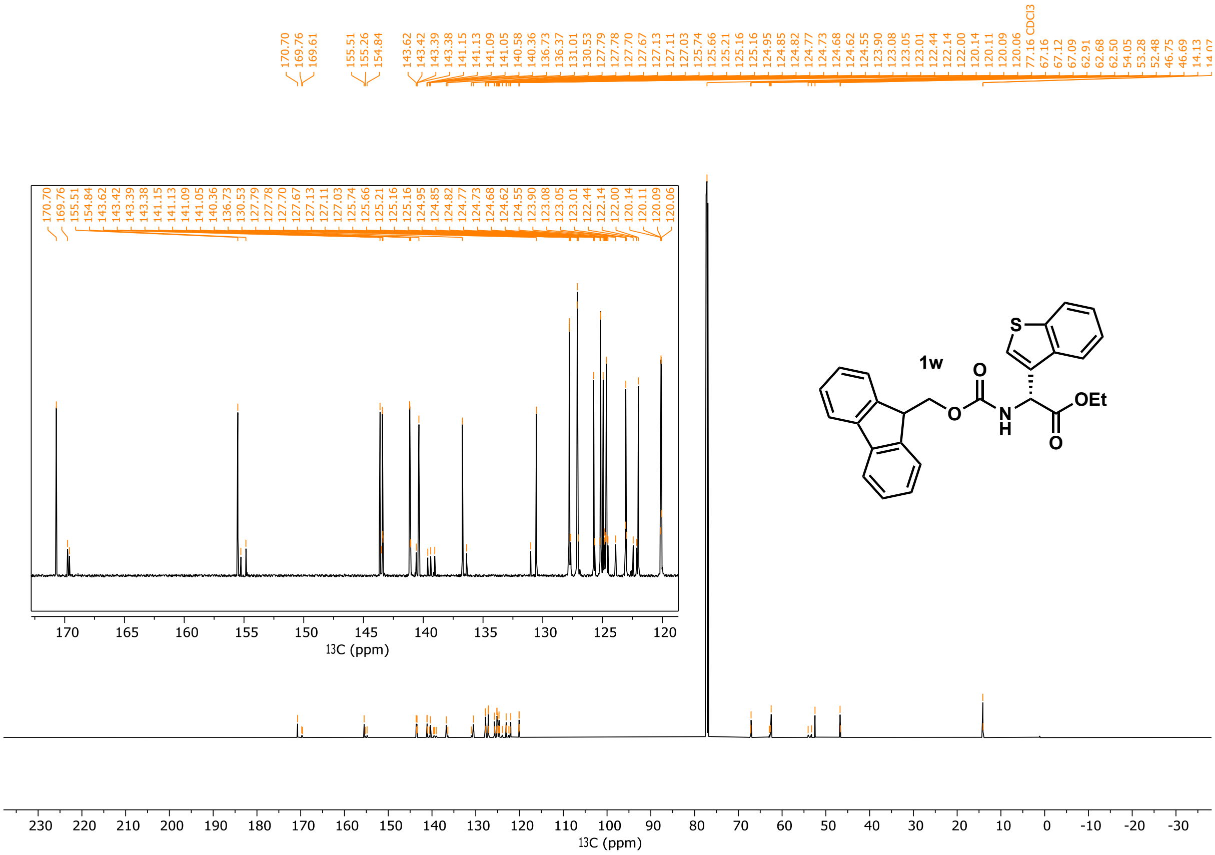

^{13}C-NMR spectrum of compound **1w**.

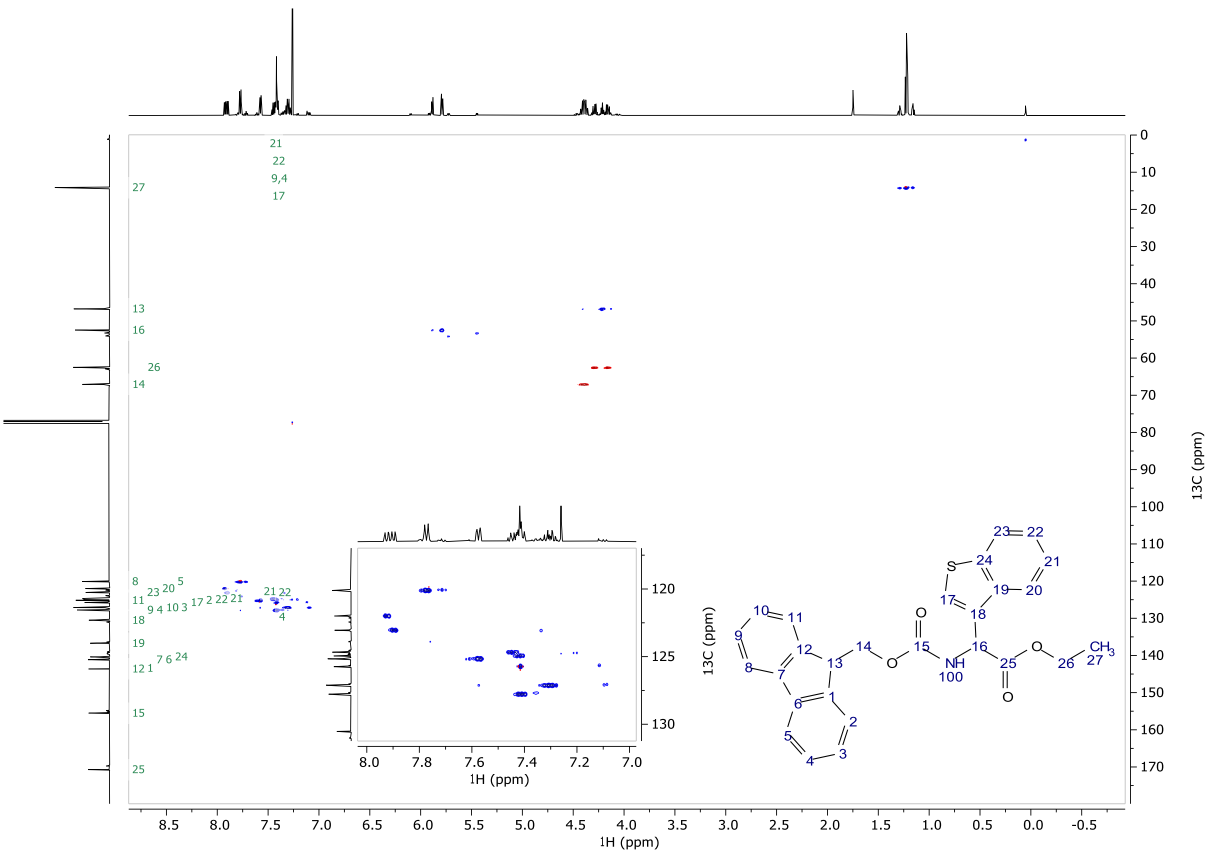

^{1}H{^{13}C}-HSQC spectrum of compound **1w**.

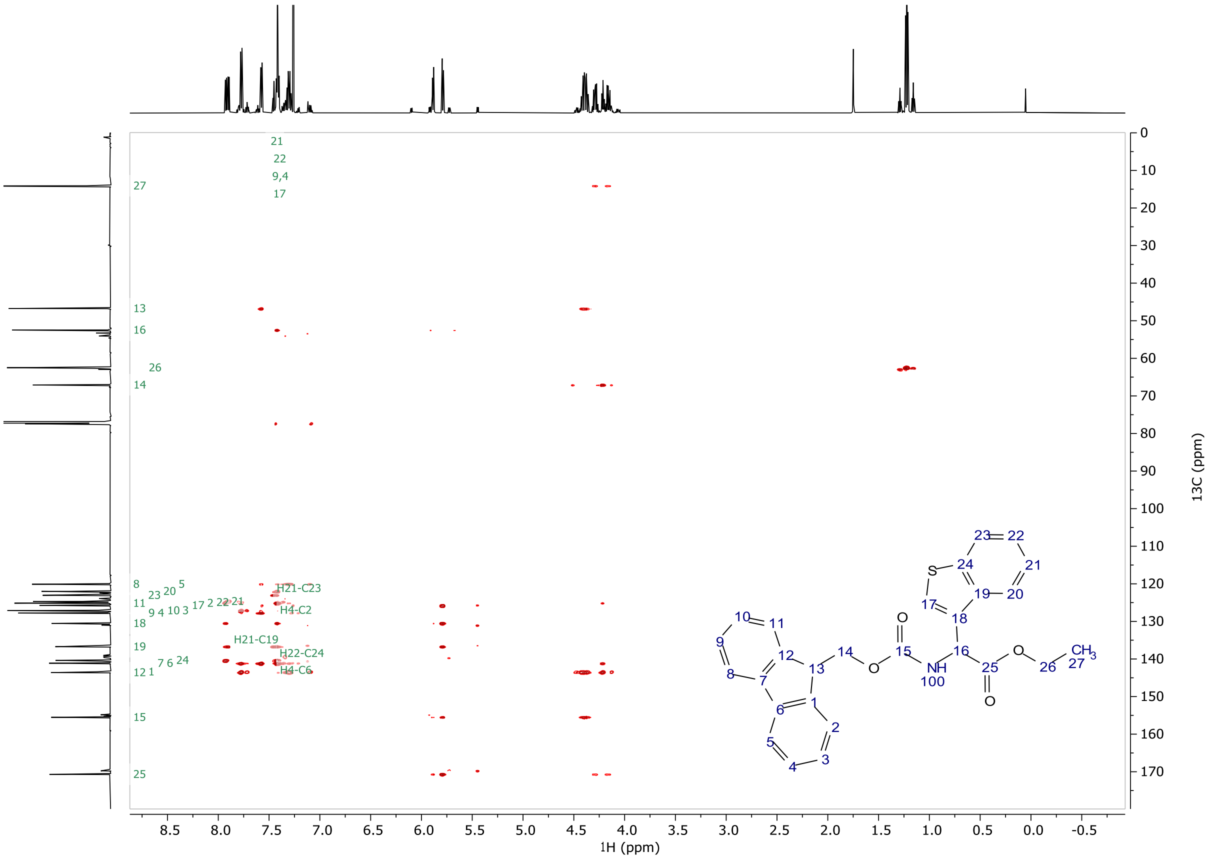

^{1}H{^{13}C}-HMBC spectrum of compound **1w**.

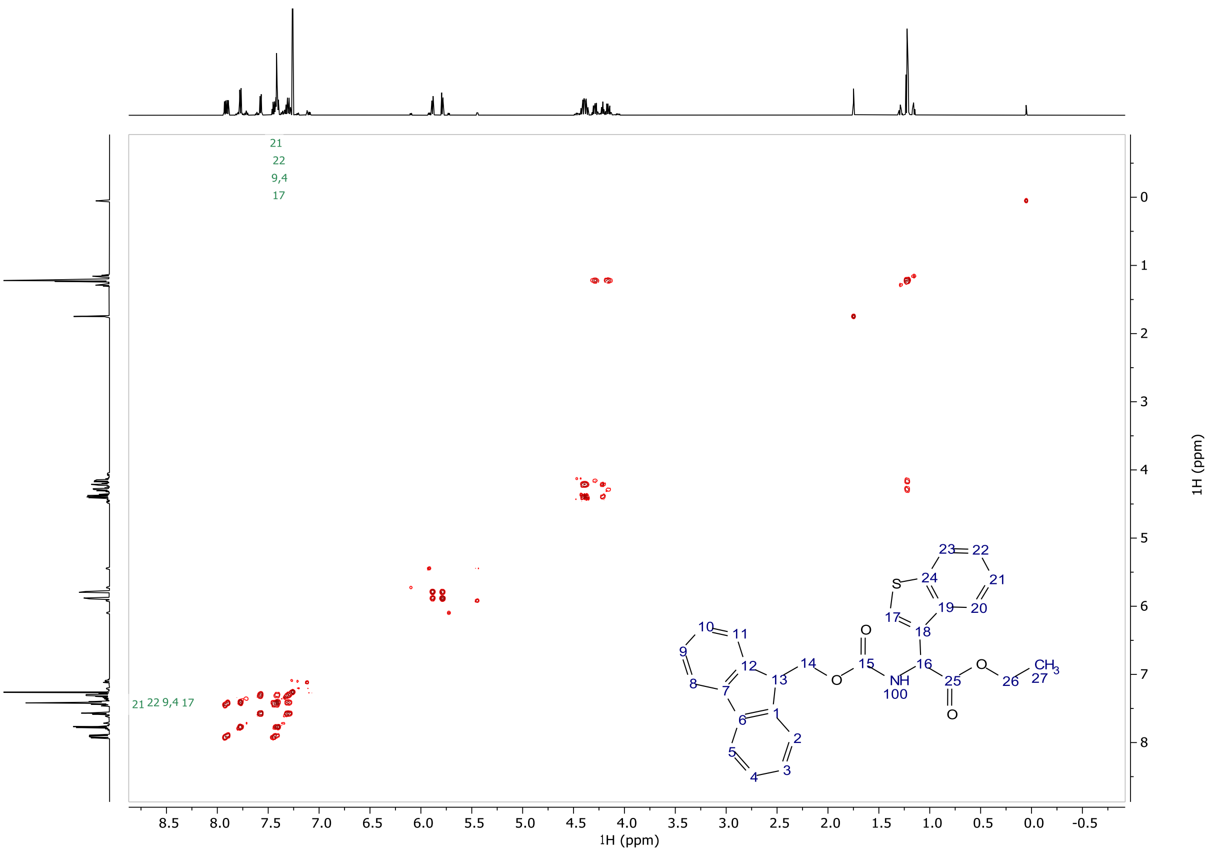

^{1}H-COSY spectrum of compound **1w**.

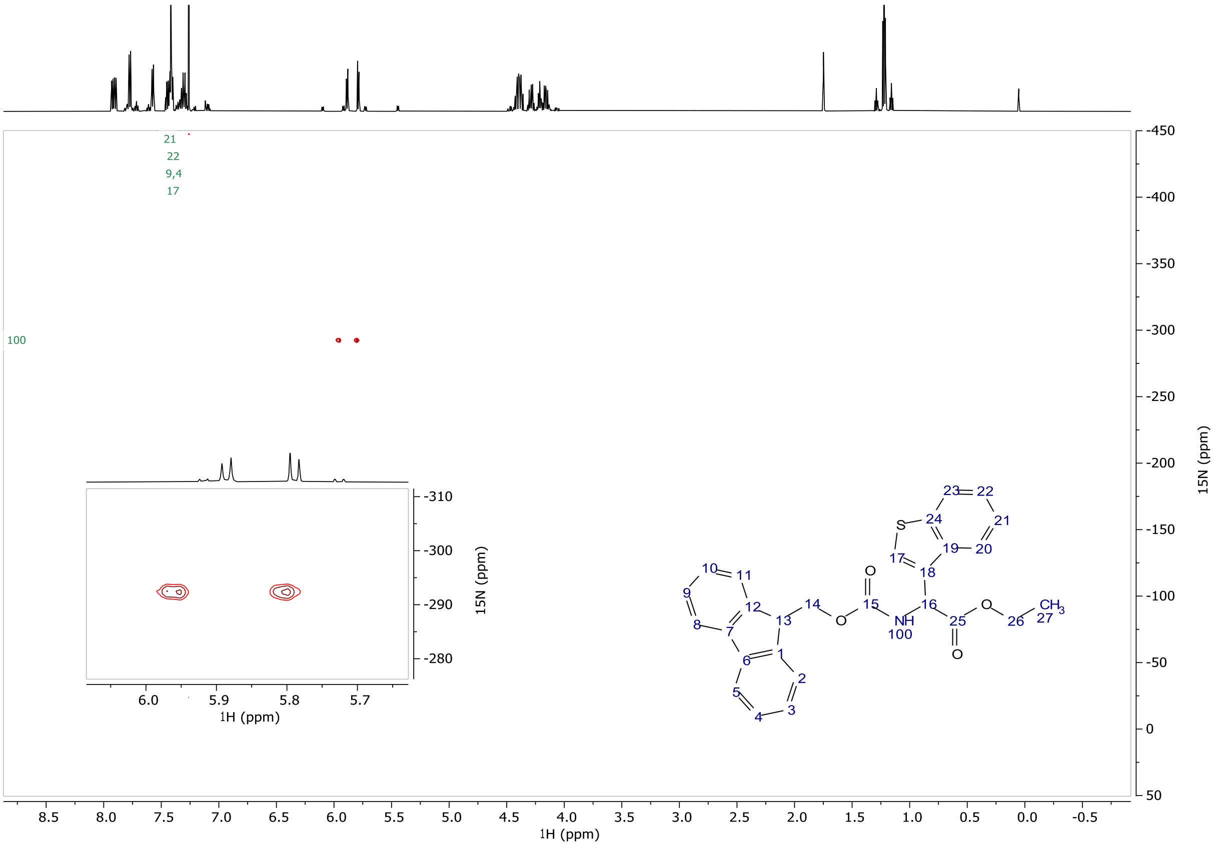

^{1}H{^{15}N}-HMBC spectrum of compound **1w**.

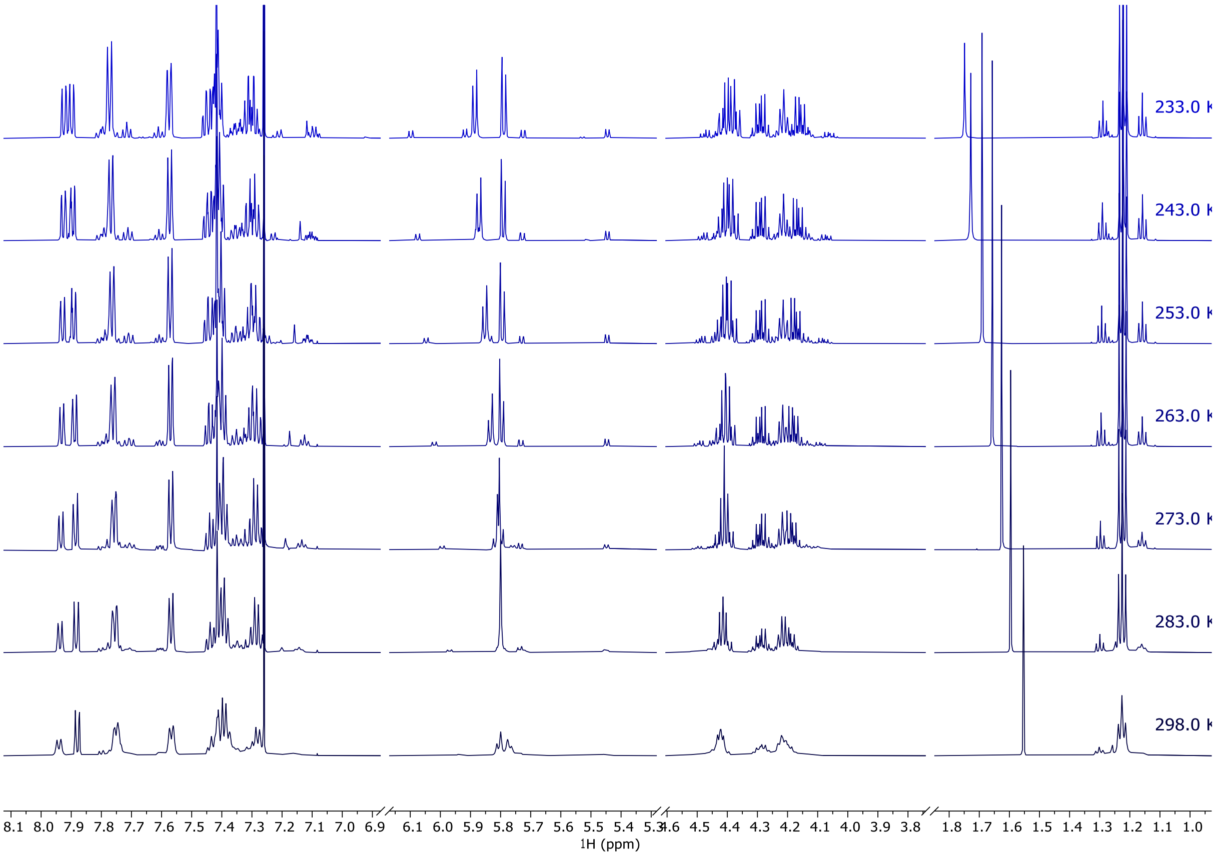

Stackplot of the temperature profile of the **^{1}H-NMR** spectra of compound **1w** from 298 K to 233 K.

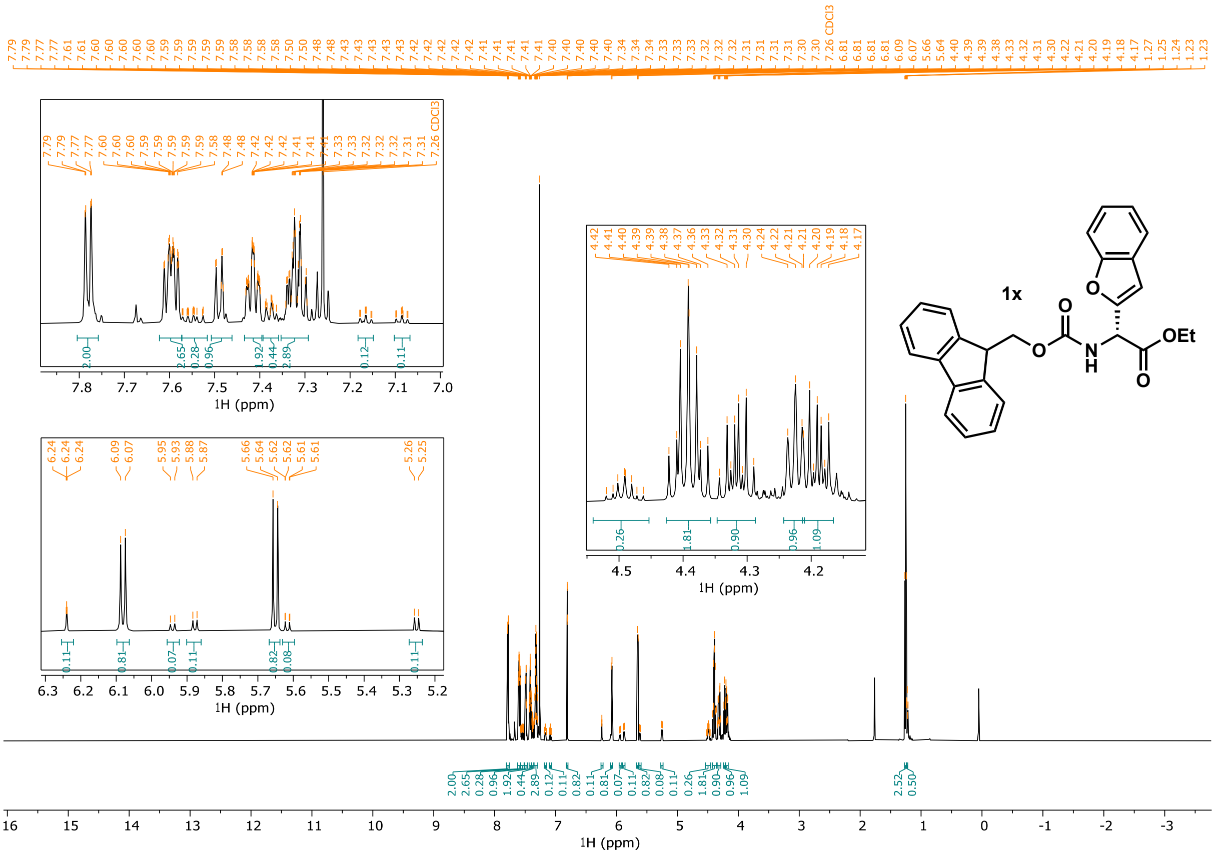

¹H-NMR spectrum of compound **1x**.

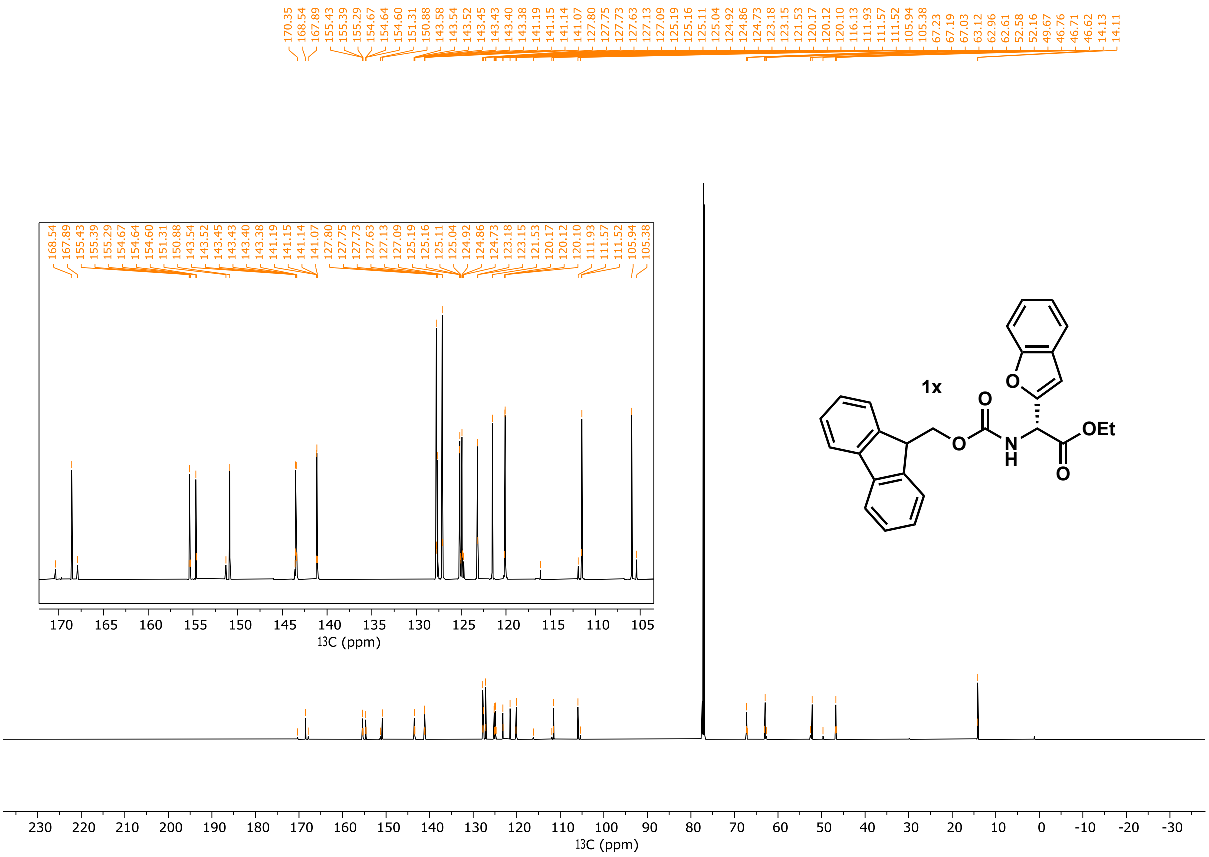

¹³C-NMR spectrum of compound **1x**.

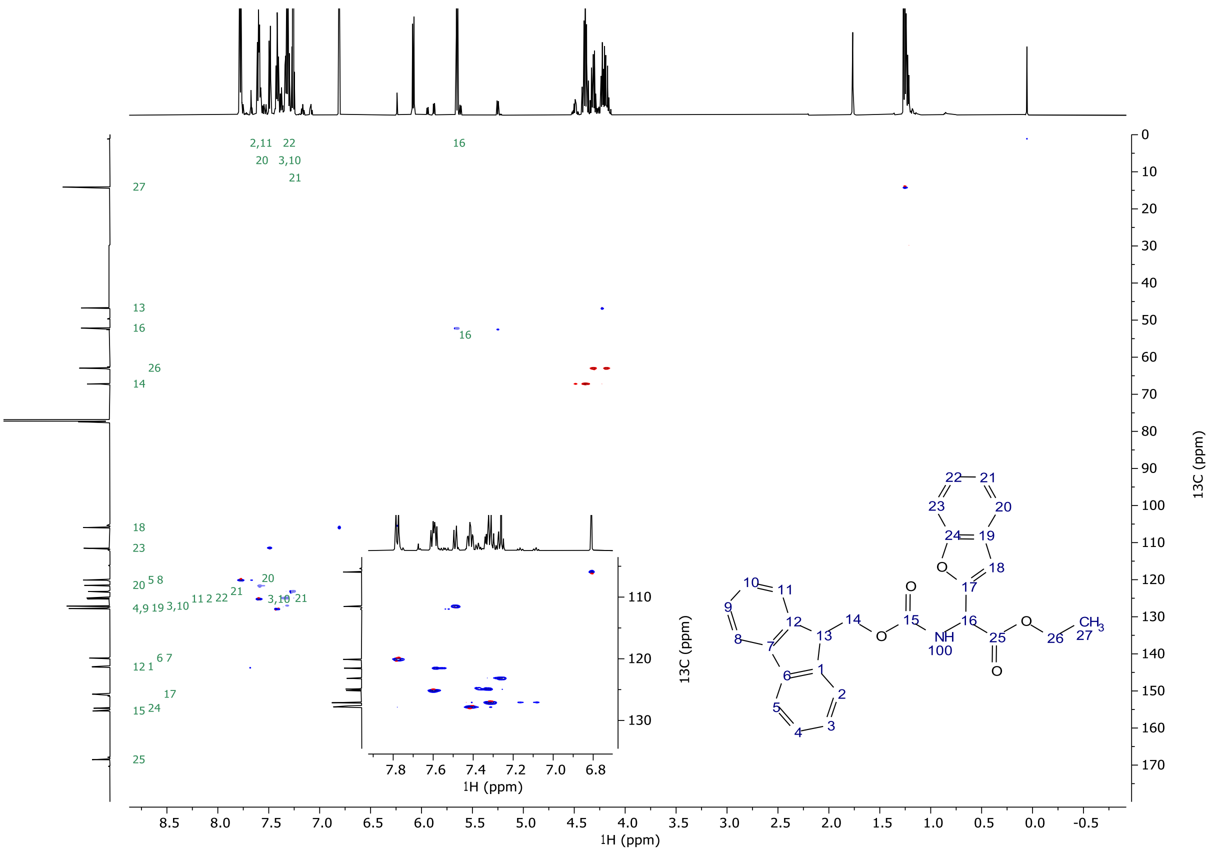

$^{1}H\{^{13}C\}$-HSQC spectrum of compound **1x**.

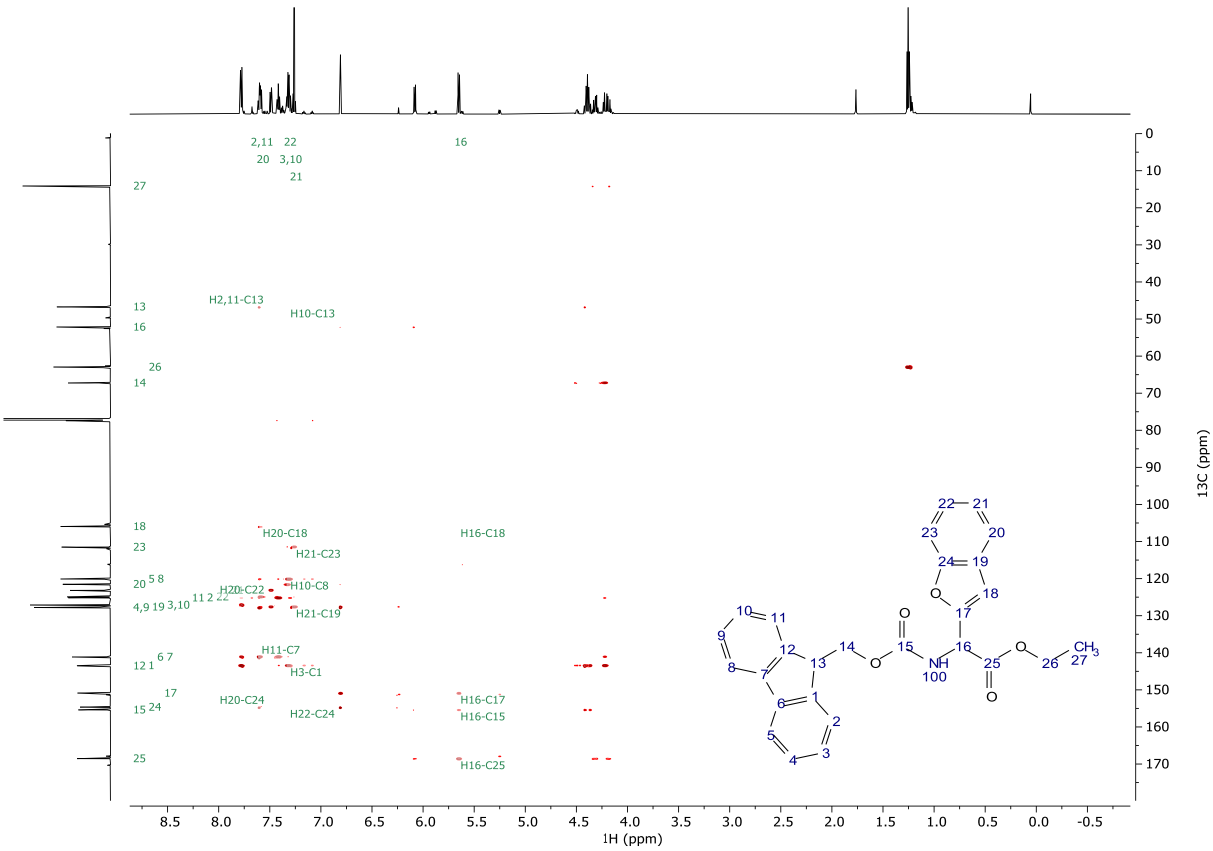

$^{1}H\{^{13}C\}$-HMBC spectrum of compound **1x**.

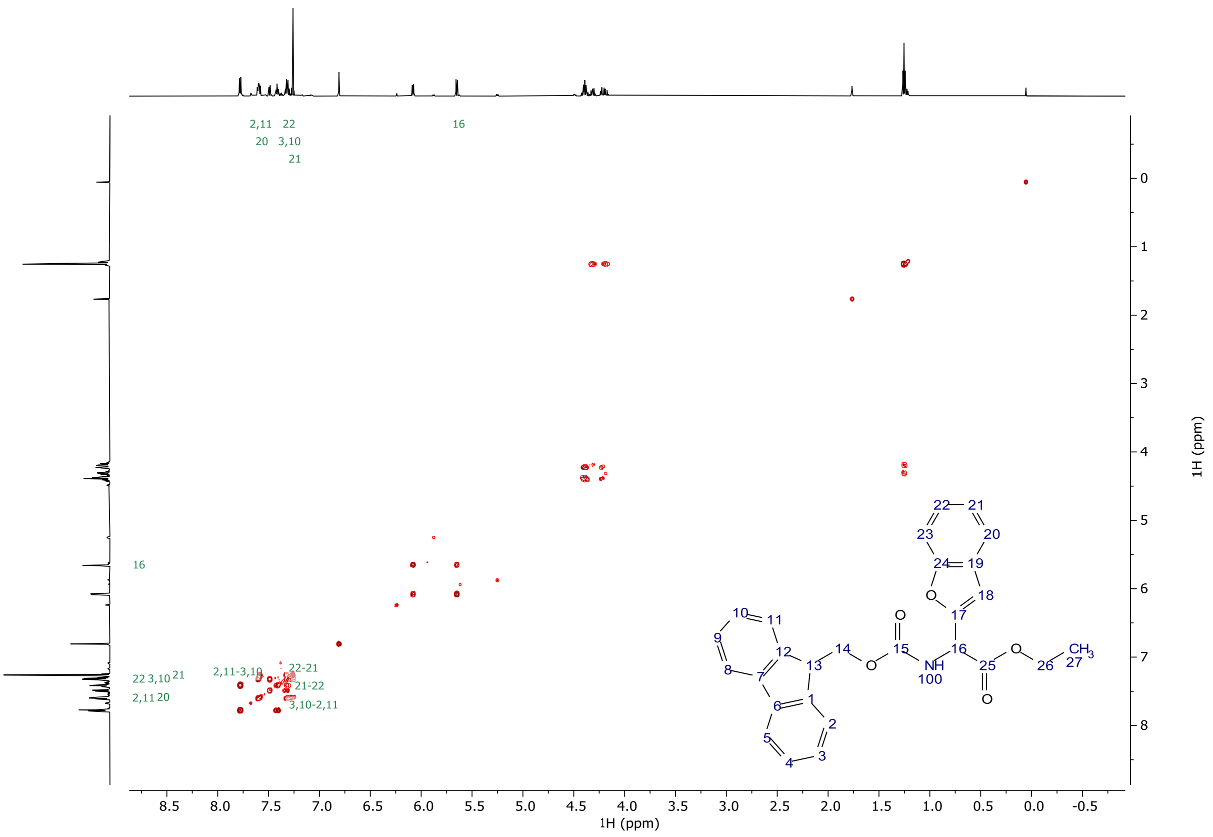

^{1}**H-COSY** spectrum of compound **1x**.

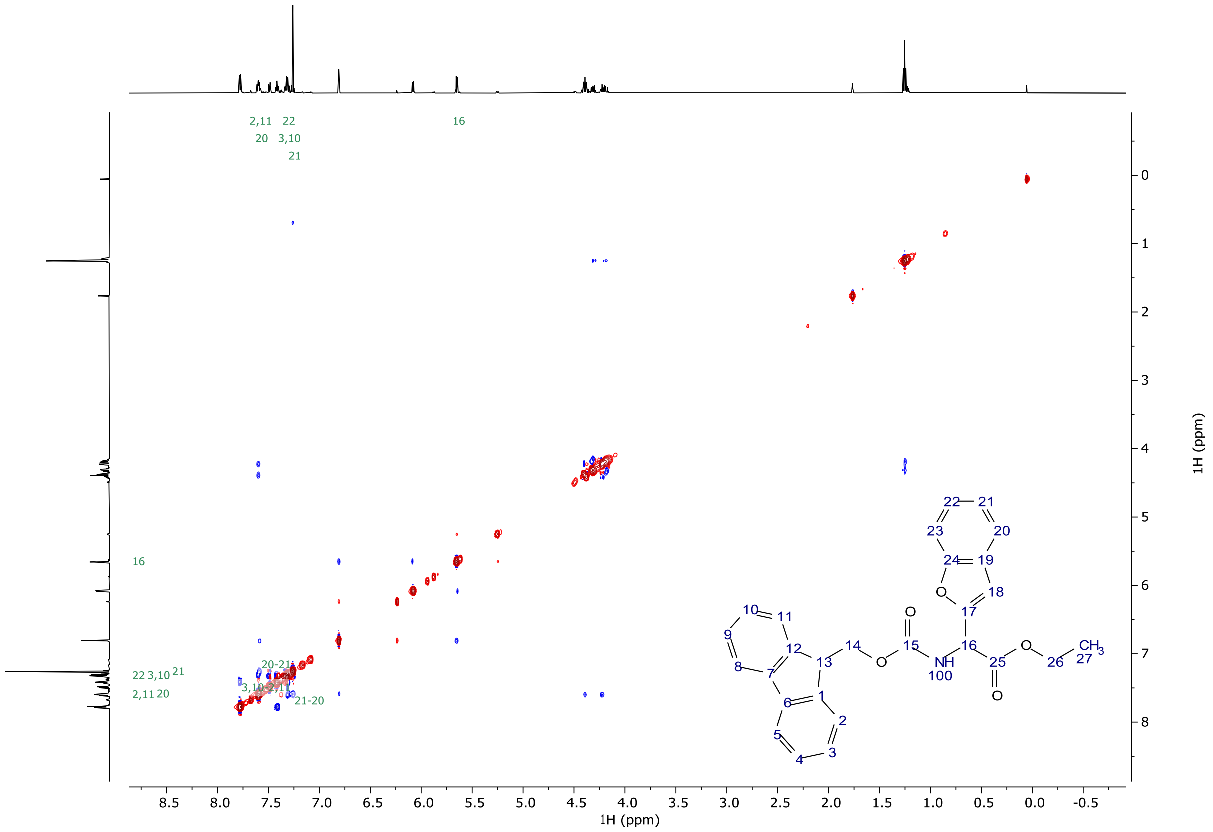

^{1}**H-NOESY** spectrum of compound **1x**.

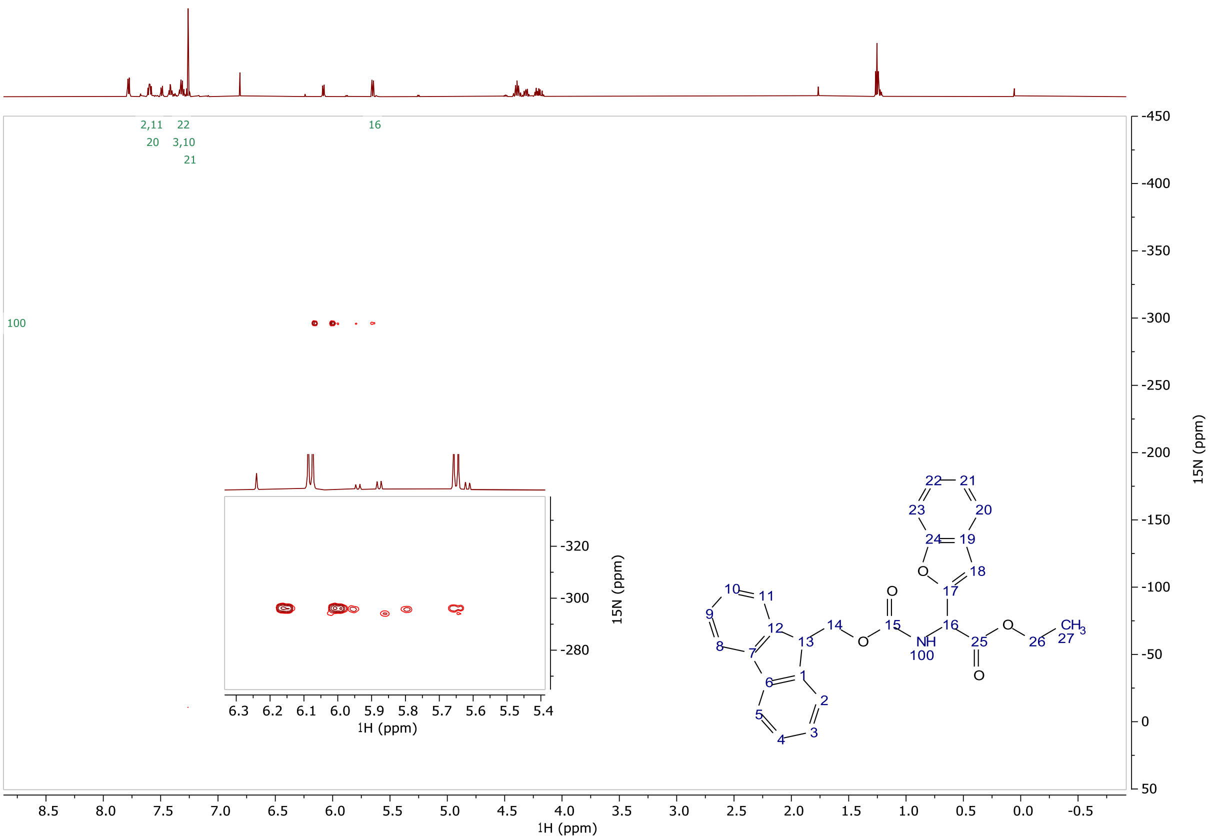

^{1}**H{^{15}N}-HMBC** spectrum of compound **1x**.

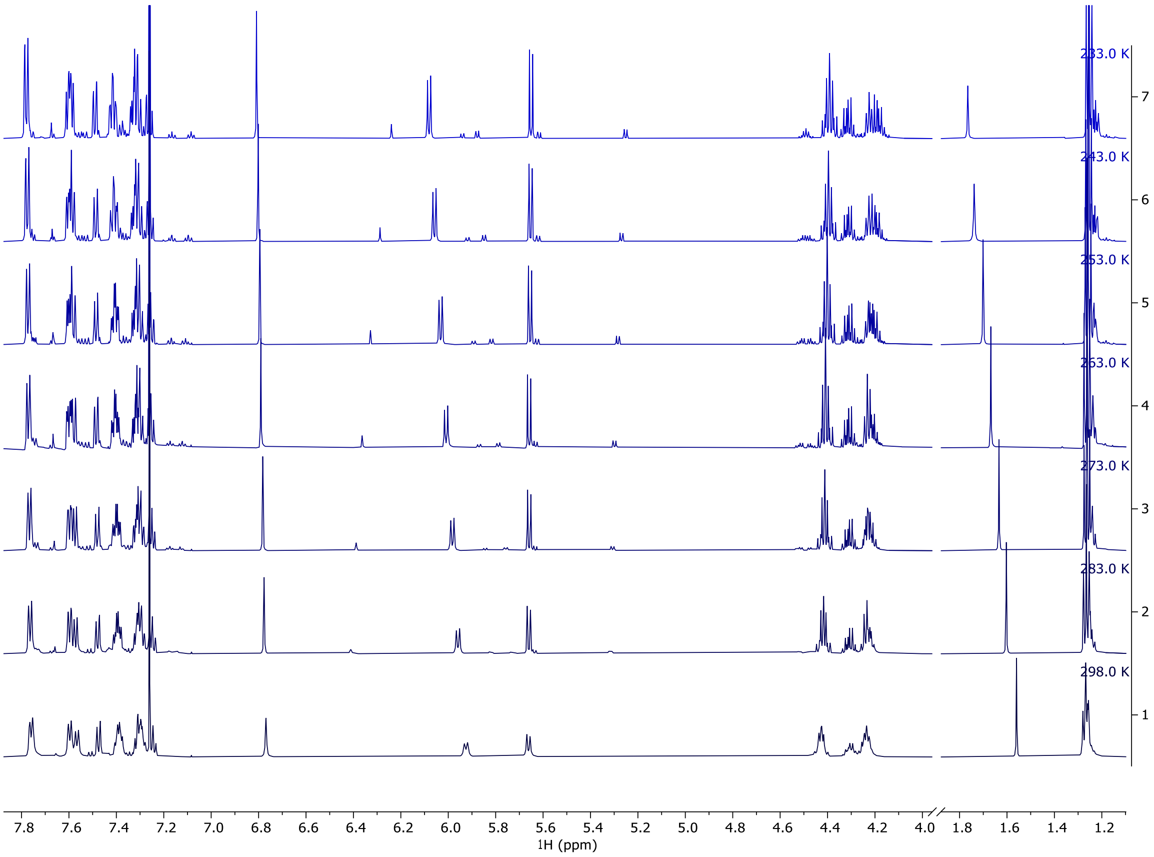

Stackplot of the temperature profile of the ^{1}**H-NMR** spectra of compound **1x** from 298 K to 233 K.

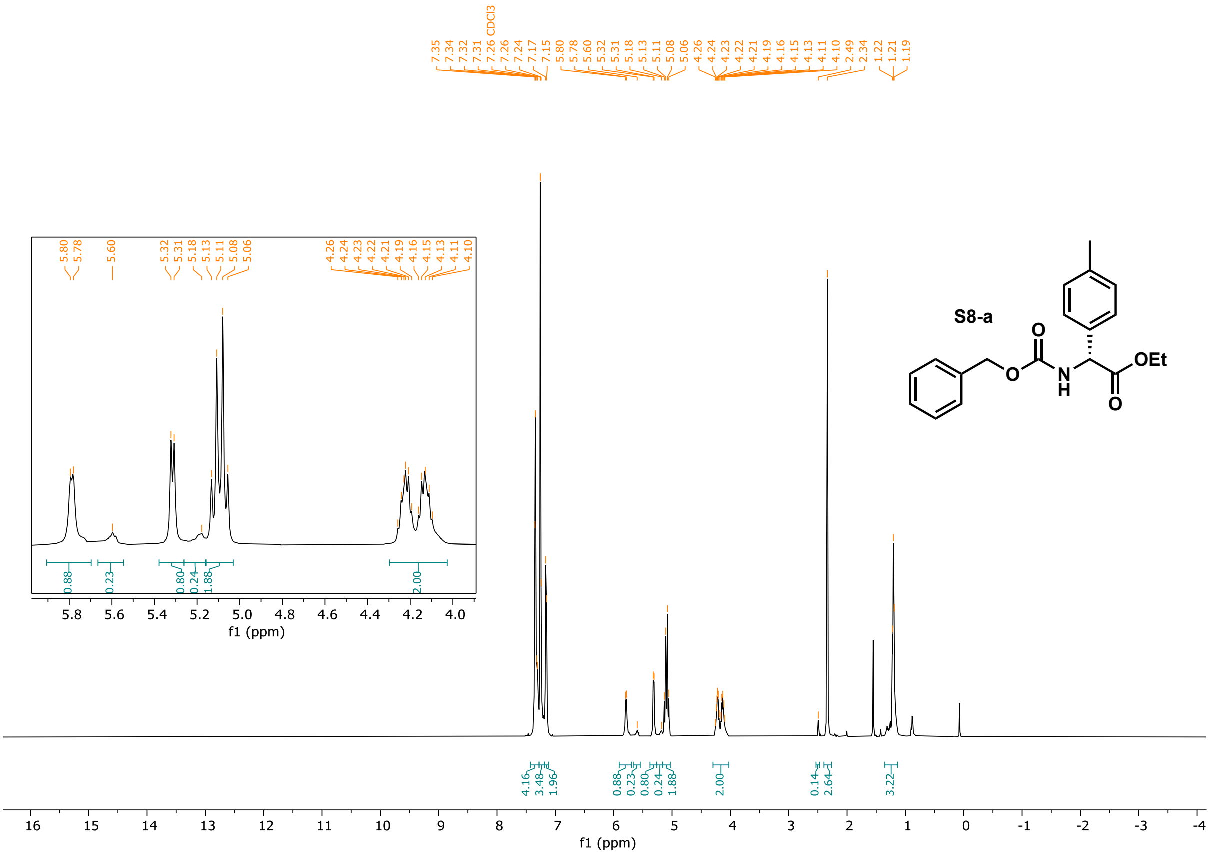

^{1}H-NMR spectrum of compound **S8-a**.

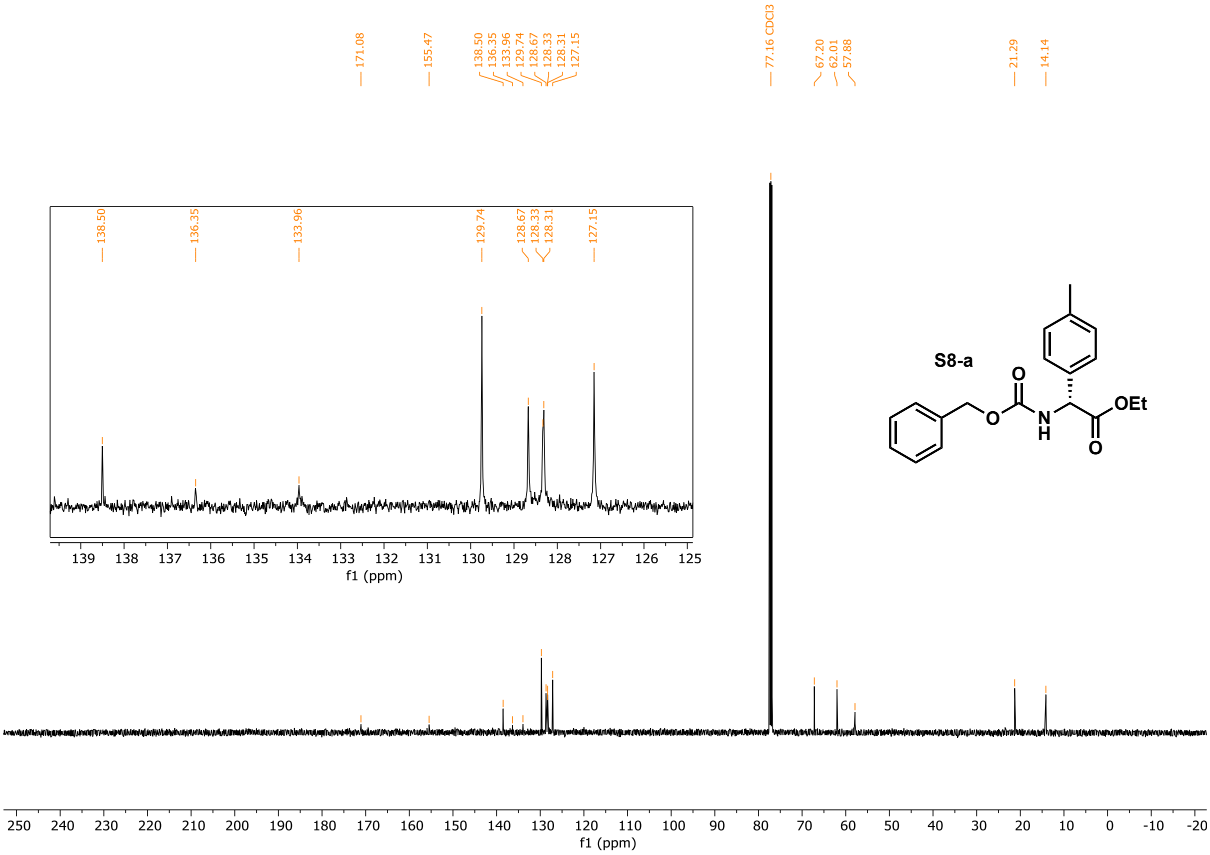

^{13}C-NMR spectrum of compound **S8-a**.

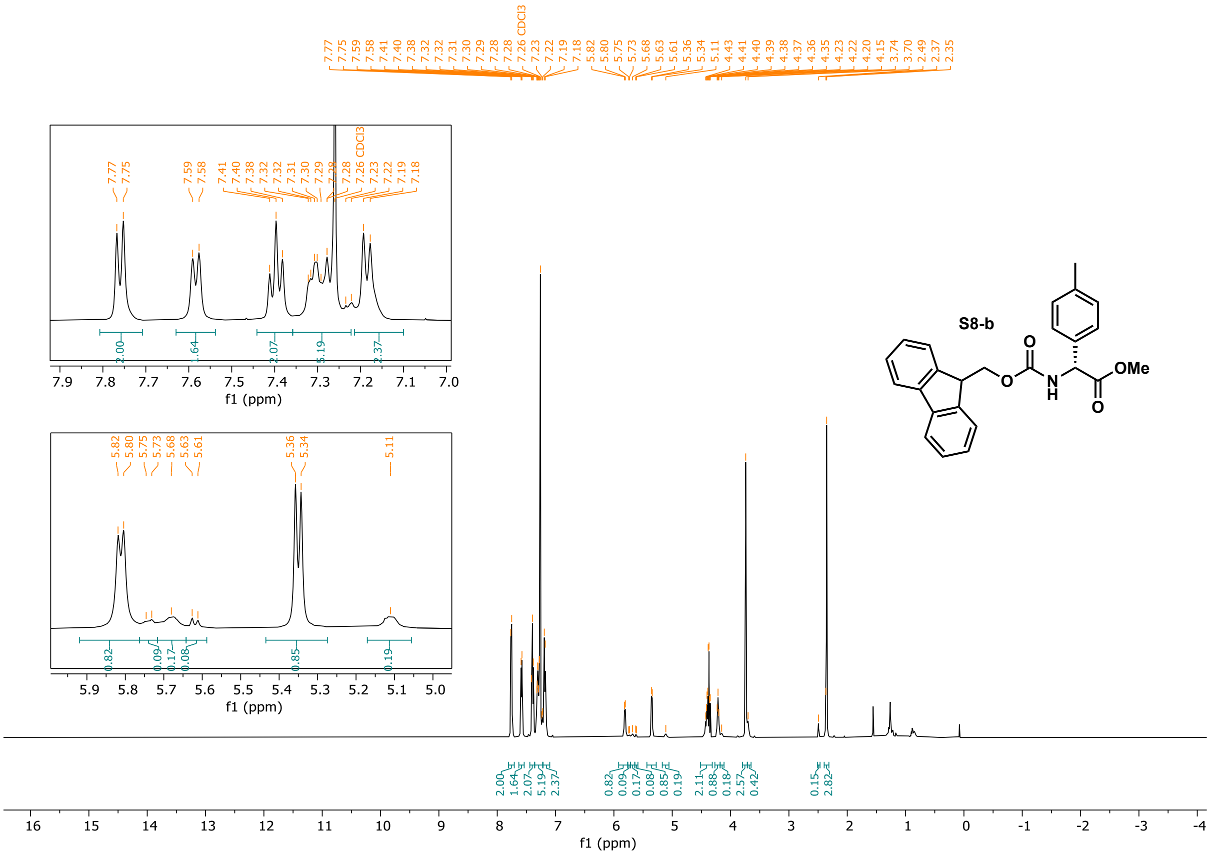

^{1}**H-NMR** spectrum of compound **S8-b**.

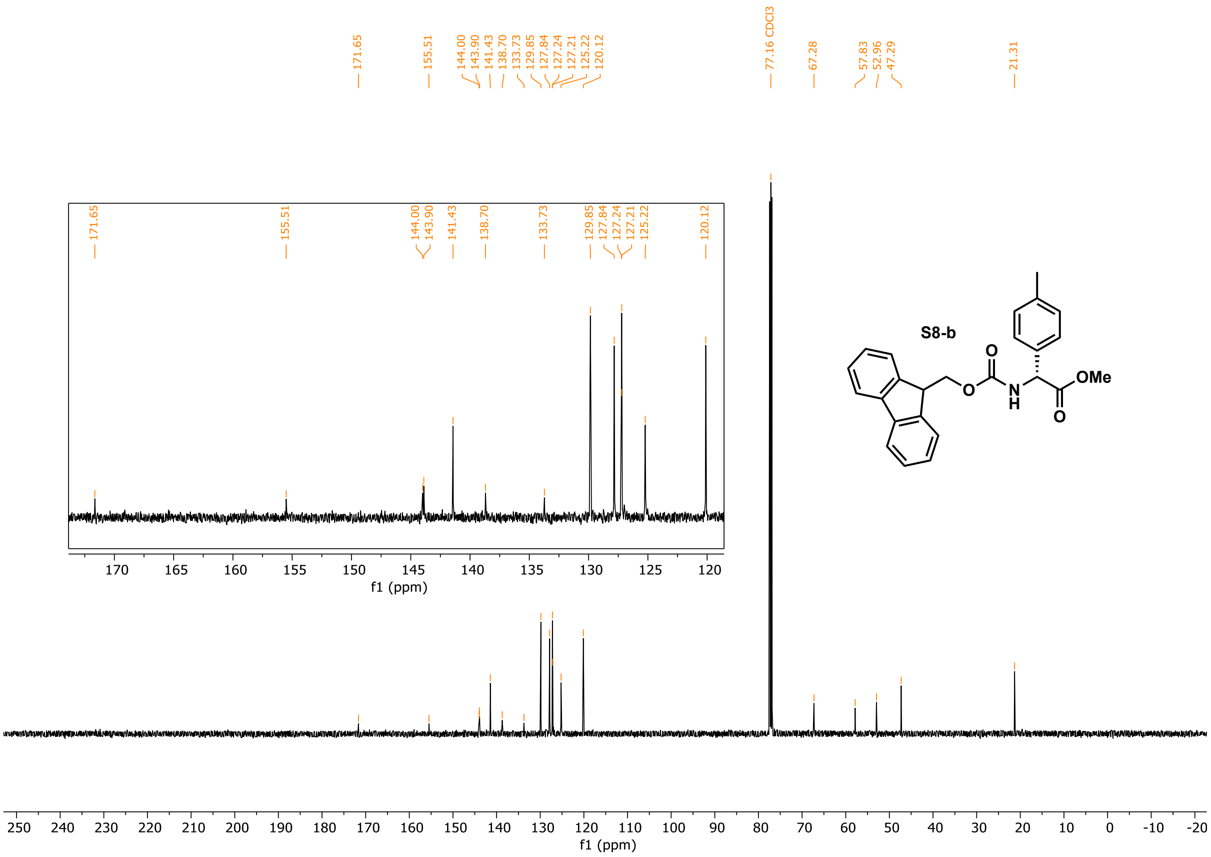

^{13}**C-NMR** spectrum of compound **S8-b**.

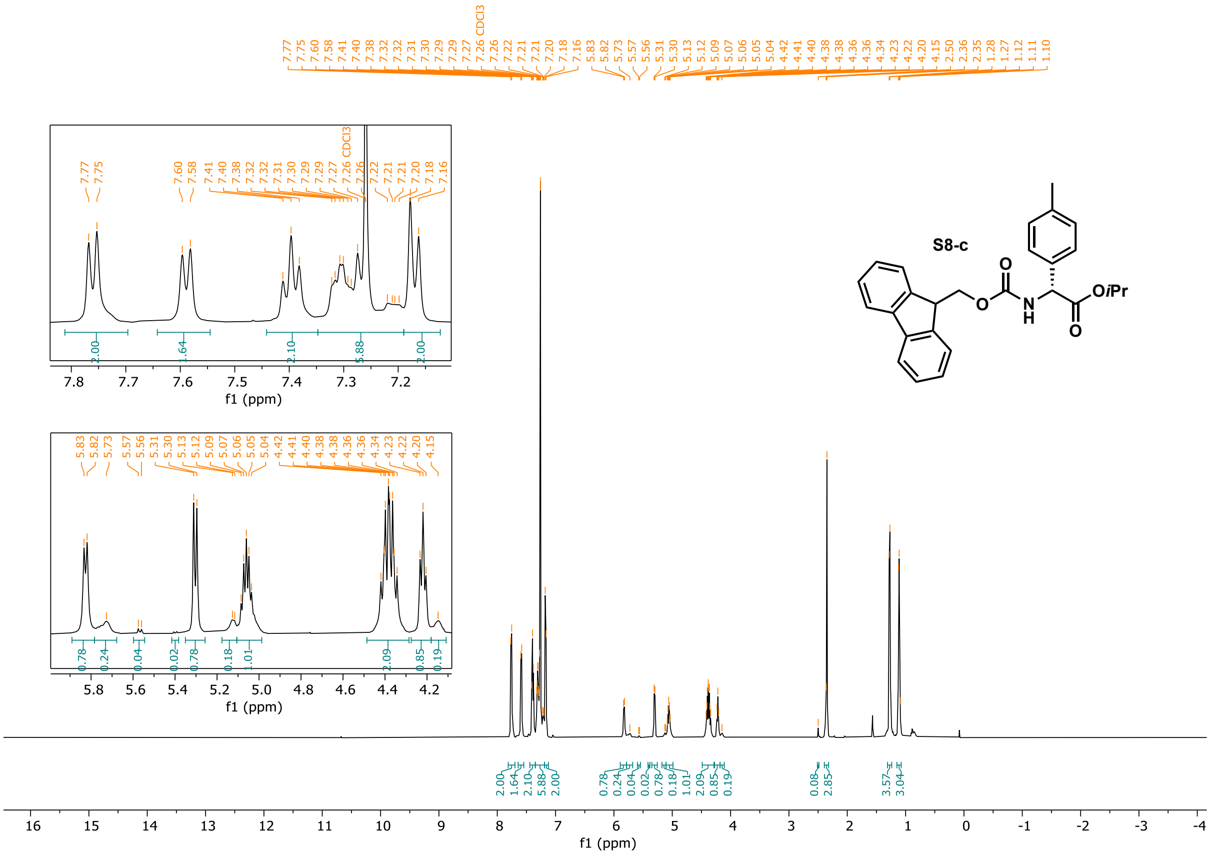

^{1}**H-NMR** spectrum of compound **S8-c**.

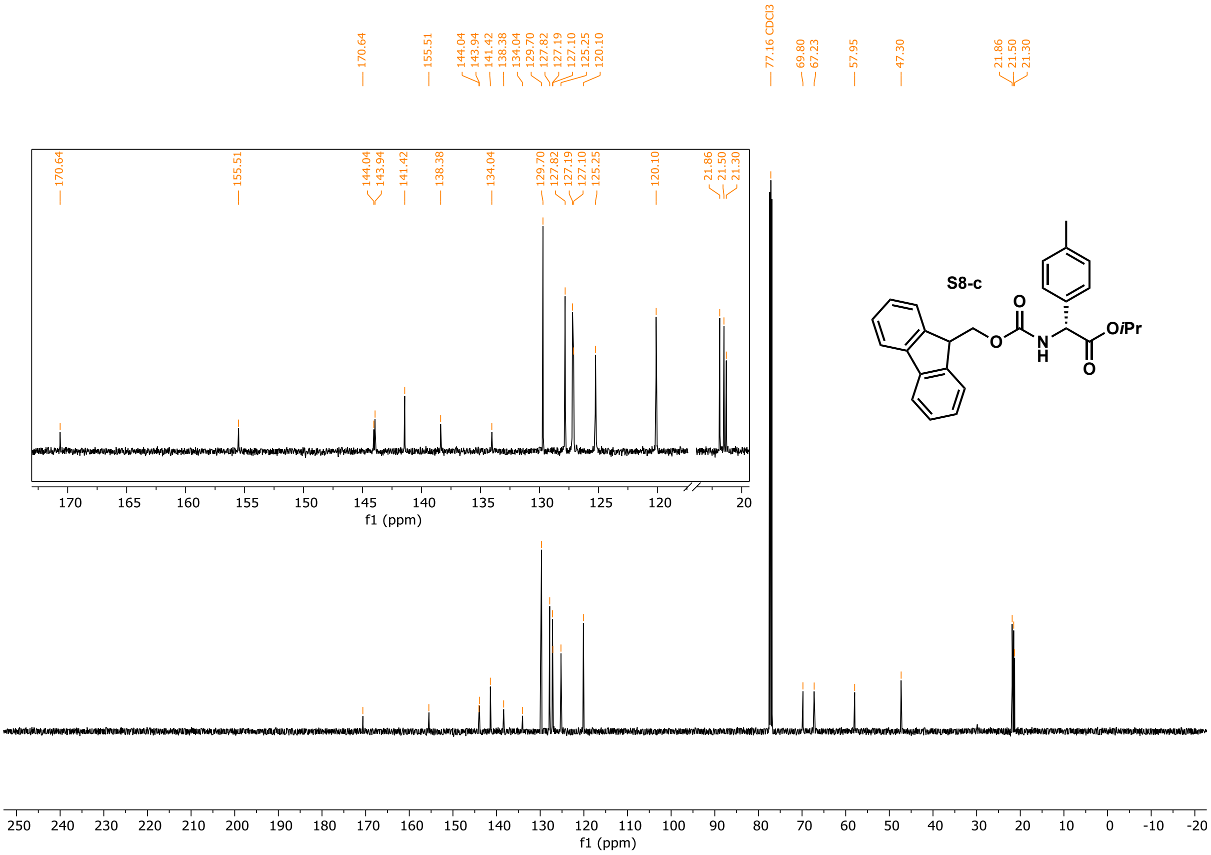

^{13}**C-NMR** spectrum of compound **S8-c**.

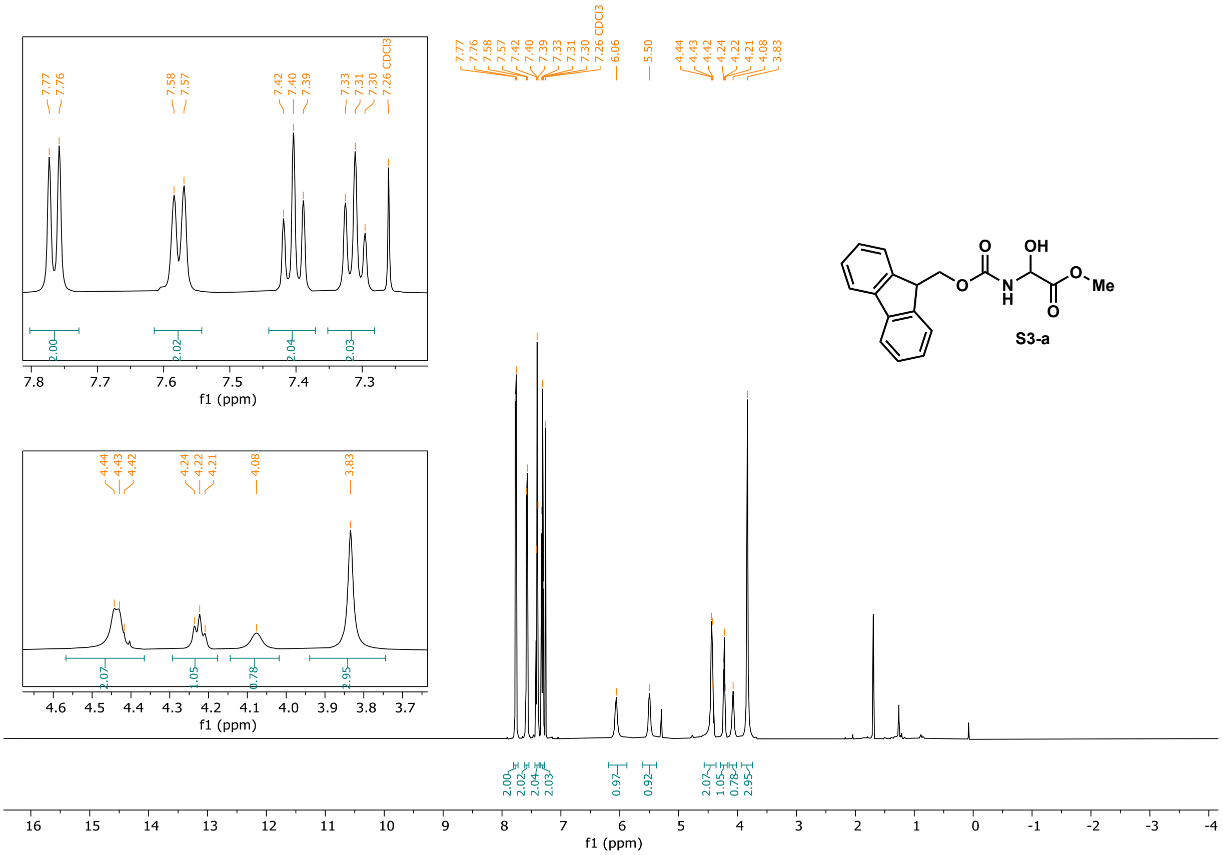

[1]**H-NMR** spectrum of compound **S3-a**.

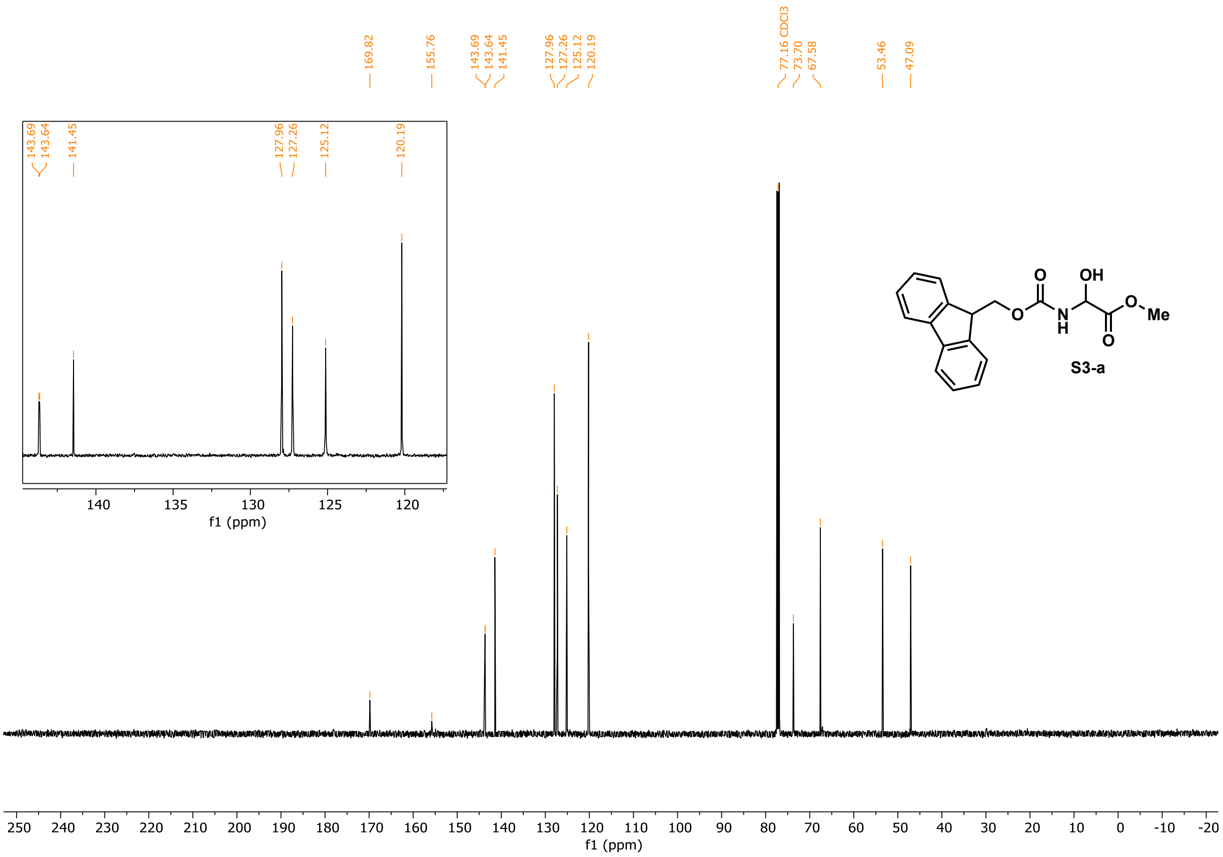

[13]**-C-NMR** spectrum of compound **S3-a**.

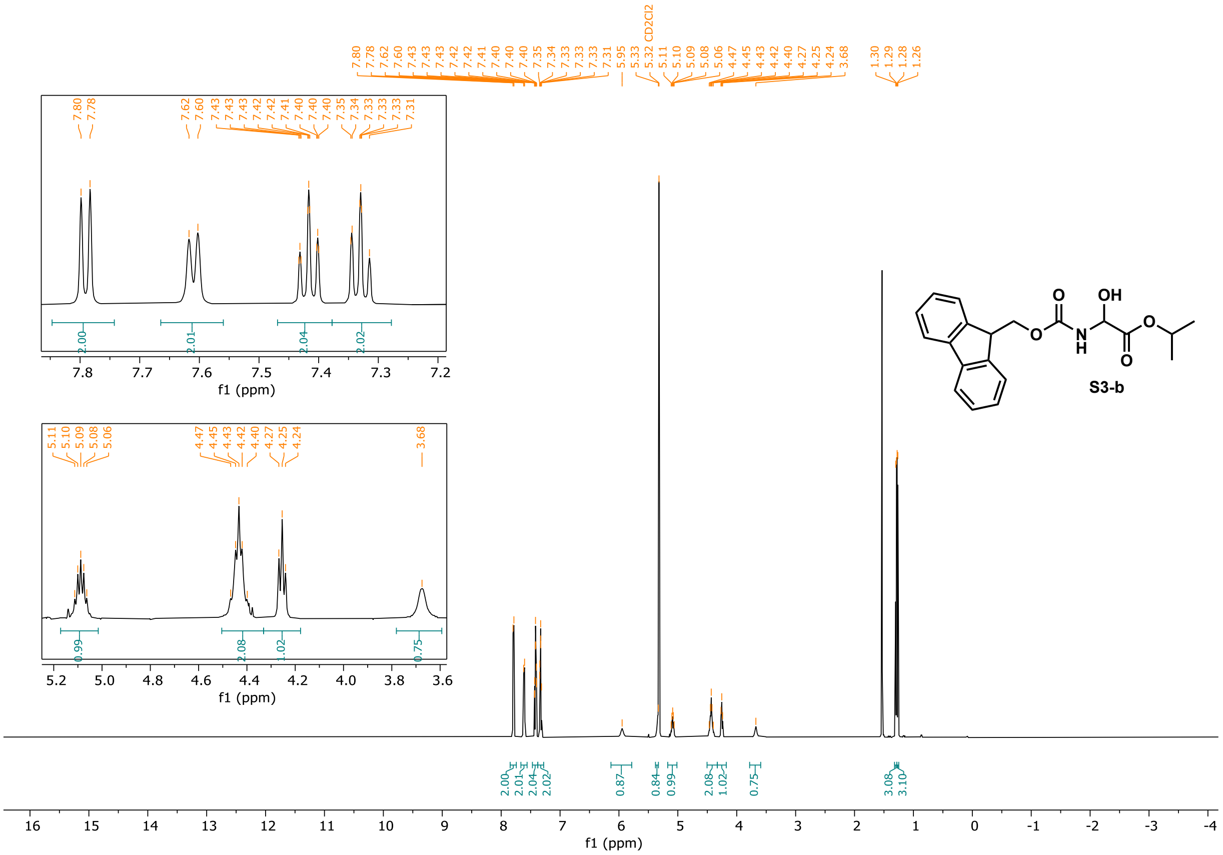

^{1}**H-NMR** spectrum of compound **S3-b**.

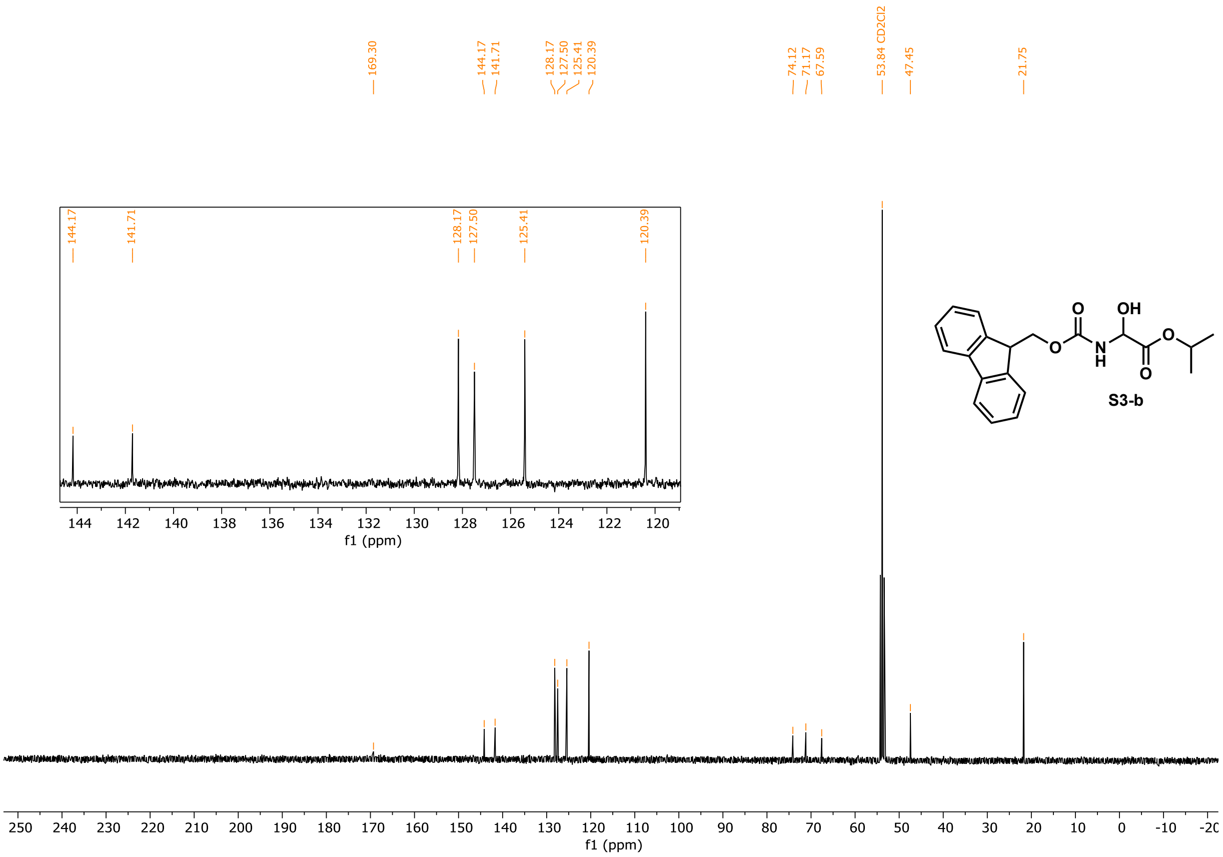

^{13}**-C-NMR** spectrum of compound **S3-b**.

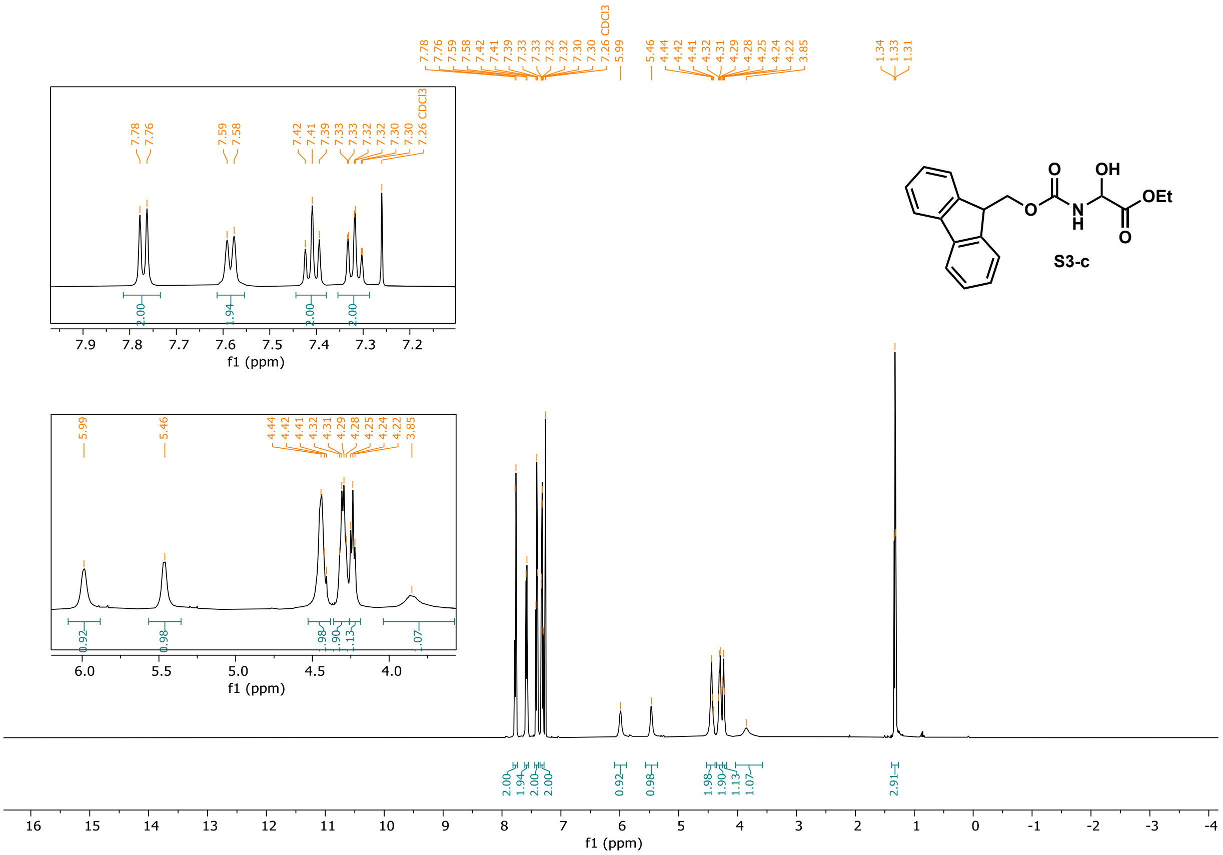

[1]**H-NMR** spectrum of compound **S3-c**.

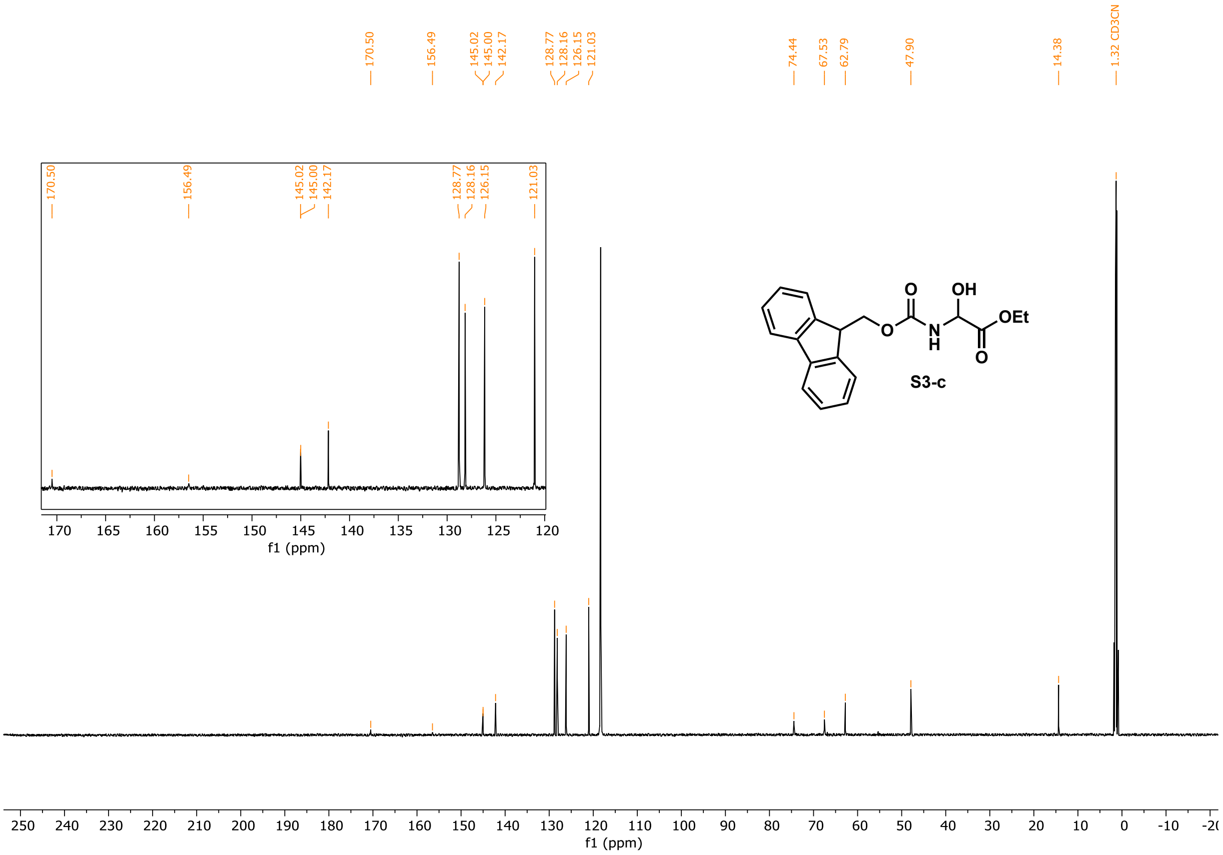

[13]**-C-NMR** spectrum of compound **S3-c**.

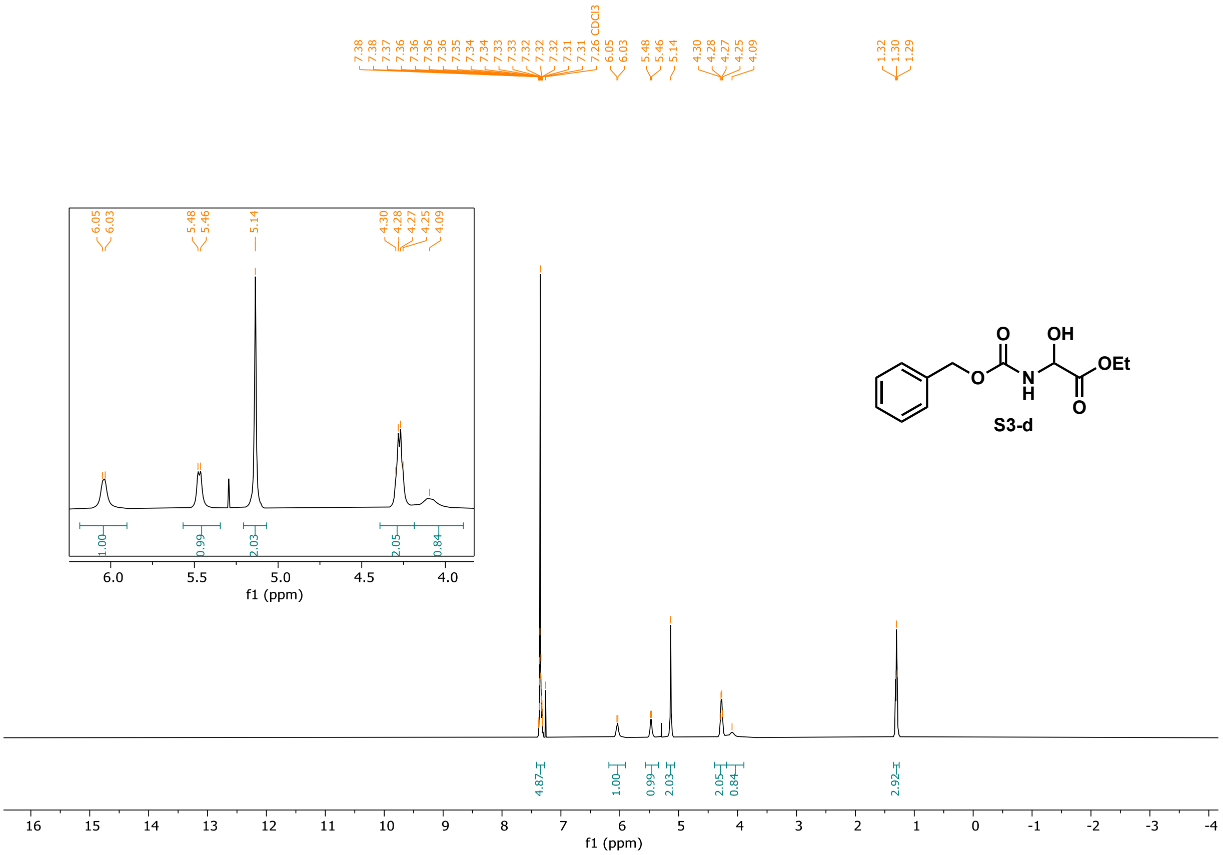

^{1}H-NMR spectrum of compound **S3-d**.

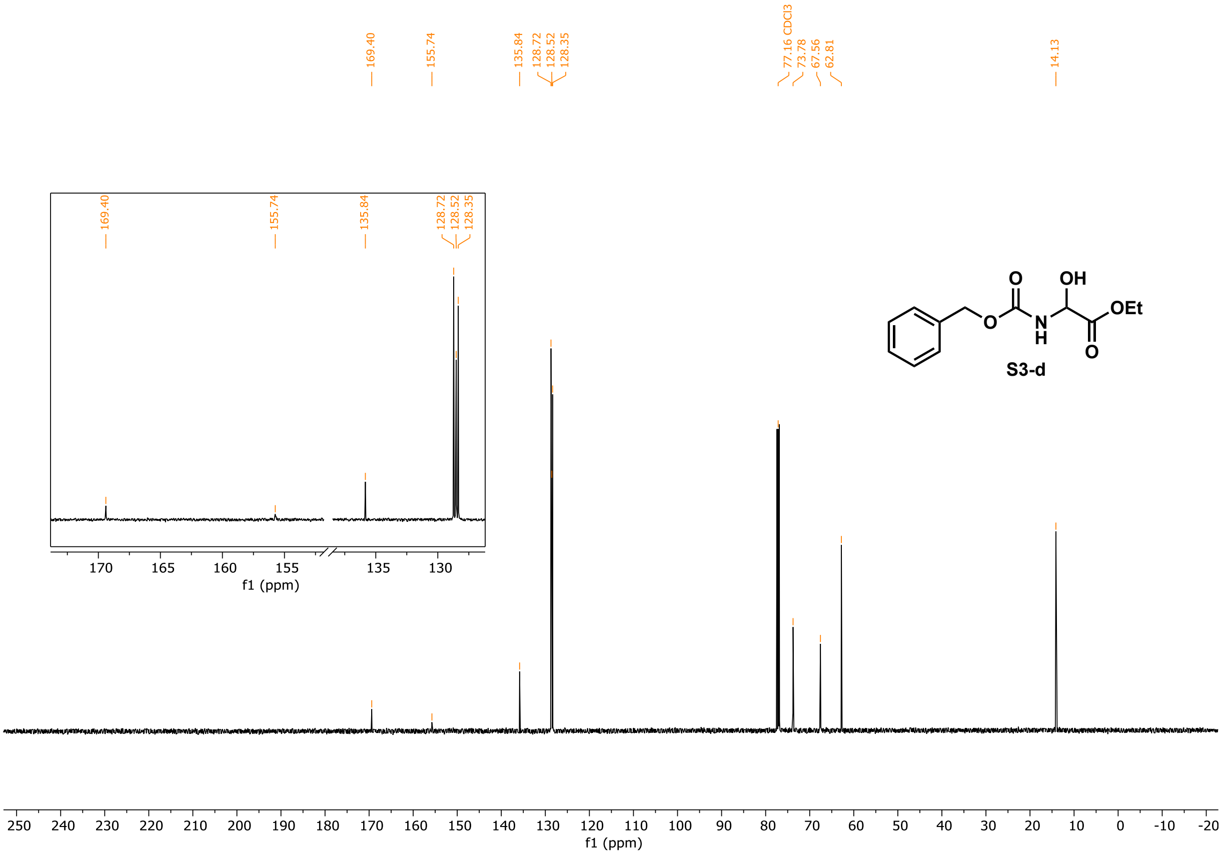

[13]-C-NMR spectrum of compound **S3-d**.

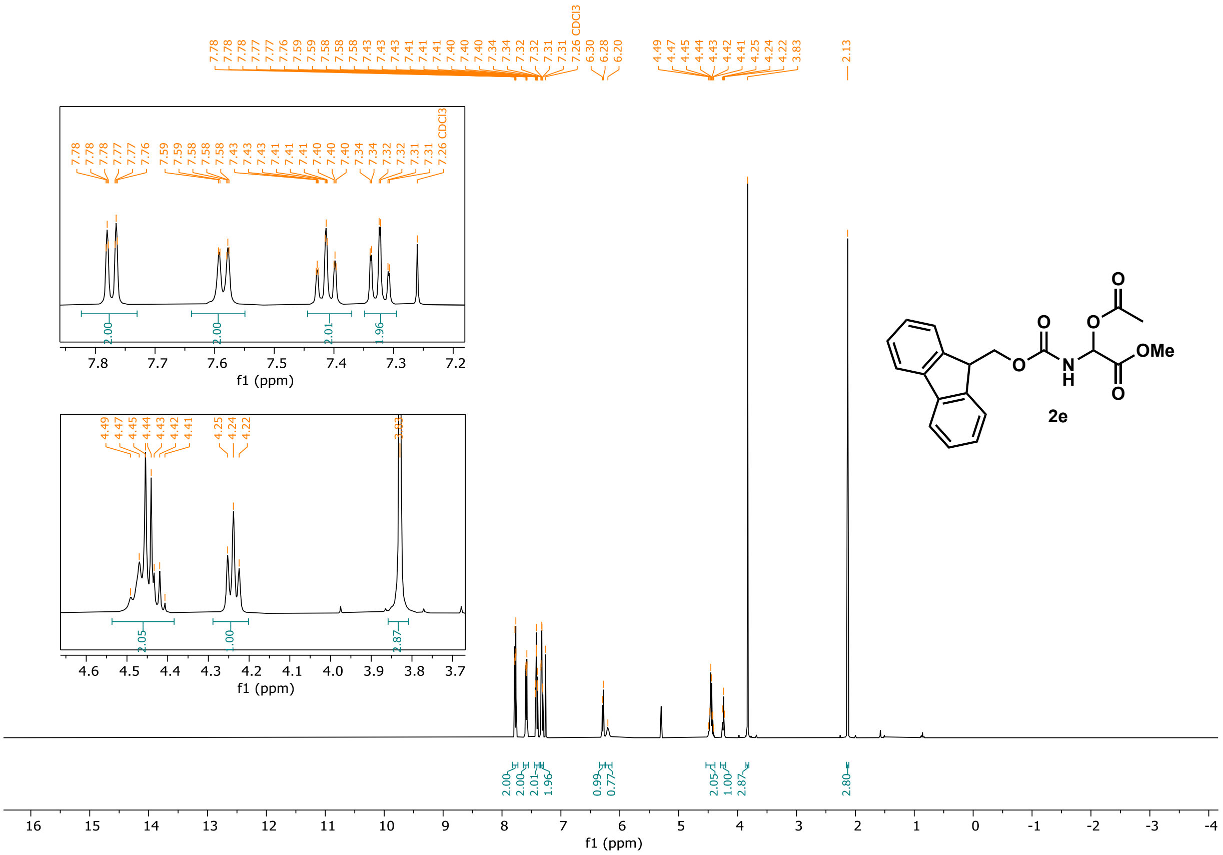

^{1}H-NMR spectrum of compound **2e**.

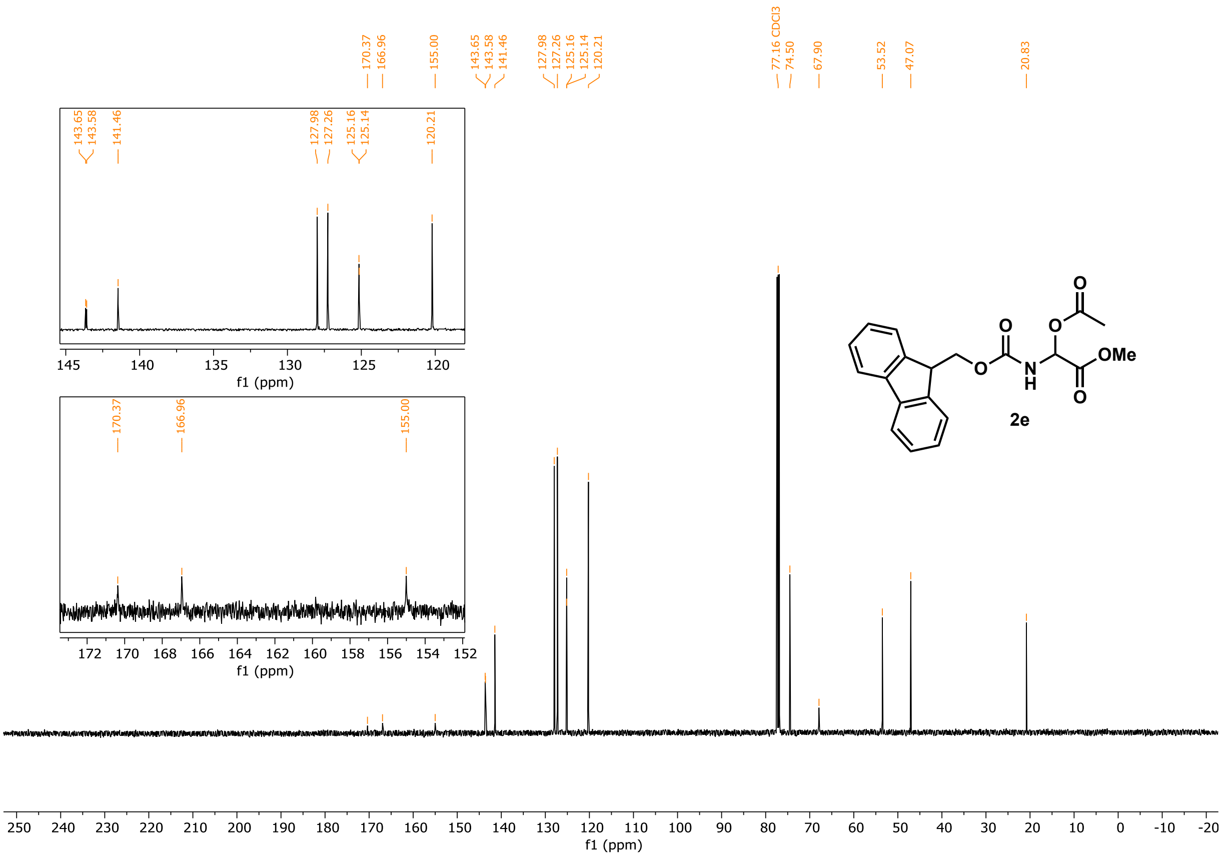

[13]-C-NMR spectrum of compound **2e**.

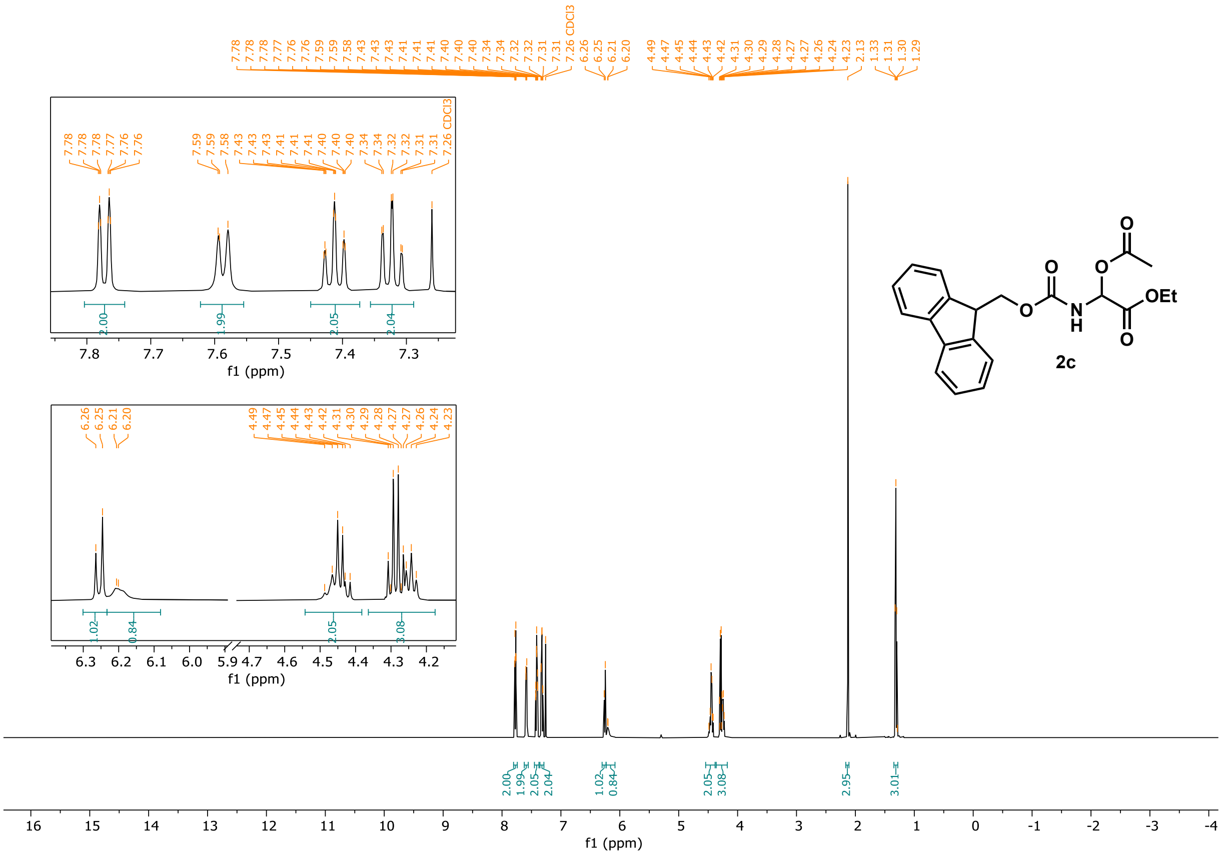

^{1}H-NMR spectrum of compound **2c**.

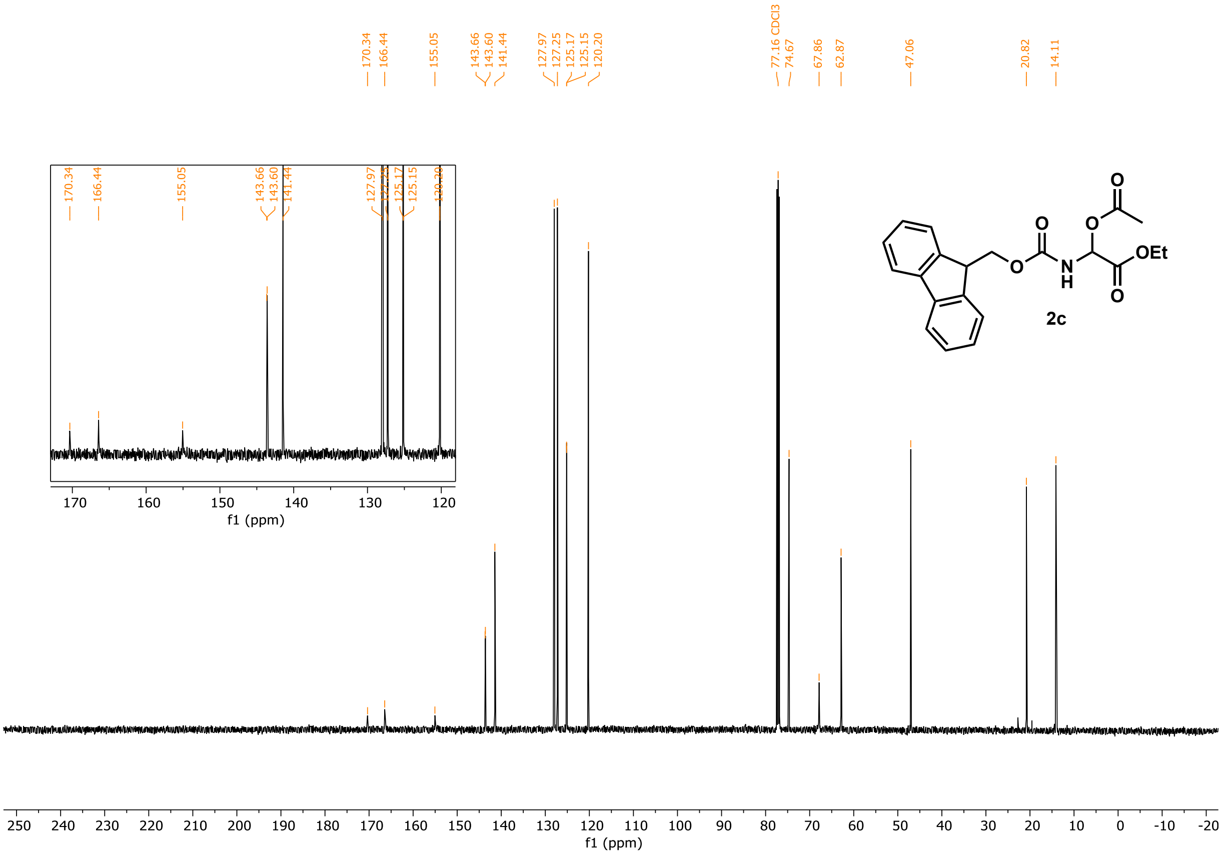

[13]-C-NMR spectrum of compound **2c**.

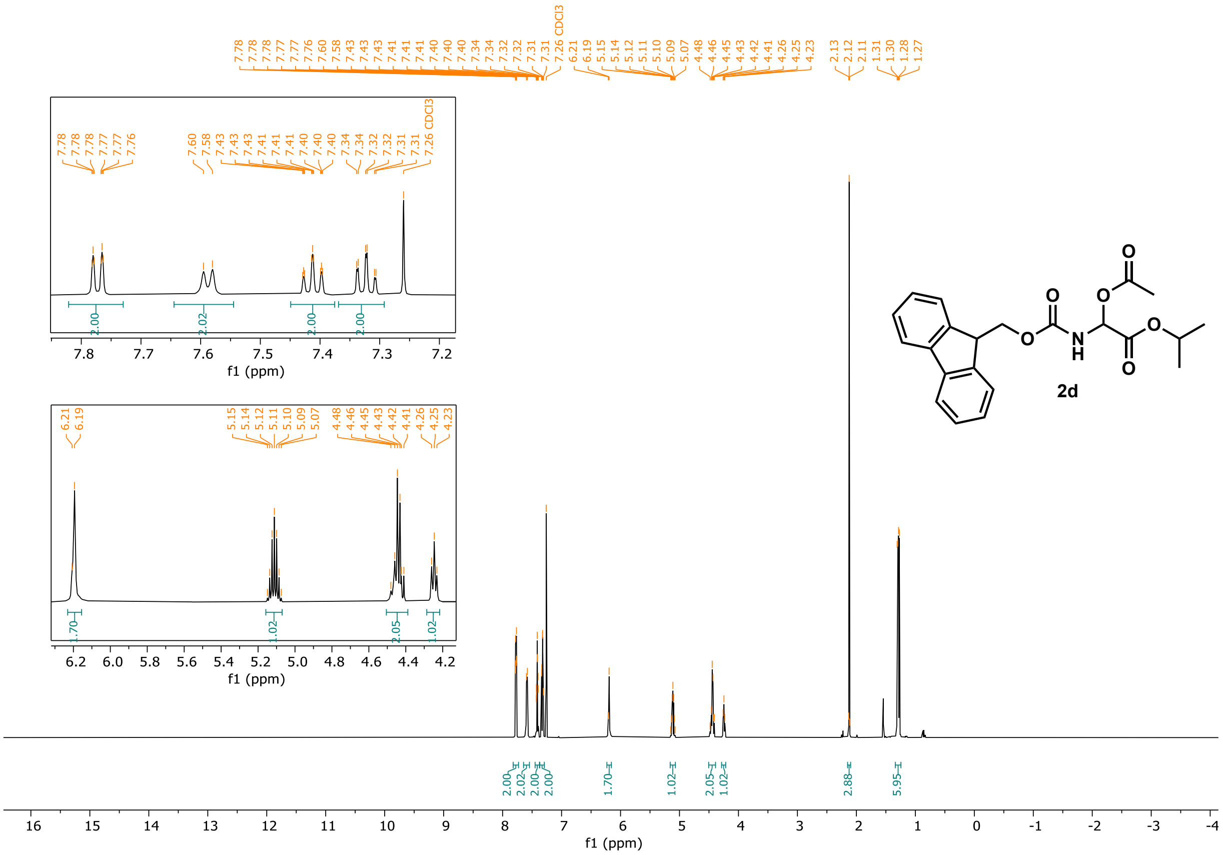

[1]**H-NMR** spectrum of compound **2d**.

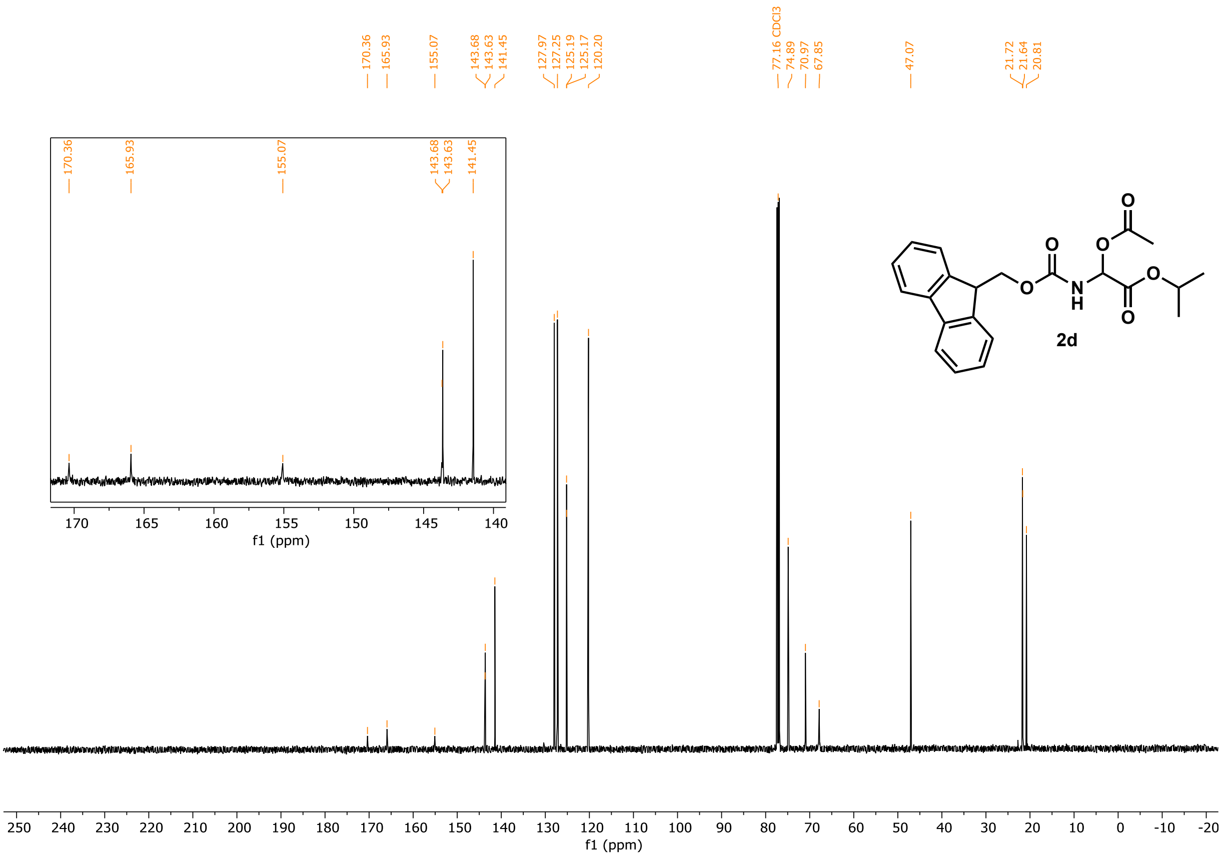

[13]**-C-NMR** spectrum of compound **2d**.

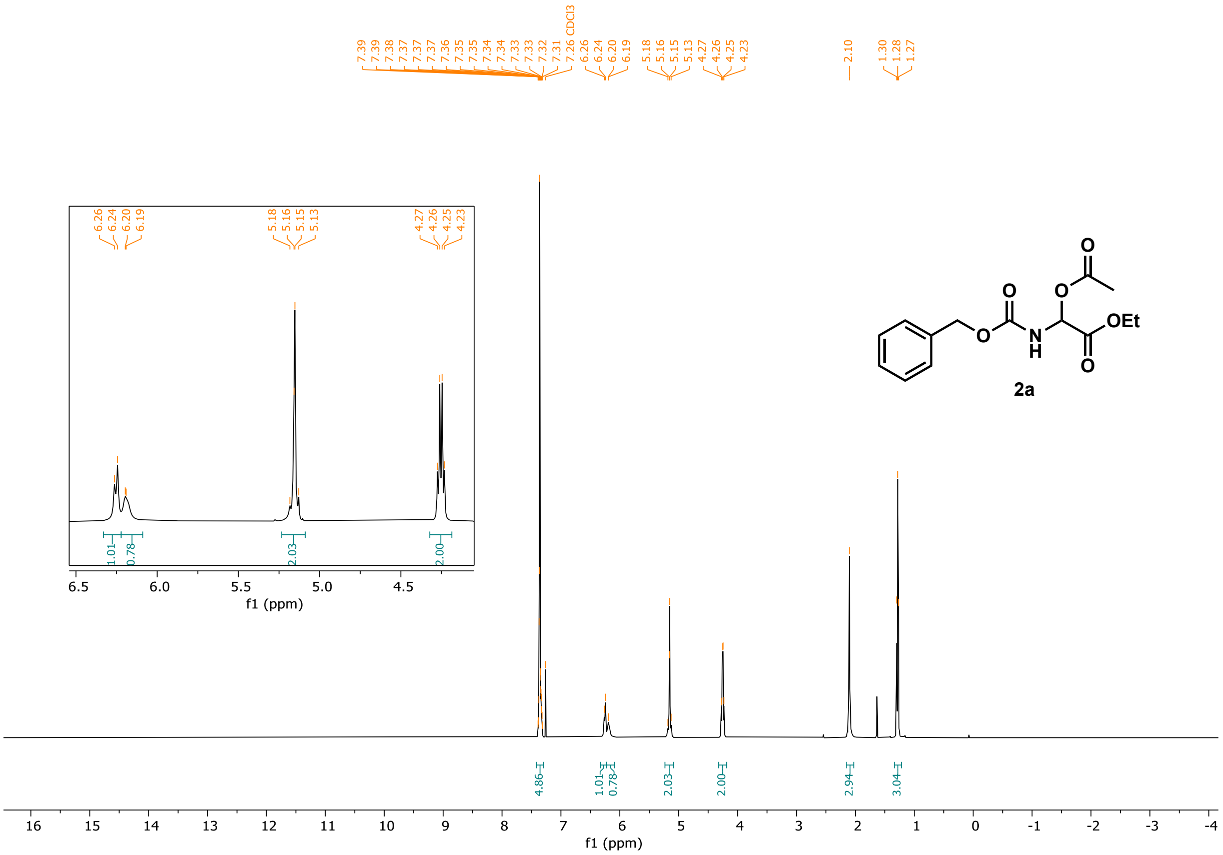

^{1}H-NMR spectrum of compound **2a**.

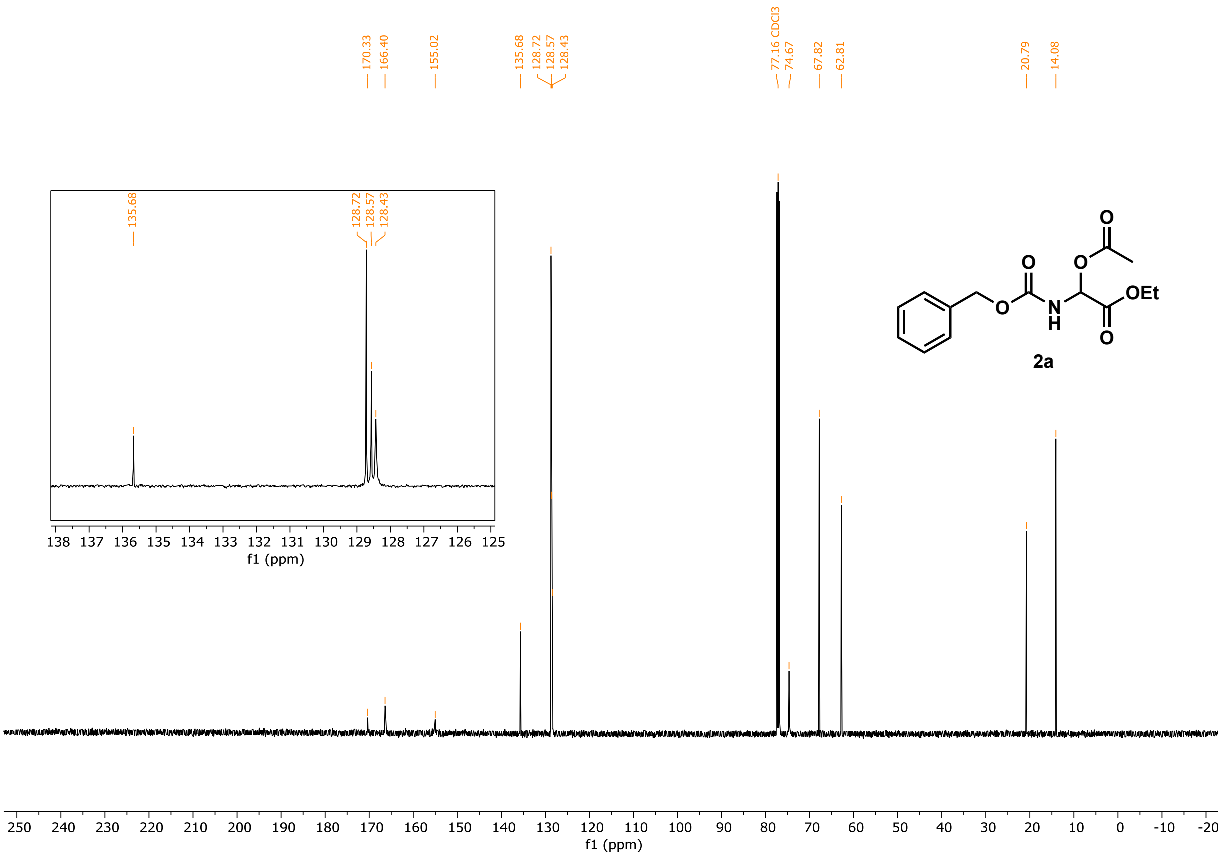

[13]-C-NMR spectrum of compound **2a**.

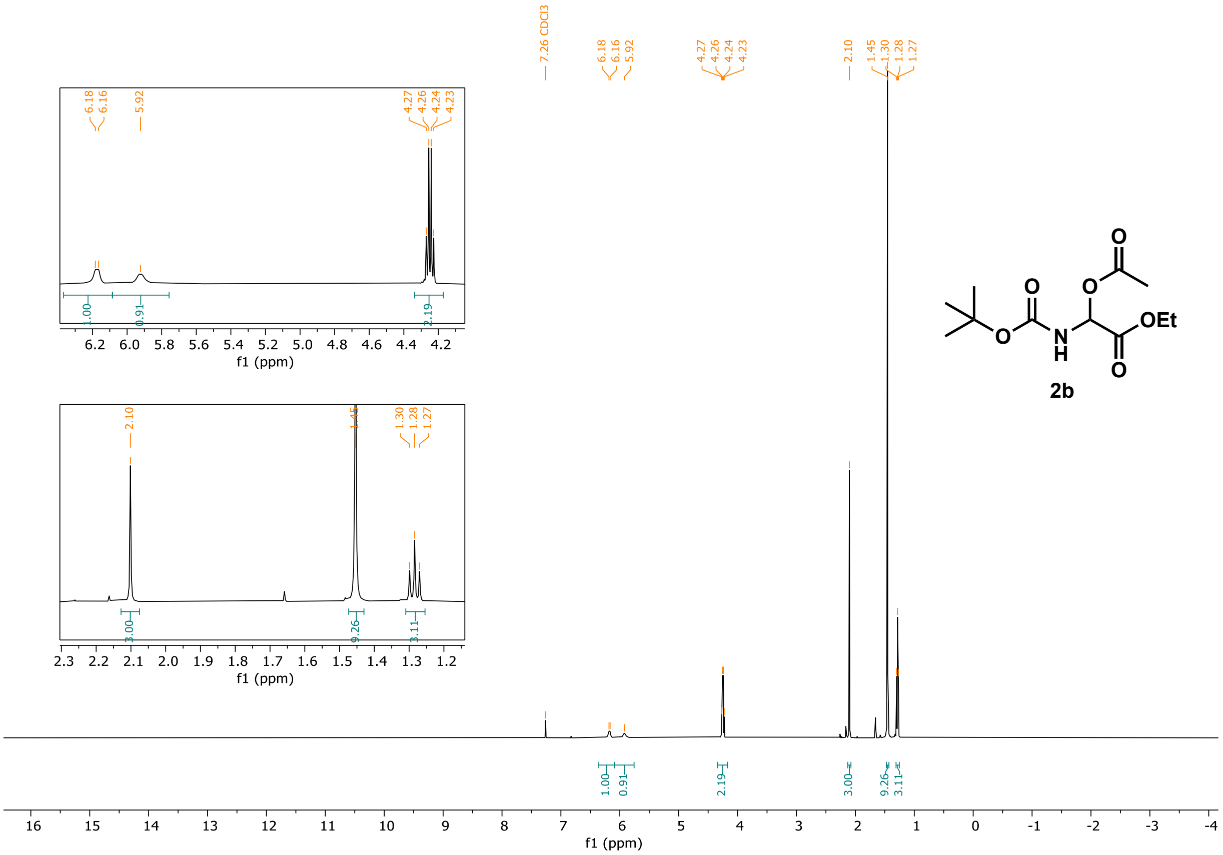

^{1}H-NMR spectrum of compound **2b**.

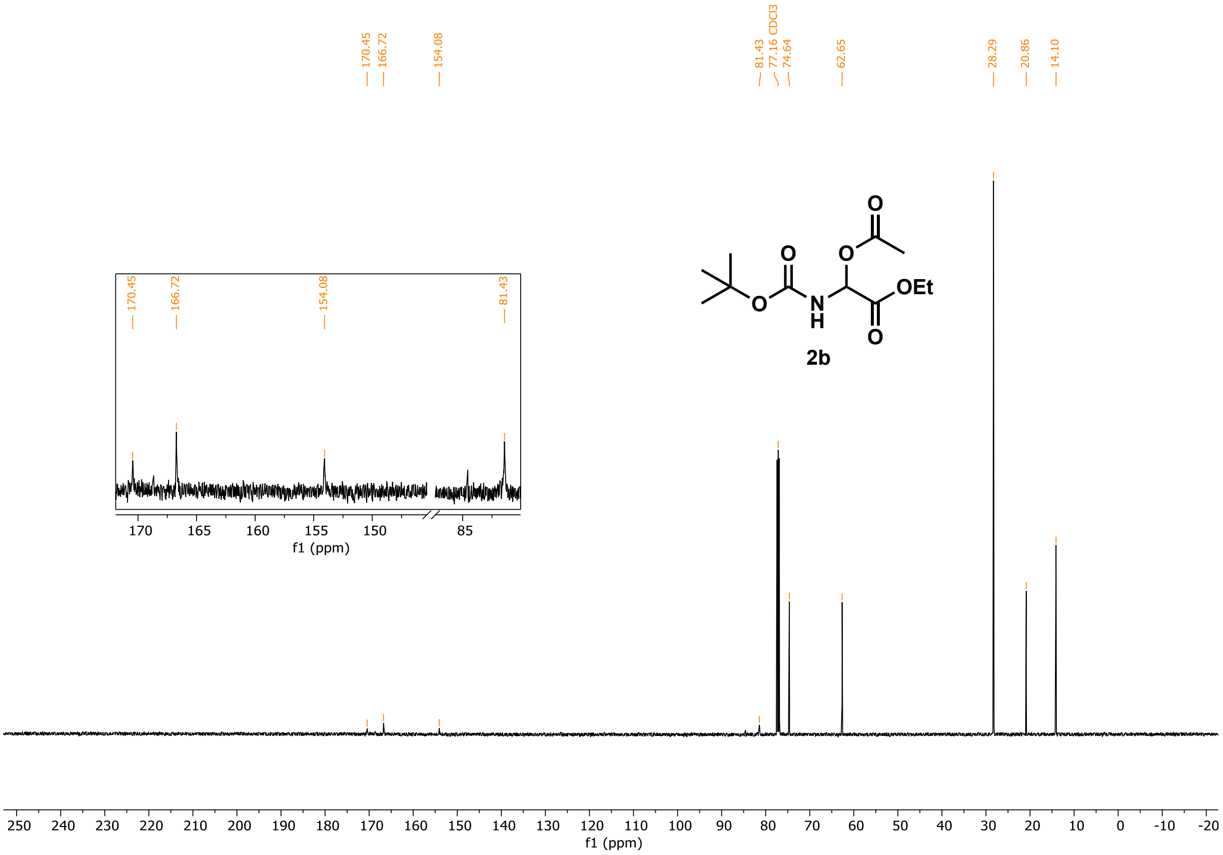

[13]-C-NMR spectrum of compound **2b**.

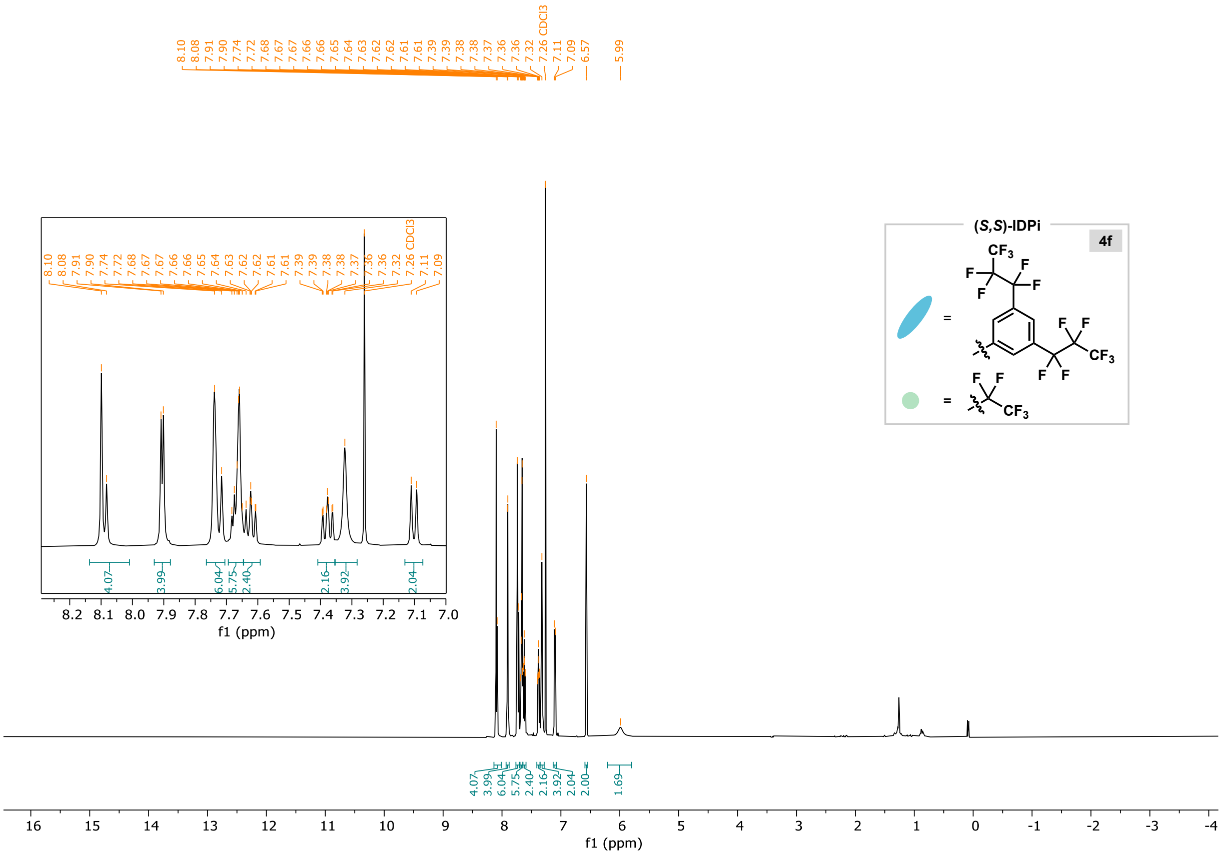

^{1}H-NMR spectrum of compound **4f**.

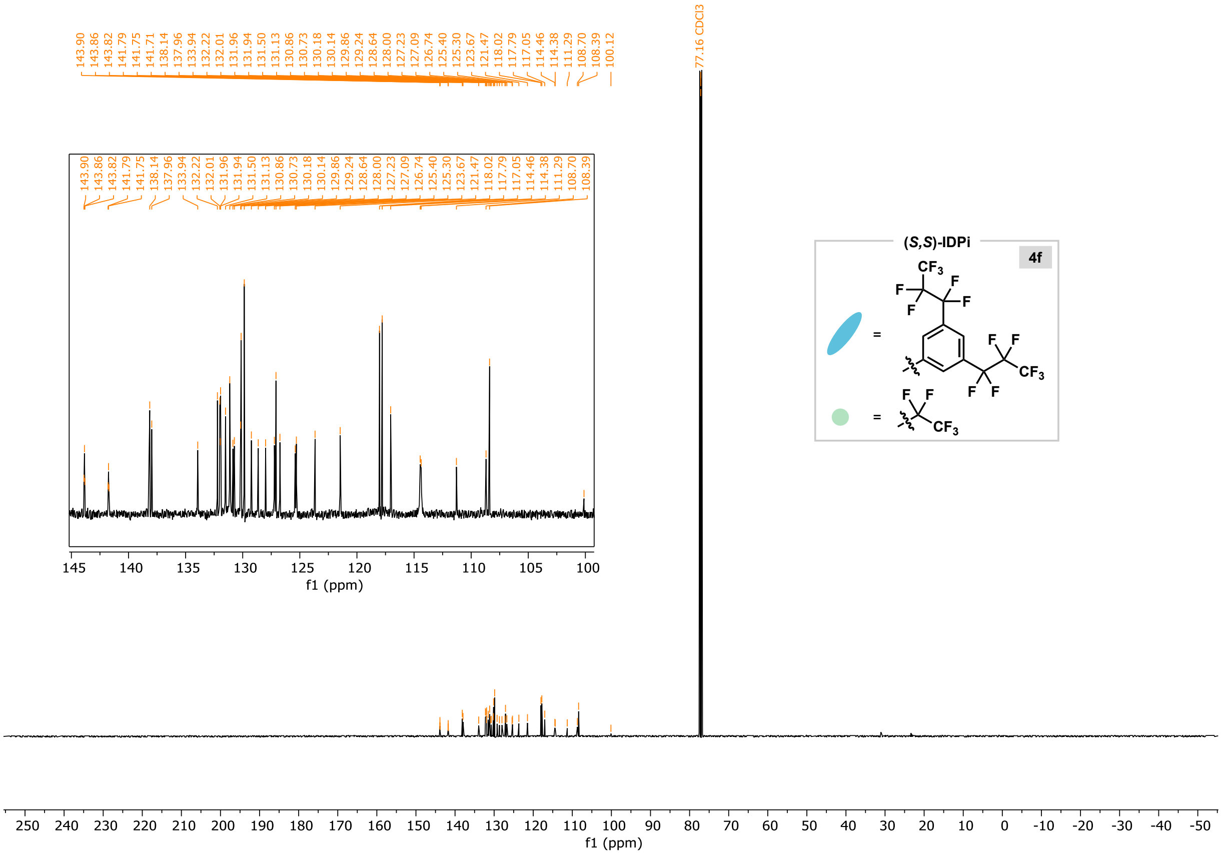

^{13}C{^{19}F}-NMR spectrum of compound **4f**.

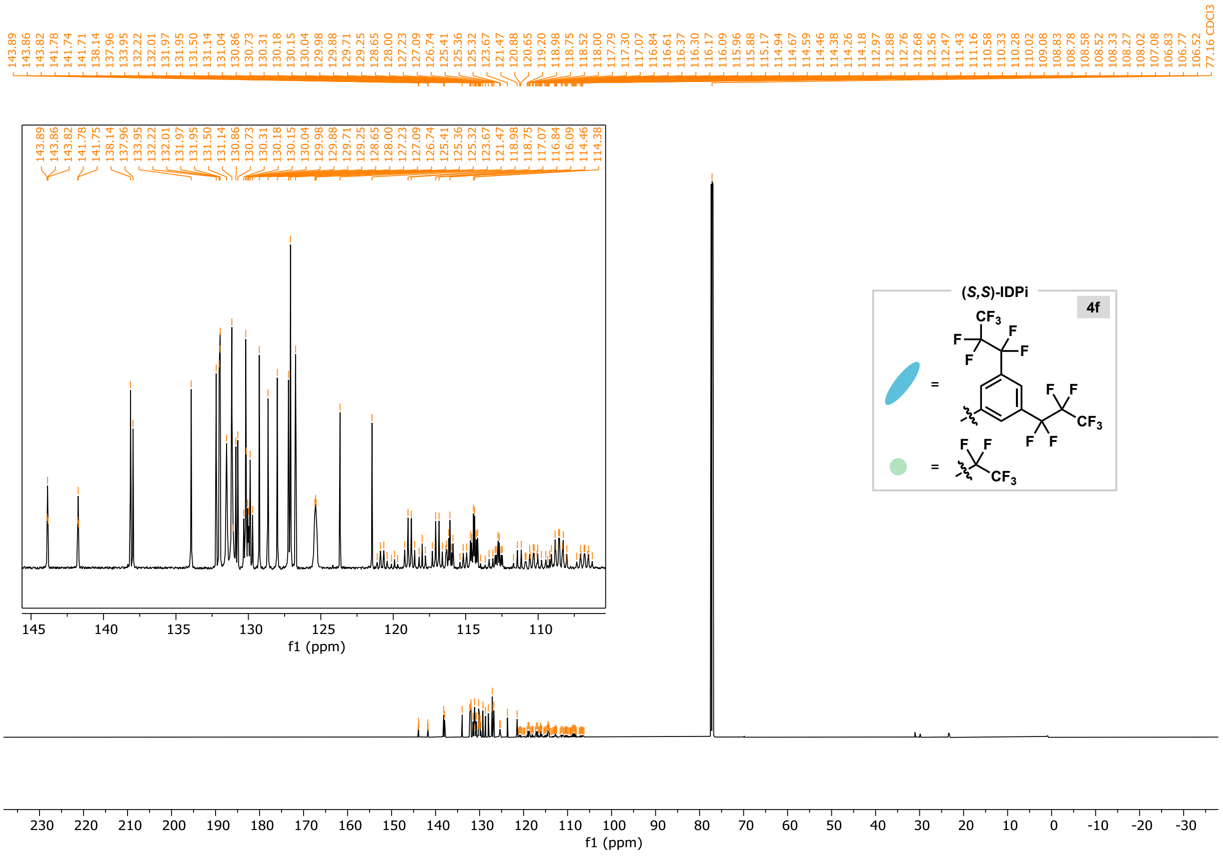

^{13}**C-NMR** spectrum of compound **4f**.

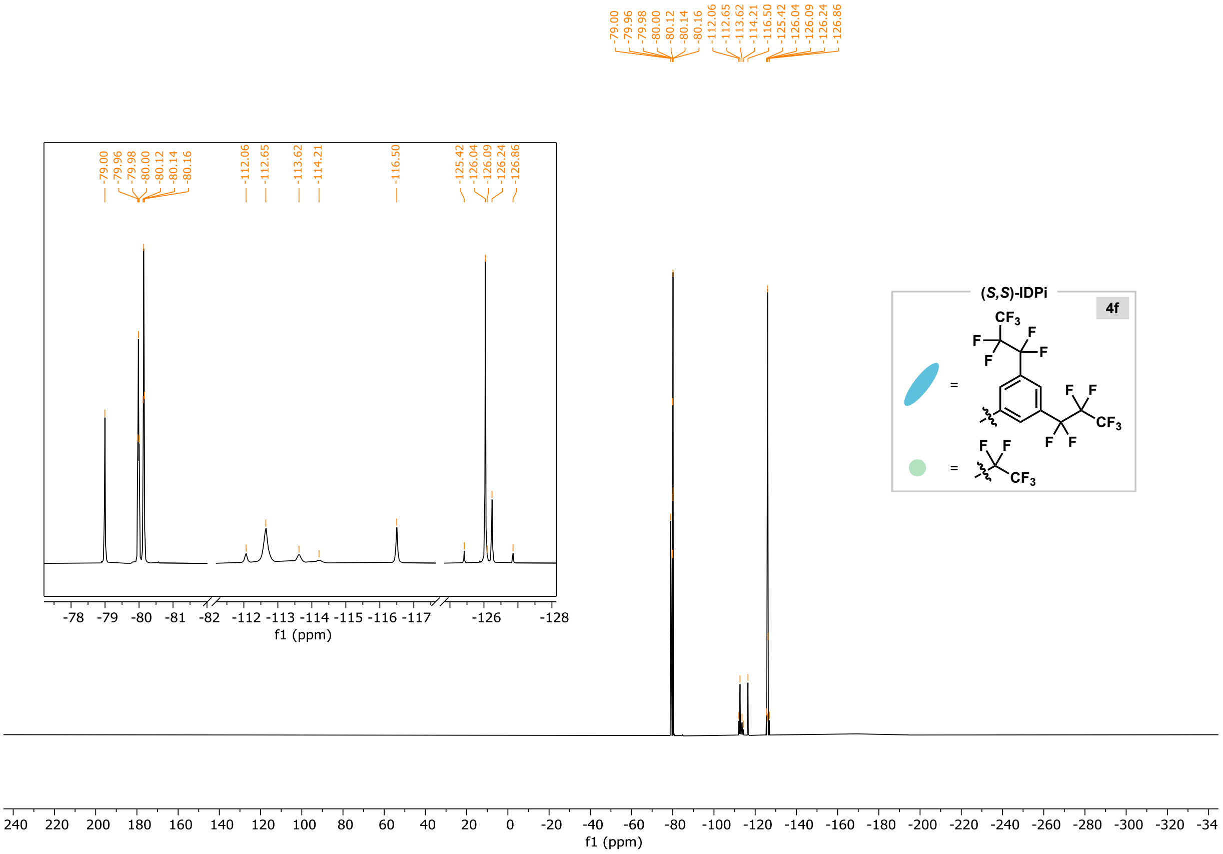

^{19}**F-NMR** spectrum of compound **4f**.

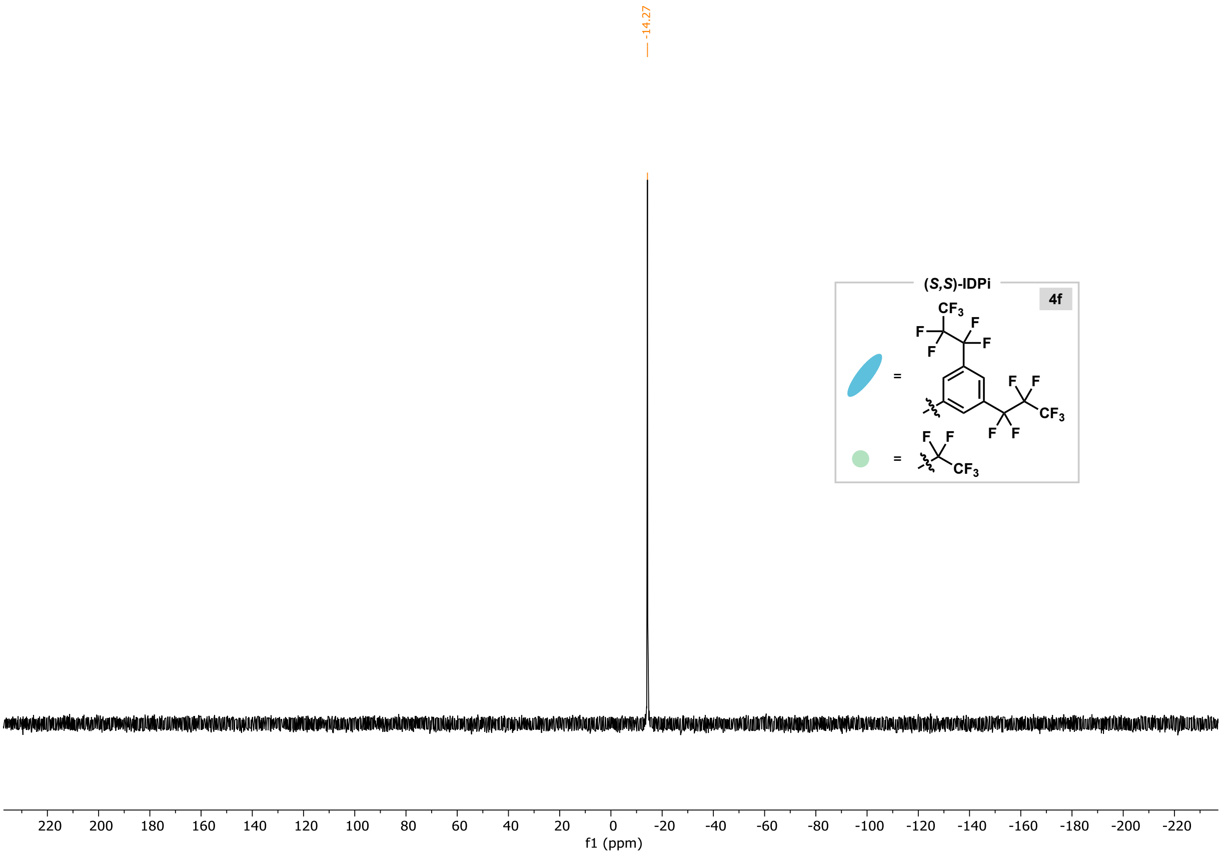

^{31}P-NMR spectrum of compound **4f**.

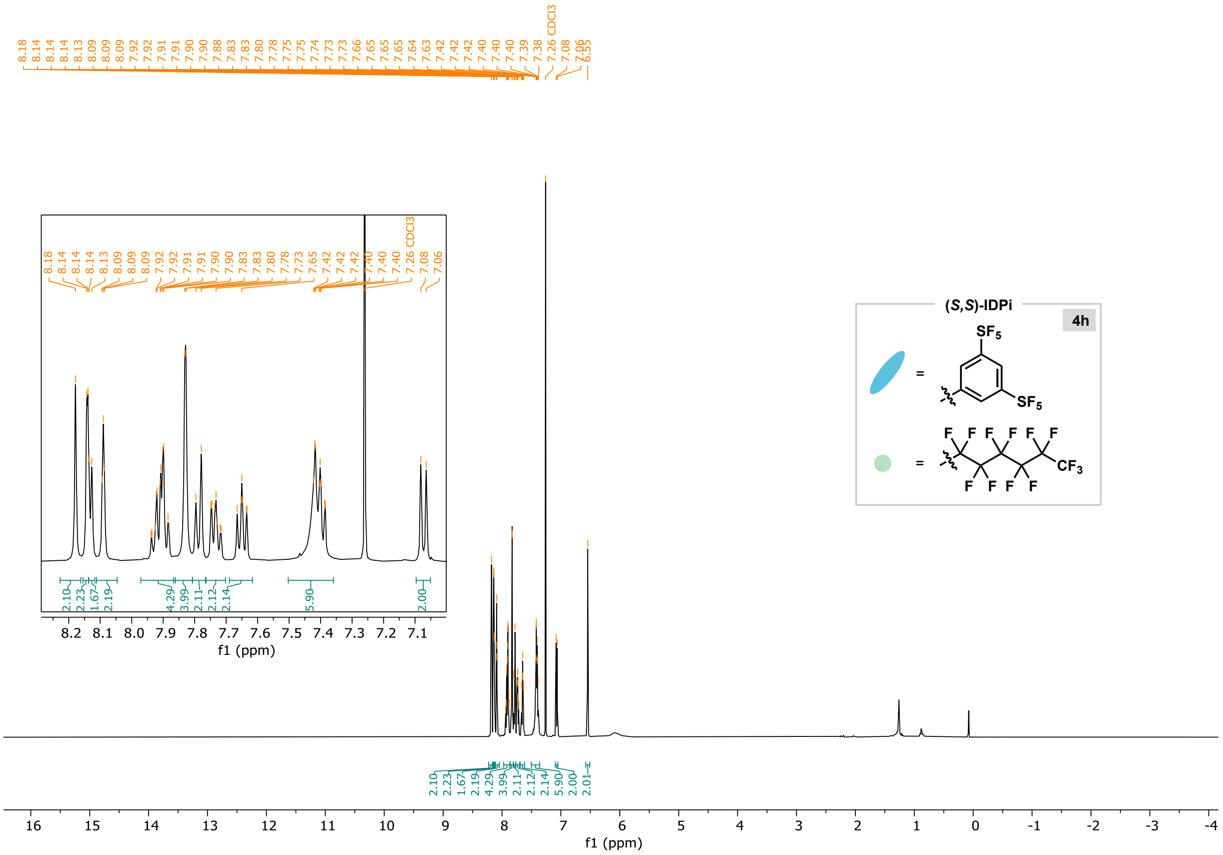

^{1}H-NMR spectrum of compound **4h**.

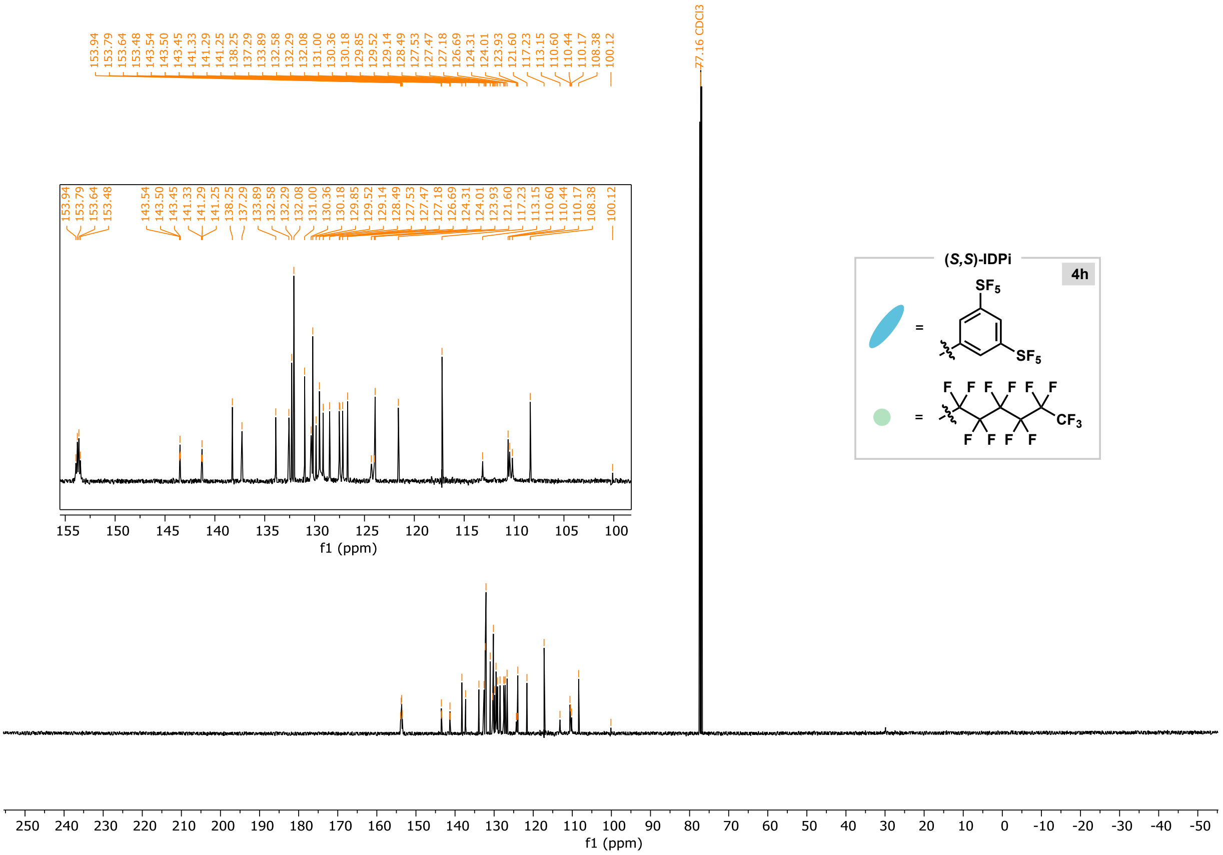

^{13}C{^{19}F}-NMR spectrum of compound **4h**.

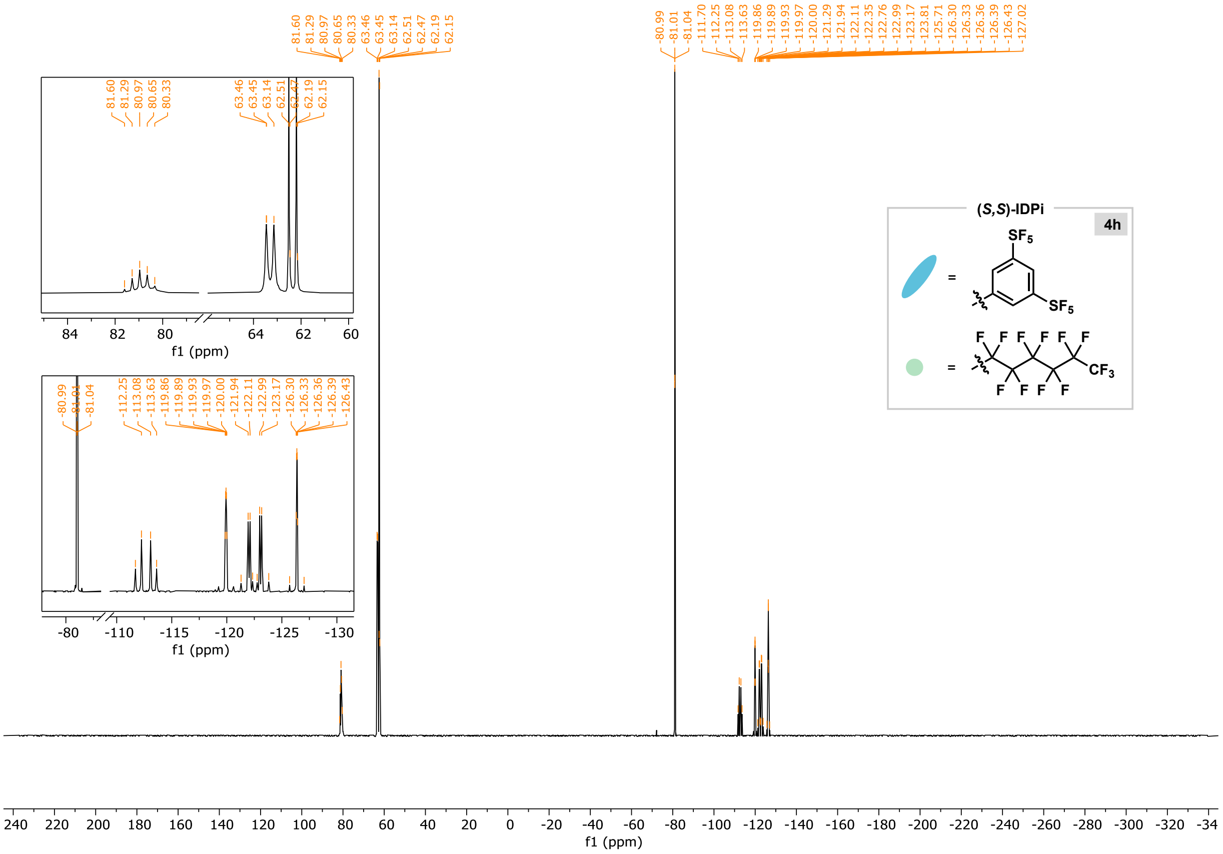

^{19}F-NMR spectrum of compound **4h**.

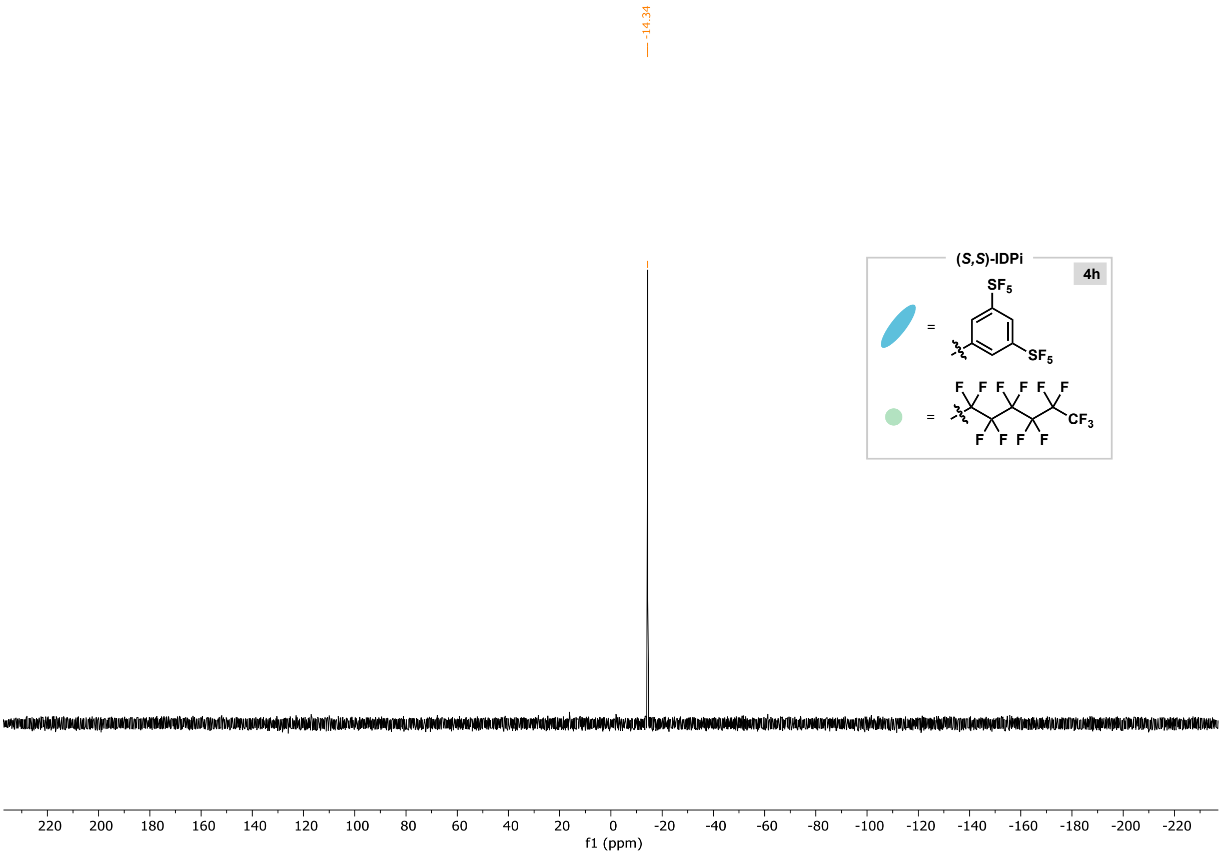

^{31}P-NMR spectrum of compound **4h**.

12. HPLC Traces

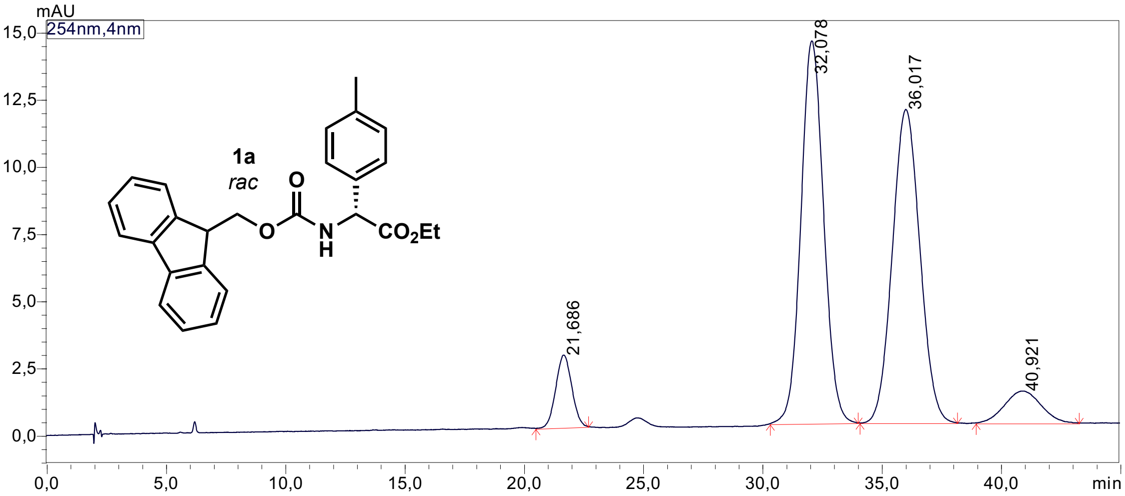

**1a-rac**: IG-3, *n*-heptane/*i*-PrOH 95:5, 298 K, 254 nm		
**peak #**	**t_R / min**	**area / %**
1 (minor regioisomer)	21.686	5.857
2 (major regioisomer)	32.078	44.341
3 (major regioisomer)	36.017	43.904
4 (minor regioisomer)	40.921	5.898

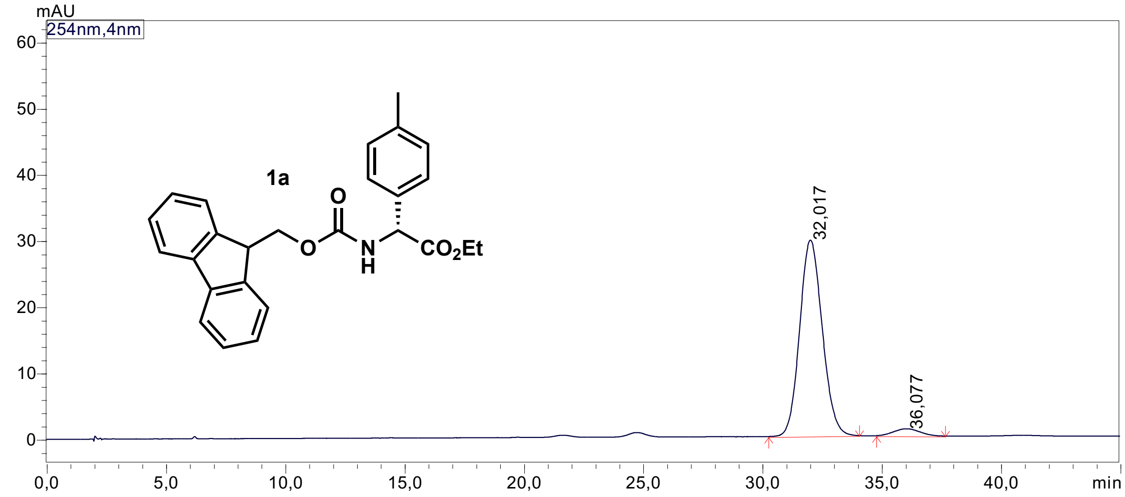

**1a**: IG-3, *n*-heptane/*i*-PrOH 95:5, 298 K, 254 nm		
**peak #**	**t_R / min**	**area / %**
1	32.017	95.943
2	36.077	4.057

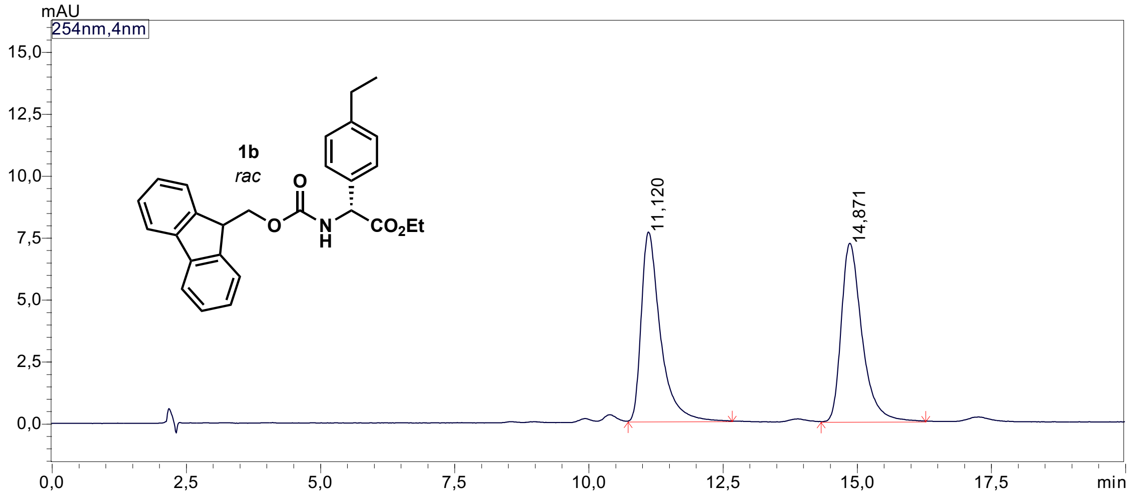

**1b-rac**: IB-3, *n*-heptane/*i*-PrOH 95:5, 298 K, 254 nm		
**peak #**	$\boldsymbol{t_R}$ **/ min**	**area / %**
1	11.120	49.486
2	14.871	50.514

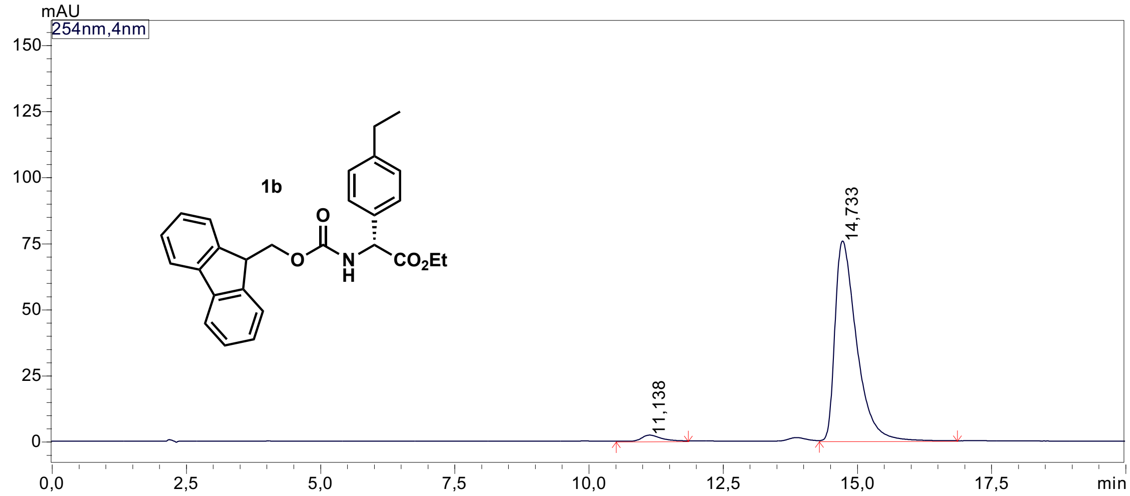

**1b**: IB-3, *n*-heptane/*i*-PrOH 95:5, 298 K, 254 nm		
**peak #**	$\boldsymbol{t_R}$ **/ min**	**area / %**
1	11.138	2.401
2	14.733	97.599

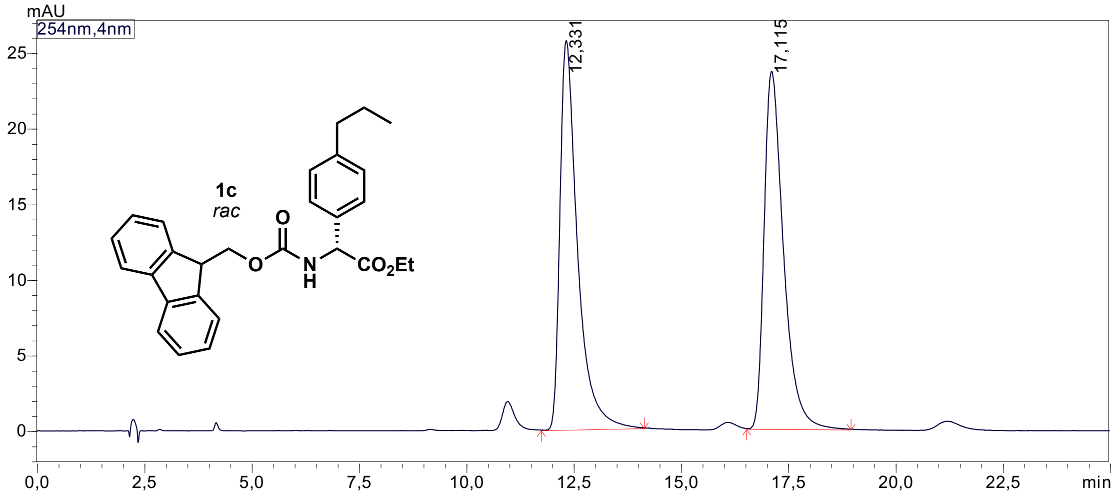

**1c-rac**: IB-3, *n*-heptane/*i*-PrOH 96:4, 298 K, 254 nm		
**peak #**	**t_R / min**	**area / %**
1	12.331	49.695
2	17.115	50.305

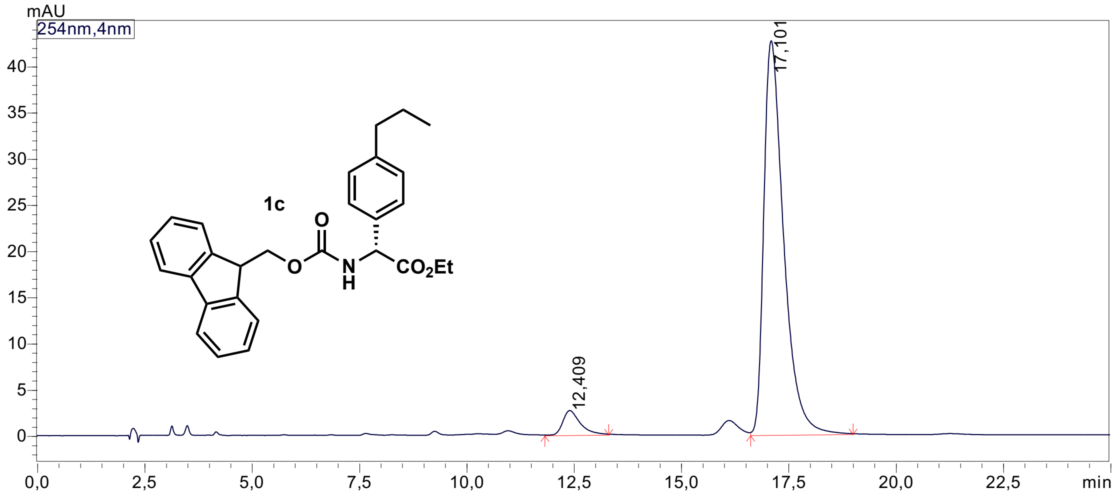

**1c**: IB-3, *n*-heptane/*i*-PrOH 96:4, 298 K, 254 nm		
**peak #**	**t_R / min**	**area / %**
1	12.409	4.945
2	17.101	95.055

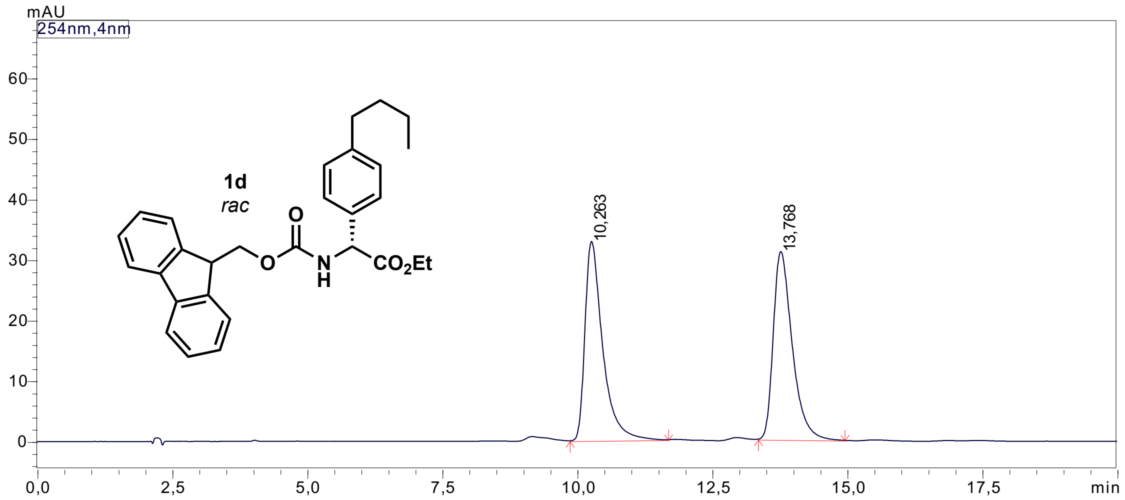

**1d-rac**: IB-3, *n*-heptane/*i*-PrOH 95:5, 298 K, 254 nm		
**peak #**	**t_R / min**	**area / %**
1	10.263	49.713
2	13.768	50.287

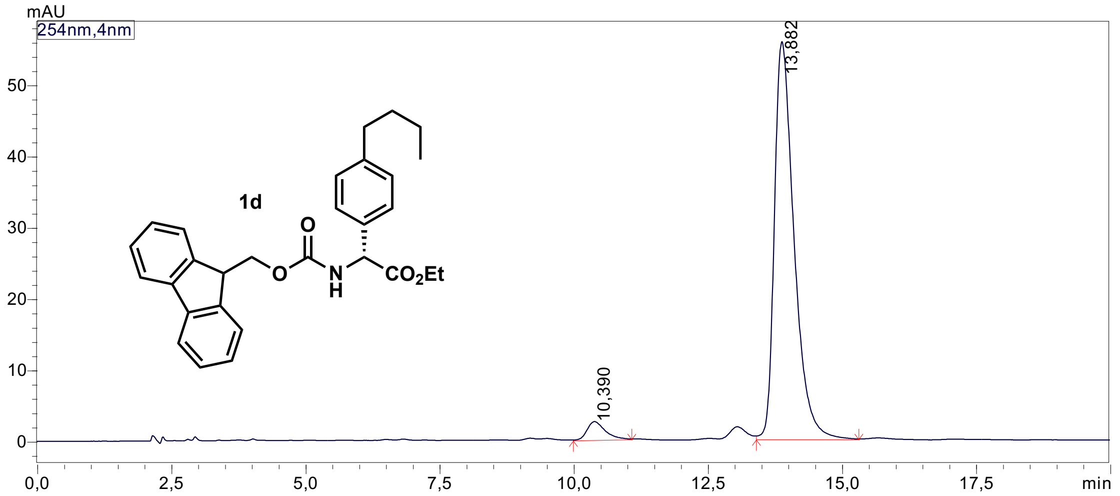

**1d**: IB-3, *n*-heptane/*i*-PrOH 95:5, 298 K, 254 nm		
**peak #**	**t_R / min**	**area / %**
1	10.390	3.917
2	13.882	96.083

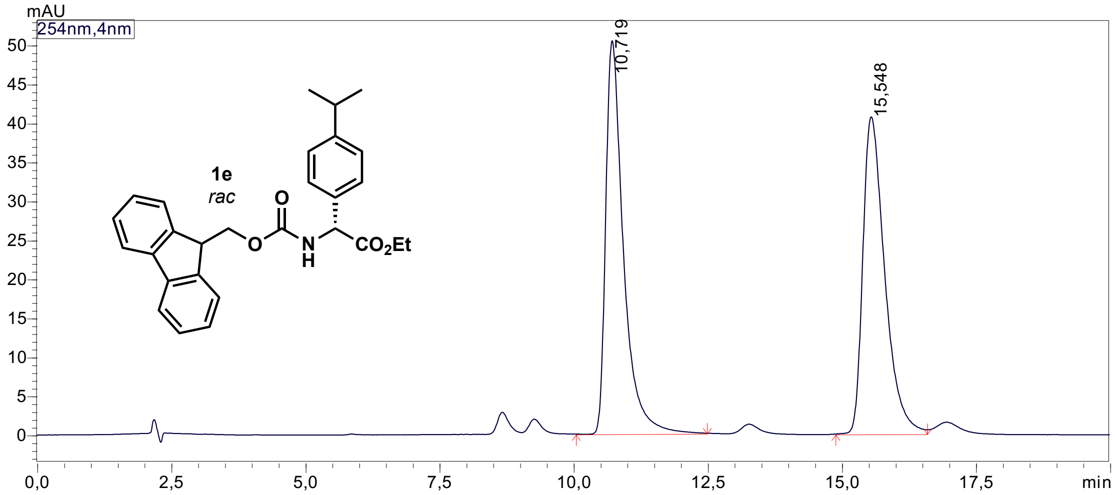

**1e-rac**: IB-3, *n*-heptane/*i*-PrOH 95:5, 298 K, 254 nm		
**peak #**	$\boldsymbol{t_R}$ **/ min**	**area / %**
1	10.719	49.965
2	15.548	50.035

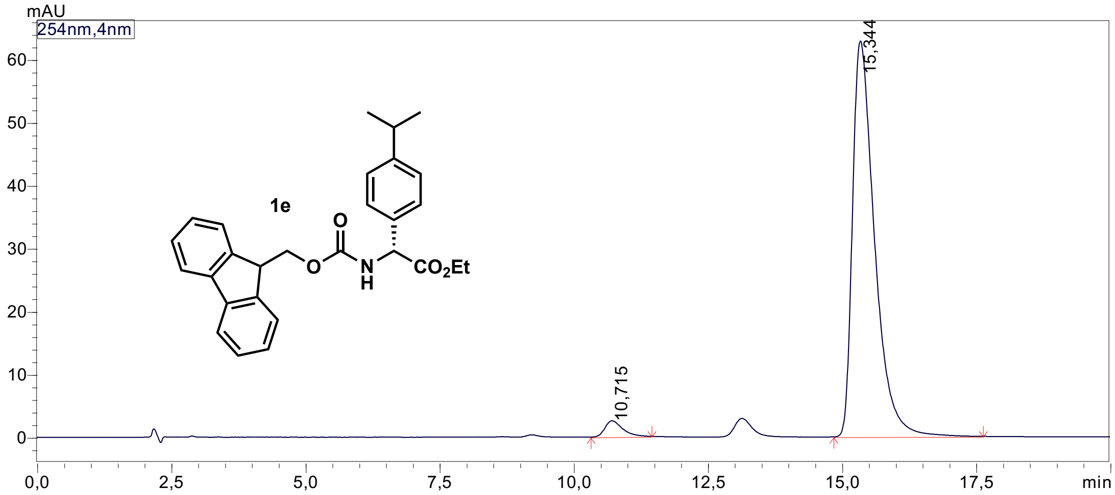

**1e**: IB-3, *n*-heptane/*i*-PrOH 95:5, 298 K, 254 nm		
**peak #**	$\boldsymbol{t_R}$ **/ min**	**area / %**
1	10.715	3.118
2	15.344	96.882

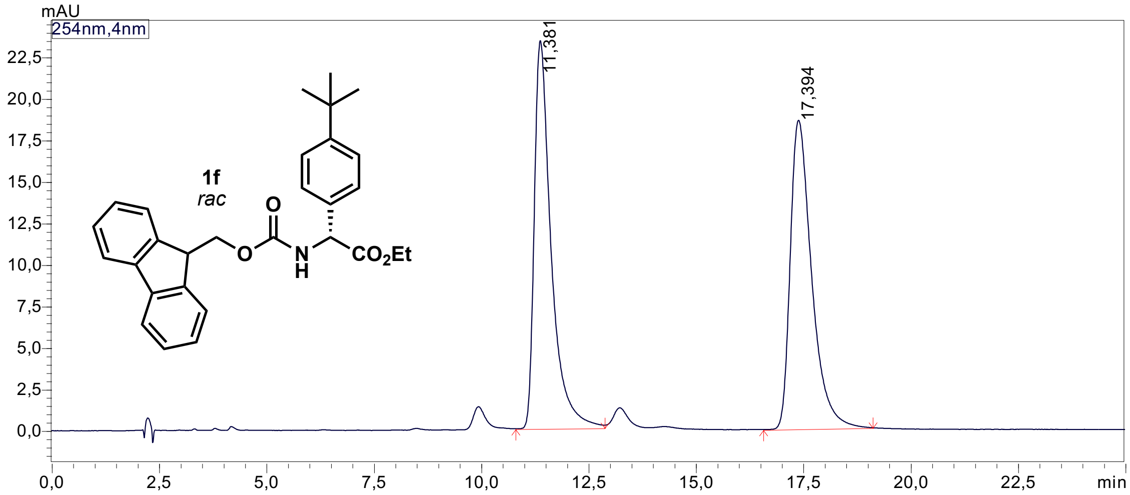

**1f-rac**: IB-3, *n*-heptane/*i*-PrOH 96:4, 298 K, 254 nm		
**peak #**	***t*$_R$ / min**	**area / %**
1	11.381	49.749
2	17.394	50.251

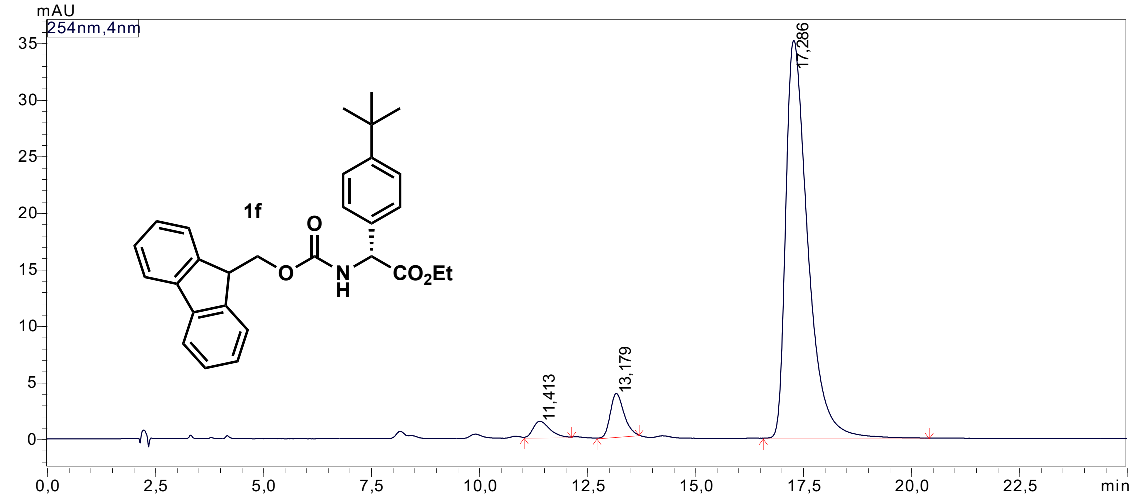

**1f**: IB-3, *n*-heptane/*i*-PrOH 96:4, 298 K, 254 nm		
**peak #**	***t*$_R$ / min**	**area / %**
1	11.413	2.620
2 (minor regioisomer)	13.179	6.500
3	17.286	90.880

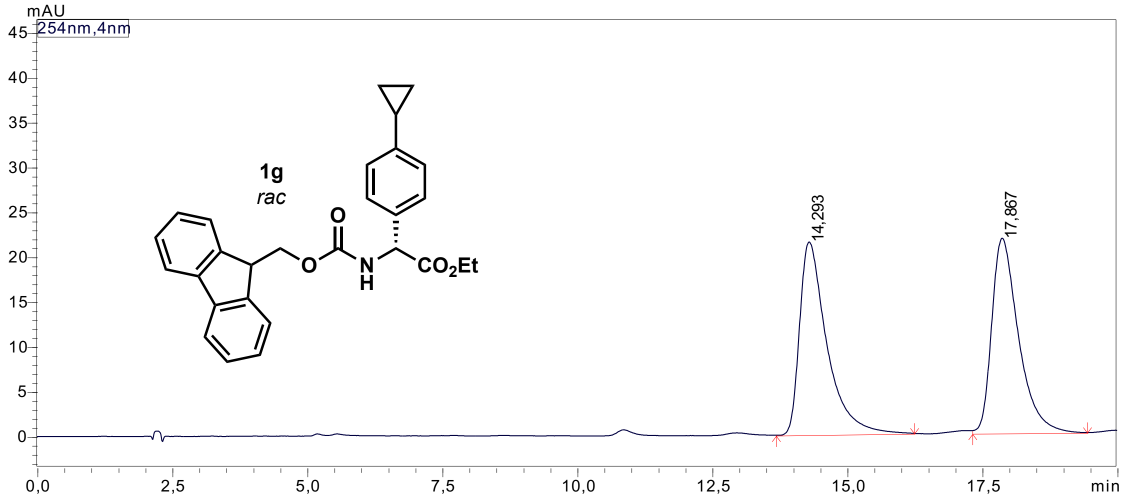

**1g-rac**: IB-3, *n*-heptane/*i*-PrOH 95:5, 298 K, 254 nm		
**peak #**	**t_R / min**	**area / %**
1	14.293	50.249
2	17.867	49.751

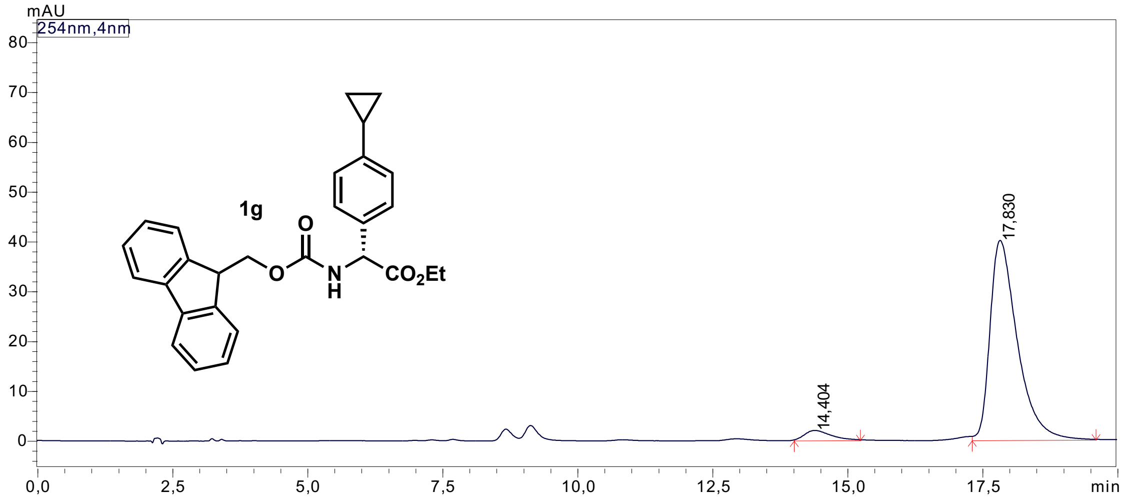

**1g**: IB-3, *n*-heptane/*i*-PrOH 95:5, 298 K, 254 nm		
**peak #**	**t_R / min**	**area / %**
1	14.404	4.219
2	17.830	95.781

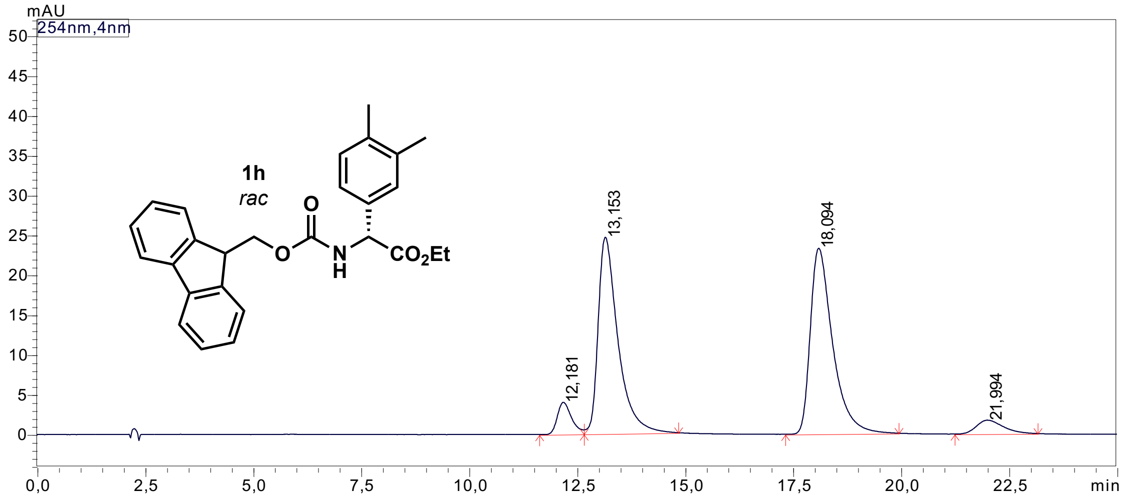

**1h-rac**: IB-3, *n*-heptane/*i*-PrOH 96:4, 298 K, 254 nm		
**peak #**	$\boldsymbol{t_R}$ **/ min**	**area / %**
1 (minor regioisomer)	12.181	5.078
2 (major regioisomer)	13.153	44.001
3 (major regioisomer)	18.094	46.814
4 (minor regioisomer)	21.994	4.107

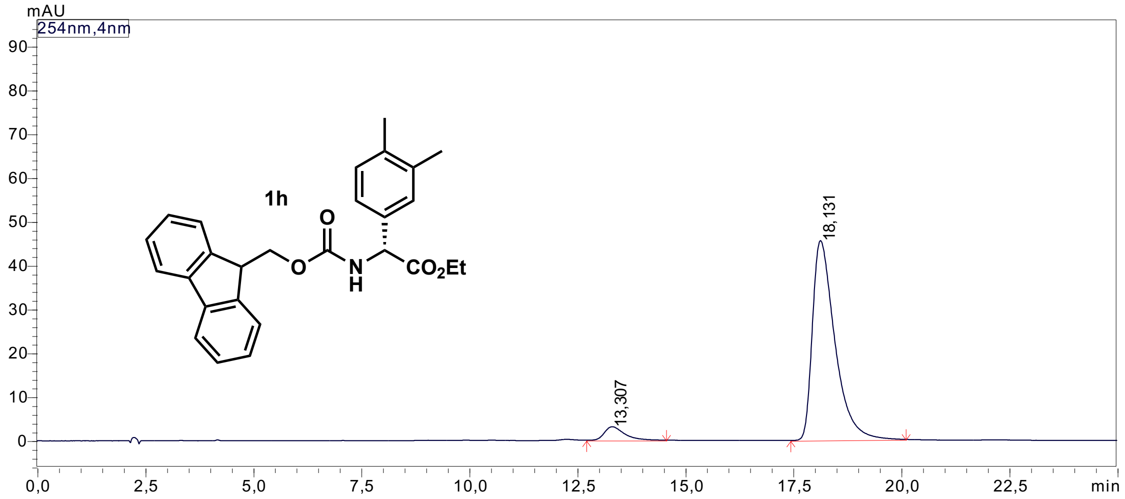

**1h**: IB-3, *n*-heptane/*i*-PrOH 96:4, 298 K, 254 nm		
**peak #**	$\boldsymbol{t_R}$ **/ min**	**area / %**
1	13.307	5.665
2	18.131	94.335

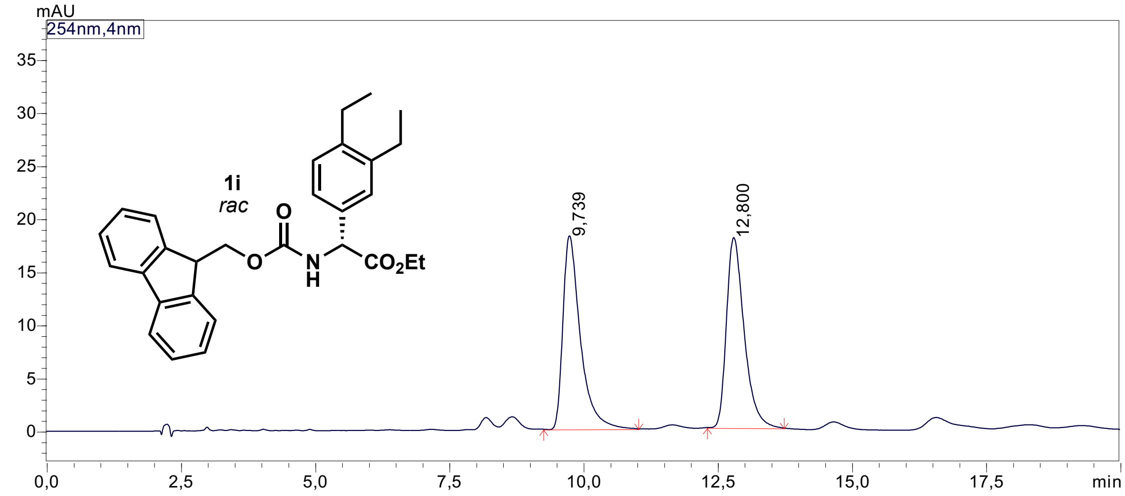

**1i-rac**: IB-3, *n*-heptane/*i*-PrOH 95:5, 298 K, 254 nm		
**peak #**	**t_R / min**	**area / %**
1	9.739	49.977
2	12.800	50.023

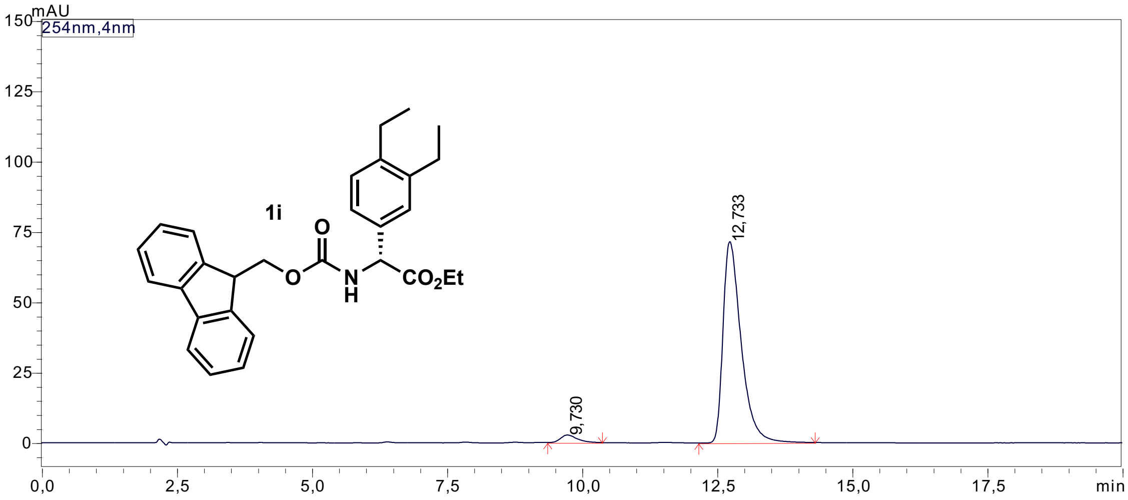

**1i**: IB-3, *n*-heptane/*i*-PrOH 95:5, 298 K, 254 nm		
**peak #**	**t_R / min**	**area / %**
1	9.730	3.255
2	12.733	96.745

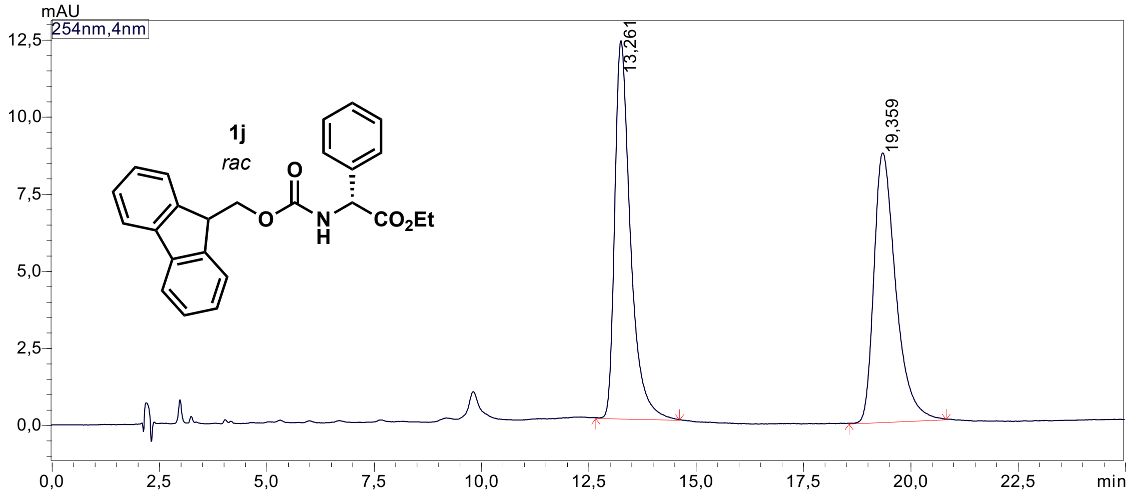

**1j-rac**: IB-3, *n*-heptane/*i*-PrOH 95:5, 298 K, 254 nm		
**peak #**	**t_R / min**	**area / %**
1	13.261	50.243
2	19.359	49.757

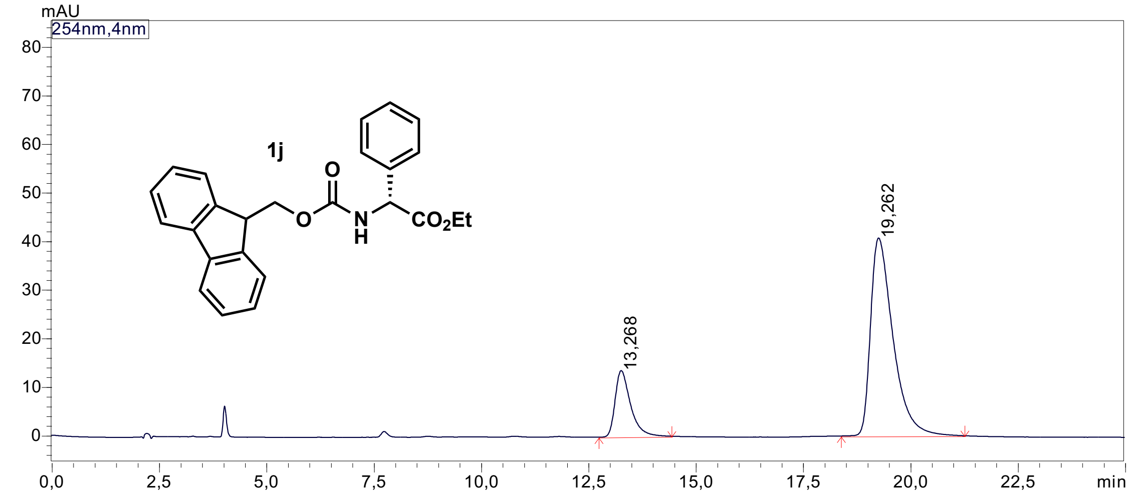

**1j**: IB-3, *n*-heptane/*i*-PrOH 95:5, 298 K, 254 nm		
**peak #**	**t_R / min**	**area / %**
1	13.268	18.751
2	19.262	81.249

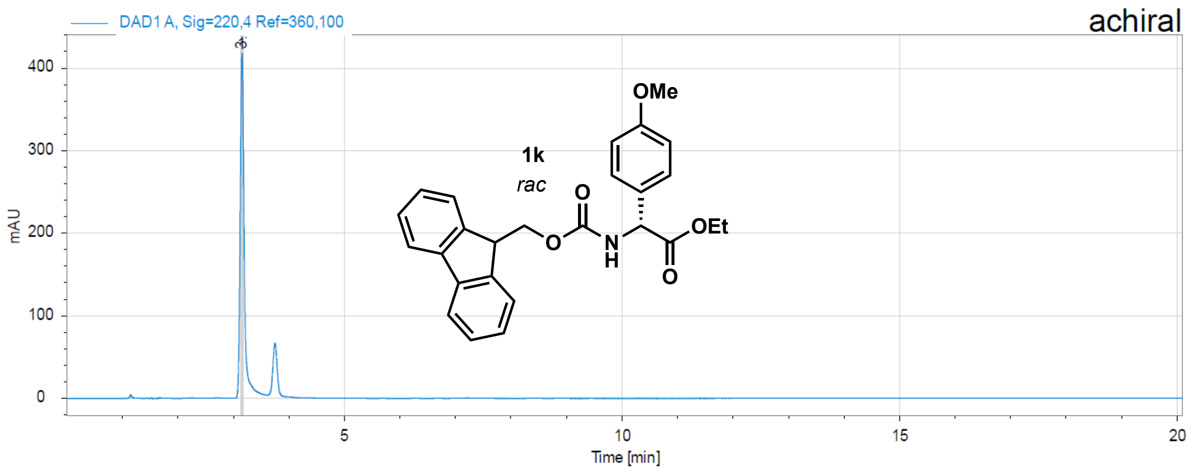

**1k-rac, 1D**: 100 mm Zorbax RX-SIL, *n*-heptane/*i*-PrOH 99:1, 308 K, 220 nm		
**peak #**	***t*$_R$ / min**	**area / %**
1	3.13 (cut start)	-

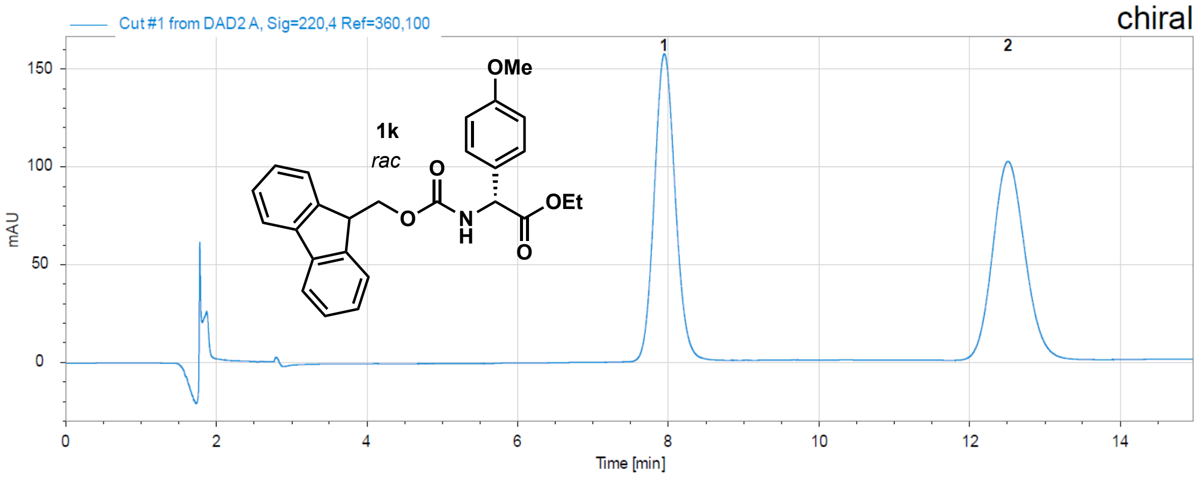

**1k-rac, 2D**: IC-3, *n*-heptane/*i*-PrOH 70:30, 298 K, 220 nm		
**peak #**	***t*$_R$ / min**	**area / %**
1	7.947	50.107
2	12.510	49.893

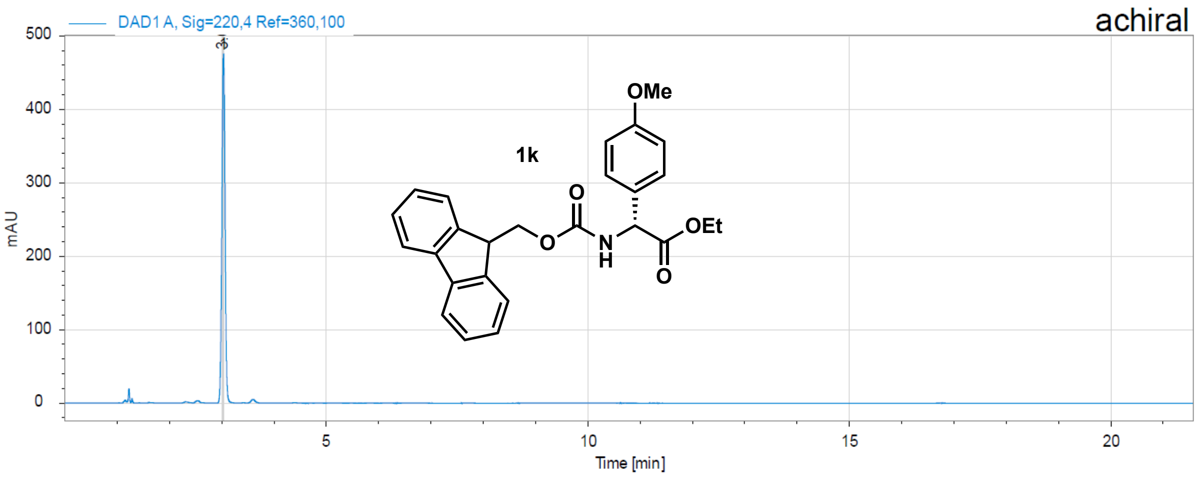

**1k, 1D**: 100 mm Zorbax RX-SIL, *n*-heptane/*i*-PrOH 99:1, 308 K, 220 nm		
**peak #**	***t*$_R$ / min**	**area / %**
1	3.034 (cut start)	98.622
2	3.598	1.378

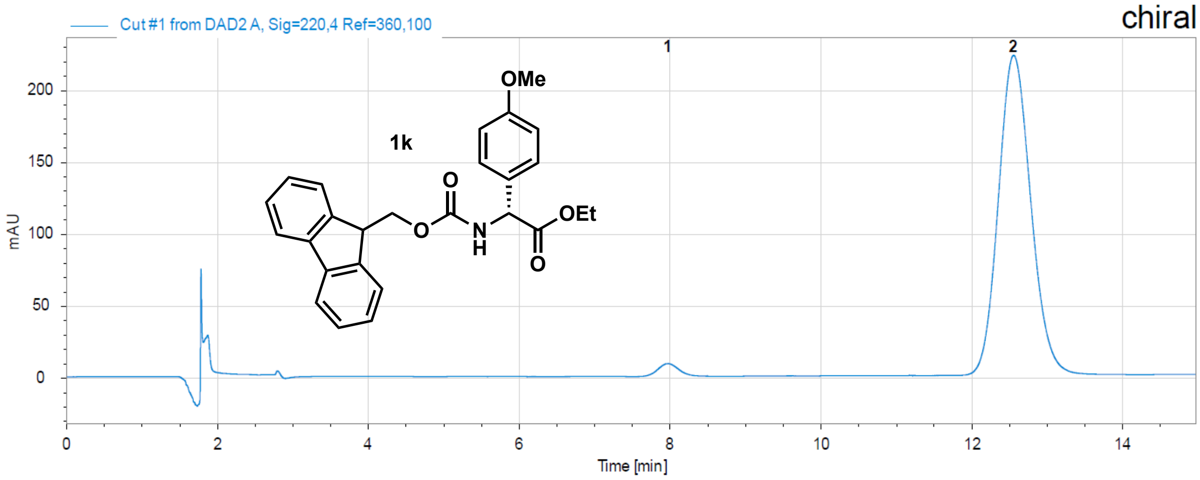

**1k, 2D**: IC-3, *n*-heptane/*i*-PrOH 70:30, 298 K, 220 nm		
**peak #**	***t*$_R$ / min**	**area / %**
1	7.971	2.567
2	12.554	97.432

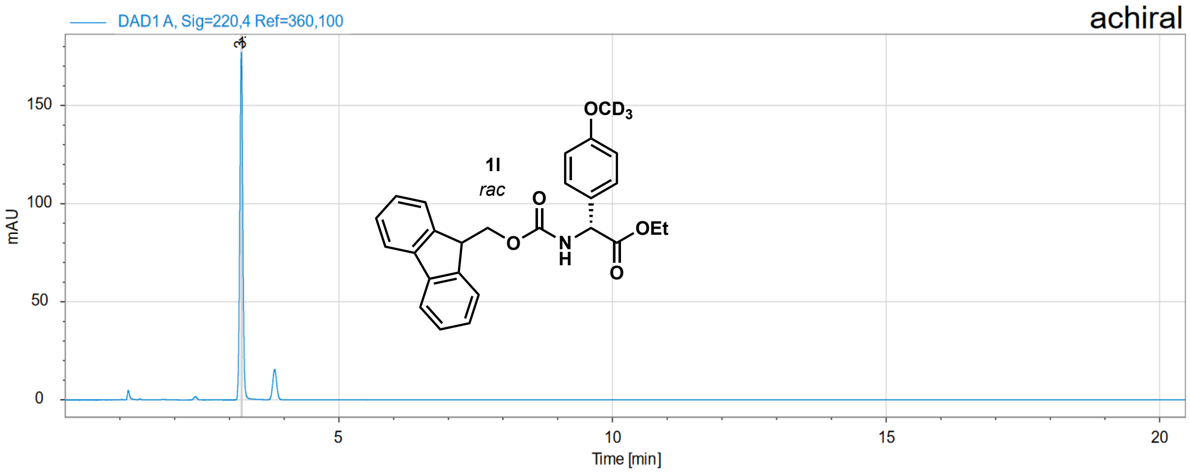

**1l-rac, 1D**: 100 mm Zorbax RX-SIL, *n*-heptane/*i*-PrOH 99:1, 308 K, 220 nm		
**peak #**	***t*$_R$ / min**	**area / %**
1	3.20 (cut start)	-

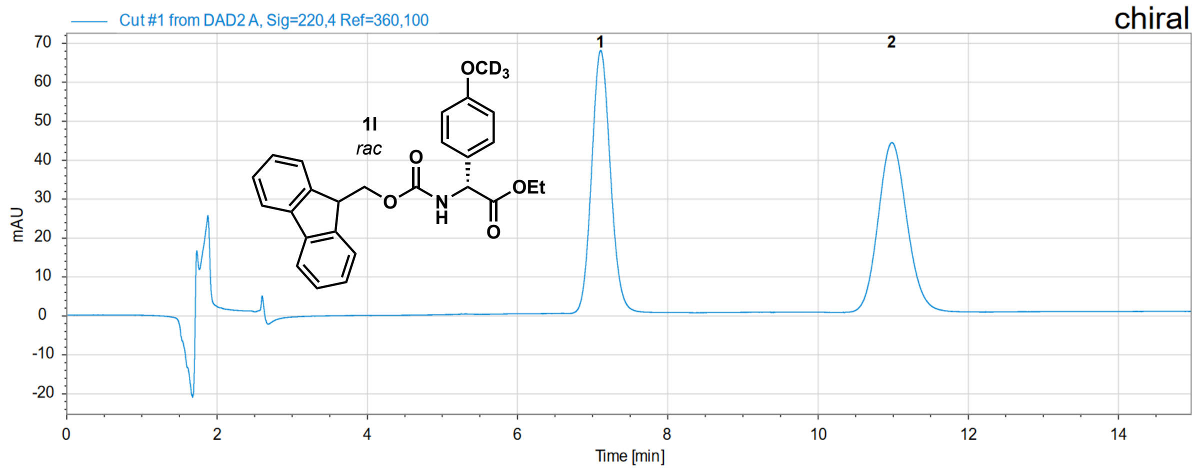

**1l-rac, 2D**: IC-3, *n*-heptane/*i*-PrOH 65:35, 298 K, 220 nm		
**peak #**	***t*$_R$ / min**	**area / %**
1	7.107	50.215
2	10.986	49.785

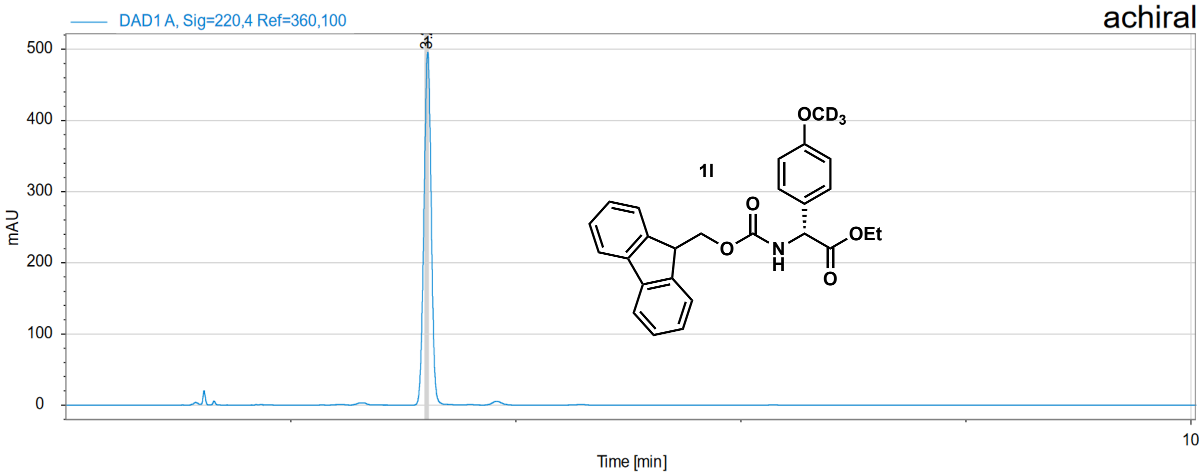

**1l, 1D**: 100 mm Zorbax RX-SIL, *n*-heptane/*i*-PrOH 99:1, 308 K, 220 nm		
**peak #**	**t_R / min**	**area / %**
1	3.125 (cut start)	98.551
2	3.829	1.449

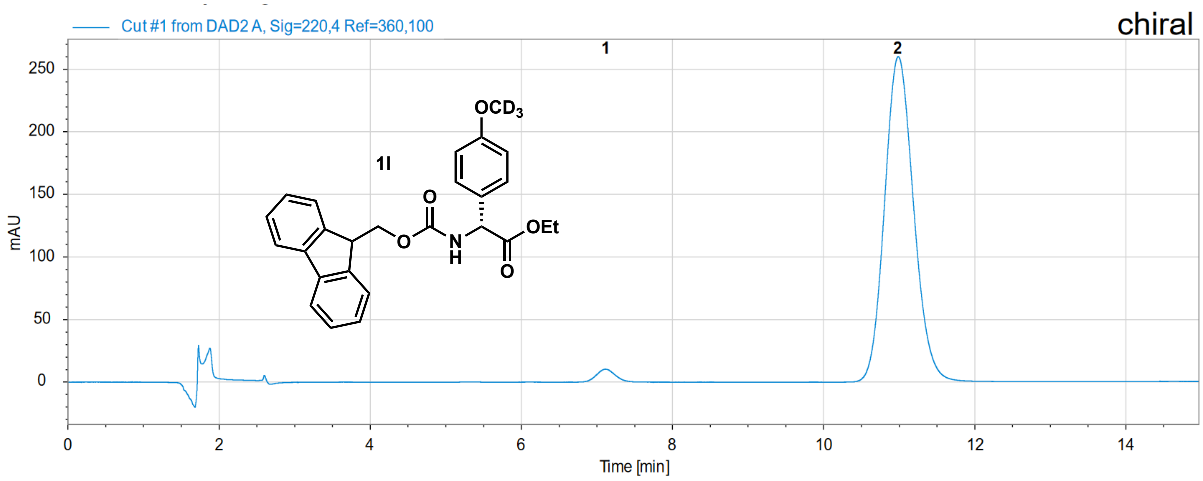

**1l, 2D**: IC-3, *n*-heptane/*i*-PrOH 65:35, 298 K, 220 nm		
**peak #**	**t_R / min**	**area / %**
1	7.113	2.508
2	10.987	97.492

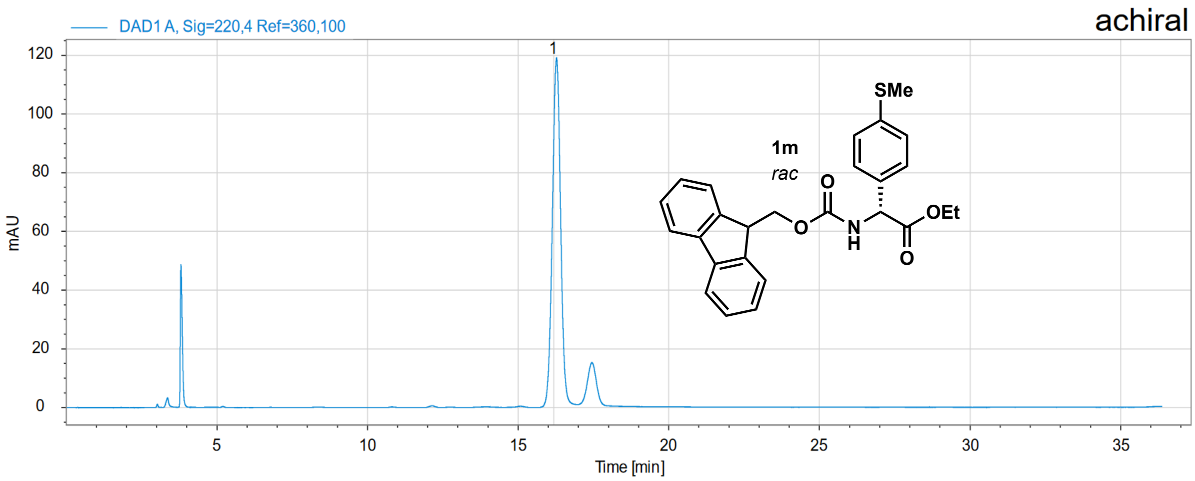

**1m-rac, 1D**: 250 mm PVA-SIL, *n*-heptane/*i*-PrOH 99:1, 308 K, 220 nm		
**peak #**	t_R **/ min**	**area / %**
1	16.16 (cut start)	-

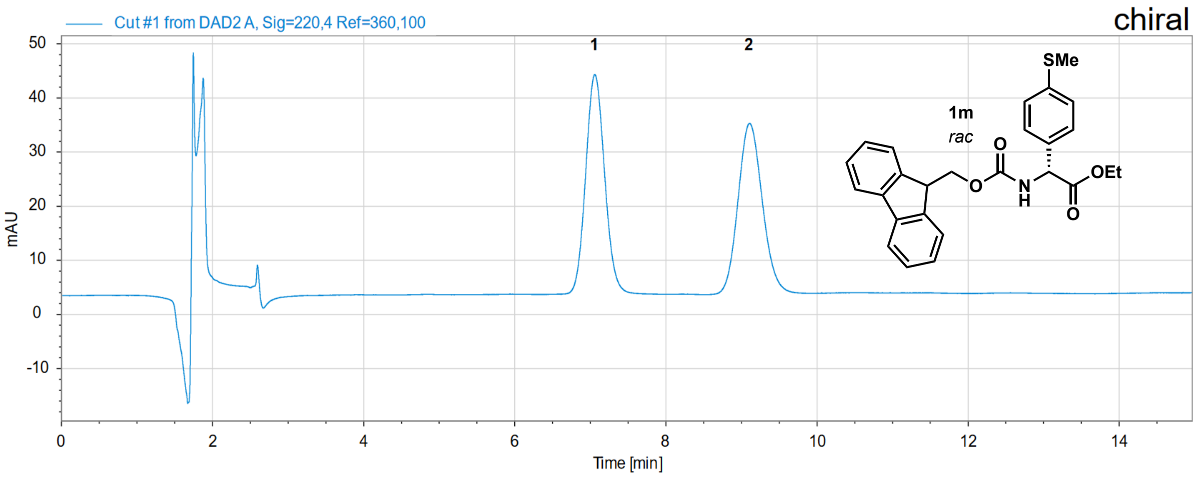

**1m-rac, 2D**: IC-3, *n*-heptane/*i*-PrOH 65:35, 298 K, 220 nm		
**peak #**	t_R **/ min**	**area / %**
1	7.060	50.071
2	9.107	49.929

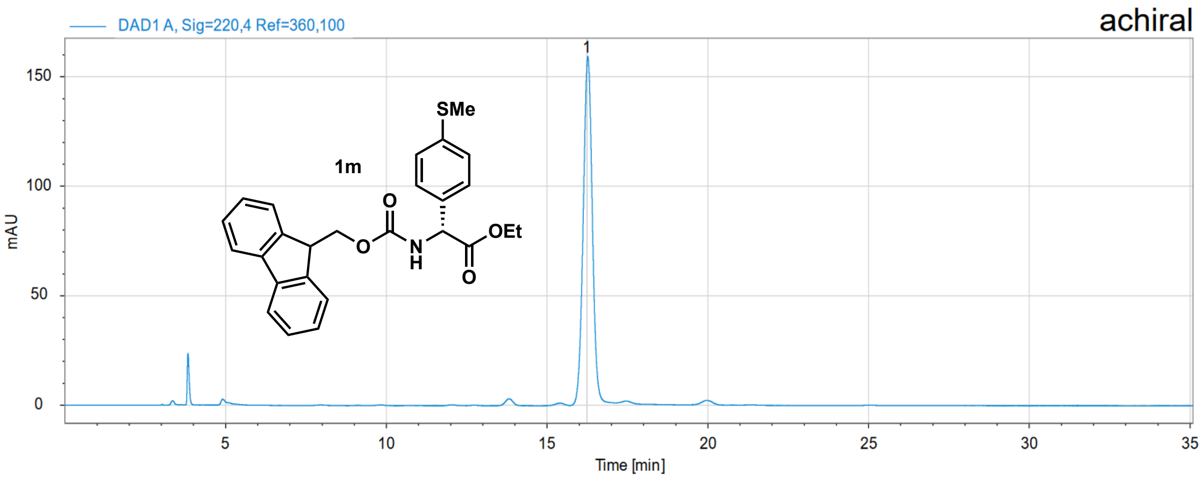

**1m, 1D**: 250 mm PVA-SIL, *n*-heptane/*i*-PrOH 99:1, 308 K, 220 nm		
**peak #**	***t*$_R$ / min**	**area / %**
1	16.267 (cut start)	97.691
2	17.458	2.309

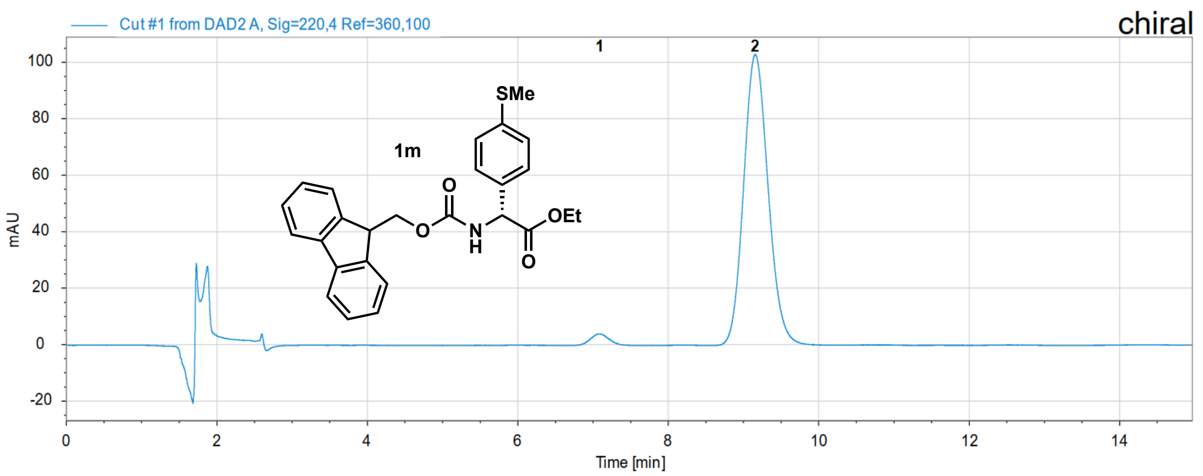

**1m, 2D**: IC-3, *n*-heptane/*i*-PrOH 65:35, 298 K, 220 nm		
**peak #**	***t*$_R$ / min**	**area / %**
1	7.090	2.953
2	9.156	97.047

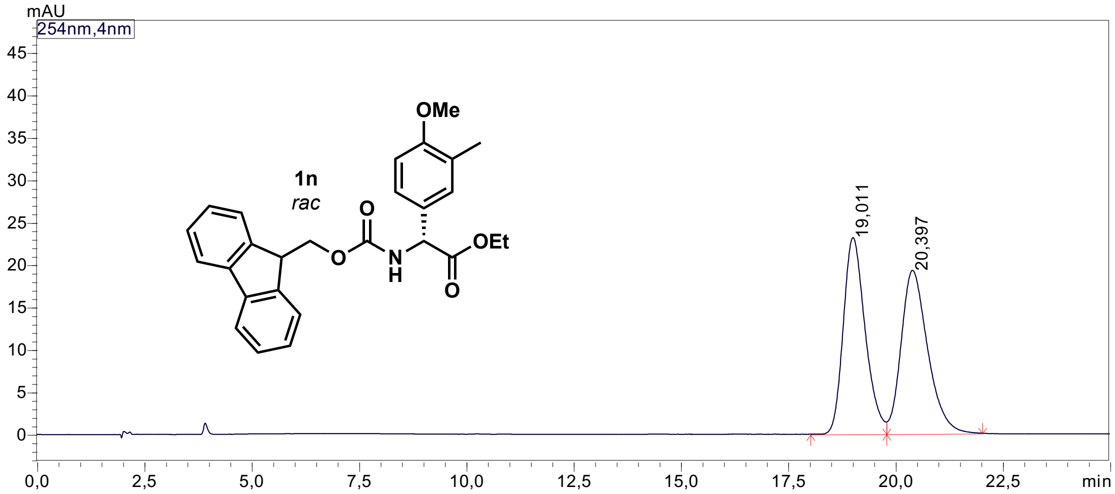

**1n-rac**: AD-3, *n*-heptane/*i*-PrOH 94:6, 298 K, 254 nm		
**peak #**	t_R **/ min**	**area / %**
1	19.011	49.747
2	20.397	50.253

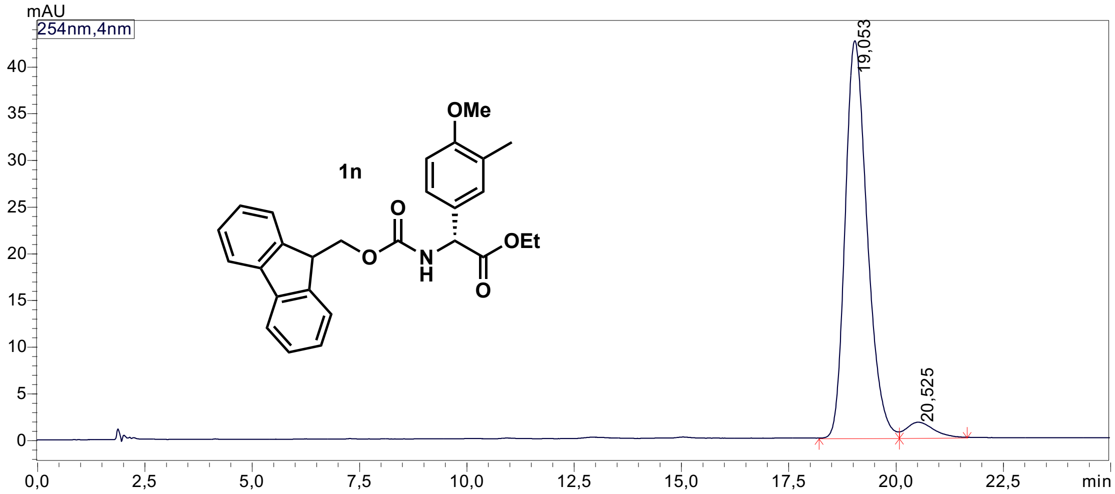

**1n**: AD-3, *n*-heptane/*i*-PrOH 94:6, 298 K, 254 nm		
**peak #**	t_R **/ min**	**area / %**
1	19.053	95.488
2	20.525	4.512

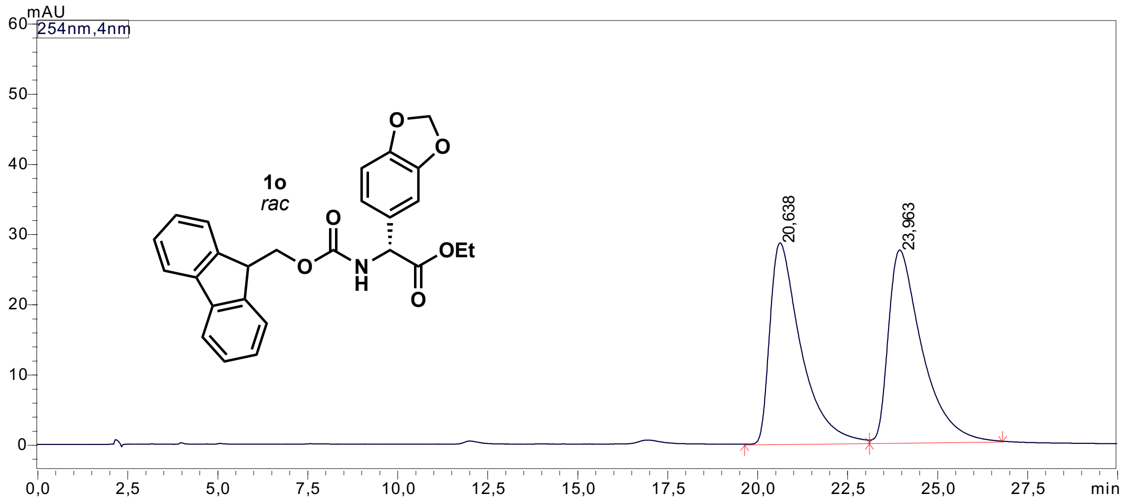

**1o-rac**: IB-3, *n*-heptane/*i*-PrOH 95:5, 298 K, 254 nm		
**peak #**	t_R **/ min**	**area / %**
1	20.638	49.099
2	23.963	50.901

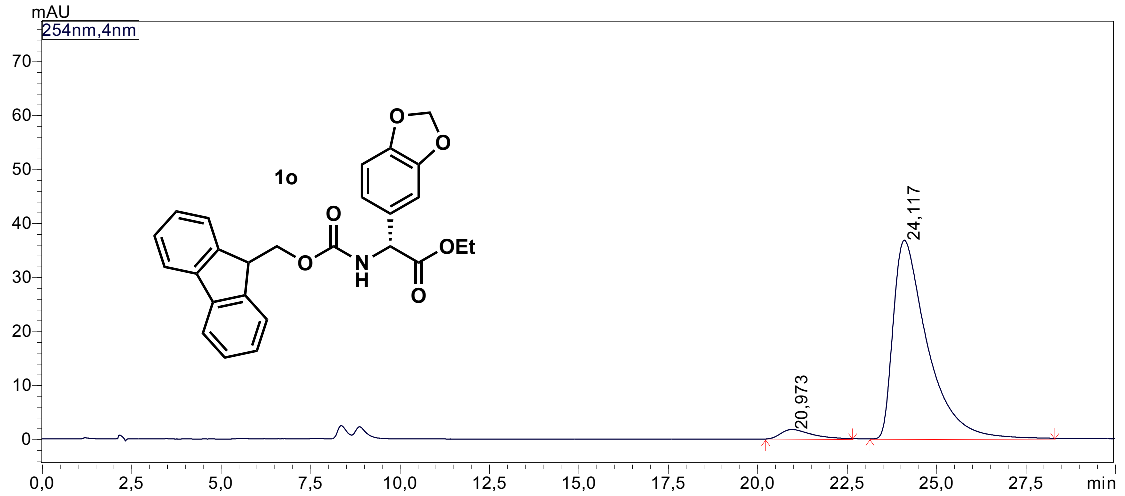

**1o**: IB-3, *n*-heptane/*i*-PrOH 95:5, 298 K, 254 nm		
**peak #**	t_R **/ min**	**area / %**
1	20.973	3.995
2	24.117	96.005

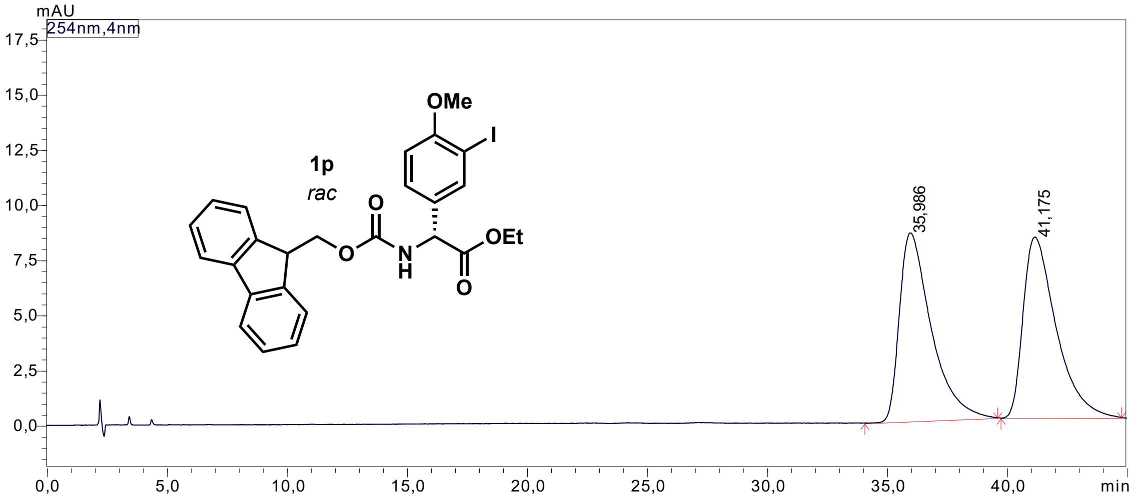

**1p-rac**: IB-3, *n*-heptane/*i*-PrOH 97:3, 298 K, 254 nm		
**peak #**	***t*$_R$ / min**	**area / %**
1	35.986	50.066
2	41.175	49.934

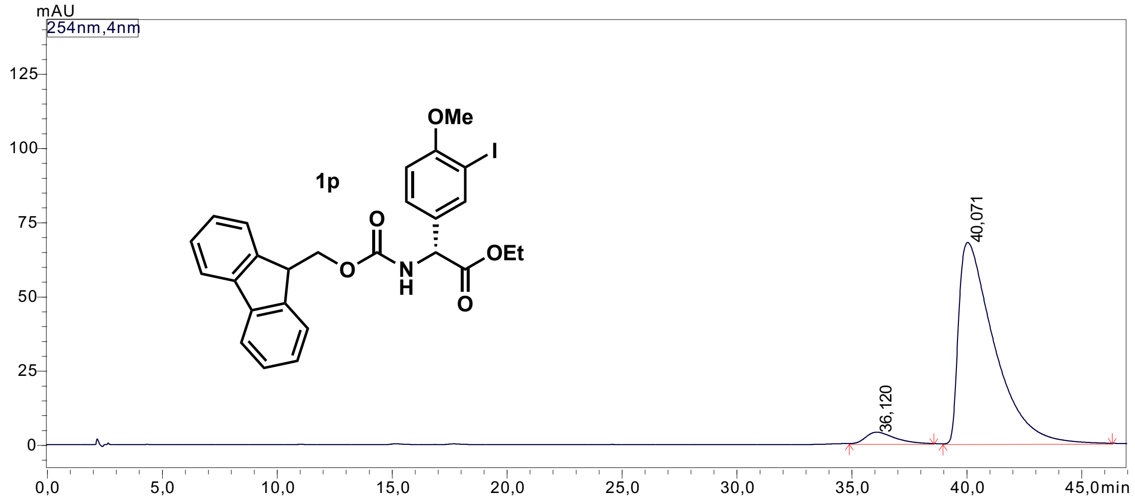

**1p**: IB-3, *n*-heptane/*i*-PrOH 97:3, 298 K, 254 nm		
**peak #**	***t*$_R$ / min**	**area / %**
1	36.120	4.324
2	40.071	95.676

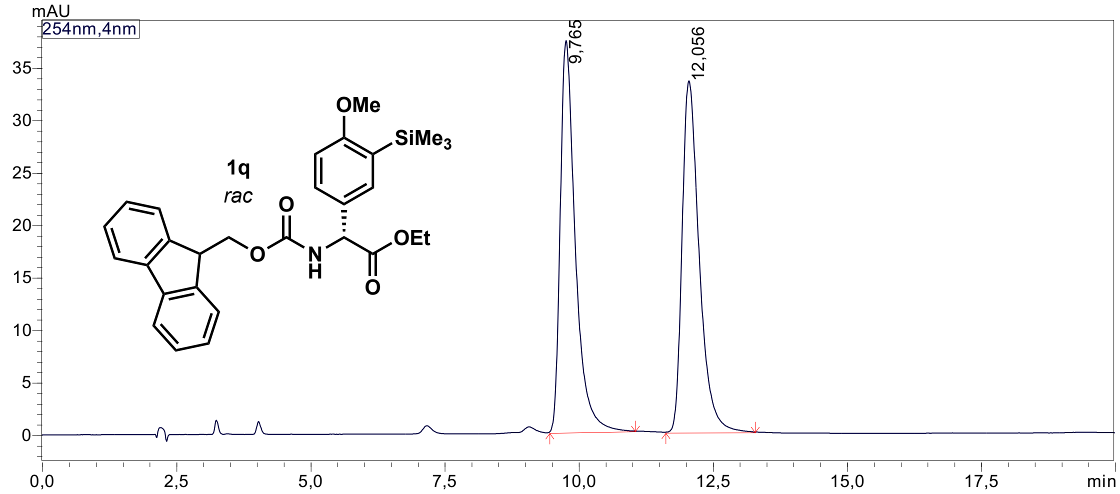

**1q-rac**: IB-3, *n*-heptane/*i*-PrOH 95:5, 298 K, 254 nm		
**peak #**	***t*R / min**	**area / %**
1	9.765	49.625
2	12.056	50.375

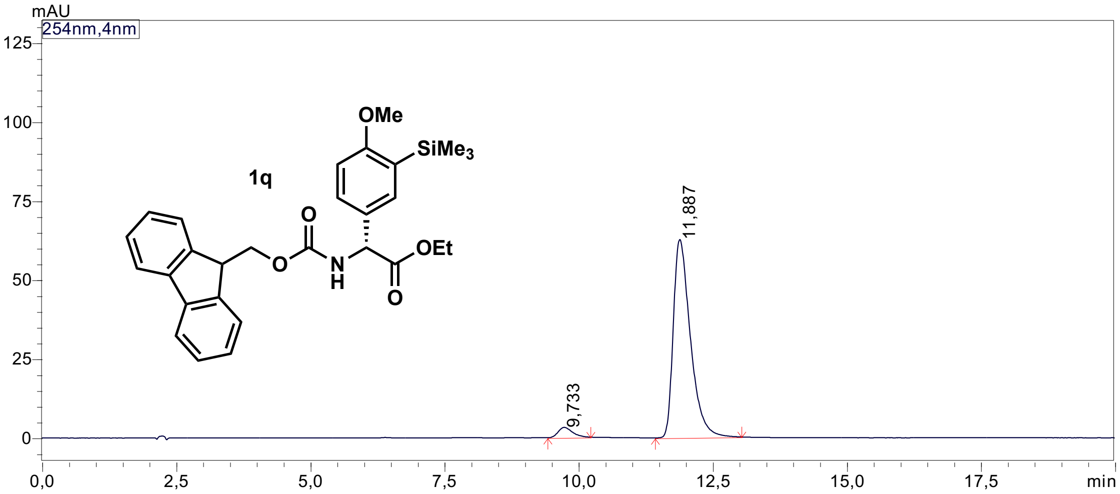

**1q**: IB-3, *n*-heptane/*i*-PrOH 95:5, 298 K, 254 nm		
**peak #**	***t*R / min**	**area / %**
1	9.733	4.081
2	11.887	95.919

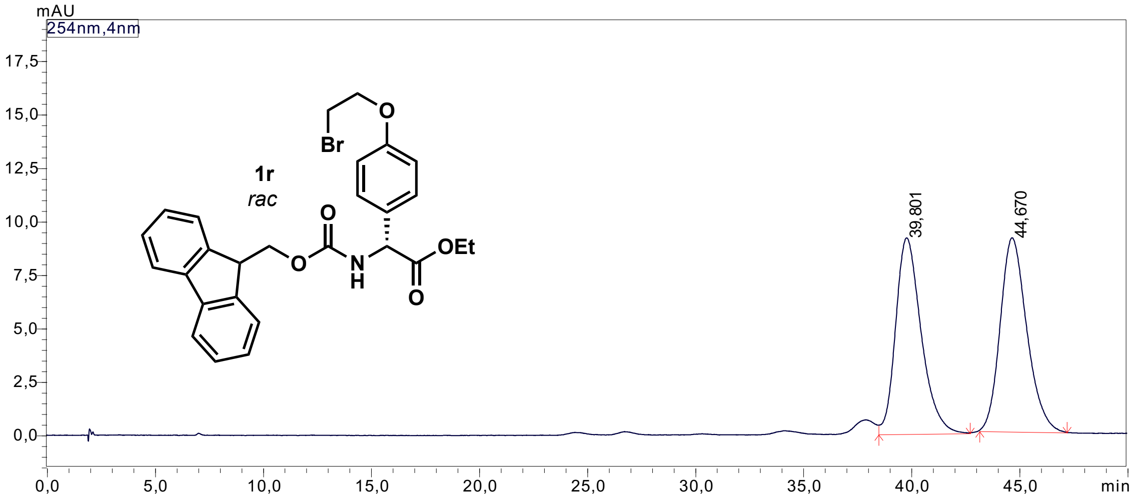

**1r-rac**: AD-3, *n*-heptane/*i*-PrOH 92:8, 298 K, 254 nm		
**peak #**	$\boldsymbol{t_R}$ **/ min**	**area / %**
1	39.801	49.174
2	44.670	50.826

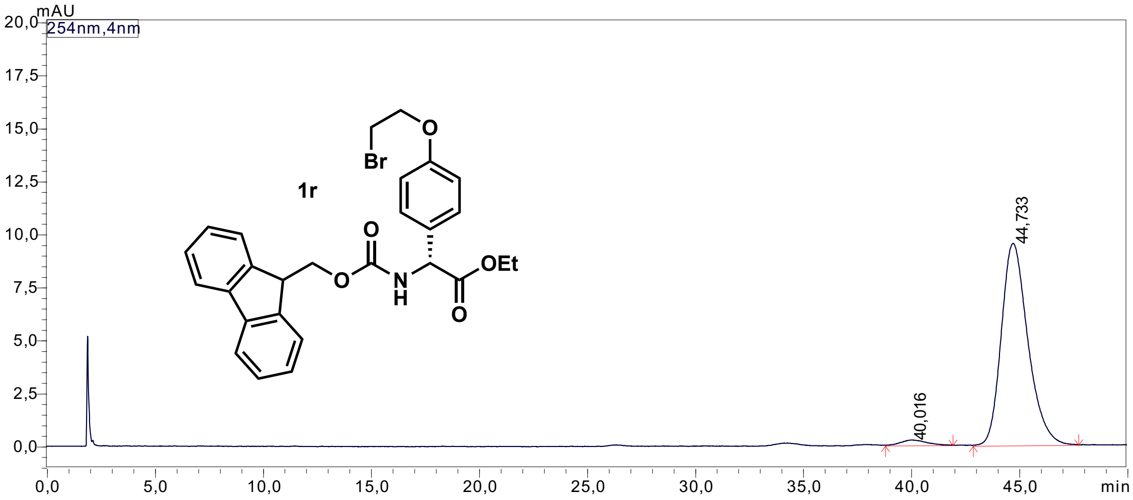

**1r**: AD-3, *n*-heptane/*i*-PrOH 92:8, 298 K, 254 nm		
**peak #**	$\boldsymbol{t_R}$ **/ min**	**area / %**
1	40.016	2.235
2	44.733	97.765

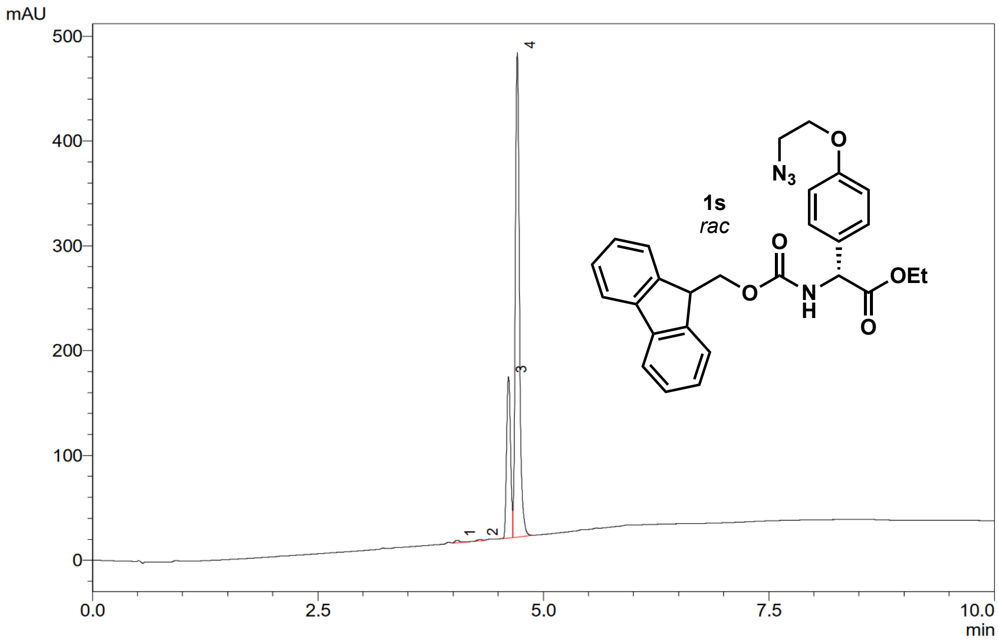

**1s-rac**: Eclipse Plus C18, MeOH/H_2O-gradient: 60:40 –5' – 90:10, 308 K, 22 nm (achiral)		
**peak #**	**t_R / min**	**area / %**
3 (minor regioisomer)	4.61	24.473
4 (major regioisomer)	4.71	75.527

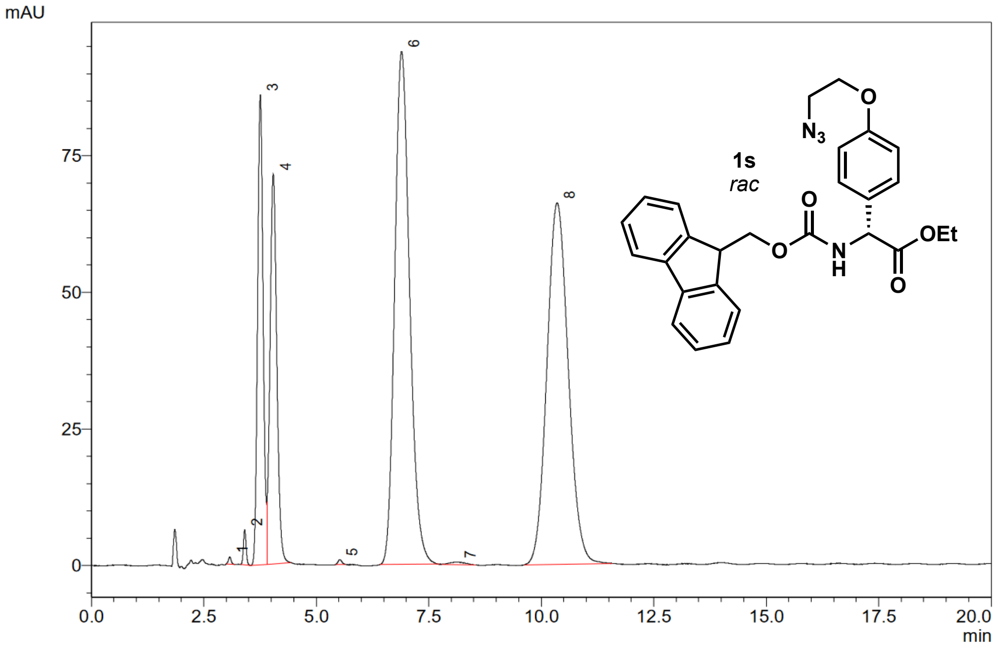

**1s-rac**: OJ-3, MeOH 100%, 298 K, 220 nm (chiral)		
**peak #**	**t_R / min**	**area / %**
3 (minor regioisomer)	3.76	12.596
4 (minor regioisomer)	4.04	12.818
6 (major regioisomer)	6.90	37.203
8 (major regioisomer)	10.35	37.384

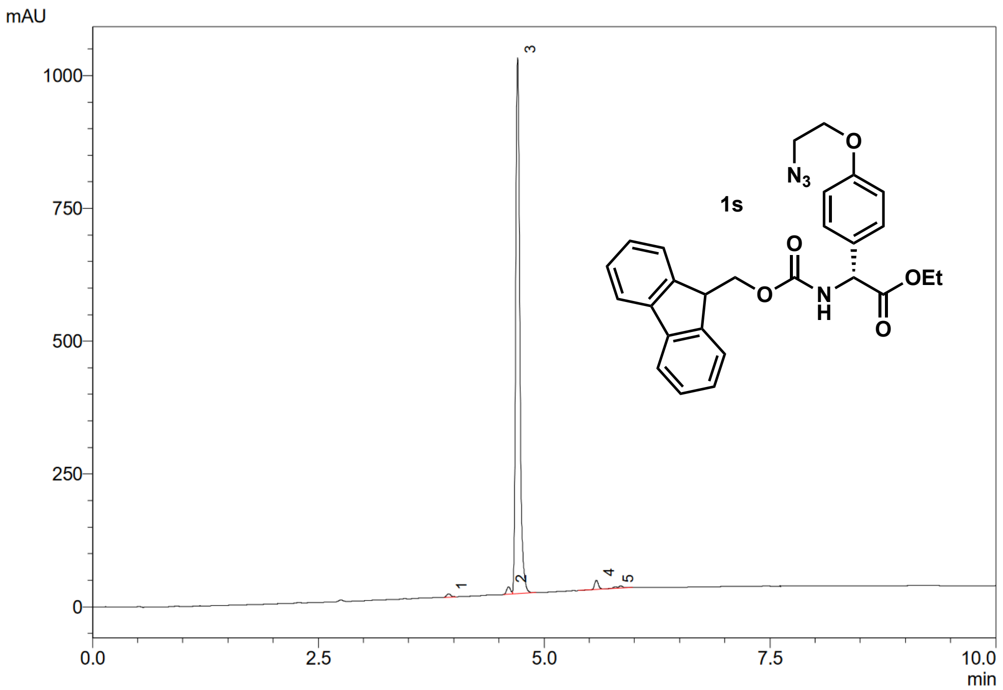

**1s**: Eclipse Plus C18, MeOH/H_2O-gradient: 60:40 –5’ – 90:10, 308 K, 22 nm (achiral)		
**peak #**	***t*$_R$ / min**	**area / %**
2 (minor regioisomer)	4.61	1.332
3 (major regioisomer)	4.71	98.668

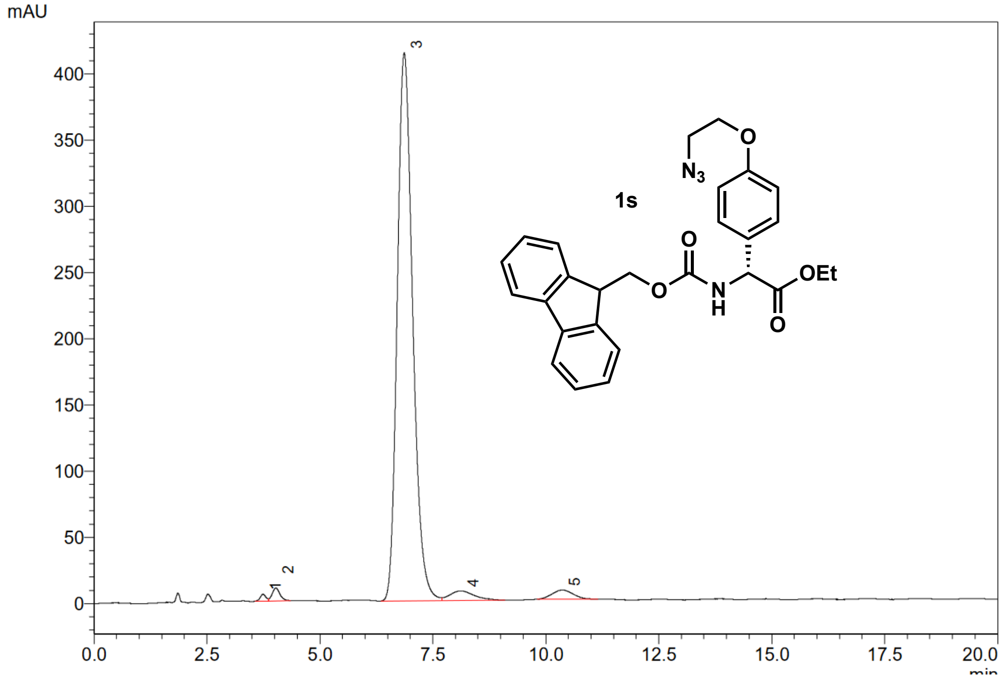

**1s**: OJ-3, MeOH 100%, 298 K, 220 nm (chiral)		
**peak #**	***t*$_R$ / min**	**area / %**
3	6.87	97.777
4	10.37	2.223

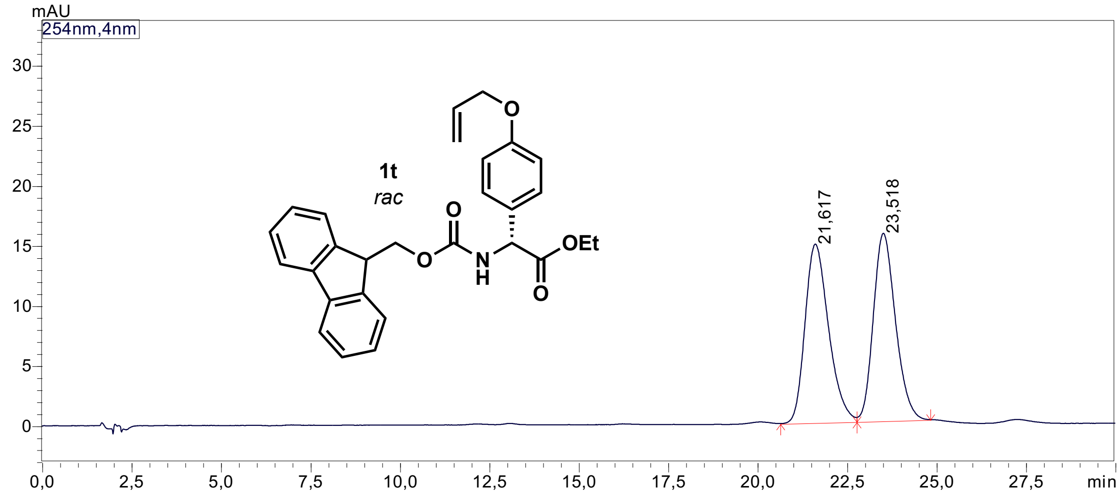

**1t-rac**: AD-3, *n*-heptane/*i*-PrOH 92:8, 298 K, 254 nm		
**peak #**	$\boldsymbol{t_R}$ **/ min**	**area / %**
1	21.617	50.318
2	23.518	49.682

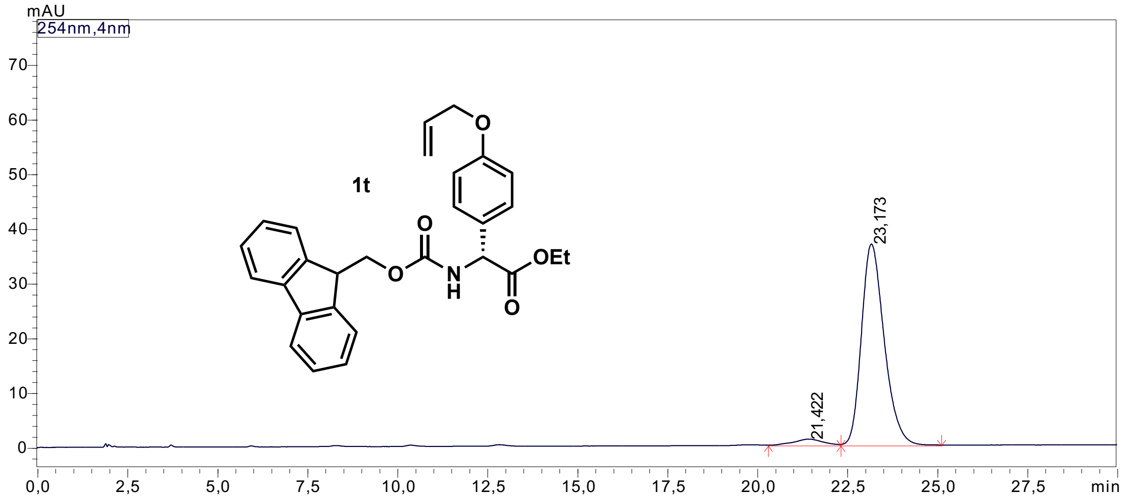

**1t**: AD-3, *n*-heptane/*i*-PrOH 92:8, 298 K, 254 nm		
**peak #**	$\boldsymbol{t_R}$ **/ min**	**area / %**
1	21.422	3.644
2	23.173	96.356

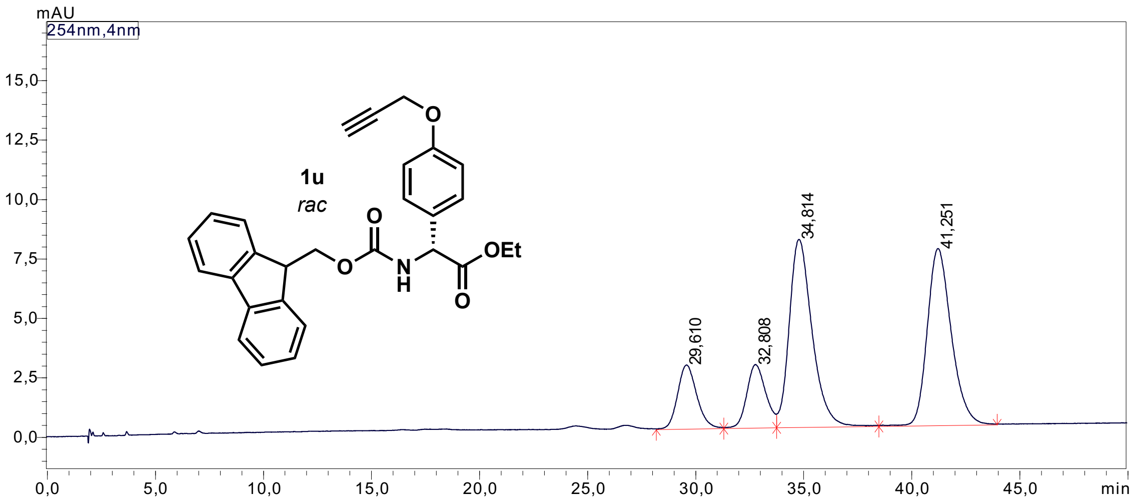

**1u-rac**: AD-3, *n*-heptane/*i*-PrOH 92:8, 298 K, 254 nm		
**peak #**	**t_R / min**	**area / %**
1 (minor regioisomer)	29.610	11.008
2 (minor regioisomer)	32.808	10.851
3 (major regioisomer)	34.814	39.388
4 (major regioisomer)	41.251	38.753

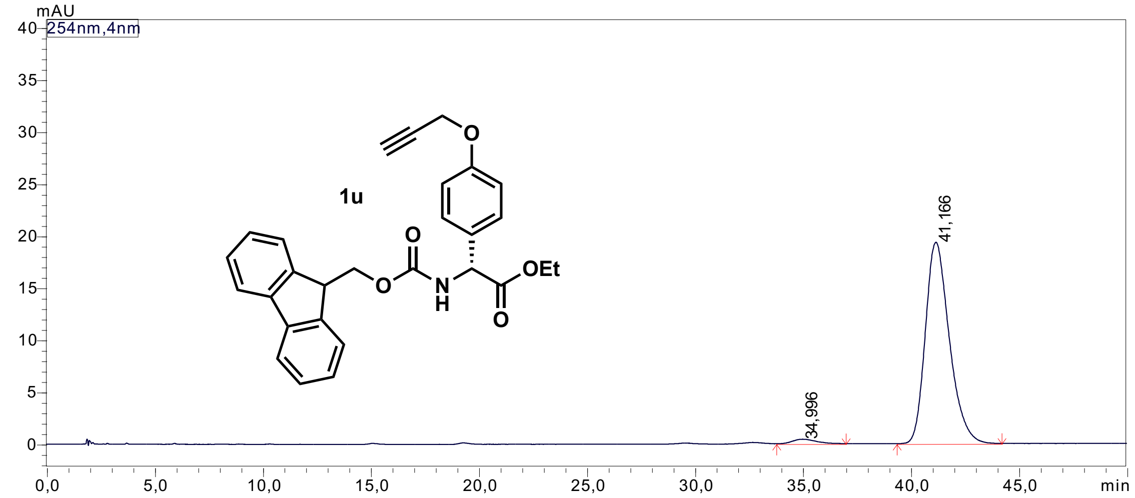

**1u**: AD-3, *n*-heptane/*i*-PrOH 92:8, 298 K, 254 nm		
**peak #**	**t_R / min**	**area / %**
1	34.996	2.113
2	41.166	97.887

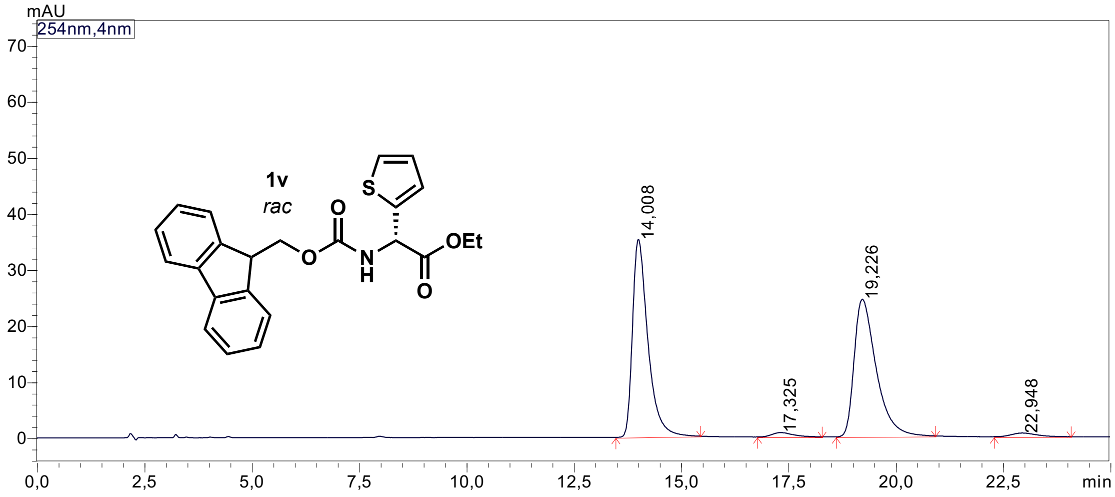

**1v-rac**: IB-3, *n*-heptane/*i*-PrOH 95:5, 298 K, 254 nm		
**peak #**	**t_R / min**	**area / %**
1 (major regioisomer)	14.008	48.710
2 (minor regioisomer)	17.325	1.460
3 (major regioisomer)	19.226	48.356
4 (minor regioisomer)	22.948	1.474

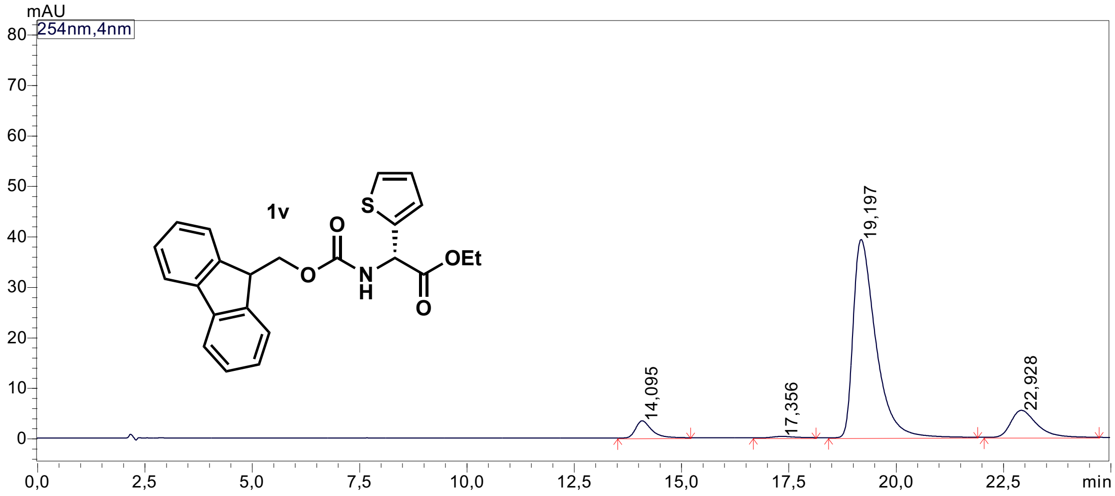

**1v**: IB-3, *n*-heptane/*i*-PrOH 95:5, 298 K, 254 nm		
**peak #**	**t_R / min**	**area / %**
1 (major regioisomer)	14.095	4.935
2 (minor regioisomer)	17.356	0.507
3 (major regioisomer)	19.197	81.744
4 (minor regioisomer)	22.928	12.814

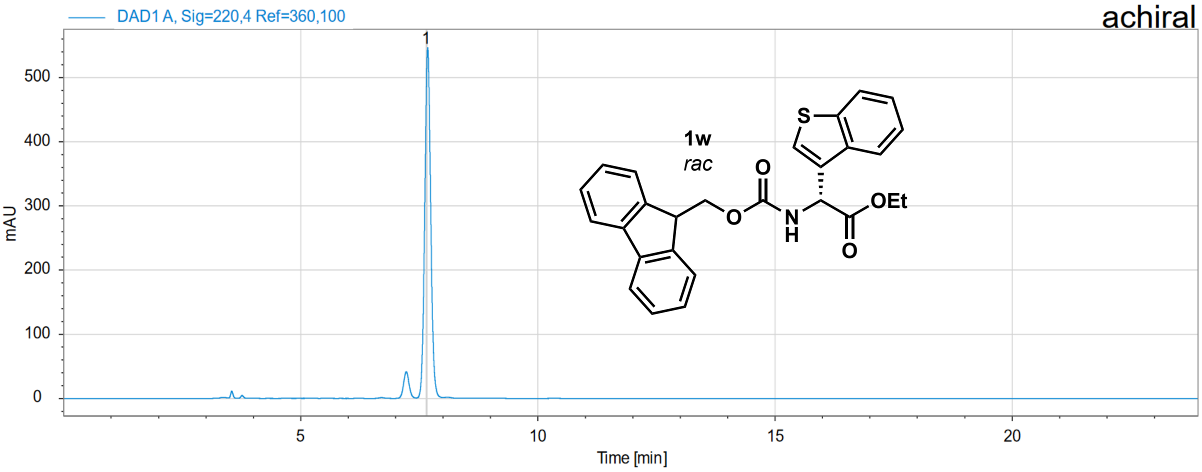

**1w-rac, 1D**: 250 mm PVA-SIL, *n*-heptane/*i*-PrOH 95:5, 308 K, 220 nm (achiral)		
**peak #**	**t_R / min**	**area / %**
2 (major regioisomer)	7.63 (start cut)	-

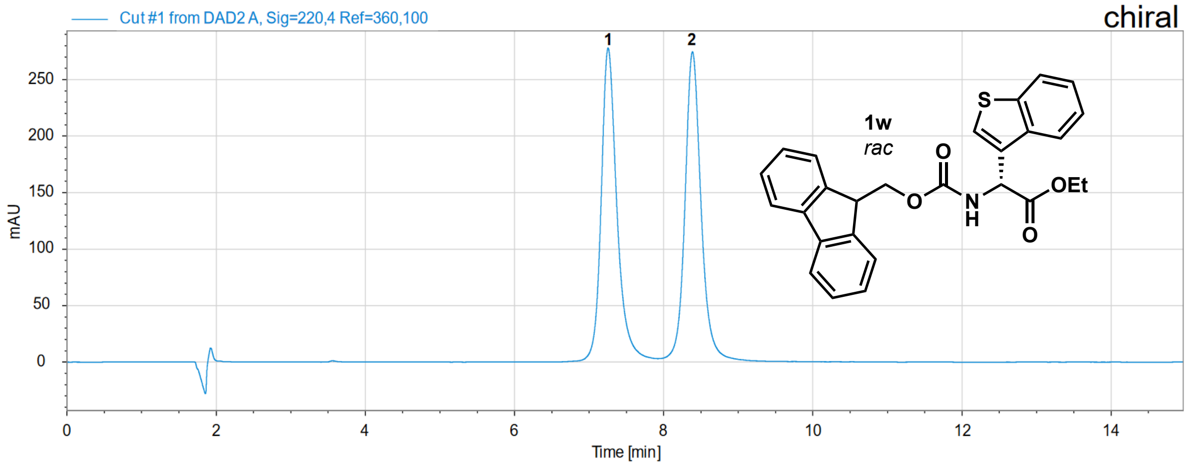

**1w-rac, 2D**: IB-3, *n*-heptane/*i*-PrOH 80:20, 298 K, 220 nm (chiral)		
**peak #**	**t_R / min**	**area / %**
1	7.255	49.814
2	8.386	50.186

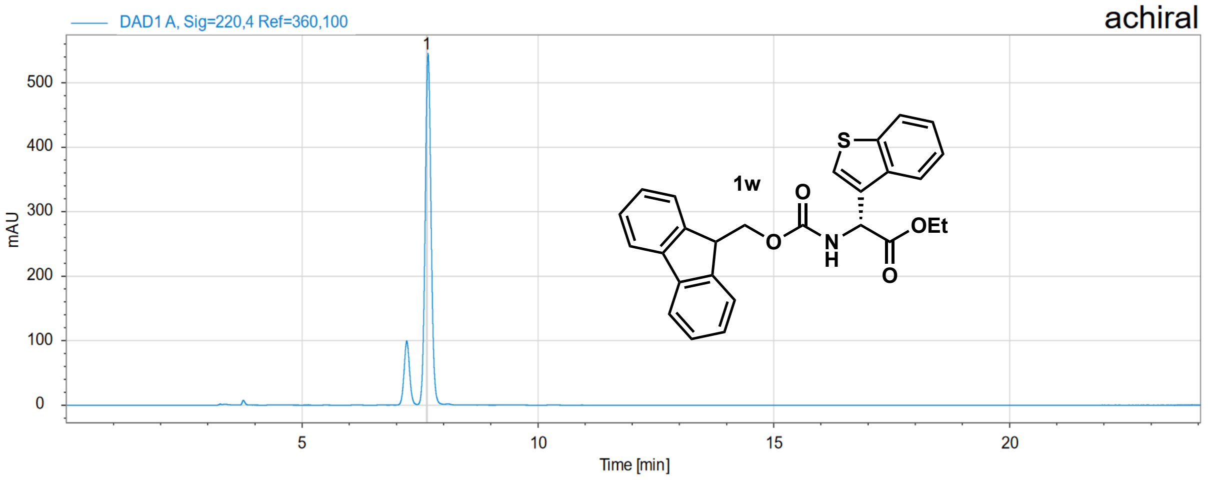

**1w, 1D**: 250 mm PVA-SIL, *n*-heptane/*i*-PrOH 95:5, 308 K, 220 nm (achiral)		
**peak #**	**t_R / min**	**area / %**
1 (minor regioisomer)	7.220	14.416
2 (major regioisomer)	7.671 (start cut)	85.583

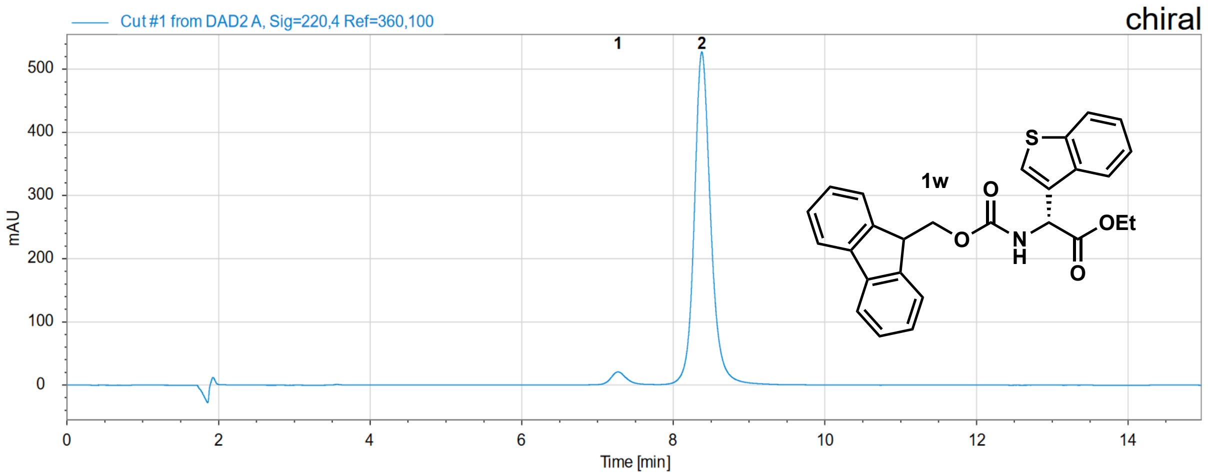

**1w, 2D**: IB-3, *n*-heptane/*i*-PrOH 80:20, 298 K, 220 nm (chiral)		
**peak #**	**t_R / min**	**area / %**
1	7.271	3.538
2	8.376	96.462

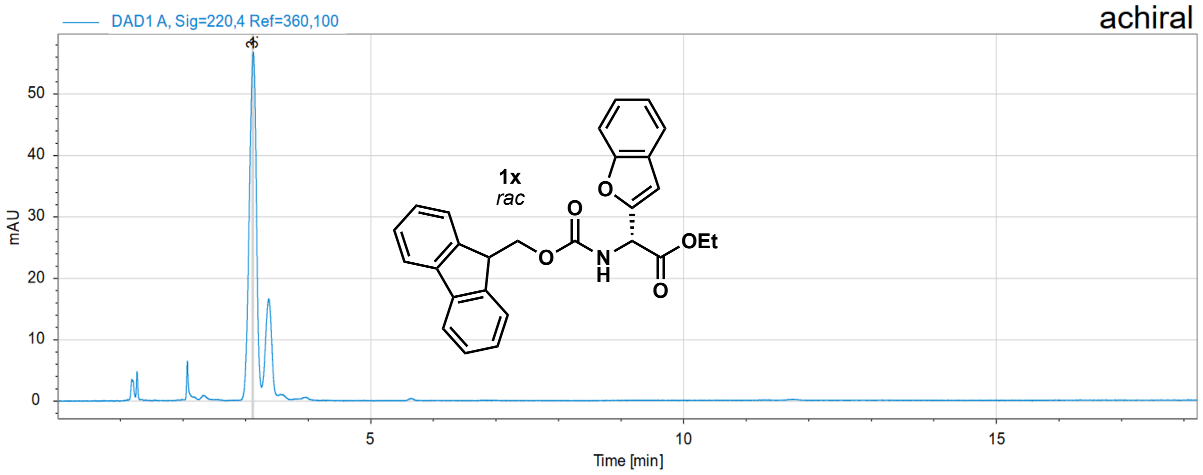

**1x-rac, 1D**: 100 mm Rx-SIL, *n*-heptane/*i*-PrOH 99.5:0.5, 308 K, 220 nm (achiral)		
**peak #**	**t_R / min**	**area / %**
1 (major regioisomer)	3.09 (start cut)	-

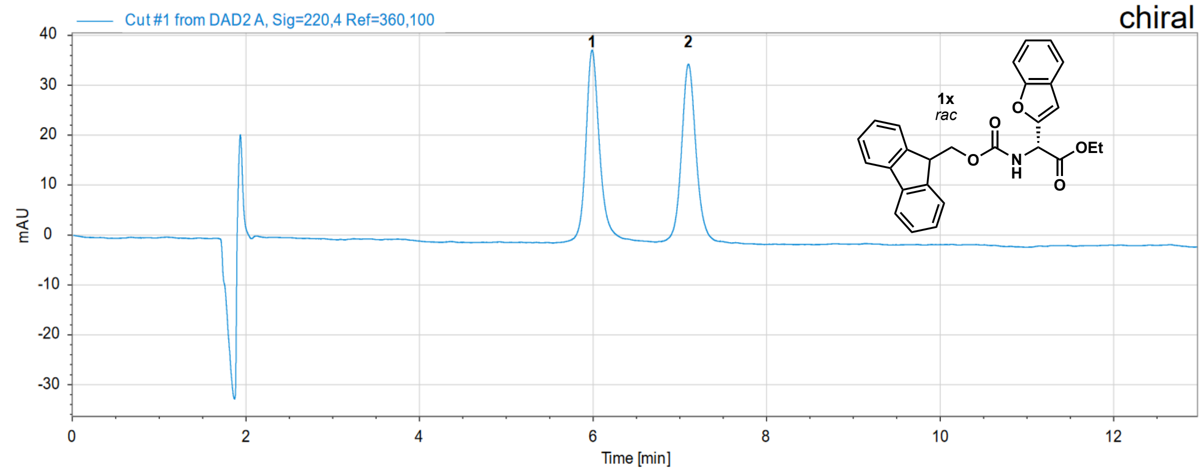

**1x-rac, 2D**: IB-3, *n*-heptane/*i*-PrOH 80:20, 298 K, 220 nm (chiral)		
**peak #**	**t_R / min**	**area / %**
1	5.989	50.996
2	7.100	49.006

achiral

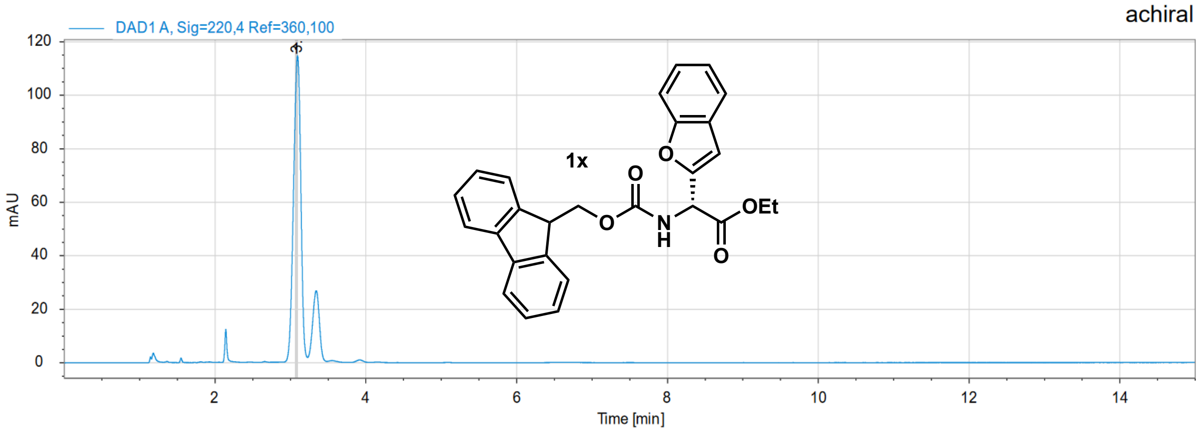

**1x, 1D**: 100 mm Rx-SIL, *n*-heptane/*i*-PrOH 99.5:0.5, 308 K, 220 nm (achiral)		
**peak #**	t_R **/ min**	**area / %**
1 (major regioisomer)	3.093 (start cut)	82.369
2 (minor regioisomer)	3.341	17.631

chiral

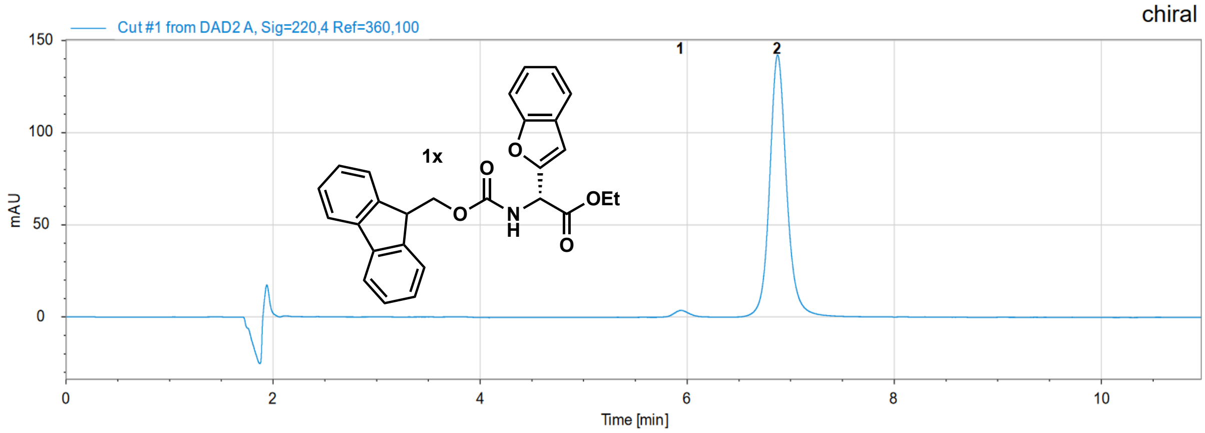

**1x, 2D**: IB-3, *n*-heptane/*i*-PrOH 80:20, 298 K, 220 nm (chiral)		
**peak #**	t_R **/ min**	**area / %**
1	5.940	2.318
2	6.874	97.681

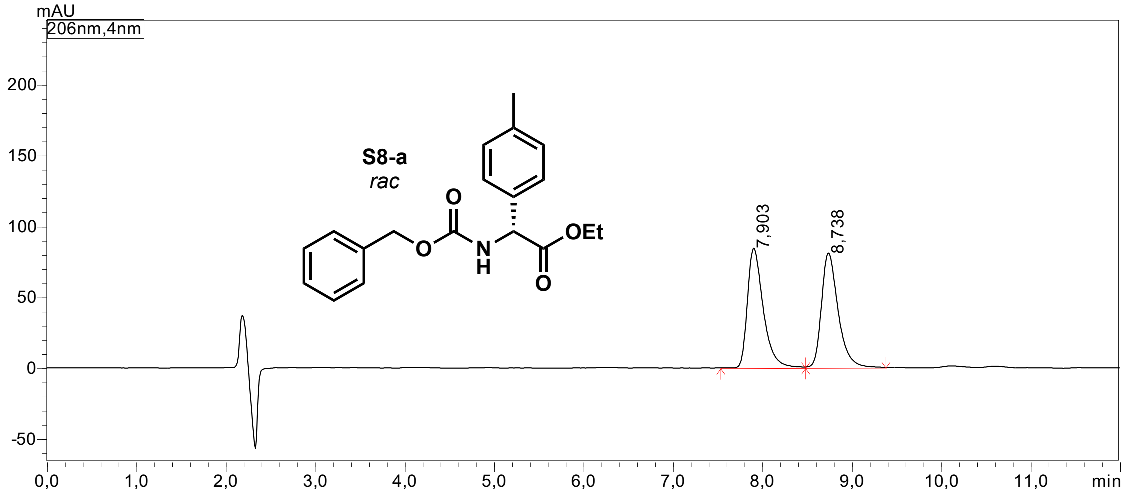

**S8-a-rac**: IB-3, *n*-heptane/*i*-PrOH 96:4, 298 K, 206 nm		
**peak #**	t_R **/ min**	**area / %**
1	7.903	49.869
2	8.738	50.131

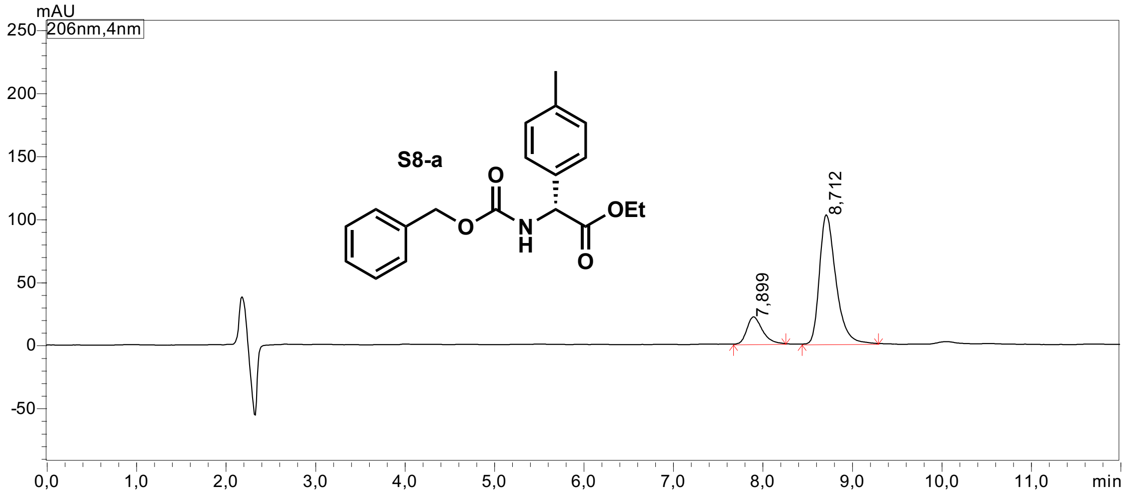

**S8-a**: IB-3, *n*-heptane/*i*-PrOH 96:4, 298 K, 206 nm		
**peak #**	t_R **/ min**	**area / %**
1	7.899	16.345
2	8.712	83.655

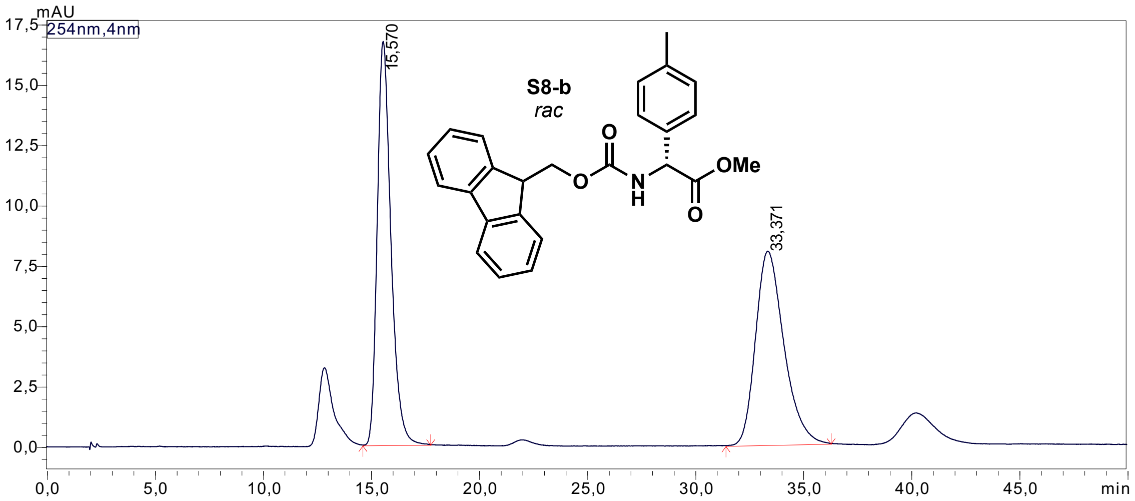

**S8-b-rac**: IC-3, *n*-heptane/*i*-PrOH 90:10, 298 K, 254 nm		
**peak #**	**t_R / min**	**area / %**
1	15.570	50.243
2	33.371	49.757

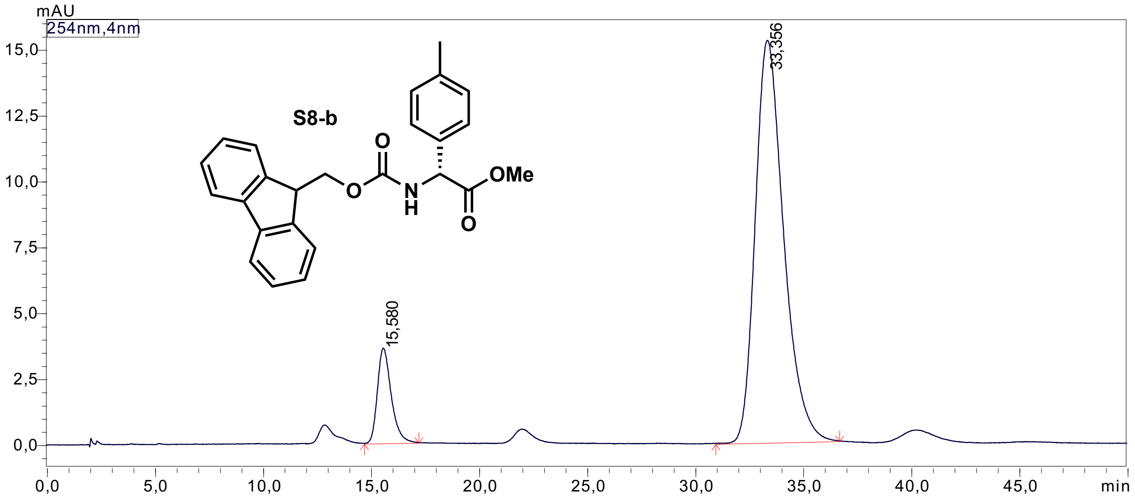

**S8-b**: IC-3, *n*-heptane/*i*-PrOH 90:10, 298 K, 254 nm		
**peak #**	**t_R / min**	**area / %**
1	15.580	10.237
2	33.356	89.763

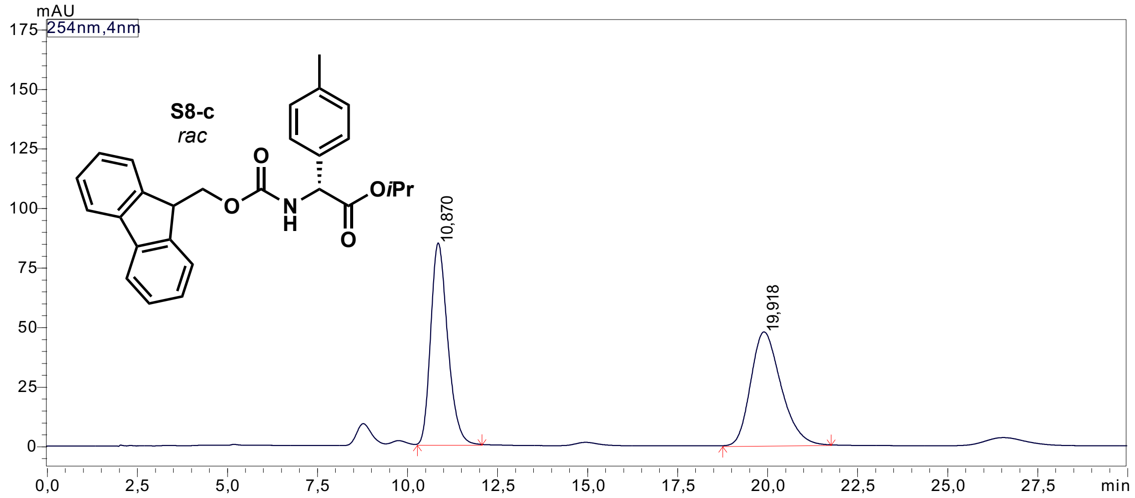

**S8-c-rac**: IC-3, *n*-heptane/*i*-PrOH 90:10, 298 K, 254 nm		
**peak #**	**t_R / min**	**area / %**
1	10.870	49.871
2	19.918	50.129

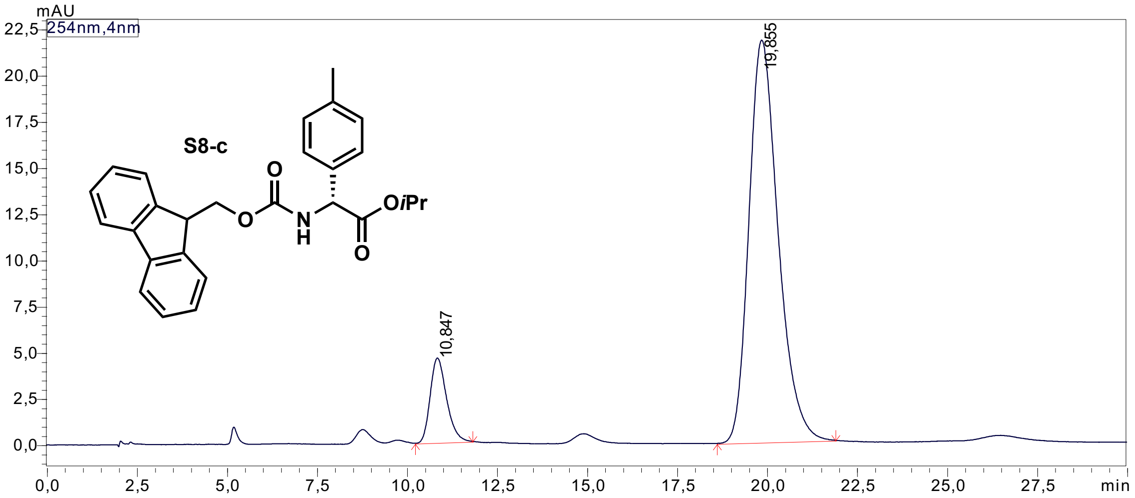

**S8-c**: IC-3, *n*-heptane/*i*-PrOH 90:10, 298 K, 254 nm		
**peak #**	**t_R / min**	**area / %**
1	10.847	10.520
2	19.855	89.480

13. Crystallographic Data

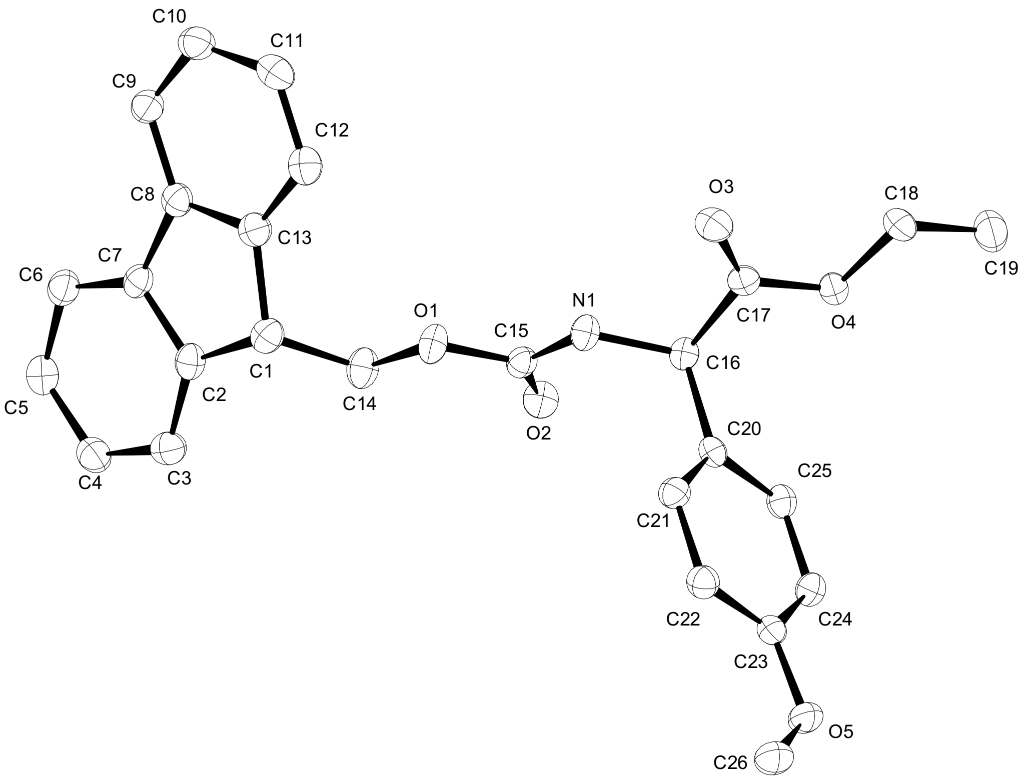

Figure SI-28: X-Ray structure of compound 1k (H atoms omitted).

Table SI-3: Crystal data and structure refinement.

Identification code	14764	
Empirical formula	$C_{26} H_{25} N O_5$	
Color	colorless	
Formula weight	431.47 g · mol^{-1}	
Temperature	100(2) K	
Wavelength	1.54178 Å	
Crystal system	ORTHORHOMBIC	
Space group	**$P2_12_12_1$, (no. 19)**	
Unit cell dimensions	a = 4.9650(2) Å	α= 90°.
	b = 15.3800(5) Å	β= 90°.
	c = 28.5256(9) Å	γ= 90°.
Volume	2178.26(13) $Å^3$	

Z	4	
Density (calculated)	1.316 Mg · m^{-3}	
Absorption coefficient	0.743 mm^{-1}	
F(000)	912 e	
Crystal size	0.107 x 0.096 x 0.031 mm^3	
θ range for data collection	3.098 to 72.230°.	
Index ranges	-6 ≤ h ≤ 5, -17 ≤ k ≤ 18, -35 ≤ 1 ≤ 35	
Reflections collected	78403	
Independent reflections	4200 [R_{int} = 0.0876]	
Reflections with I>2σ(I)	3509	
Completeness to θ = 67.679°	99.3 %	
Absorption correction	Gaussian	
Max. and min. transmission	0.98 and 0.94	
Refinement method	Full-matrix least-squares on F^2	
Data / restraints / parameters	4200 / 0 / 299	
Goodness-of-fit on F^2	1.087	
Final R indices [I>2 σ (I)]	R_1 = 0.0379	wR^2 = 0.0923
R indices (all data)	R_1 = 0.0560	wR^2 = 0.0992
Absolute structure parameter	0.23(14)	
Largest diff. peak and hole	0.4 and -0.3 e · Å^{-3}	

Table SI-4: Bond lengths [Å] and angles [°].

O(1)-C(14)	1.454(3)	O(1)-C(15)	1.347(3)
O(2)-C(15)	1.220(3)	O(3)-C(17)	1.207(3)
O(4)-C(17)	1.330(3)	O(4)-C(18)	1.454(3)
O(5)-C(23)	1.371(3)	O(5)-C(26)	1.432(3)
N(1)-C(15)	1.347(3)	N(1)-C(16)	1.452(3)
N(1)-H(1)	0.92(4)	C(1)-C(2)	1.520(4)
C(1)-C(13)	1.521(4)	C(1)-C(14)	1.521(4)
C(2)-C(3)	1.381(4)	C(2)-C(7)	1.402(4)
C(3)-C(4)	1.388(4)	C(4)-C(5)	1.392(4)
C(5)-C(6)	1.391(4)	C(6)-C(7)	1.390(4)
C(7)-C(8)	1.465(4)	C(8)-C(9)	1.391(4)
C(8)-C(13)	1.400(4)	C(9)-C(10)	1.396(4)
C(10)-C(11)	1.391(4)	C(11)-C(12)	1.392(4)
C(12)-C(13)	1.380(4)	C(16)-C(17)	1.528(4)
C(16)-C(20)	1.522(4)	C(16)-H(16)	0.97(3)
C(18)-C(19)	1.504(4)	C(20)-C(21)	1.388(4)
C(20)-C(25)	1.398(4)	C(21)-C(22)	1.394(4)
C(22)-C(23)	1.389(4)	C(23)-C(24)	1.398(4)
C(24)-C(25)	1.376(4)		

C(15)-O(1)-C(14)	113.3(2)	C(17)-O(4)-C(18)	116.3(2)
C(23)-O(5)-C(26)	117.2(2)	C(15)-N(1)-C(16)	118.6(2)
C(15)-N(1)-H(1)	120(2)	C(16)-N(1)-H(1)	119(2)
C(2)-C(1)-C(13)	102.1(2)	C(2)-C(1)-C(14)	110.3(2)
C(14)-C(1)-C(13)	115.5(2)	C(3)-C(2)-C(1)	129.1(2)
C(3)-C(2)-C(7)	120.8(2)	C(7)-C(2)-C(1)	110.1(2)
C(2)-C(3)-C(4)	118.8(3)	C(3)-C(4)-C(5)	120.8(3)
C(6)-C(5)-C(4)	120.6(3)	C(7)-C(6)-C(5)	118.7(2)
C(2)-C(7)-C(8)	108.7(2)	C(6)-C(7)-C(2)	120.3(3)
C(6)-C(7)-C(8)	131.0(2)	C(9)-C(8)-C(7)	130.4(2)
C(9)-C(8)-C(13)	120.8(3)	C(13)-C(8)-C(7)	108.8(2)
C(8)-C(9)-C(10)	118.4(3)	C(11)-C(10)-C(9)	120.8(3)
C(10)-C(11)-C(12)	120.3(3)	C(13)-C(12)-C(11)	119.4(3)
C(8)-C(13)-C(1)	110.1(2)	C(12)-C(13)-C(1)	129.5(2)
C(12)-C(13)-C(8)	120.3(3)	O(1)-C(14)-C(1)	107.9(2)
O(1)-C(15)-N(1)	111.5(2)	O(2)-C(15)-O(1)	123.2(2)
O(2)-C(15)-N(1)	125.3(2)	N(1)-C(16)-C(17)	108.8(2)

N(1)-C(16)-C(20)	113.8(2)	N(1)-C(16)-H(16)	108.8(16)
C(17)-C(16)-H(16)	106.5(16)	C(20)-C(16)-C(17)	109.4(2)
C(20)-C(16)-H(16)	109.2(16)	O(3)-C(17)-O(4)	125.2(2)
O(3)-C(17)-C(16)	124.9(2)	O(4)-C(17)-C(16)	109.9(2)
O(4)-C(18)-C(19)	106.8(2)	C(21)-C(20)-C(16)	121.8(2)
C(21)-C(20)-C(25)	118.5(2)	C(25)-C(20)-C(16)	119.8(2)
C(20)-C(21)-C(22)	121.2(2)	C(23)-C(22)-C(21)	119.5(2)
O(5)-C(23)-C(22)	124.7(2)	O(5)-C(23)-C(24)	115.5(2)
C(22)-C(23)-C(24)	119.8(2)	C(25)-C(24)-C(23)	120.1(2)
C(24)-C(25)-C(20)	121.0(2)		

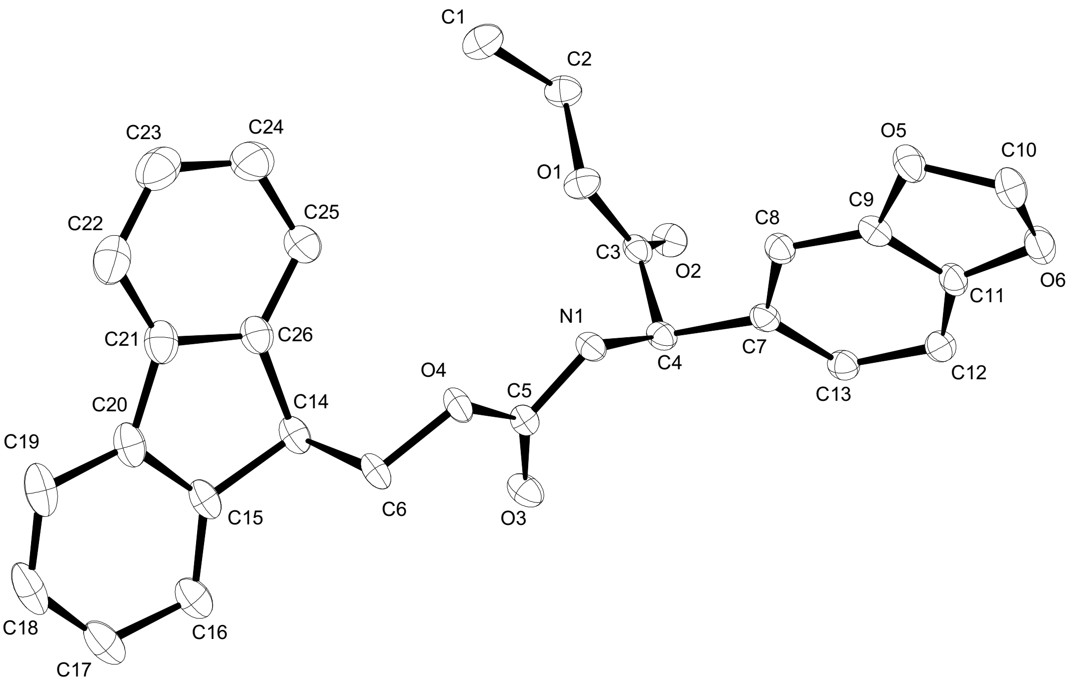

Figure SI-29: X-Ray structure of compound 1o (H atoms omitted).

Table SI-5: Crystal data and structure refinement.

Identification code	15002	
Empirical formula	$C_{26}H_{23}NO_6$	
Color	colorless	
Formula weight	445.45 g·mol^{-1}	
Temperature	100(2) K	
Wavelength	0.71073 Å	
Crystal system	MONOCLINIC	
Space group	**$P2_1$, (no. 4)**	
Unit cell dimensions	a = 11.8304(5) Å	α= 90°.
	b = 5.3051 (2) Å	β= 92.125(2)°.
	c = 16.9907(7) Å	γ = 90°.
Volume	1065.63(7) Å^3	
Z	2	

Density (calculated)	1.388 Mg · m^{-3}	
Absorption coefficient	0.099 mm^{-1}	
F(000)	468 e	
Crystal size	0.220 x 0.114 x 0.050 mm^3	
θ range for data collection	2.062 to 31.055°.	
Index ranges	-17 ≤ h ≤ 17, -7 ≤ k ≤ 7, -24 ≤ 1 ≤ 24	
Reflections collected	241683	
Independent reflections	6831 [R_{int} = 0.0441]	
Reflections with I>2 σ (I)	6614	
Completeness to θ = 25.242°	99.8 %	
Absorption correction	Semi-empirical from equivalents	
Max. and min. transmission	1.00 and 0.97	
Refinement method	Full-matrix least-squares on F^2	
Data / restraints / parameters	6831 / 1 / 311	
Goodness-of-fit on F^2	1.079	
Final R indices [I>2 σ (I)]	R_1 = 0.0305	wR^2 = 0.0829
R indices (all data)	R_1 = 0.0317	wR^2 = 0.0842
Absolute structure parameter	0.14(11)	
Largest diff. peak and hole	0.3 and -0.2 e · Å^{-3}	

Table SI-6: Bond lengths [Å] and angles [°].

O(1)-C(2)	1.4568(13)	O(1)-C(3)	1.3270(13)
O(2)-C(3)	1.2115(14)	O(3)-C(5)	1.2169(14)
O(4)-C(5)	1.3507(14)	O(4)-C(6)	1.4418(13)
O(5)-C(9)	1.3747(14)	O(5)-C(10)	1.4365(15)
O(6)-C(10)	1.4398(18)	O(6)-C(11)	1.3759(14)
N(1)-H(1)	0.842(19)	N(1)-C(4)	1.4497(14)
N(1)-C(5)	1.3570(13)	C(1)-C(2)	1.5062(18)
C(3)-C(4)	1.5334(15)	C(4)-H(4)	0.94(2)
C(4)-C(7)	1.5227(15)	C(6)-C(14)	1.5348(17)
C(7)-C(8)	1.4084(15)	C(7)-C(13)	1.3915(15)
C(8)-C(9)	1.3758(15)	C(9)-C(11)	1.3877(16)
C(11)-C(12)	1.3751(17)	C(12)-C(13)	1.4076(15)
C(14)-H(14)	1.01(3)	C(14)-C(15)	1.5162(16)
C(14)-C(26)	1.5127(18)	C(15)-C(16)	1.3871(18)
C(15)-C(20)	1.4037(18)	C(16)-C(17)	1.3991(18)
C(17)-C(18)	1.395(3)	C(18)-C(19)	1.391(3)
C(19)-C(20)	1.3946(18)	C(20)-C(21)	1.470(2)
C(21)-C(22)	1.391(2)	C(21)-C(26)	1.4045(18)
C(22)-C(23)	1.399(3)	C(23)-C(24)	1.392(3)
C(24)-C(25)	1.396(2)	C(25)-C(26)	1.3939(18)

C(3)-O(1)-C(2)	117.65(9)	C(5)-O(4)-C(6)	115.87(9)
C(9)-O(5)-C(10)	104.62(10)	C(11)-O(6)-C(10)	104.62(9)
C(4)-N(1)-H(1)	116.6(13)	C(5)-N(1)-H(1)	119.5(13)
C(5)-N(1)-C(4)	120.49(9)	O(1)-C(2)-C(1)	106.19(10)
O(1)-C(3)-C(4)	111.23(9)	O(2)-C(3)-O(1)	125.10(10)
O(2)-C(3)-C(4)	123.56(10)	N(1)-C(4)-C(3)	112.12(9)
N(1)-C(4)-H(4)	108.2(12)	N(1)-C(4)-C(7)	112.48(9)
C(3)-C(4)-H(4)	106.6(12)	C(7)-C(4)-C(3)	105.98(8)
C(7)-C(4)-H(4)	111.3(12)	O(3)-C(5)-O(4)	125.18(10)
O(3)-C(5)-N(1)	125.77(11)	O(4)-C(5)-N(1)	109.02(10)
O(4)-C(6)-C(14)	111.07(9)	C(8)-C(7)-C(4)	119.28(9)
C(13)-C(7)-C(4)	119.53(10)	C(13)-C(7)-C(8)	121.07(10)
C(9)-C(8)-C(7)	116.57(10)	O(5)-C(9)-C(8)	127.92(11)
O(5)-C(9)-C(11)	109.71(10)	C(8)-C(9)-C(11)	122.29(11)
O(5)-C(10)-O(6)	107.16(10)	O(6)-C(11)-C(9)	109.67(10)
C(12)-C(11)-O(6)	128.20(11)	C(12)-C(11)-C(9)	122.07(10)

C(11)-C(12)-C(13)	116.54(10)	C(7)-C(13)-C(12)	121.45(11)
C(6)-C(14)-H(14)	109.9(13)	C(15)-C(14)-C(6)	108.37(10)
C(15)-C(14)-H(14)	113.9(13)	C(26)-C(14)-C(6)	112.23(10)
C(26)-C(14)-H(14)	110.1(13)	C(26)-C(14)-C(15)	102.24(10)
C(16)-C(15)-C(14)	128.72(12)	C(16)-C(15)-C(20)	121.13(12)
C(20)-C(15)-C(14)	110.11(11)	C(15)-C(16)-C(17)	118.35(14)
C(18)-C(17)-C(16)	120.49(14)	C(19)-C(18)-C(17)	121.28(13)
C(18)-C(19)-C(20)	118.35(15)	C(15)-C(20)-C(21)	108.54(11)
C(19)-C(20)-C(15)	120.39(13)	C(19)-C(20)-C(21)	131.03(14)
C(22)-C(21)-C(20)	130.81(13)	C(22)-C(21)-C(26)	120.80(14)
C(26)-C(21)-C(20)	108.32(11)	C(21)-C(22)-C(23)	118.50(15)
C(24)-C(23)-C(22)	120.75(15)	C(23)-C(24)-C(25)	120.83(15)
C(26)-C(25)-C(24)	118.65(14)	C(21)-C(26)-C(14)	110.35(11)
C(25)-C(26)-C(14)	129.21(12)	C(25)-C(26)-C(21)	120.44(12)

Note: the absolute structure of compounds **1k** and **1o** was not determined unambiguously via X-ray analysis (see respective absolute structure parameter). For the determination of the absolute structure of arylglycine products **1** prepared in this work please see section 7 of this supporting information.

References

(1) Bendelsmith, A. J.; Kim, S. C.; Wasa, M.; Roche, S. P.; Jacobsen, E. N. Enantioselective Synthesis of α-Allyl Amino Esters via Hydrogen-Bond-Donor Catalysis. *J. Am. Chem. Soc.* **2019**, *141* (29), 11414–11419. https://doi.org/10.1021/jacs.9b05556.

(2) You, Y.; Zhang, L.; Cui, L.; Mi, X.; Luo, S. Catalytic Asymmetric Mannich Reaction with N-Carbamoyl Imine Surrogates of Formaldehyde and Glyoxylate. *Angew. Chem. Int. Ed.* **2017**, *56* (44), 13814–13818. https://doi.org/10.1002/anie.201707005.

(3) Maciá, E.; Foubelo, F.; Yus, M. Indium-Mediated Diastereoselective Allylation of N-Tert-Butanesulfinyl Imines Derived from α-Ketoesters. *Tetrahedron* **2016**, *72* (40), 6001–6010. https://doi.org/10.1016/j.tet.2016.07.020.

(4) Fan, B.; Trant, J. F.; Wong, A. D.; Gillies, E. R. Polyglyoxylates: A Versatile Class of Triggerable Self-Immolative Polymers from Readily Accessible Monomers. *J. Am. Chem. Soc.* **2014**, *136* (28), 10116–10123. https://doi.org/10.1021/ja504727u.

(5) Zhang, X.; Wang, M.; Ding, R.; Xu, Y.-H.; Loh, T.-P. Highly Enantioselective and Anti - Diastereoselective Catalytic Intermolecular Glyoxylate–Ene Reactions: Effect of the Geometrical Isomers of Alkenes. *Org. Lett.* **2015**, *17* (11), 2736–2739. https://doi.org/10.1021/acs.orglett.5b01151.

(6) Blaquiere, N.; Shore, D. G.; Rousseaux, S.; Fagnou, K. Decarboxylative Ketone Aldol Reactions: Development and Mechanistic Evaluation under Metal-Free Conditions. *J. Org. Chem.* **2009**, *74* (16), 6190–6198. https://doi.org/10.1021/jo901022j.

(7) Bloux, H.; Dahiya, A.; Hébert, A.; Fabis, F.; Schoenebeck, F.; Cailly, T. Base-Mediated Radio-Iodination of Arenes by Using Organosilane and Organogermane as Radiolabelling Precursors. *Chem. – A Eur. J.* **2023**, *29* (19). https://doi.org/10.1002/chem.202203366.

(8) Guo, S.; Ma, L.; Zhao, J.; Küçüköz, B.; Karatay, A.; Hayvali, M.; Yaglioglu, H. G.; Elmali, A. BODIPY Triads Triplet Photosensitizers Enhanced with Intramolecular Resonance Energy Transfer (RET): Broadband Visible Light Absorption and Application in Photooxidation. *Chem. Sci.* **2014**, *5* (2), 489–500. https://doi.org/10.1039/C3SC52323C.

(9) Liu, L.; Kim, H.; Xie, Y.; Farès, C.; Kaib, P. S. J.; Goddard, R.; List, B. Catalytic Asymmetric [4+2]-Cycloaddition of Dienes with Aldehydes. *J. Am. Chem. Soc.* **2017**, *139* (39), 13656–13659. https://doi.org/10.1021/jacs.7b08357.

(10) Gatzenmeier, T.; Turberg, M.; Yepes, D.; Xie, Y.; Neese, F.; Bistoni, G.; List, B. Scalable and Highly Diastereo- and Enantioselective Catalytic Diels–Alder Reaction of α,β-Unsaturated Methyl Esters. *J. Am. Chem. Soc.* **2018**, *140* (40), 12671–12676. https://doi.org/10.1021/jacs.8b07092.

(11) Kaib, P. S. J.; Schreyer, L.; Lee, S.; Properzi, R.; List, B. Extremely Active Organocatalysts Enable a Highly Enantioselective Addition of Allyltrimethylsilane to Aldehydes. *Angew. Chemie Int. Ed.* **2016**, *55* (42), 13200–13203. https://doi.org/10.1002/anie.201607828.

(12) Schwengers, S. A.; De, C. K.; Grossmann, O.; Grimm, J. A. A.; Sadlowski, N. R.; Gerosa, G. G.; List, B. Unified Approach to Imidodiphosphate-Type Brønsted Acids with Tunable Confinement and Acidity. *J. Am. Chem. Soc.* **2021**, *143* (36), 14835–14844. https://doi.org/10.1021/jacs.1c07067.

(13) Grossmann, O.; Maji, R.; Aukland, M. H.; Lee, S.; List, B. Catalytic Asymmetric Additions of Enol Silanes to In Situ Generated Cyclic, Aliphatic N-Acyliminium Ions. *Angew. Chem. Int. Ed.* **2022**, *61* (9), 1–6. https://doi.org/10.1002/anie.202115036.

(14) Gómez-Gallego, M.; Sierra, M. A. Kinetic Isotope Effects in the Study of Organometallic Reaction Mechanisms. *Chem. Rev.* **2011**, *111* (8), 4857–4963. https://doi.org/10.1021/cr100436k.

(15) Effenberger, F.; Maier, A. H. Changing the O Rtho/ P Ara Ratio in Aromatic Acylation Reactions by Changing Reaction Conditions: A Mechanistic Explanation from Kinetic Measurements 1. *J. Am. Chem. Soc.* **2001**, *123* (15), 3429–3433. https://doi.org/10.1021/ja0022066.

(16) Olah, G. A.; Kuhn, S. J.; Flood, S. H.; Hardie, B. A. Aromatic Substitution. XXII. 1a Acetylation of Benzene, Alkylbenzenes, and Halobenzenes with Methyloxocarbonium (Acetylium) Hexafluoro- and Hexachloroantimonate. *J. Am. Chem. Soc.* **1964**, *86* (11), 2203–2209. https://doi.org/10.1021/ja01065a020.

(17) Dowdy, D.; Gore, P. H.; Waters, D. N. The Friedel–Crafts Acetylation of Naphthalene in 1,2-Dichloroethane Solution. Kinetics and Mechanism. *J. Chem. Soc., Perkin Trans. 2* **1991**, No. 8, 1149–1159. https://doi.org/10.1039/P29910001149.

(18) Denney, D. B.; Klemchuk, P. P. Deuterium Isotope Effects in Some Friedel-Crafts Cyclizations of 2-(2-Deuteriophenyl)-Benzoyl Chloride. *J. Am. Chem. Soc.* **1958**, *80* (22), 6014–6016. https://doi.org/10.1021/ja01555a032.

(19) Liu, Y.; Meng, G.; Liu, R.; Szostak, M. Sterically-Controlled Intermolecular Friedel-Crafts Acylation with Twisted Amides: Via Selective N-C Cleavage under Mild Conditions. *Chem. Commun.* **2016**, *52* (41), 6841–6844. https://doi.org/10.1039/c6cc02324j.

(20) Nakane, R.; Kurihara, O.; Takematsu, A. Friedel-Crafts Isopropylation in Nonpolar Solvents. *J. Org. Chem.* **1971**, *36* (19), 2753–2756. https://doi.org/10.1021/jo00818a006.

(21) Olah, G. A.; Kuhn, S. J.; Flood, S. H. Aromatic Substitution. X. The AlCl3•CH3NO2-Catalyzed Benzylation of Benzene and N-Alkylbenzenes with Benzyl Chloride in Nitromethane Solution. *J. Am. Chem. Soc.* **1962**, *84* (9), 1688–1695. https://doi.org/10.1021/ja00868a039.

(22) Olah, G. A.; Kuhn, S. J.; Flood, S. H. Aromatic Substitution. VIII. 1 Mechanism of the Nitronium Tetrafluoroborate Nitration of Alkylbenzenes in Tetramethylene Sulfone Solution. Remarks on Certain Aspects of Electrophilic Aromatic Substitution 2. *J. Am. Chem. Soc.* **1961**, *83* (22), 4571–4580. https://doi.org/10.1021/ja01483a017.

(23) Zhu, H.; Meyer, M. P. Cationic Intermediates in Friedel-Crafts Acylation: Structural Information from Theory and Experiment. *Chem. Commun.* **2011**, *47* (1), 409–411. https://doi.org/10.1039/c0cc02286a.

(24) Pracht, P.; Caldeweyher, E.; Ehlert, S.; Grimme, S. *Chemrxiv* **2019**. https://doi.org/10.26434/chemrxiv.8326202.v1.

(25) Pracht, P.; Bohle, F.; Grimme, S. Automated Exploration of the Low-Energy Chemical Space with Fast Quantum Chemical Methods. *Phys. Chem. Chem. Phys.* **2020**, *22* (14), 7169–7192. https://doi.org/10.1039/C9CP06869D.

(26) Bannwarth, C.; Ehlert, S.; Grimme, S. GFN2-XTB—An Accurate and Broadly Parametrized Self-Consistent Tight-Binding Quantum Chemical Method with Multipole Electrostatics and Density-Dependent Dispersion Contributions. *J. Chem. Theory Comput.* **2019**, *15* (3), 1652–1671. https://doi.org/10.1021/acs.jctc.8b01176.

(27) Neese, F. Software Update: The <scp>ORCA</Scp> Program System—Version 5.0. *WIREs Comput. Mol. Sci.* **2022**, *12* (5). https://doi.org/10.1002/wcms.1606.

(28) Perdew, J. P.; Burke, K.; Ernzerhof, M. Generalized Gradient Approximation Made Simple. *Phys. Rev. Lett.* **1996**, *77* (18), 3865–3868. https://doi.org/10.1103/PhysRevLett.77.3865.

(29) Grimme, S.; Antony, J.; Ehrlich, S.; Krieg, H. A Consistent and Accurate Ab Initio Parametrization of Density Functional Dispersion Correction (DFT-D) for the 94 Elements H-Pu. *J. Chem. Phys.* **2010**, *132* (15), 154104. https://doi.org/10.1063/1.3382344.

(30) Grimme, S.; Ehrlich, S.; Goerigk, L. Effect of the Damping Function in Dispersion Corrected Density Functional Theory. *J. Comput. Chem.* **2011**, *32* (7), 1456–1465. https://doi.org/10.1002/jcc.21759.

(31) Weigend, F.; Ahlrichs, R. Balanced Basis Sets of Split Valence, Triple Zeta Valence and Quadruple Zeta Valence Quality for H to Rn: Design and Assessment of Accuracy. *Phys. Chem. Chem. Phys.* **2005**, *7* (18), 3297. https://doi.org/10.1039/b508541a.

(32) Eichkorn, K.; Treutler, O.; Öhm, H.; Häser, M.; Ahlrichs, R. Auxiliary Basis Sets to Approximate Coulomb Potentials. *Chem. Phys. Lett.* **1995**, *240* (4), 283–290. https://doi.org/10.1016/0009-2614(95)00621-A.

(33) Neese, F. An Improvement of the Resolution of the Identity Approximation for the Formation of the Coulomb Matrix. *J. Comput. Chem.* **2003**, *24* (14), 1740–1747. https://doi.org/10.1002/jcc.10318.

(34) Weigend, F. Accurate Coulomb-Fitting Basis Sets for H to Rn. *Phys. Chem. Chem. Phys.* **2006**, *8* (9), 1057–1065. https://doi.org/10.1039/b515623h.

(35) Zhao, Y.; Truhlar, D. G. The M06 Suite of Density Functionals for Main Group Thermochemistry, Thermochemical Kinetics, Noncovalent Interactions, Excited States, and Transition Elements: Two New Functionals and Systematic Testing of Four M06 Functionals and 12 Other Functionals. *Theor. Chem. Acc.* **2008**, *119* (5–6), 525–525. https://doi.org/10.1007/s00214-007-0401-8.

(36) *“otherm.py,” can be found under https://github.com/duartegroup/otherm*.

(37) Grimme, S. Supramolecular Binding Thermodynamics by Dispersion-Corrected Density Functional Theory. *Chem. - A Eur. J.* **2012**, *18* (32), 9955–9964. https://doi.org/10.1002/chem.201200497.
